# Clinical radiomics-based machine learning versus three-dimension convolutional neural network analysis for differentiation of thymic epithelial tumors from other prevascular mediastinal tumors on chest computed tomography scan

**DOI:** 10.3389/fonc.2023.1105100

**Published:** 2023-04-18

**Authors:** Chao-Chun Chang, En-Kuei Tang, Yu-Feng Wei, Chia-Ying Lin, Fu-Zong Wu, Ming-Ting Wu, Yi-Sheng Liu, Yi-Ting Yen, Mi-Chia Ma, Yau-Lin Tseng

**Affiliations:** ^1^ Division of Thoracic Surgery, Department of Surgery, National Cheng Kung University Hospital, College of Medicine, National Cheng Kung University, Tainan, Taiwan; ^2^ Division of Thoracic Surgery, Department of Surgery, Kaohsiung Veterans General Hospital, Kaohsiung, Taiwan; ^3^ School of Medicine for International Students, College of Medicine, I-Shou University, Kaohsiung, Taiwan; ^4^ Division of Chest Medicine, Department of Internal Medicine, E-Da Cancer Hospital, Kaohsiung, Taiwan; ^5^ Department of Medical Imaging, National Cheng Kung University Hospital, College of Medicine, National Cheng Kung University, Tainan, Taiwan; ^6^ Department of Radiology, Kaohsiung Veterans General Hospital, Kaohsiung, Taiwan; ^7^ Faculty of Clinical Medicine, National Yang Ming Chiao Tung University, Taipei, Taiwan; ^8^ Institute of Education, National Sun Yat-sen University, Kaohsiung, Taiwan; ^9^ School of Medicine, National Yang Ming Chiao Tung University, Taipei, Taiwan; ^10^ Institute of Clinical Medicine, National Yang Ming Chiao Tung University, Taipei, Taiwan; ^11^ Division of Trauma and Acute Care Surgery, Department of Surgery, National Cheng Kung University Hospital, College of Medicine, National Cheng Kung University, Tainan, Taiwan; ^12^ Department of Statistics and Institute of Data Science, National Cheng Kung University, Tainan, Taiwan

**Keywords:** radiomics, convolutional neural networks, deep learning, machine learning, prevascular mediastinal tumor

## Abstract

**Purpose:**

To compare the diagnostic performance of radiomic analysis with machine learning (ML) model with a convolutional neural network (CNN) in differentiating thymic epithelial tumors (TETs) from other prevascular mediastinal tumors (PMTs).

**Methods:**

A retrospective study was performed in patients with PMTs and undergoing surgical resection or biopsy in National Cheng Kung University Hospital, Tainan, Taiwan, E-Da Hospital, Kaohsiung, Taiwan, and Kaohsiung Veterans General Hospital, Kaohsiung, Taiwan between January 2010 and December 2019. Clinical data including age, sex, myasthenia gravis (MG) symptoms and pathologic diagnosis were collected. The datasets were divided into UECT (unenhanced computed tomography) and CECT (enhanced computed tomography) for analysis and modelling. Radiomics model and 3D CNN model were used to differentiate TETs from non-TET PMTs (including cyst, malignant germ cell tumor, lymphoma and teratoma). The macro F1-score and receiver operating characteristic (ROC) analysis were performed to evaluate the prediction models.

**Result:**

In the UECT dataset, there were 297 patients with TETs and 79 patients with other PMTs. The performance of radiomic analysis with machine learning model using LightGBM with Extra Tree (macro F1-Score = 83.95%, ROC-AUC = 0.9117) had better performance than the 3D CNN model (macro F1-score = 75.54%, ROC-AUC = 0.9015). In the CECT dataset, there were 296 patients with TETs and 77 patients with other PMTs. The performance of radiomic analysis with machine learning model using LightGBM with Extra Tree (macro F1-Score = 85.65%, ROC-AUC = 0.9464) had better performance than the 3D CNN model (macro F1-score = 81.01%, ROC-AUC = 0.9275).

**Conclusion:**

Our study revealed that the individualized prediction model integrating clinical information and radiomic features using machine learning demonstrated better predictive performance in the differentiation of TETs from other PMTs at chest CT scan than 3D CNN model.

## Introduction

Prevascular mediastinal tumor (PMT) is relatively uncommon, making up less than 1% of all solid tumors (1). PMT consists of a wide variety of entities, including thymic epithelial tumor (TET), lymphoma, germ cell tumor, ectopic thyroid, and cyst, among which TET is the most frequently encountered (2). The NCCN guidelines suggested that patients with clinically resectable TETs undergo upfront surgical resection instead of preoperative transpleural biopsy to avoid converting stage I thymoma to stage IV thymoma by spreading tumor within the pleural space (3). Chest computed tomography (CT) scan is the standard assessment for PMT. It was reported that the accuracy rate of PMT interpretation *via* traditional radiographic features on CT scan reaches as high as 61% in experienced radiologists, leaving much room for improvements in the era of advanced technology (4).

The applications of radiomics in diagnostic medicine and outcome analysis have been increasingly proposed lately (5, 6). By combining image-filtering and feature-extraction methods, it is possible to extract a large number of high-order radiomic features from CT images (5). Studies have shown significant radiomic parameters such as skewness, kurtosis, and entropy, correlated with thymic tumor histology (7, 8). Redundancy is often the scenario in highly dimensional data, and classification model could be developed only through proper feature selection and proper machine learning (9).

Convolutional neural networks (CNNs) are a class of deep learning (DL) models that combine imaging filters with artificial neural networks through a series of successive linear and nonlinear layers (10, 11). CNN is far more data hungry because of its millions of learnable parameters to estimate, and therefore is more computationally expensive, resulting in the requirement of graphical processing units (GPUs) for model training. The major drawback in the application of 3D deep learning on medical images is its dependency on data availability and high computational cost (12). With powerful GPUs becoming increasingly available, we have seen exponential growth in the applications of 3D deep learning in different medical image modalities (11).

Nonetheless, with the low incidence of tumor occurrence and the resultant limited radiographic data and information, it is yet to be clarified if 3D CNN out-performs radiomics with ML in differentiating various kinds of mediastinal tumor. Our study aimed to compare the model using radiomics with traditional machine learning with 3D CNN model in differentiating TETs from PMTs, thus providing a prediction tool and the opportunity of improvement on the decisions for invasive diagnostic or treatment modalities.

## Materials and methods

### Study population

**Figure 1:**
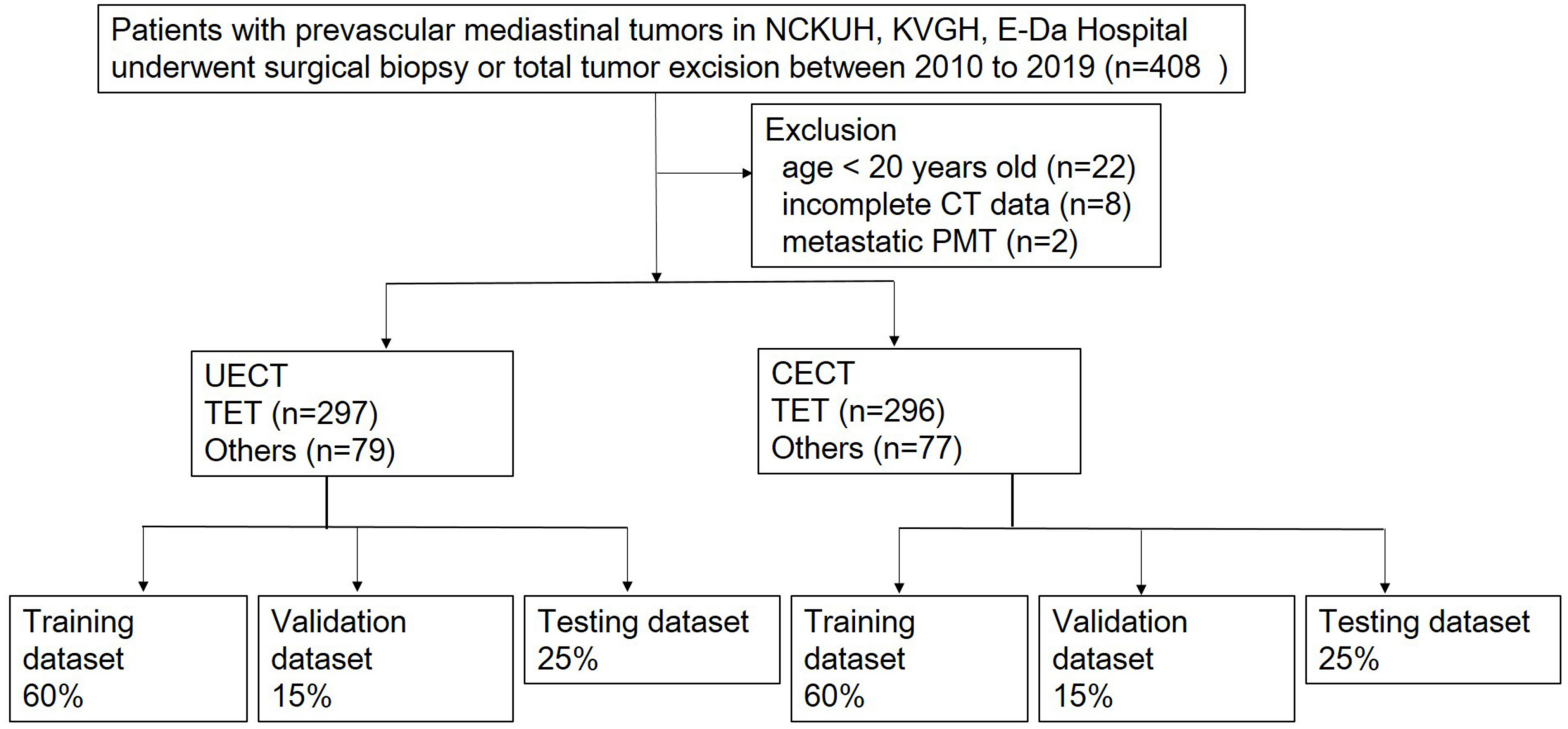
Flowchart of patients inclusion. NCKUH, National Cheng Kung University Hospital; KVGH, Kaohsiung Veteran General Hospital.

A retrospective study was performed in patients with PMTs and undergoing surgical resection or biopsy in National Cheng-Kung University Hospital, Tainan, Taiwan, E-Da Hospital, Kaohsiung, Taiwan, and Kaohsiung Veterans General Hospital, Kaohsiung, Taiwan between January 2010 and December 2019. Informed consent was waived because the study was retrospective, and it was respectively approved by the Institutional Review Board of National Cheng Kung University Hospital (A-ER-111-211), E-Da Hospital (EMRP-110-145), and Kaohsiung Veteran General Hospital (VGHKS19-CT6-08). Exclusion criteria were patients younger than 20-year-old, missing imaging data, or metastatic prevascular mediastinal tumors. The patients younger than 20-year-old were excluded because the most common types of tumors in this age group are lymphomas and malignant germ cell tumors, and the rare occurrence of thymic epithelial tumors (TET) does not have much impact on histological classification of tumors (13). Clinical data including age, sex, myasthenia gravis (MG) symptoms and pathologic diagnosis were collected. We divided our dataset into UECT (unenhanced computed tomography) and CECT (enhanced computed tomography) for analysis and modelling. Radiomic model and 3D CNN model were adopted respectively to differentiate TETs from non-TET PMTs (including cyst, malignant germ cell tumor, lymphoma and teratoma). The flowchart of patient inclusion was shown in **Figure 1**.

### Image acquisition

All CT images were obtained using Siemens SOMATOM Definition Flash, Siemens SOMATOM Definition AS, Siemens SOMATOM Sensation 16, GE Optima CT660, GE Revolution CT, GE Bright Speed Elite, GE light speed VCT, and TOSHIBA CT64-TSX-01A64. The CT protocols were as follows: 120 kVp; tube current, 150–200 mAs with automatic tube current modulation. The section thickness ranged between 0.7 mm and 1.5 mm, and the image size was 512 × 512 pixels. The detailed protocol and contrast materials are summarized in **Table E1**. Three patients received solely non-enhanced CT scan due to renal function impairment, while the other patients received both non-enhanced and enhanced CT scan. Contrast enhanced images were obtained after intravenous administration of contrast medium (injection dose 60-120 mL at a rate of 1.5-3 ml/s) followed by a 20 ml saline flush. Contrast enhanced images were obtained 90s after contrast agent administration. All images were reconstructed into 5-mm sections.

### Tumor segmentation and image preprocessing

CT images were imported into the open-source software 3D Slicer 4.10.2 and the tumors were then contoured manually by one of two observers (C.Y.L. a radiologist with 9 years of experience and C.C.C, a thoracic surgeon with 9 years of experience) blinded to patient diagnosis using the built-in paint tool (14). The delineation of tumor at UECT and CECT was performed in the mediastinal setting (window level, 50 HU; window width, 350 HU) on the axial CT plane. Consensus was reached by discussion if the interobserver variability was apparent. For normalization, all CT voxels were resampled to 1 mm3 using a cubic interpolation.

### Radiomics feature extraction, selection and model building

**Figure 2:**
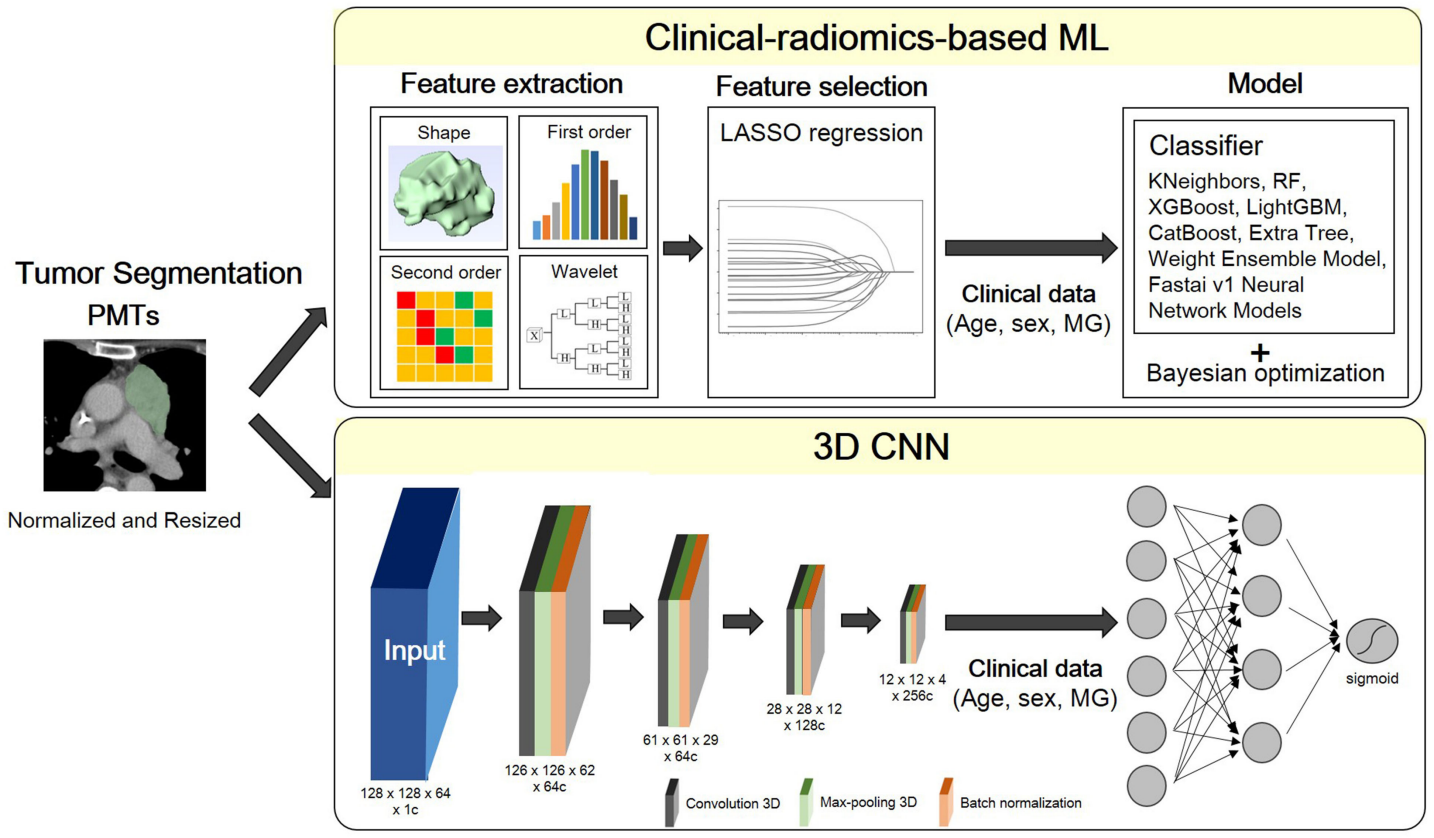
Flowchart of the proposed scheme. PMT, prevascular tumor; LASSO, least absolute shrinkage and selection operator; MG, myasthenia gravis; RF, random forest; XGBoost, extreme gradient boosting; LightGBM, light gradient boosting machine.

The global framework showing the radiomic analysis process is shown in **Figure 2**. The whole PMTs in each CT examination served as VOIs, from which 3D radiomic features were extracted using the open-source platform PyRadiomics (15). A total of 851 radiomic features, including 14 shape features, 18 intensity histogram features, 74 texture features, and 745 wavelet features were extracted for further analysis.

A multivariate logistic regression model was developed using the least absolute shrinkage and selection operator (LASSO) with L1 penalty to filter the features to reduce the redundancy of the features. The features with non-zero coefficients at optimized hyperparameter lambda were selected and used in ML. Relevant clinical information including age, sex and MG symptoms were also input as feature vectors in ML.

In combination with feature-selection method, eight ML classifiers were used to differentiate thymoma from other PMTs: KNeighbors, random forest (RF), extreme gradient boosting (XGBoost), Light Gradient Boosting Machine (LightGBM), CatBoost, Extra Tree, and Weight Ensemble Model (WeightEnsemble_L2). A Bayesian optimization algorithm (BOA) was applied to optimize the hyperparameters of these models. The flowchart of the proposed scheme was illustrated in **Figure 2**.

### 3D convolutional neural networks model

A fully convolutional neural network (CNN), as described in a recently published previous work was used (16). ROI patches were extracted from each CT image by defining a bounding box that enclosed each previously defined ROI. All patches were resized to 128 × 128 × 64. Owing to the limited training data, we applied random rotation (-30°~30°) for training data augmentation. As radiomics-based machine learning model, the clinical information including age, sex and MG symptoms were also input as feature vectors in this neural network model.

All the models are implemented in Python 3.8.9 based on tensorflow 2.8.0 and trained on 1 Tesla V100-DGXS-32GB. The loss function during training was the binary cross-entropy metric and was optimized using an Adam optimizer with a batch size of 2, and an initial learning rate of 10-5. Early stopping was employed to prevent overfitting, and training was stopped once model performance stops improving on a validation dataset after 350 constitutive training epochs (maximum epoch value = 1000). The best model observed during training would be the output model. The flowchart of the proposed scheme was illustrated in **Figure 2**.

### Statistical analyses

Continuous variables were compared using the Student t-test, and categorical variables were compared using the chi-square test. P values of < 0.05 were considered statistically significant.

Clinical information (age, sex and presence of MG symptom) was added into radiomic model and 3D CNN model. Each dataset was randomly split into training, validation and testing sets in the ratio of 60:15:25. The models were evaluated through repeated random sub-sampling validation.

The accuracy, macro precision, macro recall, and macro F1-score for each dataset were calculated and determined to verify the performance of the different models. The macro F1-score reflected the effectiveness on small classes and is an effective evaluation metric for an imbalanced dataset. Receiver operating characteristic (ROC) analysis was performed, and the area under the curve (AUC) was used to evaluate the prediction models. The analysis was performed using python 3.8.9 with scikit-learn 1.0.1, autogluon 0.2.0, and statsmodels 0.13.1.

## Result

### Basic clinical characteristics

The clinicopathological characteristics of patients in our study are shown in **Table 1**. In UECT dataset, there were 297 patients with TETs and 79 patients with other PMTs. In CECT dataset, there were 296 patients with TETs and 77 patients with other PMTs. There was significant difference in age and MG symptoms in UECT dataset [TETs *vs*. other PMTs: 61.70 ± 12.99 *vs*. 51.19 ± 17.74, p < 0.001; 87 (29.3%) *vs*. 4 (5.1%), p < 0.001]. In TETs, there were 19.2% thymic carcinoma and 80.8% thymomas. In other PMTs, there were mostly cyst (54.4%). In CECT dataset, there was significant difference in age and MG symptoms [TETs *vs*. other PMTs: 61.46 ± 13.06 *vs*. 49.26 ± 17.76, p < 0.001; 87 (29.4%) *vs* 4 (5.2%), p < 0.001]. In TETs, there were 19.3% thymic carcinoma and 80.7% thymomas. In other PMTs, there were mostly cyst (53.2%).

**Table 1:** Baseline characteristics of patients included for analysis.

Variables	UECT			CECT		
	TET (n=297)	Others (n=79)	*p* value	TET (n=296)	Others (n=77)	*p* value
Sex (male)	135 (45.5%)	36 (45.6%)	1	134 (45.3%)	36 (46.8%)	0.898
Age (y)*	61.70 ± 12.99	51.19 ± 17.74	<0.001	61.46 ± 13.06	49.26 ± 17.76	<0.001
Myasthenia gravis	87 (29.3%)	4 (5.1%)	<0.001	87 (29.4%)	4 (5.2%)	<0.001
Pathology						
Thymoma	240 (80.8%)			239 (80.7%)		
Thymic carcinoma	57 (19.2%)			57 (19.3%)		
Cyst		43 (54.4%)			41 (53.2%)	
Malignant germ cell tumor		10 (12.7%)			10 (13.0%)	
Lymphoma		11 (13.9%)			11 (14.3%)	
Teratoma		15 (19.0%)			(19.5%)	

Except where indicated, data are numbers of patients, with percentages in parentheses.

* Data are means ± SDs.

### Radiomics feature selection and optimal signature construction

After performing tumor segmentation in the included patients, 851 radiomics features were extracted (**Supplementary Tables 1**, **2**). The top five feature selection using LASSO logistic regression with different values of lambda was shown in **Tables 2**, **3**, and the top 20 feature selection was showed in **Supplementary Tables 3**, **4**.

**Table 2:** Top five variable feature selection performed by LASSO Logistic Regression at various lambda value using UECT.

Feature Selection	Lambda	Selected Variable		
Selection 1	0.04863	original_firstorder_Median	wavelet-LLH_glcm_MCC	wavelet-LLH_glrlm_RunEntropy
		wavelet-LLH_glszm_LargeAreaLowGrayLevelEmphasis	wavelet-HLH_ngtdm_Busyness	wavelet-LLL_firstorder_RootMeanSquared
		wavelet-LLL_glszm_LargeAreaEmphasis		
Selection 2	0.03511	original_firstorder_Median	original_glrlm_RunEntropy	wavelet-LLH_glcm_Correlation
		wavelet-LLH_glcm_MCC	wavelet-LLH_glrlm_RunEntropy	wavelet-LLH_glrlm_ShortRunLowGrayLevelEmphasis
		wavelet-LLH_glszm_LargeAreaLowGrayLevelEmphasis	wavelet-LHL_glszm_SmallAreaEmphasis	wavelet-LHH_glrlm_RunEntropy
		wavelet-HLH_ngtdm_Busyness	wavelet-HHL_glszm_GrayLevelNonUniformityNormalized	wavelet-LLL_firstorder_RootMeanSquared
		wavelet-LLL_glszm_LargeAreaEmphasis		
Selection 3	0.02535	original_shape_Sphericity	original_firstorder_Median	original_glrlm_RunEntropy
		wavelet-LLH_glcm_Correlation	wavelet-LLH_glcm_MCC	wavelet-LLH_glrlm_RunEntropy
		wavelet-LLH_glrlm_ShortRunLowGrayLevelEmphasis	wavelet-LLH_glszm_LargeAreaLowGrayLevelEmphasis	wavelet-LHL_firstorder_Skewness
		wavelet-LHL_glszm_SmallAreaEmphasis	wavelet-LHH_glcm_ClusterShade	wavelet-LHH_glrlm_RunEntropy
		wavelet-HLH_ngtdm_Busyness	wavelet-HHL_glszm_GrayLevelNonUniformityNormalized	wavelet-HHH_glszm_SizeZoneNonUniformityNormalized
		wavelet-HHH_glszm_SmallAreaEmphasis	wavelet-LLL_firstorder_RootMeanSquared	wavelet-LLL_glszm_LargeAreaEmphasis
Selection 4	0.01831	original_shape_Sphericity	original_firstorder_Median	original_glrlm_RunEntropy
		wavelet-LLH_glcm_Correlation	wavelet-LLH_glcm_MCC	wavelet-LLH_glrlm_RunEntropy
		wavelet-LLH_glrlm_ShortRunLowGrayLevelEmphasis	wavelet-LLH_glszm_LowGrayLevelZoneEmphasis	wavelet-LHL_glszm_SmallAreaEmphasis
		wavelet-LHH_firstorder_Mean	wavelet-LHH_glcm_MaximumProbability	wavelet-LHH_glrlm_RunEntropy
		wavelet-LHH_ngtdm_Busyness	wavelet-HLL_glszm_ZoneVariance	wavelet-HLH_glcm_MCC
		wavelet-HLH_glszm_SmallAreaLowGrayLevelEmphasis	wavelet-HLH_ngtdm_Busyness	wavelet-HHL_glszm_GrayLevelNonUniformityNormalized
		wavelet-HHH_glszm_SizeZoneNonUniformityNormalized	wavelet-HHH_glszm_SmallAreaEmphasis	wavelet-LLL_firstorder_RootMeanSquared
Selection 5	0.01322	original_shape_Sphericity	original_shape_SurfaceVolumeRatio	original_firstorder_Median
		original_firstorder_c	original_glrlm_RunEntropy	wavelet-LLH_firstorder_Kurtosis
		wavelet-LLH_glcm_Correlation	wavelet-LLH_glcm_MCC	wavelet-LLH_glcm_MaximumProbability
		wavelet-LLH_glrlm_ShortRunLowGrayLevelEmphasis	wavelet-LLH_glszm_LowGrayLevelZoneEmphasis	wavelet-LHL_firstorder_Skewness
		wavelet-LHL_glszm_SmallAreaEmphasis	wavelet-LHH_firstorder_Mean	wavelet-LHH_glcm_MaximumProbability
		wavelet-LHH_glrlm_RunEntropy	wavelet-LHH_glszm_SizeZoneNonUniformityNormalized	wavelet-LHH_ngtdm_Busyness
		wavelet-HLL_firstorder_Maximum	wavelet-HLL_glszm_ZoneVariance	wavelet-HLH_glcm_MCC
		wavelet-HLH_glcm_MaximumProbability	wavelet-HLH_glszm_SmallAreaLowGrayLevelEmphasis	wavelet-HLH_ngtdm_Busyness
		wavelet-HHL_glcm_Idmn	wavelet-HHL_glszm_GrayLevelNonUniformityNormalized	wavelet-HHH_glrlm_ShortRunLowGrayLevelEmphasis
		wavelet-HHH_glszm_SizeZoneNonUniformityNormalized	wavelet-HHH_glszm_SmallAreaEmphasis	wavelet-LLL_firstorder_RootMeanSquared
		wavelet-LLL_glcm_MaximumProbability		

**Table 3:** Top five variable feature selection performed by Lasso Logistic Regression at various lambda value using CECT.

Feature Selection	Lambda	Selected Variable		
Selection 1	0.04863	wavelet-LHH_firstorder_Mean	wavelet-LHH_glcm_SumEntropy	wavelet-LHH_gldm_LargeDependenceEmphasis
		wavelet-LHH_ngtdm_Busyness	wavelet-HLH_glszm_SizeZoneNonUniformityNormalized	wavelet-HLH_ngtdm_Busyness
		wavelet-LLL_firstorder_Median		
Selection 2	0.03511	wavelet-LHL_firstorder_Mean	wavelet-LHH_firstorder_Mean	wavelet-LHH_glcm_SumEntropy
		wavelet-LHH_glrlm_RunLengthNonUniformityNormalized	wavelet-LHH_glrlm_RunPercentage	wavelet-LHH_ngtdm_Busyness
		wavelet-HLL_glszm_LargeAreaHighGrayLevelEmphasis	wavelet-HLH_glszm_SizeZoneNonUniformityNormalized	wavelet-HLH_ngtdm_Busyness
		wavelet-HHL_glcm_Imc1	wavelet-HHL_glcm_InverseVariance	wavelet-LLL_firstorder_Median
Selection 3	0.02535	wavelet-LHL_firstorder_Mean	wavelet-LHL_firstorder_Skewness	wavelet-LHH_firstorder_Mean
		wavelet-LHH_glcm_JointEnergy	wavelet-LHH_glcm_SumEntropy	wavelet-LHH_glrlm_RunPercentage
		wavelet-LHH_glszm_SizeZoneNonUniformityNormalized	wavelet-LHH_ngtdm_Busyness	wavelet-HLL_firstorder_90Percentile
		wavelet-HLL_glszm_LargeAreaHighGrayLevelEmphasis	wavelet-HLH_glszm_SizeZoneNonUniformityNormalized	wavelet-HLH_ngtdm_Busyness
		wavelet-HHL_glcm_Imc1	wavelet-HHL_glcm_InverseVariance	wavelet-HHL_glcm_MCC
		wavelet-LLL_firstorder_Median		
Selection 4	0.01831	wavelet-LLH_glszm_GrayLevelVariance	wavelet-LLH_glszm_LowGrayLevelZoneEmphasis	wavelet-LHL_firstorder_Mean
		wavelet-LHL_firstorder_Skewness	wavelet-LHL_glcm_InverseVariance	wavelet-LHH_firstorder_Mean
		wavelet-LHH_glcm_Imc2	wavelet-LHH_glcm_JointEnergy	wavelet-LHH_glcm_SumEntropy
		wavelet-LHH_glrlm_RunLengthNonUniformityNormalized	wavelet-LHH_glrlm_RunPercentage	wavelet-LHH_glszm_SizeZoneNonUniformityNormalized
		wavelet-LHH_ngtdm_Busyness	wavelet-HLL_firstorder_90Percentile	wavelet-HLL_glcm_Correlation
		wavelet-HLH_glszm_SizeZoneNonUniformityNormalized	wavelet-HLH_ngtdm_Busyness	wavelet-HHL_glcm_Imc1
		wavelet-HHL_glcm_InverseVariance	wavelet-HHL_glcm_MCC	wavelet-HHL_glszm_LargeAreaHighGrayLevelEmphasis
		wavelet-HHL_glszm_SmallAreaLowGrayLevelEmphasis	wavelet-LLL_firstorder_Median	
Selection 5	0.01322	original_shape_Sphericity	wavelet-LLH_glszm_GrayLevelVariance	wavelet-LLH_glszm_LowGrayLevelZoneEmphasis
		wavelet-LHL_firstorder_Maximum	wavelet-LHL_firstorder_Mean	wavelet-LHL_firstorder_Median
		wavelet-LHL_firstorder_Skewness	wavelet-LHL_glcm_InverseVariance	wavelet-LHH_firstorder_Mean
		wavelet-LHH_glcm_Imc2	wavelet-LHH_glcm_JointEnergy	wavelet-LHH_glcm_SumEntropy
		wavelet-LHH_glrlm_RunLengthNonUniformityNormalized	wavelet-LHH_glrlm_RunPercentage	wavelet-LHH_glszm_SizeZoneNonUniformityNormalized
		wavelet-LHH_ngtdm_Busyness	wavelet-HLL_firstorder_90Percentile	wavelet-HLL_glcm_Correlation
		wavelet-HLH_glcm_MCC	wavelet-HLH_glrlm_LongRunLowGrayLevelEmphasis	wavelet-HLH_glszm_GrayLevelNonUniformityNormalized
		wavelet-HLH_glszm_SizeZoneNonUniformityNormalized	wavelet-HLH_ngtdm_Busyness	wavelet-HHL_glcm_Imc1
		wavelet-HHL_glcm_InverseVariance	wavelet-HHL_glcm_MCC	wavelet-HHL_glcm_MaximumProbability
		wavelet-HHL_glszm_LargeAreaHighGrayLevelEmphasis	wavelet-HHL_glszm_SmallAreaLowGrayLevelEmphasis	wavelet-HHH_firstorder_Median
		wavelet-LLL_firstorder_Median		

**Figure 3:**
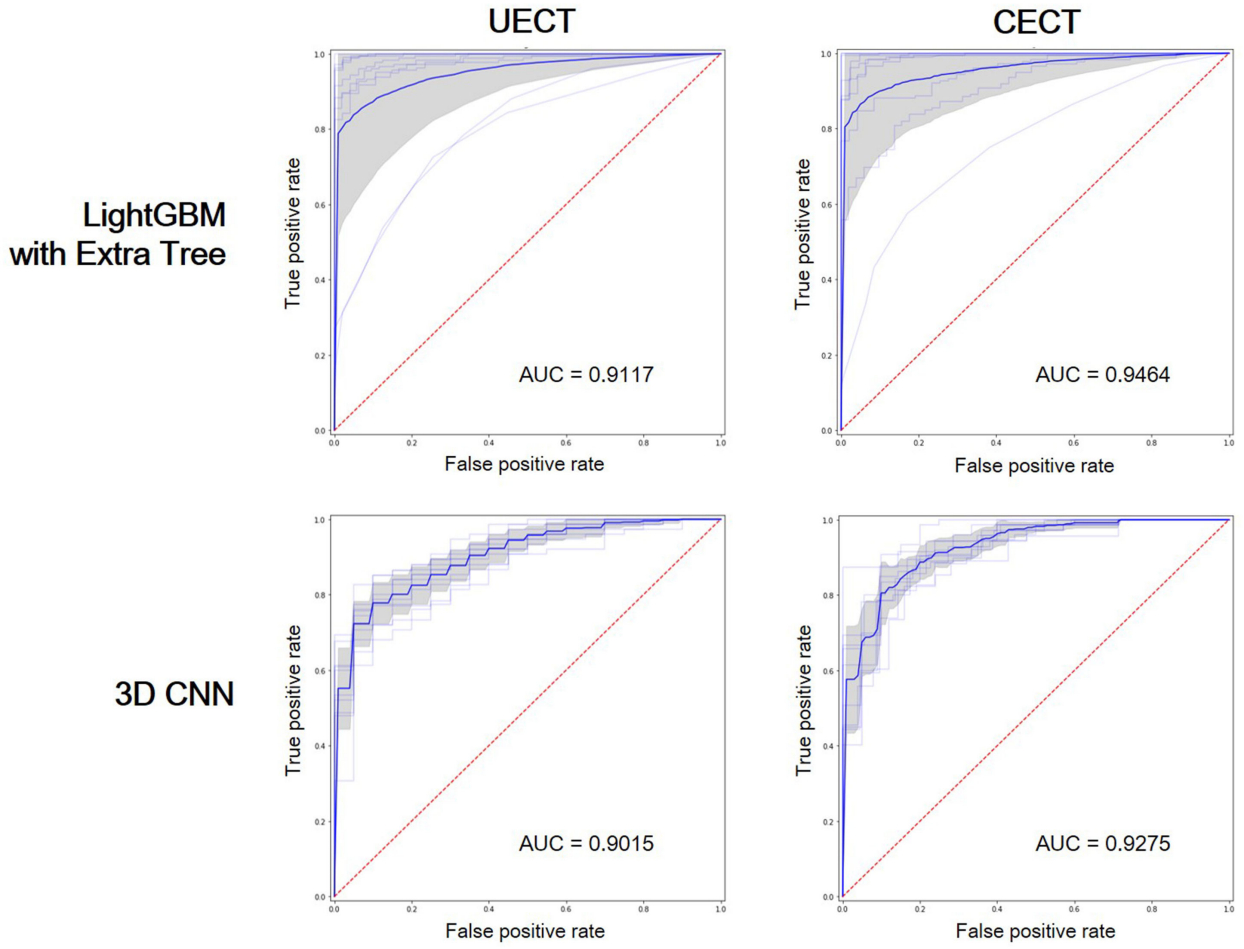
Receiver operating characteristic (ROC) curves showed the performance of LightGBM with Extra Tree and 3D CNN on UECT and CECT. AUC, area under curve.

The result of best feature selection using various machine learning methods was demonstrated in **Table 4**. In UECT dataset, LightGBM with Extra Tree using features in selection_5 had best performance (macro F1-Score = 83.95%, accuracy = 89.99%). The ROC curve was shown in **Figure 3** with AUC = 0.9117. In CECT dataset, LightGBM with Extra Tree using features in selection_4 had best performance (Macro F1-Score = 85.65%、accuracy = 91.15%). The ROC curve was shown in **Figure 3** with AUC = 0.9464. The results of Bayesian optimization of different models and various feature selection in UECT and CECT were showed in **Supplementary Tables 5**, **6**.

**Table 4:** The best result of radiomics models and 3D CNN model to differentiate thymoma from other prevascular mediastinal tumors.

	Feature selection	Macro F1-Score	Macro Precision	Macro Recall	Accuracy
UECT					
CatBoost	Selection 4	0.8121	0.8686	0.7813	0.8882
ExtraTrees with Entropy	Selection 1	0.7921	0.8519	0.7606	0.8786
ExtraTrees with Gini	Selection 1	0.7985	0.8595	0.7663	0.8818
Kneighbors with Distance weights	All feature	0.6128	0.6599	0.6015	0.7861
Kneighbors with Uniform weights	All feature	0.6135	0.6623	0.6015	0.7861
LightGBM	Selection 1	0.8258	0.8586	0.8052	0.8914
LightGBM with Extra Trees	Selection 5	0.8395	0.8773	0.816	0.8999
NeuralNetFastAI	Selection 9	0.833	0.835	0.8376	0.8851
Random Forest with Entropy	Selection 8	0.7952	0.8569	0.7631	0.8797
Random Forest with Gini	Selection 4	0.7984	0.8567	0.7685	0.8797
WeightedEnsemble_L2	Selection 9	0.8328	0.8605	0.8145	0.8946
XGBoost	Selection 1	0.8061	0.8305	0.7891	0.8775
3D CNN		0.7554	0.7679	0.7531	0.8416
CECT					
CatBoost	Selection 4	0.8374	0.8824	0.8111	0.9027
ExtraTrees with Entropy	Selection 1	0.796	0.8747	0.7595	0.8856
ExtraTrees with Gini	Selection 1	0.7981	0.8781	0.7602	0.8867
Kneighbors with Distance weights	All feature	0.6344	0.7036	0.6189	0.8059
Kneighbors with Uniform weights	All feature	0.6257	0.6865	0.6129	0.7985
LightGBM	Selection 2	0.8533	0.8806	0.836	0.9081
LightGBM with Extra Trees	Selection 4	0.8565	0.8889	0.8353	0.9115
NeuralNetFastAI	Selection 7	0.8388	0.8351	0.8564	0.8854
Random Forest with Entropy	Selection 3	0.8328	0.8932	0.8017	0.9039
Random Forest with Gini	Selection 3	0.8344	0.8892	0.8029	0.9027
WeightedEnsemble_L2	Selection 5	0.8506	0.8763	0.8341	0.9072
XGBoost	Selection 3	0.8496	0.8601	0.8432	0.9029
3D CNN		0.8101	0.8178	0.8082	0.8673

### 3D CNN analysis

The result of 3D CNN classification was shown in **Table 4**. In UECT dataset, macro F1-score was 75.54%, accuracy 84.16%. The ROC curve was shown in **Figure 3** with AUC = 0.9015. In CECT dataset, macro F1-score was 81.01%, accuracy 86.73%. The ROC curve was shown in **Figure 3** with AUC = 0.9275. Because we used repeated random sub-sampling validation method for 10 times, the total training time was 14.5 hours in UECT dataset and 14.1 hours in CECT dataset.

**Figure 4:**
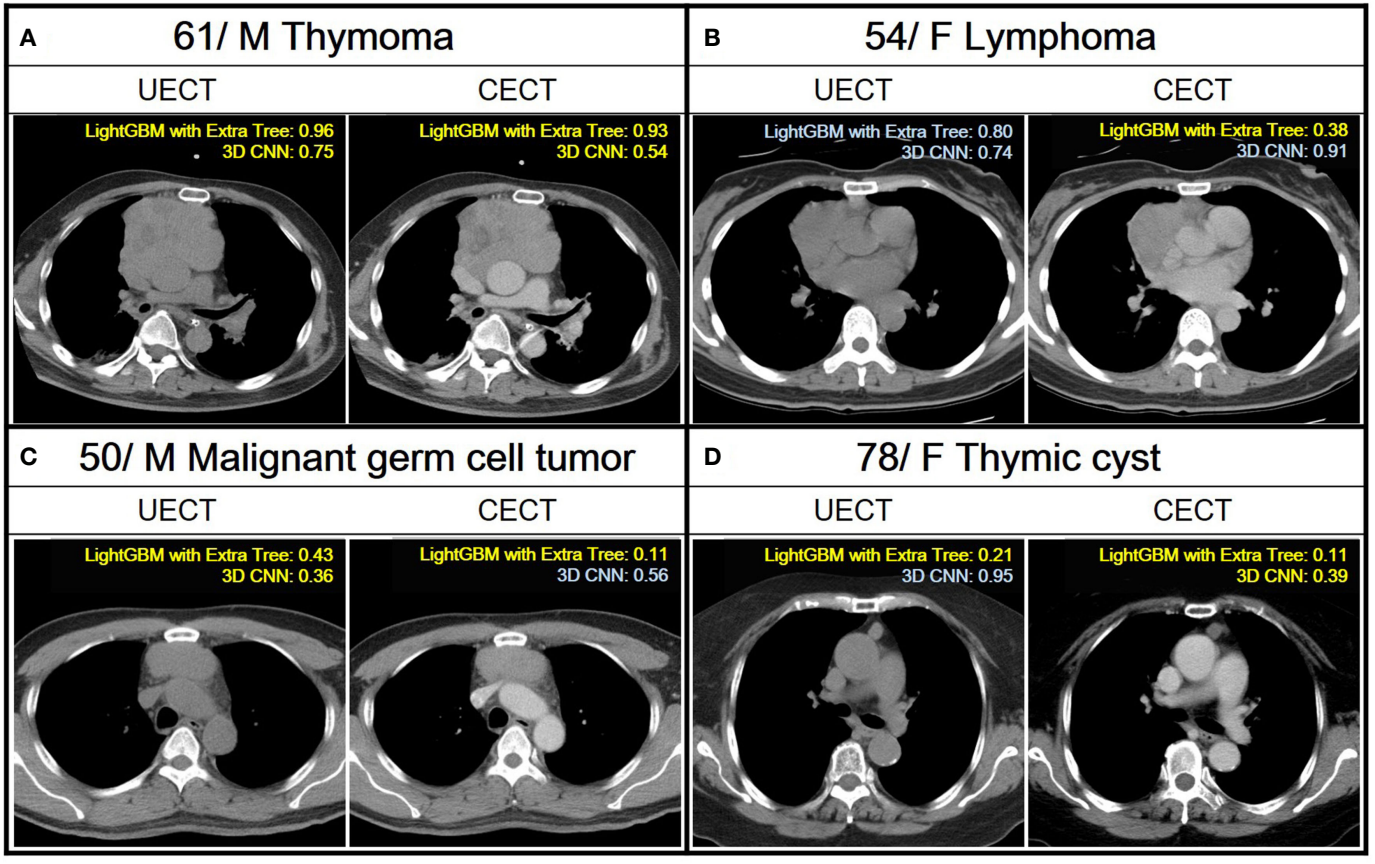
Demonstration of the application of ML and 3D CNN classification models to analyze four distinct cases with varying pathologies. A case is classified as TET if the confidence score obtained from a ML model and 3D CNN using their UECT and CECT, respectively, is greater than 0.50, and classified as non-TET if it is less than or equal to 0.50. Yellow text indicates correct predictions, while blue text indicates incorrect predictions. **(A)** A 61 years old male with thymoma. Both LightGBM with Extra Tree and 3D CNN had correct prediction from UECT and CECT. **(B)** A 54 years old female with lymphoma. The LightGBM with ExtraTree had correct prediction from CECT. **(C)** A 50 years old male with malignant germ cell tumor. The LightGBM with Extra Tree had correct prediction from both UECT and CECT, while 3D CNN had correct prediction from UECT. **(D)** A 78 years old female with thymic cyst. The LightGBM with Extra Tree had correct prediction from both UECT and CECT, while 3D CNN had correct prediction from CECT.

In comparison, the performance of radiomic analysis with machine learning model using LightGBM with Extra Tree had better performance than the 3D CNN model in both UECT and CECT dataset. Four cases were illustrated to differentiate thymic epithelial tumors from other prevascular mediastinal tumors with our ML and 3D CNN classification models in the **Figure 4**.

## Discussion

Our result showed radiomics with ensemble machine learning achieved better performance than 3D CNN in differentiating TETs from other PMTs. Deep learning (DL) model presented more stable shape than radiomics with ML model on ROC curve. Radiomics with ML and DL are active research in the field of oncology (17). Some studies showed that the DL model had better performance than the ML-based radiomics (18–20), some showed ML-based radiomics out-performed DL model (21), and some demonstrated DL-based radiomics model had the best performance (22, 23). Prior studies had performed radiomics based ML or DL to classify thymoma form other PMT (24–26). However, our study was the first to compare the performance of radiomics-based ML with DL using the same dataset to differentiated thymoma form other PMT. While it is well-known that with large datasets, the performance of DL model was superior to hand-crafted feature extraction, a large dataset is not always available in medicine and may be limited by factors such as disease incidence, prevalence, and obstacles in data procurement. For small dataset, studies have suggested feature engineering may be a more suitable machine learning strategy with notable advantages of radiomics for medical imaging analysis. At present, studies that directly compare radiomics and deep learning clinical model performance are relatively unexplored. In this study, we address these questions and further aim to enhance interpretability of such machine learning models (27).

There are a large number of vectors and associated computational cost in DL. We used 3D CNN in our study, which was relatively simple; however, the trainable parameters were up to 1,351,873. Because of the long training time, it would be of more difficulty for researchers to modify the algorithm repeatedly. In comparison, radiomics with ML had lower computational cost but was associated with more complicated process. After radiomic feature extraction, most feature vectors are redundant. Therefore, feature selection is demanded to build a model *via* ML. The method of ML has substantial impact on its performance. In our study, not all radiomics with ML method out-performed 3D CNN. In consistent with prior study, ensemble learning had the best result. Ensemble learning can also be applied in DL, with the cost of longer training time.

Our results revealed that dataset of CECT worked better than UECT in classifying thymoma from other PMTs using both radiomics models and 3D CNN model, which was consistent with our clinical experience. The imaging characters at chest CT scan of low-risk thymoma and thymic cyst showed round or oval shape, smooth contour, while high-risk thymoma showed irregular shape and contour. Nonseminomatous germ cell tumor demonstrated marked hemorrhagic necrosis, while teratoma revealed fat component (2). From the result of LASSO selection in UECT and CECT, sphericity in shape feature played an important role in two dataset model, consistent with the finding in our conventional CT scan. High resolution medical imaging contains many features that is difficult to discover by visual inspection. The ability of multi-scale and multiresolution in wavelet transform has been verified in many imaging studies, and often applied to image compression, edge detection, feature extraction, and texture analysis. Our study demonstrated that wavelet-based features were selected by two datasets, suggesting the importance of high order features in imaging identification. However, other shape features and original first order features are more important in classification in the UECT dataset than in the CECT dataset. Compared with CECT, UECT lacks contrast agent to demonstrate richer texture features of soft tissue, and the septa within the tumor or the range of necrosis are less clearly seen. This could be responsible for the reason that UECT had a different tendency of feature selection from CECT dataset.

In differentiating TET from cysts, radiologists primarily focus on the Hounsfield units (HU) changes between non-enhanced and contrast-enhanced scans. Previous studies have found that cysts have a mean attenuation value of around 23 HU and a maximal attenuation value of 58 HU (28). However, some thymic cysts may have increased CT attenuation if hemorrhage or infection occurs, and relying solely on non-enhanced scans may lead to misdiagnosis. According to our research approach, we have developed separate models for predicting TET from non-enhanced and contrast-enhanced CT scans. Interestingly, although contrast-enhanced CT has better predictive performance, using non-enhanced CT alone, whether based on radiomic-based machine learning or 3D convolutional neural network, achieves an AUC of > 0.9, which is close to the performance of the contrast-enhanced CT group. The difference of macro F1-score in all ML methods and 3D CNN in both UECT and CECT datasets was less than five percent. Tumor segmentation in UECT is sometimes difficult due to its proximity to adjacent vessel, heart, pericardial effusion, or consolidated lung. However, radiomics-based ML achieved an accuracy of 90%, indicating that our model had good performance using UECT. As the LDCT for lung cancer screening become more prevalent, there is increasing number of incidentally found asymptomatic PMTs. Once the UECT dataset provides high accuracy in differentiating PMTs, patients do not need to undergo CECT, and radiation dose and contrast agent exposure with the likelihood of kidney injury could therefore be minimized. Besides, previous literature has reported that approximately 22% to 68% of non-therapeutic thymectomies were unnecessary (28). In our dataset, 14% (53/376) of the patients had cysts or lymphoma and could have been otherwise managed instead of being operated on. Our model achieved an accuracy of 0.91. Therefore, we believe that increasing the accuracy of preoperative imaging diagnosis will help to reduce unnecessary invasive procedures.

Our study had several limitations. First, tumors were manually segmented; this could be user-dependent, time-consuming and labor-intensive. Prior studies have demonstrated DL-based tumor segmentation algorithm with robust performance. Automated tumor segmentation could probably be integrated into an automated processing pipeline to minimize subjectivity and facilitate large-scale studies. Second, after radiomics feature extraction, we used only LASSO regression with variable lambda value for feature selection. Adopting different feature selection methods could result in different outcome. Third, we used a relatively simple 3D CNN model for classification. Although complicated model is computationally expensive, future advancement in hardware (e.g., GPU or cloud computing) and algorithmic development is expectable. Fourth, because of the retrospective nature of this analysis, a selection bias was unavoidable. Lastly, CT images in our study were obtained using heterogeneous CT scanners, with various acquisition parameters, which can affect radiomic features and analysis. Nonetheless, the diagnostic performance of the radiomics model remained high in the validation cohort, which verified the good generalizability of the model.

As DL gradually becomes the mainstream of imaging study, some studies showed DL was superior to radiomics with ML in visual classification. However, it is susceptible to overfitting and takes a large number of data for model training and parameter tuning. Owing to the limited size of dataset, our proposed radiomics-based ML and 3D CNN scheme may be overfitting during training process. In rare disease with limited case number, radiomics-based ML may have better efficacy with lower computational cost. The best method of computing depends on the subject of study and size of dataset. Further studies are mandatory to evaluate the efficacy of ML and DL in the same dataset.

## Conclusion

To conclude, an ensemble ML method with radiomic feature can be useful for differentiating TETs from other type of PMTs, performed slightly better than a 3D CNN, and demonstrated good generalizability across institutions.

## Data availability statement

The original contributions presented in the study are included in the article/**Supplementary Material**. Further inquiries can be directed to the corresponding authors.

## Ethics statement

The studies involving human participants were reviewed and approved by National Cheng Kung University Hospital (A-ER-111-211), E-Da Hospital (EMRP-110-145), and Kaohsiung Veteran General Hospital (VGHKS19-CT6-08). Written informed consent for participation was not required for this study in accordance with the national legislation and the institutional requirements.

## Author contributions

(I) Conception and design: C-CC, Y-TY. (II) Administrative support: None. (III) Provision of study materials or patients: C-CC, E-KT, Y-FW, C-YL, F-ZW, M-TW, Y-SL, Y-TY and Y-LT. (IV) Collection and assembly of data: C-CC, E-KT, Y-FW, F-ZW and C-YL. (V) Data analysis and interpretation: C-CC, C-YL and M-CM. All authors contributed to the article and approved the submitted version.
