# Corrigendum: Clinical radiomics-based machine learning versus three-dimension convolutional neural network analysis for differentiation of thymic epithelial tumors from other prevascular mediastinal tumors on chest computed tomography scan

**DOI:** 10.3389/fonc.2023.1220962

**Published:** 2023-05-31

**Authors:** Chao-Chun Chang, En-Kuei Tang, Yu-Feng Wei, Chia-Ying Lin, Fu-Zong Wu, Ming-Ting Wu, Yi-Sheng Liu, Yi-Ting Yen, Mi-Chia Ma, Yau-Lin Tseng

**Affiliations:** ^1^ Division of Thoracic Surgery, Department of Surgery, National Cheng Kung University Hospital, College of Medicine, National Cheng Kung University, Tainan, Taiwan; ^2^ Division of Thoracic Surgery, Department of Surgery, Kaohsiung Veterans General Hospital, Kaohsiung, Taiwan; ^3^ School of Medicine for International Students, College of Medicine, I-Shou University, Kaohsiung, Taiwan; ^4^ Division of Chest Medicine, Department of Internal Medicine, E-Da Cancer Hospital, Kaohsiung, Taiwan; ^5^ Department of Medical Imaging, National Cheng Kung University Hospital, College of Medicine, National Cheng Kung University, Tainan, Taiwan; ^6^ Department of Radiology, Kaohsiung Veterans General Hospital, Kaohsiung, Taiwan; ^7^ Faculty of Clinical Medicine, National Yang Ming Chiao Tung University, Taipei, Taiwan; ^8^ Institute of Education, National Sun Yat-sen University, Kaohsiung, Taiwan; ^9^ School of Medicine, National Yang Ming Chiao Tung University, Taipei, Taiwan; ^10^ Institute of Clinical Medicine, National Yang Ming Chiao Tung University, Taipei, Taiwan; ^11^ Division of Trauma and Acute Care Surgery, Department of Surgery, National Cheng Kung University Hospital, College of Medicine, National Cheng Kung University, Tainan, Taiwan; ^12^ Department of Statistics and Institute of Data Science, National Cheng Kung University, Tainan, Taiwan

**Keywords:** radiomics, convolutional neural networks, deep learning, machine learning, prevascular mediastinal tumor

In the published article, there was a mistake in the Funding statement. The funding statement for the National Cheng Kung University Hospital of Taiwan was displayed as “NCKUH-11103026”. The correct statement is “National Cheng Kung University Hospital of Taiwan [NCKUH-11103026 and NCKUH-11201007].” The correct Funding statement appears below.

