# Supplementary material for: Clinical radiomics-based machine learning versus three-dimension convolutional neural network analysis for differentiation of thymic epithelial tumors from other prevascular mediastinal tumors on chest computed tomography scan
Source: Front Oncol. 2023 Apr 18;13:1105100. doi: 10.3389/fonc.2023.1105100 (PMC10151670; doi:10.3389/fonc.2023.1105100)
Supplement: Supplementary file 1 [file Table_1.docx]

**Supplementary table 1. Radiomics features extracted from UECT**

|  | **Others (n = 79)** | **TET (n = 297)** | ***p* value** |
| --- | --- | --- | --- |
|  | **Mean (SD)** | **Mean (SD)** |  |
| **original_shape_Elongation** | 0.7129 (0.1479) | 0.7317 (0.1319) | 0.2376 |
| **original_shape_Flatness** | 0.5079 (0.1356) | 0.5041 (0.1426) | 0.7457 |
| **original_shape_LeastAxisLength** | 33.3433 (21.6906) | 28.8018 (13.7901) | 0.2566 |
| **original_shape_MajorAxisLength** | 64.4230 (35.3562) | 58.2589 (25.9760) | 0.3133 |
| **original_shape_Maximum2DDiameterColumn** | 71.6033 (40.1969) | 63.6329 (28.3567) | 0.3047 |
| **original_shape_Maximum2DDiameterRow** | 69.8959 (38.3155) | 63.6546 (27.7987) | 0.4442 |
| **original_shape_Maximum2DDiameterSlice** | 59.2945 (36.7642) | 54.5320 (26.1231) | 0.5397 |
| **original_shape_Maximum3DDiameter** | 78.0235 (43.0699) | 70.3879 (30.9204) | 0.3164 |
| **original_shape_MeshVolume** | $1.32\times{10}^{5}$ ($2.30\times{10}^{5}$) | $6.83\times{10}^{4}$ ($1.04\times{10}^{5}$) | 0.0103 |
| **original_shape_MinorAxisLength** | 46.6454 (27.9686) | 42.0838 (18.5898) | 0.3529 |
| **original_shape_Sphericity** | 0.6369 (0.0788) | 0.6470 (0.0738) | 0.9429 |
| **original_shape_SurfaceArea** | $1.63\times{10}^{4}$ ($1.86\times{10}^{4}$) | $1.11\times{10}^{4}$ ($1.04\times{10}^{4}$) | 0.0305 |
| **original_shape_SurfaceVolumeRatio** | 0.2904 (0.1871) | 0.2619 (0.1193) | 0.0371 |
| **original_shape_VoxelVolume** | $1.32\times{10}^{5}$ ($2.30\times{10}^{5}$) | $6.83\times{10}^{4}$ ($1.04\times{10}^{5}$) | 0.0103 |
| **original_firstorder_10Percentile** | -17.3835 (30.3262) | -4.8641 (42.0439) | 0.0009 |
| **original_firstorder_90Percentile** | 39.7848 (19.5806) | 65.9846 (33.2593) | < 0.0001 |
| **original_firstorder_Energy** | $1.58\times{10}^{8}$ ($2.45\times{10}^{8}$) | $2.23\times{10}^{8}$ ($3.96\times{10}^{8}$) | 0.1954 |
| **original_firstorder_Entropy** | 1.9080 (0.4919) | 2.1093 (0.6454) | 0.0652 |
| **original_firstorder_InterquartileRange** | 26.6741 (13.8869) | 30.0243 (23.6038) | 0.6473 |
| **original_firstorder_Kurtosis** | 29.7147 (25.3058) | 35.6412 (51.0080) | 0.1864 |
| **original_firstorder_Maximum** | 165.1646 (87.0850) | 255.4832 (259.1946) | 0.0029 |
| **original_firstorder_MeanAbsoluteDeviation** | 19.8016 (8.6766) | 25.7695 (19.1084) | 0.0375 |
| **original_firstorder_Mean** | 12.7251 (22.6389) | 32.5889 (18.7630) | < 0.0001 |
| **original_firstorder_Median** | 17.3608 (23.9315) | 39.0436 (14.8034) | < 0.0001 |
| **original_firstorder_Minimum** | -368.8861 (163.9073) | -425.0336 (184.3526) | 0.0065 |
| **original_firstorder_Range** | 534.0506 (208.1543) | 680.5168 (335.5774) | 0.0001 |
| **original_firstorder_RobustMeanAbsoluteDeviation** | 11.6741 (5.7539) | 13.4228 (10.7138) | 0.9444 |
| **original_firstorder_RootMeanSquared** | 39.2238 (15.9431) | 57.7190 (27.7506) | < 0.0001 |
| **original_firstorder_Skewness** | -2.9554 (1.7893) | -2.8514 (2.9355) | 0.858 |
| **original_firstorder_TotalEnergy** | $1.58\times{10}^{8}$ ($2.45\times{10}^{8}$) | $2.23\times{10}^{8}$ ($3.96\times{10}^{8}$) | 0.1953 |
| **original_firstorder_Uniformity** | 0.3661 (0.1159) | 0.3418 (0.1140) | 0.3341 |
| **original_firstorder_Variance** | 1121.5118 (981.9918) | 2686.0900 (4677.0538) | 0.0026 |
| **original_glcm_Autocorrelation** | 312.2624 (220.3367) | 431.6045 (353.1844) | 0.0011 |
| **original_glcm_ClusterProminence** | 960.6057 (1988.2852) | $1.33\times{10}^{4}$ ($9.24\times{10}^{4}$) | 0.0401 |
| **original_glcm_ClusterShade** | -39.5703 (69.9384) | -26.4666 (1071.8660) | 0.4976 |
| **original_glcm_ClusterTendency** | 5.2153 (4.5899) | 12.7607 (24.8496) | 0.0043 |
| **original_glcm_Contrast** | 0.7460 (0.4186) | 1.8964 (4.7191) | 0.0134 |
| **original_glcm_Correlation** | 0.6756 (0.1333) | 0.7092 (0.1440) | 0.0003 |
| **original_glcm_DifferenceAverage** | 0.4819 (0.1459) | 0.6381 (0.6310) | 0.1655 |
| **original_glcm_DifferenceEntropy** | 1.1902 (0.2122) | 1.3316 (0.4673) | 0.0648 |
| **original_glcm_DifferenceVariance** | 0.4834 (0.2893) | 1.0566 (2.0015) | 0.0068 |
| **original_glcm_Id** | 0.7866 (0.0536) | 0.7659 (0.0989) | 0.6953 |
| **original_glcm_Idm** | 0.7801 (0.0580) | 0.7550 (0.1150) | 0.6182 |
| **original_glcm_Idmn** | 0.9974 (0.0036) | 0.9980 (0.0029) | 0.0296 |
| **original_glcm_Idn** | 0.9759 (0.0156) | 0.9782 (0.0133) | 0.0051 |
| **original_glcm_Imc1** | -0.2479 (0.0773) | -0.2588 (0.0815) | 0.0188 |
| **original_glcm_Imc2** | 0.7252 (0.1407) | 0.7544 (0.1245) | 0.0052 |
| **original_glcm_InverseVariance** | 0.3694 (0.0719) | 0.3587 (0.0644) | 0.4094 |
| **original_glcm_JointAverage** | 16.3852 (6.5738) | 19.4088 (7.2361) | 0.0004 |
| **original_glcm_JointEnergy** | 0.2127 (0.1023) | 0.1955 (0.1002) | 0.4627 |
| **original_glcm_JointEntropy** | 3.1612 (0.7499) | 3.4921 (1.1505) | 0.1191 |
| **original_glcm_MCC** | 0.7837 (0.0756) | 0.7951 (0.1000) | 0.0138 |
| **original_glcm_MaximumProbability** | 0.3700 (0.1362) | 0.3494 (0.1368) | 0.8318 |
| **original_glcm_SumAverage** | 32.7705 (13.1476) | 38.8176 (14.4723) | 0.0004 |
| **original_glcm_SumEntropy** | 2.5547 (0.5678) | 2.7629 (0.6914) | 0.0718 |
| **original_glcm_SumSquares** | 1.4903 (1.2276) | 3.6643 (6.9367) | 0.0042 |
| **original_gldm_DependenceEntropy** | 6.0736 (0.5446) | 6.2524 (0.5614) | 0.0213 |
| **original_gldm_DependenceNonUniformity** | 7016.1658 ($1.27\times{10}^{4}$) | 3611.3797 (6087.6819) | 0.0043 |
| **original_gldm_DependenceNonUniformityNormalized** | 0.0502 (0.0189) | 0.0521 (0.0303) | 0.2057 |
| **original_gldm_DependenceVariance** | 42.0169 (8.6131) | 42.7385 (11.3980) | 0.025 |
| **original_gldm_GrayLevelNonUniformity** | $5.97\times{10}^{4}$ ($1.07\times{10}^{5}$) | $2.61\times{10}^{4}$ ($4.62\times{10}^{4}$) | 0.003 |
| **original_gldm_GrayLevelVariance** | 1.8746 (1.5734) | 4.3779 (7.4818) | 0.0027 |
| **original_gldm_HighGrayLevelEmphasis** | 310.7031 (219.3213) | 429.0879 (351.9319) | 0.0011 |
| **original_gldm_LargeDependenceEmphasis** | 274.4683 (88.6627) | 266.1478 (88.6291) | 0.687 |
| **original_gldm_LargeDependenceHighGrayLevelEmphasis** | $8.76\times{10}^{4}$ ($6.13\times{10}^{4}$) | $1.08\times{10}^{5}$ ($9.78\times{10}^{4}$) | 0.0034 |
| **original_gldm_LargeDependenceLowGrayLevelEmphasis** | 1.8193 (2.0271) | 1.4366 (3.6973) | 0.0265 |
| **original_gldm_LowGrayLevelEmphasis** | 0.0100 (0.0177) | 0.0056 (0.0109) | 0.0066 |
| **original_gldm_SmallDependenceEmphasis** | 0.0233 (0.0116) | 0.0339 (0.0434) | 0.2547 |
| **original_gldm_SmallDependenceHighGrayLevelEmphasis** | 6.1243 (6.5153) | 17.2759 (41.4553) | 0.0039 |
| **original_gldm_SmallDependenceLowGrayLevelEmphasis** | 0.0006 (0.0011) | 0.0004 (0.0025) | 0.7072 |
| **original_glrlm_GrayLevelNonUniformity** | $1.82\times{10}^{4}$ ($3.86\times{10}^{4}$) | 8059.9826 ($1.33\times{10}^{4}$) | 0.0069 |
| **original_glrlm_GrayLevelNonUniformityNormalized** | 0.2722 (0.0726) | 0.2482 (0.0801) | 0.0277 |
| **original_glrlm_GrayLevelVariance** | 2.9702 (2.4407) | 6.6419 (9.7392) | 0.0003 |
| **original_glrlm_HighGrayLevelRunEmphasis** | 304.8567 (214.9493) | 420.4433 (347.6317) | 0.0013 |
| **original_glrlm_LongRunEmphasis** | 15.1058 (13.3525) | 16.1895 (62.7364) | 0.3078 |
| **original_glrlm_LongRunHighGrayLevelEmphasis** | 4611.6039 (4166.9961) | 4965.9992 (5086.0147) | 0.186 |
| **original_glrlm_LongRunLowGrayLevelEmphasis** | 0.0921 (0.1087) | 0.4644 (6.9765) | 0.0426 |
| **original_glrlm_LowGrayLevelRunEmphasis** | 0.0111 (0.0205) | 0.0062 (0.0118) | 0.0076 |
| **original_glrlm_RunEntropy** | 4.2828 (0.3382) | 4.4690 (0.3782) | < 0.0001 |
| **original_glrlm_RunLengthNonUniformity** | $1.94\times{10}^{4}$ ($4.16\times{10}^{4}$) | $1.16\times{10}^{4}$ ($2.23\times{10}^{4}$) | 0.036 |
| **original_glrlm_RunLengthNonUniformityNormalized** | 0.3586 (0.0727) | 0.3775 (0.1136) | 0.9641 |
| **original_glrlm_RunPercentage** | 0.4613 (0.1046) | 0.4763 (0.1219) | 0.6784 |
| **original_glrlm_RunVariance** | 8.0614 (8.8219) | 8.1456 (28.3585) | 0.3633 |
| **original_glrlm_ShortRunEmphasis** | 0.6075 (0.0666) | 0.6220 (0.0884) | 0.7862 |
| **original_glrlm_ShortRunHighGrayLevelEmphasis** | 181.5860 (134.8935) | 267.9960 (241.2080) | 0.0011 |
| **original_glrlm_ShortRunLowGrayLevelEmphasis** | 0.0079 (0.0157) | 0.0039 (0.0075) | 0.0087 |
| **original_glszm_GrayLevelNonUniformity** | 304.4505 (850.9063) | 109.7532 (250.9440) | 0.0028 |
| **original_glszm_GrayLevelNonUniformityNormalized** | 0.1395 (0.0791) | 0.1007 (0.0698) | < 0.0001 |
| **original_glszm_GrayLevelVariance** | 14.2645 (9.9757) | 25.4346 (31.1579) | 0.0001 |
| **original_glszm_HighGrayLevelZoneEmphasis** | 238.5523 (165.6076) | 331.7875 (326.1464) | 0.0047 |
| **original_glszm_LargeAreaEmphasis** | $6.35\times{10}^{6}$ ($1.18\times{10}^{7}$) | $2.56\times{10}^{6}$ ($6.02\times{10}^{6}$) | 0.0029 |
| **original_glszm_LargeAreaHighGrayLevelEmphasis** | $2.15\times{10}^{9}$ ($4.96\times{10}^{9}$) | $9.87\times{10}^{8}$ ($2.41\times{10}^{9}$) | 0.036 |
| **original_glszm_LargeAreaLowGrayLevelEmphasis** | $2.85\times{10}^{4}$ ($6.85\times{10}^{4}$) | $1.91\times{10}^{4}$ ($1.61\times{10}^{5}$) | 0.0103 |
| **original_glszm_LowGrayLevelZoneEmphasis** | 0.0309 (0.0643) | 0.0154 (0.0220) | 0.004 |
| **original_glszm_SizeZoneNonUniformity** | 216.0721 (296.4607) | 281.0511 (743.1815) | 0.7326 |
| **original_glszm_SizeZoneNonUniformityNormalized** | 0.1926 (0.0743) | 0.2251 (0.0698) | 0.0218 |
| **original_glszm_SmallAreaEmphasis** | 0.4163 (0.1113) | 0.4694 (0.0896) | 0.0025 |
| **original_glszm_SmallAreaHighGrayLevelEmphasis** | 91.0049 (71.6071) | 150.1962 (174.9603) | 0.0019 |
| **original_glszm_SmallAreaLowGrayLevelEmphasis** | 0.0158 (0.0236) | 0.0097 (0.0127) | 0.006 |
| **original_glszm_ZoneEntropy** | 6.0782 (0.8416) | 6.4142 (0.7763) | 0.0001 |
| **original_glszm_ZonePercentage** | 0.0186 (0.0130) | 0.0304 (0.0491) | 0.3001 |
| **original_glszm_ZoneVariance** | $6.35\times{10}^{6}$ ($1.18\times{10}^{7}$) | $2.55\times{10}^{6}$ ($6.02\times{10}^{6}$) | 0.0029 |
| **original_ngtdm_Busyness** | 23.1379 (43.6545) | 9.4531 (14.7232) | 0.0003 |
| **original_ngtdm_Coarseness** | 0.0018 (0.0035) | 0.0012 (0.0065) | 0.5914 |
| **original_ngtdm_Complexity** | 119.7022 (94.9745) | 313.6486 (645.0876) | 0.0021 |
| **original_ngtdm_Contrast** | 0.0059 (0.0082) | 0.0067 (0.0125) | 0.1392 |
| **original_ngtdm_Strength** | 0.4928 (0.8749) | 0.7912 (1.2804) | 0.075 |
| **wavelet-LLH_firstorder_10Percentile** | -6.7487 (6.6172) | -8.2444 (8.9132) | 0.9569 |
| **wavelet-LLH_firstorder_90Percentile** | 2.2534 (1.1814) | 3.6289 (17.3569) | 0.5429 |
| **wavelet-LLH_firstorder_Energy** | $7.80\times{10}^{6}$ ($2.66\times{10}^{7}$) | $1.33\times{10}^{7}$ ($4.28\times{10}^{7}$) | 0.3255 |
| **wavelet-LLH_firstorder_Entropy** | 1.0592 (0.1526) | 1.1218 (0.2337) | 0.0977 |
| **wavelet-LLH_firstorder_InterquartileRange** | 3.3152 (2.5750) | 4.2691 (9.9061) | 0.6621 |
| **wavelet-LLH_firstorder_Kurtosis** | 56.5199 (107.2738) | 137.5371 (365.5688) | 0.0153 |
| **wavelet-LLH_firstorder_Maximum** | 67.2763 (140.4464) | 127.4043 (196.0226) | 0.0033 |
| **wavelet-LLH_firstorder_MeanAbsoluteDeviation** | 3.9341 (4.3234) | 5.2091 (6.5420) | 0.3482 |
| **wavelet-LLH_firstorder_Mean** | -1.9114 (2.4159) | -1.1545 (9.9901) | 0.3818 |
| **wavelet-LLH_firstorder_Median** | -0.6964 (1.0552) | -0.1514 (9.4768) | 0.4478 |
| **wavelet-LLH_firstorder_Minimum** | -99.2950 (189.1648) | -125.7902 (187.2879) | 0.2449 |
| **wavelet-LLH_firstorder_Range** | 166.5714 (290.5883) | 253.1945 (324.7312) | 0.0176 |
| **wavelet-LLH_firstorder_RobustMeanAbsoluteDeviation** | 1.5366 (1.2242) | 2.0039 (3.7198) | 0.5812 |
| **wavelet-LLH_firstorder_RootMeanSquared** | 9.3000 (16.6338) | 12.9788 (19.6523) | 0.1953 |
| **wavelet-LLH_firstorder_Skewness** | -2.0203 (3.9823) | -1.0334 (8.7408) | 0.2007 |
| **wavelet-LLH_firstorder_TotalEnergy** | $7.80\times{10}^{6}$ ($2.66\times{10}^{7}$) | $1.33\times{10}^{7}$ ($4.28\times{10}^{7}$) | 0.3128 |
| **wavelet-LLH_firstorder_Uniformity** | 0.5143 (0.0485) | 0.5047 (0.0560) | 0.1835 |
| **wavelet-LLH_firstorder_Variance** | 350.2555 (1632.5669) | 452.5666 (1880.0219) | 0.6366 |
| **wavelet-LLH_glcm_Autocorrelation** | 80.4134 (315.3665) | 90.6908 (273.8580) | 0.6611 |
| **wavelet-LLH_glcm_ClusterProminence** | 2759.1887 ($1.57\times{10}^{4}$) | 2032.8631 ($1.38\times{10}^{4}$) | 0.9354 |
| **wavelet-LLH_glcm_ClusterShade** | -31.6386 (223.8863) | 6.3969 (105.5397) | 0.1174 |
| **wavelet-LLH_glcm_ClusterTendency** | 1.7628 (5.2227) | 2.1706 (5.8975) | 0.5704 |
| **wavelet-LLH_glcm_Contrast** | 0.7454 (3.0374) | 0.8465 (4.3565) | 0.7015 |
| **wavelet-LLH_glcm_Correlation** | 0.4751 (0.1157) | 0.5162 (0.1025) | 0.0263 |
| **wavelet-LLH_glcm_DifferenceAverage** | 0.2756 (0.0923) | 0.2842 (0.1644) | 0.8404 |
| **wavelet-LLH_glcm_DifferenceEntropy** | 0.8196 (0.1122) | 0.8316 (0.1670) | 0.8047 |
| **wavelet-LLH_glcm_DifferenceVariance** | 0.6560 (2.9494) | 0.7287 (4.1238) | 0.7139 |
| **wavelet-LLH_glcm_Id** | 0.8702 (0.0312) | 0.8715 (0.0417) | 0.5305 |
| **wavelet-LLH_glcm_Idm** | 0.8699 (0.0313) | 0.8705 (0.0449) | 0.6 |
| **wavelet-LLH_glcm_Idmn** | 0.9842 (0.0106) | 0.9894 (0.0092) | 0.0002 |
| **wavelet-LLH_glcm_Idn** | 0.9520 (0.0175) | 0.9620 (0.0196) | < 0.0001 |
| **wavelet-LLH_glcm_Imc1** | -0.2081 (0.0964) | -0.2392 (0.0824) | 0.0172 |
| **wavelet-LLH_glcm_Imc2** | 0.5573 (0.1122) | 0.6104 (0.0928) | 0.0006 |
| **wavelet-LLH_glcm_InverseVariance** | 0.2557 (0.0646) | 0.2467 (0.0620) | 0.2395 |
| **wavelet-LLH_glcm_JointAverage** | 4.8435 (7.5780) | 5.9281 (7.4433) | 0.2183 |
| **wavelet-LLH_glcm_JointEnergy** | 0.3382 (0.0749) | 0.3338 (0.0750) | 0.5338 |
| **wavelet-LLH_glcm_JointEntropy** | 1.8595 (0.2441) | 1.9342 (0.3873) | 0.235 |
| **wavelet-LLH_glcm_MCC** | 0.5752 (0.1114) | 0.6377 (0.1013) | < 0.0001 |
| **wavelet-LLH_glcm_MaximumProbability** | 0.4573 (0.1122) | 0.4657 (0.0984) | 0.6366 |
| **wavelet-LLH_glcm_SumAverage** | 9.6870 (15.1560) | 11.8561 (14.8867) | 0.2183 |
| **wavelet-LLH_glcm_SumEntropy** | 1.5916 (0.1858) | 1.6622 (0.2686) | 0.0871 |
| **wavelet-LLH_glcm_SumSquares** | 0.6271 (1.9644) | 0.7543 (2.4464) | 0.6107 |
| **wavelet-LLH_gldm_DependenceEntropy** | 5.2650 (0.2704) | 5.2655 (0.3133) | 0.801 |
| **wavelet-LLH_gldm_DependenceNonUniformity** | 7764.0732 ($1.32\times{10}^{4}$) | 4522.9184 (6451.1263) | 0.0338 |
| **wavelet-LLH_gldm_DependenceNonUniformityNormalized** | 0.0655 (0.0307) | 0.0716 (0.0337) | 0.1345 |
| **wavelet-LLH_gldm_DependenceVariance** | 33.7844 (5.8111) | 34.6014 (5.8530) | 0.2762 |
| **wavelet-LLH_gldm_GrayLevelNonUniformity** | $6.57\times{10}^{4}$ ($1.14\times{10}^{5}$) | $3.39\times{10}^{4}$ ($5.16\times{10}^{4}$) | 0.011 |
| **wavelet-LLH_gldm_GrayLevelVariance** | 0.7692 (2.6244) | 0.9261 (3.0087) | 0.6369 |
| **wavelet-LLH_gldm_HighGrayLevelEmphasis** | 80.6669 (315.6545) | 91.2924 (274.9711) | 0.6542 |
| **wavelet-LLH_gldm_LargeDependenceEmphasis** | 373.4671 (48.0326) | 388.4362 (60.8148) | 0.0107 |
| **wavelet-LLH_gldm_LargeDependenceHighGrayLevelEmphasis** | $3.68\times{10}^{4}$ ($1.52\times{10}^{5}$) | $3.72\times{10}^{4}$ ($1.17\times{10}^{5}$) | 0.8196 |
| **wavelet-LLH_gldm_LargeDependenceLowGrayLevelEmphasis** | 78.5777 (97.8976) | 52.0754 (69.8135) | 0.0164 |
| **wavelet-LLH_gldm_LowGrayLevelEmphasis** | 0.2017 (0.2263) | 0.1294 (0.1540) | 0.0043 |
| **wavelet-LLH_gldm_SmallDependenceEmphasis** | 0.0069 (0.0033) | 0.0083 (0.0156) | 0.5493 |
| **wavelet-LLH_gldm_SmallDependenceHighGrayLevelEmphasis** | 1.0401 (4.7963) | 1.2100 (4.4756) | 0.662 |
| **wavelet-LLH_gldm_SmallDependenceLowGrayLevelEmphasis** | 0.0013 (0.0013) | 0.0009 (0.0012) | 0.0224 |
| **wavelet-LLH_glrlm_GrayLevelNonUniformity** | $2.19\times{10}^{4}$ ($4.03\times{10}^{4}$) | $1.02\times{10}^{4}$ ($1.69\times{10}^{4}$) | 0.0055 |
| **wavelet-LLH_glrlm_GrayLevelNonUniformityNormalized** | 0.4766 (0.0440) | 0.4601 (0.0503) | 0.0784 |
| **wavelet-LLH_glrlm_GrayLevelVariance** | 2.1881 (9.8342) | 2.2248 (8.3914) | 0.9002 |
| **wavelet-LLH_glrlm_HighGrayLevelRunEmphasis** | 78.9318 (304.1344) | 92.7493 (275.1204) | 0.5954 |
| **wavelet-LLH_glrlm_LongRunEmphasis** | 18.9650 (8.5040) | 21.9190 (9.5943) | 0.008 |
| **wavelet-LLH_glrlm_LongRunHighGrayLevelEmphasis** | 2830.1938 ($1.31\times{10}^{4}$) | 2344.6218 (7671.7470) | 0.8439 |
| **wavelet-LLH_glrlm_LongRunLowGrayLevelEmphasis** | 3.9754 (5.9382) | 2.9982 (4.8547) | 0.1679 |
| **wavelet-LLH_glrlm_LowGrayLevelRunEmphasis** | 0.1926 (0.1973) | 0.1267 (0.1379) | 0.0031 |
| **wavelet-LLH_glrlm_RunEntropy** | 3.8660 (0.3646) | 4.0479 (0.3002) | < 0.0001 |
| **wavelet-LLH_glrlm_RunLengthNonUniformity** | 9863.7082 ($1.85\times{10}^{4}$) | 4540.5156 (7673.6887) | 0.0044 |
| **wavelet-LLH_glrlm_RunLengthNonUniformityNormalized** | 0.2065 (0.0321) | 0.2010 (0.0548) | 0.1219 |
| **wavelet-LLH_glrlm_RunPercentage** | 0.3312 (0.0474) | 0.3179 (0.0662) | 0.0167 |
| **wavelet-LLH_glrlm_RunVariance** | 7.8560 (4.4572) | 9.4276 (5.0587) | 0.0054 |
| **wavelet-LLH_glrlm_ShortRunEmphasis** | 0.4141 (0.0443) | 0.4092 (0.0603) | 0.2914 |
| **wavelet-LLH_glrlm_ShortRunHighGrayLevelEmphasis** | 33.3505 (127.9598) | 40.7072 (118.7767) | 0.5389 |
| **wavelet-LLH_glrlm_ShortRunLowGrayLevelEmphasis** | 0.0754 (0.0677) | 0.0501 (0.0480) | 0.0014 |
| **wavelet-LLH_glszm_GrayLevelNonUniformity** | 38.3715 (42.5736) | 30.4268 (38.4620) | 0.2252 |
| **wavelet-LLH_glszm_GrayLevelNonUniformityNormalized** | 0.3742 (0.1600) | 0.3122 (0.1444) | 0.0021 |
| **wavelet-LLH_glszm_GrayLevelVariance** | 11.5884 (50.6849) | 14.9426 (43.0955) | 0.3749 |
| **wavelet-LLH_glszm_HighGrayLevelZoneEmphasis** | 66.4559 (245.2725) | 101.3167 (259.5568) | 0.2353 |
| **wavelet-LLH_glszm_LargeAreaEmphasis** | $1.06\times{10}^{8}$ ($3.03\times{10}^{8}$) | $2.91\times{10}^{7}$ ($8.26\times{10}^{7}$) | 0.0041 |
| **wavelet-LLH_glszm_LargeAreaHighGrayLevelEmphasis** | $1.56\times{10}^{9}$ ($4.02\times{10}^{9}$) | $1.89\times{10}^{9}$ ($9.42\times{10}^{9}$) | 0.605 |
| **wavelet-LLH_glszm_LargeAreaLowGrayLevelEmphasis** | $1.25\times{10}^{7}$ ($3.29\times{10}^{7}$) | $2.43\times{10}^{6}$ ($4.57\times{10}^{6}$) | < 0.0001 |
| **wavelet-LLH_glszm_LowGrayLevelZoneEmphasis** | 0.2437 (0.1601) | 0.1828 (0.1321) | 0.0007 |
| **wavelet-LLH_glszm_SizeZoneNonUniformity** | 34.5325 (43.2212) | 33.0641 (56.0757) | 0.9792 |
| **wavelet-LLH_glszm_SizeZoneNonUniformityNormalized** | 0.2258 (0.0837) | 0.2127 (0.0901) | 0.7349 |
| **wavelet-LLH_glszm_SmallAreaEmphasis** | 0.4438 (0.1220) | 0.4371 (0.1259) | 0.7179 |
| **wavelet-LLH_glszm_SmallAreaHighGrayLevelEmphasis** | 39.8364 (157.0096) | 58.0793 (156.6398) | 0.2838 |
| **wavelet-LLH_glszm_SmallAreaLowGrayLevelEmphasis** | 0.0899 (0.0658) | 0.0639 (0.0506) | 0.0005 |
| **wavelet-LLH_glszm_ZoneEntropy** | 4.1134 (1.0047) | 4.5547 (0.8645) | 0.0003 |
| **wavelet-LLH_glszm_ZonePercentage** | 0.0032 (0.0040) | 0.0048 (0.0194) | 0.5416 |
| **wavelet-LLH_glszm_ZoneVariance** | $1.05\times{10}^{8}$ ($3.02\times{10}^{8}$) | $2.87\times{10}^{7}$ ($8.22\times{10}^{7}$) | 0.0042 |
| **wavelet-LLH_ngtdm_Busyness** | 1802.4774 (5621.3647) | 694.1527 (3757.1929) | 0.1956 |
| **wavelet-LLH_ngtdm_Coarseness** | 0.0019 (0.0040) | 0.0016 (0.0132) | 0.9881 |
| **wavelet-LLH_ngtdm_Complexity** | 45.6922 (237.6996) | 60.8667 (296.4162) | 0.4901 |
| **wavelet-LLH_ngtdm_Contrast** | 0.0130 (0.0115) | 0.0094 (0.0110) | 0.025 |
| **wavelet-LLH_ngtdm_Strength** | 0.4605 (2.5939) | 0.4568 (1.8812) | 0.7299 |
| **wavelet-LHL_firstorder_10Percentile** | -18.8984 (5.5453) | -22.9786 (24.8499) | 0.4131 |
| **wavelet-LHL_firstorder_90Percentile** | 15.7792 (5.6076) | 19.5075 (24.1156) | 0.4038 |
| **wavelet-LHL_firstorder_Energy** | $4.29\times{10}^{7}$ ($1.21\times{10}^{8}$) | $4.18\times{10}^{7}$ ($2.10\times{10}^{8}$) | 0.056 |
| **wavelet-LHL_firstorder_Entropy** | 1.4378 (0.2559) | 1.5356 (0.5971) | 0.9402 |
| **wavelet-LHL_firstorder_InterquartileRange** | 17.6897 (5.4643) | 21.3802 (25.2047) | 0.2979 |
| **wavelet-LHL_firstorder_Kurtosis** | 16.4865 (21.9660) | 22.7302 (31.6107) | 0.0713 |
| **wavelet-LHL_firstorder_Maximum** | 118.1891 (71.4902) | 145.3285 (131.5965) | 0.1271 |
| **wavelet-LHL_firstorder_MeanAbsoluteDeviation** | 11.1787 (3.2401) | 13.9127 (15.3487) | 0.7937 |
| **wavelet-LHL_firstorder_Mean** | -1.5532 (1.7801) | -1.7984 (1.7375) | 0.6926 |
| **wavelet-LHL_firstorder_Median** | -1.1498 (1.5680) | -1.1456 (1.3394) | 0.5555 |
| **wavelet-LHL_firstorder_Minimum** | -173.6293 (93.0842) | -230.0970 (183.2828) | 0.0219 |
| **wavelet-LHL_firstorder_Range** | 291.8184 (144.0084) | 375.4255 (298.2620) | 0.0337 |
| **wavelet-LHL_firstorder_RobustMeanAbsoluteDeviation** | 7.3798 (2.2685) | 8.9414 (10.4511) | 0.3083 |
| **wavelet-LHL_firstorder_RootMeanSquared** | 15.4375 (4.1656) | 19.9423 (20.2093) | 0.3109 |
| **wavelet-LHL_firstorder_Skewness** | -0.9257 (1.5681) | -1.3879 (1.5938) | 0.0379 |
| **wavelet-LHL_firstorder_TotalEnergy** | $4.29\times{10}^{7}$ ($1.21\times{10}^{8}$) | $4.18\times{10}^{7}$ ($2.10\times{10}^{8}$) | 0.056 |
| **wavelet-LHL_firstorder_Uniformity** | 0.4266 (0.0577) | 0.4158 (0.0892) | 0.8065 |
| **wavelet-LHL_firstorder_Variance** | 249.9085 (138.4526) | 798.4975 (2674.3011) | 0.2025 |
| **wavelet-LHL_glcm_Autocorrelation** | 76.4810 (67.7339) | 156.9441 (306.8220) | 0.0299 |
| **wavelet-LHL_glcm_ClusterProminence** | 16.6805 (30.3809) | 662.8762 (3721.4613) | 0.1175 |
| **wavelet-LHL_glcm_ClusterShade** | -0.8789 (1.8461) | -6.9615 (49.0809) | 0.0688 |
| **wavelet-LHL_glcm_ClusterTendency** | 1.0482 (0.4269) | 2.8087 (7.9901) | 0.1018 |
| **wavelet-LHL_glcm_Contrast** | 0.8347 (0.3839) | 2.4873 (8.7822) | 0.487 |
| **wavelet-LHL_glcm_Correlation** | 0.1114 (0.0860) | 0.1477 (0.1065) | 0.0032 |
| **wavelet-LHL_glcm_DifferenceAverage** | 0.6117 (0.1412) | 0.7589 (0.8334) | 0.6899 |
| **wavelet-LHL_glcm_DifferenceEntropy** | 1.2285 (0.1995) | 1.3017 (0.5068) | 0.6166 |
| **wavelet-LHL_glcm_DifferenceVariance** | 0.3989 (0.1603) | 1.0971 (3.3619) | 0.2033 |
| **wavelet-LHL_glcm_Id** | 0.7248 (0.0401) | 0.7151 (0.0899) | 0.4622 |
| **wavelet-LHL_glcm_Idm** | 0.7153 (0.0484) | 0.7038 (0.1071) | 0.4479 |
| **wavelet-LHL_glcm_Idmn** | 0.9918 (0.0076) | 0.9925 (0.0089) | 0.0457 |
| **wavelet-LHL_glcm_Idn** | 0.9498 (0.0204) | 0.9526 (0.0220) | 0.0223 |
| **wavelet-LHL_glcm_Imc1** | -0.0990 (0.0154) | -0.1011 (0.0196) | 0.0649 |
| **wavelet-LHL_glcm_Imc2** | 0.3900 (0.0611) | 0.4029 (0.0826) | 0.2316 |
| **wavelet-LHL_glcm_InverseVariance** | 0.4706 (0.0232) | 0.4571 (0.0527) | 0.8501 |
| **wavelet-LHL_glcm_JointAverage** | 7.9121 (3.7421) | 10.1601 (7.3360) | 0.0207 |
| **wavelet-LHL_glcm_JointEnergy** | 0.2144 (0.0501) | 0.2101 (0.0612) | 0.6717 |
| **wavelet-LHL_glcm_JointEntropy** | 2.6682 (0.4834) | 2.8449 (1.1455) | 0.7777 |
| **wavelet-LHL_glcm_MCC** | 0.4436 (0.1067) | 0.4662 (0.1080) | 0.0526 |
| **wavelet-LHL_glcm_MaximumProbability** | 0.2864 (0.0601) | 0.2714 (0.0683) | 0.4006 |
| **wavelet-LHL_glcm_SumAverage** | 15.8242 (7.4842) | 20.3202 (14.6719) | 0.0207 |
| **wavelet-LHL_glcm_SumEntropy** | 1.8755 (0.2557) | 1.9864 (0.5850) | 0.8483 |
| **wavelet-LHL_glcm_SumSquares** | 0.4707 (0.1965) | 1.3240 (4.1645) | 0.1813 |
| **wavelet-LHL_gldm_DependenceEntropy** | 5.3720 (0.2109) | 5.4386 (0.3690) | 0.8027 |
| **wavelet-LHL_gldm_DependenceNonUniformity** | 8769.0439 ($1.51\times{10}^{4}$) | 4735.5745 (7661.9211) | 0.009 |
| **wavelet-LHL_gldm_DependenceNonUniformityNormalized** | 0.0631 (0.0065) | 0.0685 (0.0243) | 0.2227 |
| **wavelet-LHL_gldm_DependenceVariance** | 20.9996 (3.8396) | 20.2244 (4.8941) | 0.5526 |
| **wavelet-LHL_gldm_GrayLevelNonUniformity** | $5.19\times{10}^{4}$ ($7.92\times{10}^{4}$) | $2.80\times{10}^{4}$ ($4.23\times{10}^{4}$) | 0.0129 |
| **wavelet-LHL_gldm_GrayLevelVariance** | 0.5042 (0.2075) | 1.3891 (4.2755) | 0.1615 |
| **wavelet-LHL_gldm_HighGrayLevelEmphasis** | 76.7974 (67.7195) | 157.9779 (308.5712) | 0.0301 |
| **wavelet-LHL_gldm_LargeDependenceEmphasis** | 170.6909 (28.2621) | 173.6350 (45.4291) | 0.1037 |
| **wavelet-LHL_gldm_LargeDependenceHighGrayLevelEmphasis** | $1.29\times{10}^{4}$ ($1.09\times{10}^{4}$) | $2.21\times{10}^{4}$ ($3.79\times{10}^{4}$) | 0.0243 |
| **wavelet-LHL_gldm_LargeDependenceLowGrayLevelEmphasis** | 6.5409 (8.6920) | 5.2197 (7.9524) | 0.0289 |
| **wavelet-LHL_gldm_LowGrayLevelEmphasis** | 0.0397 (0.0491) | 0.0302 (0.0498) | 0.0214 |
| **wavelet-LHL_gldm_SmallDependenceEmphasis** | 0.0192 (0.0069) | 0.0281 (0.0507) | 0.619 |
| **wavelet-LHL_gldm_SmallDependenceHighGrayLevelEmphasis** | 1.4475 (1.6776) | 10.2085 (42.7417) | 0.0671 |
| **wavelet-LHL_gldm_SmallDependenceLowGrayLevelEmphasis** | 0.0012 (0.0017) | 0.0009 (0.0030) | 0.5379 |
| **wavelet-LHL_glrlm_GrayLevelNonUniformity** | $2.76\times{10}^{4}$ ($4.39\times{10}^{4}$) | $1.44\times{10}^{4}$ ($2.16\times{10}^{4}$) | 0.0095 |
| **wavelet-LHL_glrlm_GrayLevelNonUniformityNormalized** | 0.3911 (0.0601) | 0.3834 (0.0882) | 0.7025 |
| **wavelet-LHL_glrlm_GrayLevelVariance** | 0.6430 (0.2756) | 1.6345 (4.4794) | 0.0874 |
| **wavelet-LHL_glrlm_HighGrayLevelRunEmphasis** | 76.7290 (67.4789) | 157.8179 (308.3616) | 0.0302 |
| **wavelet-LHL_glrlm_LongRunEmphasis** | 9.2936 (3.7206) | 9.7782 (4.0889) | 0.0341 |
| **wavelet-LHL_glrlm_LongRunHighGrayLevelEmphasis** | 740.8563 (661.7494) | 1223.6871 (1940.3658) | 0.0179 |
| **wavelet-LHL_glrlm_LongRunLowGrayLevelEmphasis** | 0.3029 (0.3217) | 0.3016 (0.6275) | 0.4162 |
| **wavelet-LHL_glrlm_LowGrayLevelRunEmphasis** | 0.0406 (0.0498) | 0.0308 (0.0506) | 0.0237 |
| **wavelet-LHL_glrlm_RunEntropy** | 3.3028 (0.1559) | 3.4174 (0.3030) | 0.0042 |
| **wavelet-LHL_glrlm_RunLengthNonUniformity** | $3.71\times{10}^{4}$ ($7.93\times{10}^{4}$) | $1.79\times{10}^{4}$ ($3.19\times{10}^{4}$) | 0.0092 |
| **wavelet-LHL_glrlm_RunLengthNonUniformityNormalized** | 0.4107 (0.0529) | 0.4132 (0.1043) | 0.0865 |
| **wavelet-LHL_glrlm_RunPercentage** | 0.5702 (0.0467) | 0.5690 (0.0849) | 0.0585 |
| **wavelet-LHL_glrlm_RunVariance** | 3.3671 (1.5840) | 3.5869 (1.7155) | 0.025 |
| **wavelet-LHL_glrlm_ShortRunEmphasis** | 0.6299 (0.0491) | 0.6279 (0.0813) | 0.0962 |
| **wavelet-LHL_glrlm_ShortRunHighGrayLevelEmphasis** | 48.4757 (44.7842) | 109.6372 (240.8152) | 0.0356 |
| **wavelet-LHL_glrlm_ShortRunLowGrayLevelEmphasis** | 0.0266 (0.0330) | 0.0194 (0.0326) | 0.0179 |
| **wavelet-LHL_glszm_GrayLevelNonUniformity** | 515.9181 (1036.3669) | 243.7344 (529.9259) | 0.0087 |
| **wavelet-LHL_glszm_GrayLevelNonUniformityNormalized** | 0.3096 (0.0850) | 0.2655 (0.0952) | 0.001 |
| **wavelet-LHL_glszm_GrayLevelVariance** | 3.7637 (1.8069) | 7.0611 (10.4900) | 0.0172 |
| **wavelet-LHL_glszm_HighGrayLevelZoneEmphasis** | 73.2946 (64.8063) | 150.3549 (302.5634) | 0.0366 |
| **wavelet-LHL_glszm_LargeAreaEmphasis** | $6.96\times{10}^{6}$ ($1.69\times{10}^{7}$) | $4.17\times{10}^{6}$ ($8.88\times{10}^{6}$) | 0.0414 |
| **wavelet-LHL_glszm_LargeAreaHighGrayLevelEmphasis** | $7.06\times{10}^{8}$ ($1.58\times{10}^{9}$) | $5.58\times{10}^{8}$ ($1.66\times{10}^{9}$) | 0.6349 |
| **wavelet-LHL_glszm_LargeAreaLowGrayLevelEmphasis** | $9.91\times{10}^{4}$ ($2.37\times{10}^{5}$) | $1.93\times{10}^{5}$ ($2.00\times{10}^{6}$) | 0.342 |
| **wavelet-LHL_glszm_LowGrayLevelZoneEmphasis** | 0.0744 (0.0982) | 0.0589 (0.0870) | 0.042 |
| **wavelet-LHL_glszm_SizeZoneNonUniformity** | 161.3869 (355.9520) | 220.7243 (1145.9777) | 0.0468 |
| **wavelet-LHL_glszm_SizeZoneNonUniformityNormalized** | 0.1316 (0.0511) | 0.1465 (0.0636) | 0.8629 |
| **wavelet-LHL_glszm_SmallAreaEmphasis** | 0.2872 (0.0975) | 0.3290 (0.1074) | 0.1263 |
| **wavelet-LHL_glszm_SmallAreaHighGrayLevelEmphasis** | 20.1791 (19.4614) | 57.7144 (143.8180) | 0.0481 |
| **wavelet-LHL_glszm_SmallAreaLowGrayLevelEmphasis** | 0.0245 (0.0329) | 0.0230 (0.0366) | 0.3995 |
| **wavelet-LHL_glszm_ZoneEntropy** | 5.2870 (0.8817) | 5.5365 (0.8454) | 0.0088 |
| **wavelet-LHL_glszm_ZonePercentage** | 0.0133 (0.0069) | 0.0241 (0.0598) | 0.6589 |
| **wavelet-LHL_glszm_ZoneVariance** | $6.93\times{10}^{6}$ ($1.68\times{10}^{7}$) | $4.13\times{10}^{6}$ ($8.79\times{10}^{6}$) | 0.0405 |
| **wavelet-LHL_ngtdm_Busyness** | 122.3554 (173.2399) | 126.9763 (944.8166) | 0.0106 |
| **wavelet-LHL_ngtdm_Coarseness** | 0.0009 (0.0018) | 0.0007 (0.0044) | 0.793 |
| **wavelet-LHL_ngtdm_Complexity** | 56.9088 (57.5492) | 239.6124 (878.8473) | 0.0562 |
| **wavelet-LHL_ngtdm_Contrast** | 0.0066 (0.0066) | 0.0092 (0.0218) | 0.0681 |
| **wavelet-LHL_ngtdm_Strength** | 0.0360 (0.0428) | 0.0934 (0.2659) | 0.1341 |
| **wavelet-LHH_firstorder_10Percentile** | -1.0505 (0.5399) | -1.4018 (2.9459) | 0.8846 |
| **wavelet-LHH_firstorder_90Percentile** | 1.0491 (0.5488) | 1.4208 (3.2486) | 0.8152 |
| **wavelet-LHH_firstorder_Energy** | $1.60\times{10}^{5}$ ($3.91\times{10}^{5}$) | $2.07\times{10}^{5}$ ($9.37\times{10}^{5}$) | 0.6669 |
| **wavelet-LHH_firstorder_Entropy** | 1.0012 (0.0102) | 1.0134 (0.0967) | 0.3169 |
| **wavelet-LHH_firstorder_InterquartileRange** | 1.0314 (0.5228) | 1.4043 (3.2706) | 0.7888 |
| **wavelet-LHH_firstorder_Kurtosis** | 33.2820 (69.8600) | 121.0477 (376.1165) | 0.0166 |
| **wavelet-LHH_firstorder_Maximum** | 13.7924 (12.3066) | 23.7432 (34.1854) | 0.003 |
| **wavelet-LHH_firstorder_MeanAbsoluteDeviation** | 0.7079 (0.3601) | 0.9555 (1.9581) | 0.7709 |
| **wavelet-LHH_firstorder_Mean** | 0.0034 (0.0505) | -0.0034 (0.0974) | 0.2243 |
| **wavelet-LHH_firstorder_Median** | 0.0052 (0.0366) | -0.0238 (0.4231) | 0.4458 |
| **wavelet-LHH_firstorder_Minimum** | -13.7087 (14.1406) | -22.1080 (35.0976) | 0.0299 |
| **wavelet-LHH_firstorder_Range** | 27.5012 (25.8812) | 45.8513 (68.4784) | 0.0086 |
| **wavelet-LHH_firstorder_RobustMeanAbsoluteDeviation** | 0.4340 (0.2200) | 0.5890 (1.3546) | 0.7971 |
| **wavelet-LHH_firstorder_RootMeanSquared** | 1.0995 (0.6964) | 1.5450 (2.6158) | 0.4307 |
| **wavelet-LHH_firstorder_Skewness** | 0.1183 (1.2300) | 0.4120 (4.2067) | 0.4353 |
| **wavelet-LHH_firstorder_TotalEnergy** | $1.60\times{10}^{5}$ ($3.91\times10^5$) | $2.08\times{10}^{5}$ ($9.37\times{10}^{5}$) | 0.6808 |
| **wavelet-LHH_firstorder_Uniformity** | 0.5003 (0.0018) | 0.4980 (0.0186) | 0.2171 |
| **wavelet-LHH_firstorder_Variance** | 1.6853 (3.4010) | 9.1971 (63.2654) | 0.4106 |
| **wavelet-LHH_glcm_Autocorrelation** | 3.0454 (2.6986) | 5.8048 (17.7300) | 0.158 |
| **wavelet-LHH_glcm_ClusterProminence** | 0.5316 (0.0570) | 0.7902 (2.3779) | 0.344 |
| **wavelet-LHH_glcm_ClusterShade** | $-6.71\times{10}^{-5}$ (0.0212) | 0.0081 (0.0617) | 0.2886 |
| **wavelet-LHH_glcm_ClusterTendency** | 0.5244 (0.0256) | 0.5436 (0.1561) | 0.3752 |
| **wavelet-LHH_glcm_Contrast** | 0.4762 (0.0265) | 0.4886 (0.1689) | 0.592 |
| **wavelet-LHH_glcm_Correlation** | 0.0482 (0.0512) | 0.0552 (0.0472) | 0.703 |
| **wavelet-LHH_glcm_DifferenceAverage** | 0.4757 (0.0261) | 0.4780 (0.0620) | 0.7695 |
| **wavelet-LHH_glcm_DifferenceEntropy** | 0.9685 (0.0145) | 0.9791 (0.0770) | 0.0524 |
| **wavelet-LHH_glcm_DifferenceVariance** | 0.2394 (0.0056) | 0.2469 (0.0668) | 0.1062 |
| **wavelet-LHH_glcm_Id** | 0.7622 (0.0130) | 0.7623 (0.0194) | 0.8611 |
| **wavelet-LHH_glcm_Idm** | 0.7622 (0.0130) | 0.7619 (0.0221) | 0.8253 |
| **wavelet-LHH_glcm_Idmn** | 0.9144 (0.0231) | 0.9249 (0.0312) | 0.0045 |
| **wavelet-LHH_glcm_Idn** | 0.8506 (0.0237) | 0.8624 (0.0334) | 0.0051 |
| **wavelet-LHH_glcm_Imc1** | -0.0329 (0.0115) | -0.0318 (0.0149) | 0.048 |
| **wavelet-LHH_glcm_Imc2** | 0.1914 (0.0477) | 0.1852 (0.0537) | 0.0436 |
| **wavelet-LHH_glcm_InverseVariance** | 0.4755 (0.0260) | 0.4725 (0.0237) | 0.9577 |
| **wavelet-LHH_glcm_JointAverage** | 1.6642 (0.5168) | 1.9631 (1.3942) | 0.0664 |
| **wavelet-LHH_glcm_JointEnergy** | 0.2614 (0.0042) | 0.2589 (0.0153) | 0.0148 |
| **wavelet-LHH_glcm_JointEntropy** | 1.9686 (0.0203) | 1.9917 (0.1820) | 0.1563 |
| **wavelet-LHH_glcm_MCC** | 0.1694 (0.0537) | 0.1729 (0.0702) | 0.9861 |
| **wavelet-LHH_glcm_MaximumProbability** | 0.2976 (0.0133) | 0.2921 (0.0164) | < 0.0001 |
| **wavelet-LHH_glcm_SumAverage** | 3.3285 (1.0335) | 3.9262 (2.7885) | 0.0664 |
| **wavelet-LHH_glcm_SumEntropy** | 1.4921 (0.0305) | 1.5088 (0.0931) | 0.095 |
| **wavelet-LHH_glcm_SumSquares** | 0.2501 (0.0027) | 0.2581 (0.0803) | 0.3994 |
| **wavelet-LHH_gldm_DependenceEntropy** | 4.8832 (0.1834) | 4.9113 (0.1813) | 0.9188 |
| **wavelet-LHH_gldm_DependenceNonUniformity** | $1.21\times{10}^{4}$ ($2.14\times{10}^{4}$) | 5991.6241 (9717.8560) | 0.0088 |
| **wavelet-LHH_gldm_DependenceNonUniformityNormalized** | 0.0801 (0.0124) | 0.0796 (0.0127) | 0.5809 |
| **wavelet-LHH_gldm_DependenceVariance** | 13.6629 (3.8471) | 13.9578 (3.3366) | 0.7749 |
| **wavelet-LHH_gldm_GrayLevelNonUniformity** | $6.62\times{10}^{4}$ ($1.15\times{10}^{5}$) | $3.42\times{10}^{4}$ ($5.20\times{10}^{4}$) | 0.0103 |
| **wavelet-LHH_gldm_GrayLevelVariance** | 0.2503 (0.0032) | 0.2592 (0.0866) | 0.3699 |
| **wavelet-LHH_gldm_HighGrayLevelEmphasis** | 3.2887 (2.7017) | 6.0563 (17.7570) | 0.1582 |
| **wavelet-LHH_gldm_LargeDependenceEmphasis** | 191.0461 (14.0252) | 195.9714 (21.2667) | 0.0677 |
| **wavelet-LHH_gldm_LargeDependenceHighGrayLevelEmphasis** | 625.4101 (523.7991) | 1103.9657 (3071.0764) | 0.1475 |
| **wavelet-LHH_gldm_LargeDependenceLowGrayLevelEmphasis** | 109.6200 (31.9925) | 104.1547 (43.8220) | 0.183 |
| **wavelet-LHH_gldm_LowGrayLevelEmphasis** | 0.5695 (0.1545) | 0.5175 (0.2075) | 0.0355 |
| **wavelet-LHH_gldm_SmallDependenceEmphasis** | 0.0086 (0.0020) | 0.0093 (0.0125) | 0.764 |
| **wavelet-LHH_gldm_SmallDependenceHighGrayLevelEmphasis** | 0.0279 (0.0230) | 0.0855 (0.4071) | 0.2597 |
| **wavelet-LHH_gldm_SmallDependenceLowGrayLevelEmphasis** | 0.0049 (0.0018) | 0.0044 (0.0032) | 0.2734 |
| **wavelet-LHH_glrlm_GrayLevelNonUniformity** | $3.43\times{10}^{4}$ ($5.95\times{10}^{4}$) | $1.75\times{10}^{4}$ ($2.67\times{10}^{4}$) | 0.0094 |
| **wavelet-LHH_glrlm_GrayLevelNonUniformityNormalized** | 0.4999 (0.0020) | 0.4971 (0.0213) | 0.3481 |
| **wavelet-LHH_glrlm_GrayLevelVariance** | 0.2508 (0.0055) | 0.2627 (0.1039) | 0.3655 |
| **wavelet-LHH_glrlm_HighGrayLevelRunEmphasis** | 3.2965 (2.7089) | 6.0762 (17.7678) | 0.1561 |
| **wavelet-LHH_glrlm_LongRunEmphasis** | 6.1736 (0.7508) | 6.4024 (0.9381) | 0.2765 |
| **wavelet-LHH_glrlm_LongRunHighGrayLevelEmphasis** | 20.1604 (17.0816) | 42.0301 (151.4330) | 0.1447 |
| **wavelet-LHH_glrlm_LongRunLowGrayLevelEmphasis** | 3.5467 (1.0951) | 3.3412 (1.4034) | 0.1368 |
| **wavelet-LHH_glrlm_LowGrayLevelRunEmphasis** | 0.5680 (0.1535) | 0.5143 (0.2057) | 0.0308 |
| **wavelet-LHH_glrlm_RunEntropy** | 2.8989 (0.0873) | 2.9389 (0.0821) | 0.0021 |
| **wavelet-LHH_glrlm_RunLengthNonUniformity** | $2.56\times{10}^{4}$ ($4.53\times{10}^{4}$) | $1.28\times{10}^{4}$ ($1.98\times{10}^{4}$) | 0.0077 |
| **wavelet-LHH_glrlm_RunLengthNonUniformityNormalized** | 0.3587 (0.0180) | 0.3541 (0.0305) | 0.068 |
| **wavelet-LHH_glrlm_RunPercentage** | 0.5266 (0.0209) | 0.5201 (0.0295) | 0.0162 |
| **wavelet-LHH_glrlm_RunVariance** | 1.9460 (0.2965) | 2.0719 (0.4191) | 0.0305 |
| **wavelet-LHH_glrlm_ShortRunEmphasis** | 0.5919 (0.0193) | 0.5874 (0.0285) | 0.168 |
| **wavelet-LHH_glrlm_ShortRunHighGrayLevelEmphasis** | 1.9556 (1.6200) | 3.6816 (11.0095) | 0.1618 |
| **wavelet-LHH_glrlm_ShortRunLowGrayLevelEmphasis** | 0.3358 (0.0912) | 0.2994 (0.1192) | 0.0173 |
| **wavelet-LHH_glszm_GrayLevelNonUniformity** | 10.1741 (9.0988) | 11.1822 (29.3877) | 0.3952 |
| **wavelet-LHH_glszm_GrayLevelNonUniformityNormalized** | 0.5159 (0.0894) | 0.4946 (0.1330) | 0.3078 |
| **wavelet-LHH_glszm_GrayLevelVariance** | 0.3517 (0.4841) | 0.6609 (1.5722) | 0.1424 |
| **wavelet-LHH_glszm_HighGrayLevelZoneEmphasis** | 3.3240 (3.1806) | 6.2900 (18.7913) | 0.1874 |
| **wavelet-LHH_glszm_LargeAreaEmphasis** | $6.82\times{10}^{8}$ ($1.78\times{10}^{9}$) | $1.95\times{10}^{8}$ ($6.04\times{10}^{8}$) | 0.0049 |
| **wavelet-LHH_glszm_LargeAreaHighGrayLevelEmphasis** | $1.87\times{10}^{9}$ ($4.49\times{10}^{9}$) | $8.72\times{10}^{8}$ ($2.93\times{10}^{9}$) | 0.0782 |
| **wavelet-LHH_glszm_LargeAreaLowGrayLevelEmphasis** | $4.14\times{10}^{8}$ ($1.12\times{10}^{9}$) | $1.01\times{10}^{8}$ ($3.69\times{10}^{8}$) | 0.0029 |
| **wavelet-LHH_glszm_LowGrayLevelZoneEmphasis** | 0.5934 (0.1601) | 0.5631 (0.2162) | 0.4177 |
| **wavelet-LHH_glszm_SizeZoneNonUniformity** | 7.9489 (7.5915) | 10.8243 (32.3854) | 0.8598 |
| **wavelet-LHH_glszm_SizeZoneNonUniformityNormalized** | 0.3764 (0.1108) | 0.3550 (0.1011) | 0.0659 |
| **wavelet-LHH_glszm_SmallAreaEmphasis** | 0.5403 (0.1884) | 0.5398 (0.1603) | 0.9239 |
| **wavelet-LHH_glszm_SmallAreaHighGrayLevelEmphasis** | 1.8030 (1.9830) | 3.8354 (14.3348) | 0.19 |
| **wavelet-LHH_glszm_SmallAreaLowGrayLevelEmphasis** | 0.3236 (0.1441) | 0.3022 (0.1593) | 0.5009 |
| **wavelet-LHH_glszm_ZoneEntropy** | 2.4299 (0.5775) | 2.6051 (0.6500) | 0.0382 |
| **wavelet-LHH_glszm_ZonePercentage** | 0.0009 (0.0011) | 0.0020 (0.0148) | 0.6198 |
| **wavelet-LHH_glszm_ZoneVariance** | $6.44\times{10}^{8}$ ($1.72\times{10}^{9}$) | $1.78\times{10}^{8}$ ($5.82\times{10}^{8}$) | 0.0052 |
| **wavelet-LHH_ngtdm_Busyness** | $2.70\times{10}^{4}$ ($5.27\times{10}^{4}$) | $1.06\times{10}^{4}$ ($1.60\times{10}^{4}$) | 0.0002 |
| **wavelet-LHH_ngtdm_Coarseness** | 0.0007 (0.0014) | 0.0005 (0.0037) | 0.8383 |
| **wavelet-LHH_ngtdm_Complexity** | 1.0266 (1.8134) | 3.3485 (13.7263) | 0.0932 |
| **wavelet-LHH_ngtdm_Contrast** | 0.1036 (0.0336) | 0.0894 (0.0438) | 0.0054 |
| **wavelet-LHH_ngtdm_Strength** | 0.0010 (0.0018) | 0.0036 (0.0241) | 0.4932 |
| **wavelet-HLL_firstorder_10Percentile** | -24.0281 (17.5694) | -31.1035 (36.4995) | 0.6852 |
| **wavelet-HLL_firstorder_90Percentile** | 14.6897 (4.1533) | 20.0738 (26.3867) | 0.6778 |
| **wavelet-HLL_firstorder_Energy** | $3.53\times{10}^{7}$ ($5.57\times{10}^{7}$) | $5.62\times{10}^{7}$ ($2.09\times{10}^{8}$) | 0.7325 |
| **wavelet-HLL_firstorder_Entropy** | 1.5498 (0.3428) | 1.7196 (0.6533) | 0.2276 |
| **wavelet-HLL_firstorder_InterquartileRange** | 18.2493 (6.4004) | 23.9002 (29.4061) | 0.8608 |
| **wavelet-HLL_firstorder_Kurtosis** | 29.0713 (34.9085) | 28.3221 (26.3019) | 0.8109 |
| **wavelet-HLL_firstorder_Maximum** | 130.5670 (90.1218) | 184.9576 (169.0964) | 0.0026 |
| **wavelet-HLL_firstorder_MeanAbsoluteDeviation** | 13.3099 (6.1553) | 18.0464 (19.3538) | 0.1891 |
| **wavelet-HLL_firstorder_Mean** | -4.1840 (5.6613) | -4.9965 (6.8436) | 0.5571 |
| **wavelet-HLL_firstorder_Median** | -1.8888 (2.6408) | -1.7974 (2.6316) | 0.2691 |
| **wavelet-HLL_firstorder_Minimum** | -251.8303 (117.6275) | -309.9103 (185.2457) | 0.0244 |
| **wavelet-HLL_firstorder_Range** | 382.3973 (187.0387) | 494.8680 (336.4893) | 0.0046 |
| **wavelet-HLL_firstorder_RobustMeanAbsoluteDeviation** | 7.8094 (3.1421) | 10.2400 (12.4502) | 0.9853 |
| **wavelet-HLL_firstorder_RootMeanSquared** | 21.2192 (11.0907) | 29.1971 (27.3653) | 0.047 |
| **wavelet-HLL_firstorder_Skewness** | -2.1122 (2.6207) | -2.6105 (2.0686) | 0.0585 |
| **wavelet-HLL_firstorder_TotalEnergy** | $3.53\times{10}^{7}$ ($5.57\times{10}^{7}$) | $5.62\times{10}^{7}$ ($2.09\times{10}^{8}$) | 0.7324 |
| **wavelet-HLL_firstorder_Uniformity** | 0.4099 (0.0628) | 0.3907 (0.0907) | 0.5585 |
| **wavelet-HLL_firstorder_Variance** | 522.5498 (632.8024) | 1527.1760 (4424.7929) | 0.0444 |
| **wavelet-HLL_glcm_Autocorrelation** | 140.7202 (103.7077) | 229.3119 (321.4653) | 0.0281 |
| **wavelet-HLL_glcm_ClusterProminence** | 203.2964 (444.0308) | 2214.9851 ($1.55\times{10}^{4}$) | 0.012 |
| **wavelet-HLL_glcm_ClusterShade** | -12.5272 (25.2142) | -41.4046 (164.1266) | 0.0169 |
| **wavelet-HLL_glcm_ClusterTendency** | 2.2449 (2.7470) | 5.6082 (14.0887) | 0.0438 |
| **wavelet-HLL_glcm_Contrast** | 1.0204 (0.7244) | 3.6160 (12.4351) | 0.0598 |
| **wavelet-HLL_glcm_Correlation** | 0.2457 (0.1823) | 0.2824 (0.1849) | 0.0385 |
| **wavelet-HLL_glcm_DifferenceAverage** | 0.6403 (0.1537) | 0.8570 (0.9765) | 0.246 |
| **wavelet-HLL_glcm_DifferenceEntropy** | 1.2710 (0.2259) | 1.4091 (0.5443) | 0.234 |
| **wavelet-HLL_glcm_DifferenceVariance** | 0.5398 (0.4496) | 1.7521 (5.5570) | 0.0345 |
| **wavelet-HLL_glcm_Id** | 0.7200 (0.0340) | 0.7020 (0.0903) | 0.6987 |
| **wavelet-HLL_glcm_Idm** | 0.7102 (0.0419) | 0.6886 (0.1067) | 0.6828 |
| **wavelet-HLL_glcm_Idmn** | 0.9933 (0.0078) | 0.9944 (0.0079) | 0.0057 |
| **wavelet-HLL_glcm_Idn** | 0.9563 (0.0223) | 0.9601 (0.0198) | 0.0026 |
| **wavelet-HLL_glcm_Imc1** | -0.1223 (0.0360) | -0.1311 (0.0362) | 0.0136 |
| **wavelet-HLL_glcm_Imc2** | 0.4594 (0.1104) | 0.4966 (0.1188) | 0.0259 |
| **wavelet-HLL_glcm_InverseVariance** | 0.4742 (0.0181) | 0.4577 (0.0555) | 0.1355 |
| **wavelet-HLL_glcm_JointAverage** | 10.9225 (4.6242) | 13.2233 (7.3579) | 0.0219 |
| **wavelet-HLL_glcm_JointEnergy** | 0.2064 (0.0479) | 0.1964 (0.0600) | 0.8371 |
| **wavelet-HLL_glcm_JointEntropy** | 2.8139 (0.5738) | 3.1086 (1.2109) | 0.3439 |
| **wavelet-HLL_glcm_MCC** | 0.5249 (0.1280) | 0.5623 (0.1304) | 0.0146 |
| **wavelet-HLL_glcm_MaximumProbability** | 0.2768 (0.0410) | 0.2620 (0.0640) | 0.5038 |
| **wavelet-HLL_glcm_SumAverage** | 21.8450 (9.2484) | 26.4467 (14.7157) | 0.0219 |
| **wavelet-HLL_glcm_SumEntropy** | 2.0092 (0.3817) | 2.1773 (0.6533) | 0.2824 |
| **wavelet-HLL_glcm_SumSquares** | 0.8163 (0.8566) | 2.3061 (6.5445) | 0.0441 |
| **wavelet-HLL_gldm_DependenceEntropy** | 5.4797 (0.3241) | 5.6056 (0.4186) | 0.135 |
| **wavelet-HLL_gldm_DependenceNonUniformity** | 9158.2711 ($1.61\times{10}^{4}$) | 4705.8176 (7993.8372) | 0.007 |
| **wavelet-HLL_gldm_DependenceNonUniformityNormalized** | 0.0636 (0.0071) | 0.0673 (0.0278) | 0.399 |
| **wavelet-HLL_gldm_DependenceVariance** | 21.1865 (4.4362) | 21.5044 (5.4224) | 0.1088 |
| **wavelet-HLL_gldm_GrayLevelNonUniformity** | $5.84\times{10}^{4}$ ($9.97\times{10}^{4}$) | $2.81\times{10}^{4}$ ($4.60\times{10}^{4}$) | 0.0065 |
| **wavelet-HLL_gldm_GrayLevelVariance** | 0.9432 (1.0068) | 2.5501 (7.0752) | 0.044 |
| **wavelet-HLL_gldm_HighGrayLevelEmphasis** | 140.8328 (103.5180) | 230.6094 (325.9351) | 0.0282 |
| **wavelet-HLL_gldm_LargeDependenceEmphasis** | 168.9673 (30.1213) | 166.1405 (42.9894) | 0.469 |
| **wavelet-HLL_gldm_LargeDependenceHighGrayLevelEmphasis** | $2.44\times{10}^{4}$ ($1.84\times{10}^{4}$) | $3.06\times{10}^{4}$ ($2.71\times{10}^{4}$) | 0.0328 |
| **wavelet-HLL_gldm_LargeDependenceLowGrayLevelEmphasis** | 3.3517 (5.8262) | 2.8375 (6.8514) | 0.1225 |
| **wavelet-HLL_gldm_LowGrayLevelEmphasis** | 0.0220 (0.0347) | 0.0175 (0.0444) | 0.0952 |
| **wavelet-HLL_gldm_SmallDependenceEmphasis** | 0.0225 (0.0144) | 0.0347 (0.0588) | 0.6593 |
| **wavelet-HLL_gldm_SmallDependenceHighGrayLevelEmphasis** | 2.8683 (2.9014) | 19.9610 (95.0446) | 0.0328 |
| **wavelet-HLL_gldm_SmallDependenceLowGrayLevelEmphasis** | 0.0008 (0.0015) | 0.0007 (0.0044) | 0.9813 |
| **wavelet-HLL_glrlm_GrayLevelNonUniformity** | $2.97\times{10}^{4}$ ($5.09\times{10}^{4}$) | $1.43\times{10}^{4}$ ($2.30\times{10}^{4}$) | 0.0064 |
| **wavelet-HLL_glrlm_GrayLevelNonUniformityNormalized** | 0.3732 (0.0714) | 0.3511 (0.0917) | 0.3231 |
| **wavelet-HLL_glrlm_GrayLevelVariance** | 1.2981 (1.3480) | 3.1593 (7.4181) | 0.0198 |
| **wavelet-HLL_glrlm_HighGrayLevelRunEmphasis** | 139.6653 (102.7084) | 228.7815 (325.5624) | 0.0296 |
| **wavelet-HLL_glrlm_LongRunEmphasis** | 9.3263 (4.0454) | 9.1273 (4.0494) | 0.3418 |
| **wavelet-HLL_glrlm_LongRunHighGrayLevelEmphasis** | 1383.6683 (1221.2119) | 1664.3742 (1490.7582) | 0.0617 |
| **wavelet-HLL_glrlm_LongRunLowGrayLevelEmphasis** | 0.1645 (0.2353) | 0.1528 (0.2943) | 0.492 |
| **wavelet-HLL_glrlm_LowGrayLevelRunEmphasis** | 0.0229 (0.0359) | 0.0181 (0.0451) | 0.0972 |
| **wavelet-HLL_glrlm_RunEntropy** | 3.4278 (0.2298) | 3.5861 (0.3688) | 0.0015 |
| **wavelet-HLL_glrlm_RunLengthNonUniformity** | $3.05\times{10}^{4}$ ($5.49\times{10}^{4}$) | $1.75\times{10}^{4}$ ($2.91\times{10}^{4}$) | 0.019 |
| **wavelet-HLL_glrlm_RunLengthNonUniformityNormalized** | 0.4180 (0.0610) | 0.4310 (0.1041) | 0.5244 |
| **wavelet-HLL_glrlm_RunPercentage** | 0.5739 (0.0533) | 0.5830 (0.0835) | 0.4725 |
| **wavelet-HLL_glrlm_RunVariance** | 3.5233 (1.7645) | 3.4929 (1.7421) | 0.1943 |
| **wavelet-HLL_glrlm_ShortRunEmphasis** | 0.6382 (0.0584) | 0.6478 (0.0809) | 0.7478 |
| **wavelet-HLL_glrlm_ShortRunHighGrayLevelEmphasis** | 87.9151 (64.9955) | 163.0837 (292.6746) | 0.0282 |
| **wavelet-HLL_glrlm_ShortRunLowGrayLevelEmphasis** | 0.0155 (0.0247) | 0.0118 (0.0295) | 0.0839 |
| **wavelet-HLL_glszm_GrayLevelNonUniformity** | 414.1122 (1154.2341) | 199.9799 (403.6633) | 0.0228 |
| **wavelet-HLL_glszm_GrayLevelNonUniformityNormalized** | 0.2282 (0.1072) | 0.1913 (0.1033) | 0.0109 |
| **wavelet-HLL_glszm_GrayLevelVariance** | 7.2408 (4.8746) | 11.7635 (12.6759) | 0.0015 |
| **wavelet-HLL_glszm_HighGrayLevelZoneEmphasis** | 121.7690 (91.2232) | 203.3144 (321.0117) | 0.0459 |
| **wavelet-HLL_glszm_LargeAreaEmphasis** | $9.60\times{10}^{6}$ ($1.97\times{10}^{7}$) | $4.15\times{10}^{6}$ ($1.08\times{10}^{7}$) | 0.002 |
| **wavelet-HLL_glszm_LargeAreaHighGrayLevelEmphasis** | $1.62\times{10}^{9}$ ($4.05\times{10}^{9}$) | $6.58\times{10}^{8}$ ($2.32\times{10}^{9}$) | 0.0548 |
| **wavelet-HLL_glszm_LargeAreaLowGrayLevelEmphasis** | $8.43\times{10}^{4}$ ($1.66\times{10}^{5}$) | $1.53\times{10}^{5}$ ($1.73\times{10}^{6}$) | 0.0562 |
| **wavelet-HLL_glszm_LowGrayLevelZoneEmphasis** | 0.0455 (0.0850) | 0.0339 (0.0598) | 0.0882 |
| **wavelet-HLL_glszm_SizeZoneNonUniformity** | 165.8004 (280.8101) | 255.0373 (1043.8943) | 0.3745 |
| **wavelet-HLL_glszm_SizeZoneNonUniformityNormalized** | 0.1548 (0.0593) | 0.1682 (0.0694) | 0.8577 |
| **wavelet-HLL_glszm_SmallAreaEmphasis** | 0.3439 (0.1036) | 0.3745 (0.1072) | 0.2944 |
| **wavelet-HLL_glszm_SmallAreaHighGrayLevelEmphasis** | 41.4173 (34.0221) | 87.3995 (188.0577) | 0.0289 |
| **wavelet-HLL_glszm_SmallAreaLowGrayLevelEmphasis** | 0.0160 (0.0276) | 0.0140 (0.0223) | 0.3208 |
| **wavelet-HLL_glszm_ZoneEntropy** | 5.6056 (0.7605) | 5.9597 (0.7912) | < 0.0001 |
| **wavelet-HLL_glszm_ZonePercentage** | 0.0171 (0.0157) | 0.0316 (0.0701) | 0.6414 |
| **wavelet-HLL_glszm_ZoneVariance** | $9.58\times{10}^{6}$ ($1.97\times{10}^{7}$) | $4.12\times{10}^{6}$ ($1.07\times{10}^{7}$) | 0.002 |
| **wavelet-HLL_ngtdm_Busyness** | 90.5820 (149.4907) | 101.7978 (916.2688) | 0.0034 |
| **wavelet-HLL_ngtdm_Coarseness** | 0.0010 (0.0020) | 0.0007 (0.0040) | 0.6593 |
| **wavelet-HLL_ngtdm_Complexity** | 95.4197 (82.7642) | 450.7712 (1803.5220) | 0.0064 |
| **wavelet-HLL_ngtdm_Contrast** | 0.0075 (0.0089) | 0.0097 (0.0233) | 0.0144 |
| **wavelet-HLL_ngtdm_Strength** | 0.1464 (0.3810) | 0.1984 (0.5009) | 0.9074 |
| **wavelet-HLH_firstorder_10Percentile** | -1.1965 (0.7581) | -1.5415 (3.4351) | 0.8057 |
| **wavelet-HLH_firstorder_90Percentile** | 1.2213 (0.9002) | 1.5693 (3.6152) | 0.762 |
| **wavelet-HLH_firstorder_Energy** | $1.76\times{10}^{5}$ ($3.10\times{10}^{5}$) | $2.42\times{10}^{5}$ ($1.00\times{10}^{6}$) | 0.6803 |
| **wavelet-HLH_firstorder_Entropy** | 1.0043 (0.0180) | 1.0166 (0.1215) | 0.4502 |
| **wavelet-HLH_firstorder_InterquartileRange** | 1.1087 (0.6605) | 1.4719 (3.5918) | 0.9081 |
| **wavelet-HLH_firstorder_Kurtosis** | 55.5873 (135.5882) | 164.3989 (403.9034) | 0.0039 |
| **wavelet-HLH_firstorder_Maximum** | 20.1479 (21.5852) | 32.0107 (40.2794) | 0.004 |
| **wavelet-HLH_firstorder_MeanAbsoluteDeviation** | 0.8432 (0.5363) | 1.0977 (2.3154) | 0.9884 |
| **wavelet-HLH_firstorder_Mean** | 0.0219 (0.1108) | 0.0304 (0.1556) | 0.8355 |
| **wavelet-HLH_firstorder_Median** | -0.0002 (0.0318) | -0.0003 (0.0730) | 0.9198 |
| **wavelet-HLH_firstorder_Minimum** | -16.8660 (21.4948) | -26.3134 (35.5733) | 0.0153 |
| **wavelet-HLH_firstorder_Range** | 37.0138 (42.4142) | 58.3241 (74.6570) | 0.0063 |
| **wavelet-HLH_firstorder_RobustMeanAbsoluteDeviation** | 0.4737 (0.2880) | 0.6244 (1.4876) | 0.9081 |
| **wavelet-HLH_firstorder_RootMeanSquared** | 1.4529 (1.1994) | 1.9157 (3.2250) | 0.5079 |
| **wavelet-HLH_firstorder_Skewness** | 0.9199 (2.2912) | 1.5222 (4.9981) | 0.1934 |
| **wavelet-HLH_firstorder_TotalEnergy** | $1.76\times{10}^{5}$ ($3.10\times{10}^{5}$) | $2.42\times{10}^{5}$ ($1.00\times{10}^{6}$) | 0.689 |
| **wavelet-HLH_firstorder_Uniformity** | 0.4998 (0.0021) | 0.4978 (0.0214) | 0.5168 |
| **wavelet-HLH_firstorder_Variance** | 3.5187 (8.3227) | 14.0102 (127.4514) | 0.6385 |
| **wavelet-HLH_glcm_Autocorrelation** | 3.8015 (4.9924) | 6.4831 (14.6969) | 0.0983 |
| **wavelet-HLH_glcm_ClusterProminence** | 0.5971 (0.4339) | 1.5607 (15.2781) | 0.7219 |
| **wavelet-HLH_glcm_ClusterShade** | 0.0047 (0.0188) | 0.0443 (0.6475) | 0.5666 |
| **wavelet-HLH_glcm_ClusterTendency** | 0.5375 (0.0420) | 0.5626 (0.3358) | 0.8724 |
| **wavelet-HLH_glcm_Contrast** | 0.4682 (0.0406) | 0.4917 (0.3479) | 0.8608 |
| **wavelet-HLH_glcm_Correlation** | 0.0689 (0.0737) | 0.0714 (0.0597) | 0.976 |
| **wavelet-HLH_glcm_DifferenceAverage** | 0.4662 (0.0371) | 0.4715 (0.0880) | 0.7971 |
| **wavelet-HLH_glcm_DifferenceEntropy** | 0.9668 (0.0242) | 0.9768 (0.0985) | 0.0808 |
| **wavelet-HLH_glcm_DifferenceVariance** | 0.2399 (0.0162) | 0.2515 (0.1593) | 0.5058 |
| **wavelet-HLH_glcm_Id** | 0.7671 (0.0185) | 0.7662 (0.0234) | 0.805 |
| **wavelet-HLH_glcm_Idm** | 0.7671 (0.0185) | 0.7658 (0.0266) | 0.7943 |
| **wavelet-HLH_glcm_Idmn** | 0.9201 (0.0286) | 0.9325 (0.0339) | 0.0026 |
| **wavelet-HLH_glcm_Idn** | 0.8587 (0.0321) | 0.8718 (0.0376) | 0.0048 |
| **wavelet-HLH_glcm_Imc1** | -0.0369 (0.0186) | -0.0364 (0.0166) | 0.0303 |
| **wavelet-HLH_glcm_Imc2** | 0.1992 (0.0526) | 0.1960 (0.0562) | 0.0224 |
| **wavelet-HLH_glcm_InverseVariance** | 0.4654 (0.0370) | 0.4641 (0.0301) | 0.8467 |
| **wavelet-HLH_glcm_JointAverage** | 1.7754 (0.8002) | 2.1003 (1.4356) | 0.0392 |
| **wavelet-HLH_glcm_JointEnergy** | 0.2622 (0.0066) | 0.2605 (0.0164) | 0.0487 |
| **wavelet-HLH_glcm_JointEntropy** | 1.9692 (0.0330) | 1.9916 (0.2244) | 0.1919 |
| **wavelet-HLH_glcm_MCC** | 0.1909 (0.0779) | 0.1906 (0.0730) | 0.6496 |
| **wavelet-HLH_glcm_MaximumProbability** | 0.2969 (0.0149) | 0.2958 (0.0216) | 0.2336 |
| **wavelet-HLH_glcm_SumAverage** | 3.5508 (1.6004) | 4.2006 (2.8713) | 0.0392 |
| **wavelet-HLH_glcm_SumEntropy** | 1.5018 (0.0316) | 1.5149 (0.1136) | 0.1901 |
| **wavelet-HLH_glcm_SumSquares** | 0.2514 (0.0093) | 0.2636 (0.1702) | 0.7708 |
| **wavelet-HLH_gldm_DependenceEntropy** | 4.9319 (0.2128) | 4.9774 (0.2042) | 0.3731 |
| **wavelet-HLH_gldm_DependenceNonUniformity** | $1.23\times{10}^{4}$ ($2.25\times{10}^{4}$) | 5878.6648 (9950.4347) | 0.0079 |
| **wavelet-HLH_gldm_DependenceNonUniformityNormalized** | 0.0782 (0.0139) | 0.0761 (0.0125) | 0.5869 |
| **wavelet-HLH_gldm_DependenceVariance** | 14.9399 (4.9157) | 15.5633 (4.4563) | 0.6128 |
| **wavelet-HLH_gldm_GrayLevelNonUniformity** | $6.62\times{10}^{4}$ ($1.15\times{10}^{5}$) | $3.41\times{10}^{4}$ ($5.20\times{10}^{4}$) | 0.0103 |
| **wavelet-HLH_gldm_GrayLevelVariance** | 0.2519 (0.0109) | 0.2658 (0.1908) | 0.712 |
| **wavelet-HLH_gldm_HighGrayLevelEmphasis** | 4.0430 (5.0048) | 6.7395 (14.7859) | 0.0983 |
| **wavelet-HLH_gldm_LargeDependenceEmphasis** | 198.7527 (20.5506) | 202.7473 (24.6339) | 0.1427 |
| **wavelet-HLH_gldm_LargeDependenceHighGrayLevelEmphasis** | 797.3104 (1018.6381) | 1236.0123 (2414.0492) | 0.0951 |
| **wavelet-HLH_gldm_LargeDependenceLowGrayLevelEmphasis** | 111.8070 (39.3122) | 102.5086 (49.4637) | 0.0357 |
| **wavelet-HLH_gldm_LowGrayLevelEmphasis** | 0.5543 (0.1806) | 0.4915 (0.2281) | 0.0076 |
| **wavelet-HLH_gldm_SmallDependenceEmphasis** | 0.0085 (0.0020) | 0.0094 (0.0126) | 0.7698 |
| **wavelet-HLH_gldm_SmallDependenceHighGrayLevelEmphasis** | 0.0355 (0.0510) | 0.1266 (0.9249) | 0.2204 |
| **wavelet-HLH_gldm_SmallDependenceLowGrayLevelEmphasis** | 0.0048 (0.0019) | 0.0041 (0.0024) | 0.0204 |
| **wavelet-HLH_glrlm_GrayLevelNonUniformity** | $3.38\times{10}^{4}$ ($5.86\times{10}^{4}$) | $1.74\times{10}^{4}$ ($2.66\times{10}^{4}$) | 0.0101 |
| **wavelet-HLH_glrlm_GrayLevelNonUniformityNormalized** | 0.4994 (0.0036) | 0.4967 (0.0236) | 0.3776 |
| **wavelet-HLH_glrlm_GrayLevelVariance** | 0.2536 (0.0205) | 0.2708 (0.2179) | 0.7403 |
| **wavelet-HLH_glrlm_HighGrayLevelRunEmphasis** | 4.0619 (5.0158) | 6.7674 (14.8150) | 0.0973 |
| **wavelet-HLH_glrlm_LongRunEmphasis** | 6.4519 (0.7979) | 6.7478 (0.9516) | 0.0621 |
| **wavelet-HLH_glrlm_LongRunHighGrayLevelEmphasis** | 25.7600 (32.2151) | 46.8320 (125.9361) | 0.0838 |
| **wavelet-HLH_glrlm_LongRunLowGrayLevelEmphasis** | 3.6253 (1.2975) | 3.3693 (1.6348) | 0.0597 |
| **wavelet-HLH_glrlm_LowGrayLevelRunEmphasis** | 0.5512 (0.1792) | 0.4885 (0.2262) | 0.0074 |
| **wavelet-HLH_glrlm_RunEntropy** | 2.9359 (0.1086) | 2.9731 (0.0959) | 0.0067 |
| **wavelet-HLH_glrlm_RunLengthNonUniformity** | $2.44\times{10}^{4}$ ($4.23\times{10}^{4}$) | $1.26\times{10}^{4}$ ($1.94\times{10}^{4}$) | 0.0099 |
| **wavelet-HLH_glrlm_RunLengthNonUniformityNormalized** | 0.3516 (0.0243) | 0.3490 (0.0340) | 0.1559 |
| **wavelet-HLH_glrlm_RunPercentage** | 0.5177 (0.0271) | 0.5127 (0.0324) | 0.0327 |
| **wavelet-HLH_glrlm_RunVariance** | 2.0819 (0.3568) | 2.2331 (0.4017) | 0.0044 |
| **wavelet-HLH_glrlm_ShortRunEmphasis** | 0.5844 (0.0256) | 0.5823 (0.0308) | 0.5178 |
| **wavelet-HLH_glrlm_ShortRunHighGrayLevelEmphasis** | 2.3827 (2.9565) | 4.1327 (9.7811) | 0.0967 |
| **wavelet-HLH_glrlm_ShortRunLowGrayLevelEmphasis** | 0.3213 (0.1058) | 0.2818 (0.1300) | 0.0049 |
| **wavelet-HLH_glszm_GrayLevelNonUniformity** | 12.5728 (9.9877) | 12.4138 (30.1674) | 0.157 |
| **wavelet-HLH_glszm_GrayLevelNonUniformityNormalized** | 0.5224 (0.1369) | 0.4865 (0.1521) | 0.062 |
| **wavelet-HLH_glszm_GrayLevelVariance** | 0.4839 (0.8167) | 0.8127 (1.7423) | 0.1608 |
| **wavelet-HLH_glszm_HighGrayLevelZoneEmphasis** | 4.2537 (5.9603) | 7.2957 (16.5134) | 0.0982 |
| **wavelet-HLH_glszm_LargeAreaEmphasis** | $6.12\times{10}^{8}$ ($1.79\times{10}^{9}$) | $1.95\times{10}^{8}$ ($7.92\times{10}^{8}$) | 0.0298 |
| **wavelet-HLH_glszm_LargeAreaHighGrayLevelEmphasis** | $1.80\times{10}^{9}$ ($4.69\times{10}^{9}$) | $9.60\times{10}^{8}$ ($3.59\times{10}^{9}$) | 0.162 |
| **wavelet-HLH_glszm_LargeAreaLowGrayLevelEmphasis** | $3.63\times{10}^{8}$ ($1.12\times{10}^{9}$) | $1.00\times{10}^{8}$ ($4.87\times{10}^{8}$) | 0.0236 |
| **wavelet-HLH_glszm_LowGrayLevelZoneEmphasis** | 0.5899 (0.2043) | 0.5425 (0.2468) | 0.0605 |
| **wavelet-HLH_glszm_SizeZoneNonUniformity** | 9.5796 (9.9132) | 11.8605 (33.2411) | 0.5201 |
| **wavelet-HLH_glszm_SizeZoneNonUniformityNormalized** | 0.3344 (0.1013) | 0.3331 (0.1049) | 0.7768 |
| **wavelet-HLH_glszm_SmallAreaEmphasis** | 0.5185 (0.1718) | 0.5332 (0.1492) | 0.4031 |
| **wavelet-HLH_glszm_SmallAreaHighGrayLevelEmphasis** | 2.3049 (3.3854) | 4.4301 (12.2895) | 0.0992 |
| **wavelet-HLH_glszm_SmallAreaLowGrayLevelEmphasis** | 0.2946 (0.1402) | 0.2836 (0.1555) | 0.3577 |
| **wavelet-HLH_glszm_ZoneEntropy** | 2.7274 (0.7047) | 2.8187 (0.6699) | 0.1487 |
| **wavelet-HLH_glszm_ZonePercentage** | 0.0012 (0.0015) | 0.0022 (0.0130) | 0.7271 |
| **wavelet-HLH_glszm_ZoneVariance** | $5.85\times{10}^{8}$ ($1.74\times{10}^{9}$) | $1.77\times{10}^{8}$ ($7.56\times{10}^{8}$) | 0.0261 |
| **wavelet-HLH_ngtdm_Busyness** | $2.78\times{10}^{4}$ ($5.69\times{10}^{4}$) | 9315.4574 ($1.52\times{10}^{4}$) | 0.0001 |
| **wavelet-HLH_ngtdm_Coarseness** | 0.0008 (0.0016) | 0.0006 (0.0039) | 0.8239 |
| **wavelet-HLH_ngtdm_Complexity** | 1.7856 (4.0828) | 4.8808 (19.6488) | 0.0783 |
| **wavelet-HLH_ngtdm_Contrast** | 0.0965 (0.0395) | 0.0792 (0.0468) | 0.0024 |
| **wavelet-HLH_ngtdm_Strength** | 0.0014 (0.0029) | 0.0050 (0.0291) | 0.4114 |
| **wavelet-HHL_firstorder_10Percentile** | -11.7460 (3.4226) | -16.4999 (28.7817) | 0.3973 |
| **wavelet-HHL_firstorder_90Percentile** | 11.6714 (3.4667) | 16.3358 (28.8146) | 0.2867 |
| **wavelet-HHL_firstorder_Energy** | $1.67\times{10}^{7}$ ($4.43\times{10}^{7}$) | $3.43\times{10}^{7}$ ($2.82\times{10}^{8}$) | 0.0234 |
| **wavelet-HHL_firstorder_Entropy** | 1.1287 (0.1351) | 1.2498 (0.6245) | 0.985 |
| **wavelet-HHL_firstorder_InterquartileRange** | 12.1877 (3.6056) | 16.9459 (30.0086) | 0.2831 |
| **wavelet-HHL_firstorder_Kurtosis** | 6.0755 (7.5050) | 8.0734 (9.8279) | 0.099 |
| **wavelet-HHL_firstorder_Maximum** | 72.0986 (38.1937) | 99.4334 (107.6655) | 0.0615 |
| **wavelet-HHL_firstorder_MeanAbsoluteDeviation** | 7.3884 (2.1217) | 10.4018 (17.9580) | 0.4756 |
| **wavelet-HHL_firstorder_Mean** | -0.0355 (0.2907) | -0.0299 (0.4225) | 0.9301 |
| **wavelet-HHL_firstorder_Median** | -0.0436 (0.1879) | -0.0786 (0.5061) | 0.2067 |
| **wavelet-HHL_firstorder_Minimum** | -56.2056 (26.0807) | -80.7372 (98.1730) | 0.052 |
| **wavelet-HHL_firstorder_Range** | 128.3042 (58.7918) | 180.1706 (202.9570) | 0.0473 |
| **wavelet-HHL_firstorder_RobustMeanAbsoluteDeviation** | 5.0635 (1.4999) | 7.0436 (12.4329) | 0.2822 |
| **wavelet-HHL_firstorder_RootMeanSquared** | 9.5185 (2.6092) | 13.6088 (22.7402) | 0.9382 |
| **wavelet-HHL_firstorder_Skewness** | 0.1413 (0.6998) | 0.2706 (0.5751) | 0.0768 |
| **wavelet-HHL_firstorder_TotalEnergy** | $1.67\times{10}^{7}$ ($4.43\times{10}^{7}$) | $3.43\times{10}^{7}$ ($2.82\times{10}^{8}$) | 0.0234 |
| **wavelet-HHL_firstorder_Uniformity** | 0.4805 (0.0255) | 0.4649 (0.0850) | 0.7005 |
| **wavelet-HHL_firstorder_Variance** | 97.2384 (54.6837) | 700.4028 (3585.7662) | 0.93 |
| **wavelet-HHL_glcm_Autocorrelation** | 11.8022 (8.6471) | 33.4317 (101.3797) | 0.0424 |
| **wavelet-HHL_glcm_ClusterProminence** | 1.0882 (0.7866) | 533.7246 (4590.1138) | 0.191 |
| **wavelet-HHL_glcm_ClusterShade** | 0.0118 (0.0964) | 0.3051 (3.0307) | 0.0055 |
| **wavelet-HHL_glcm_ClusterTendency** | 0.5840 (0.1163) | 2.4556 (11.0609) | 0.4845 |
| **wavelet-HHL_glcm_Contrast** | 0.5859 (0.1244) | 2.5649 (11.7674) | 0.9144 |
| **wavelet-HHL_glcm_Correlation** | -0.0015 (0.0199) | 0.0068 (0.0314) | 0.0258 |
| **wavelet-HHL_glcm_DifferenceAverage** | 0.5379 (0.0529) | 0.7139 (0.9457) | 0.5246 |
| **wavelet-HHL_glcm_DifferenceEntropy** | 1.0368 (0.1169) | 1.1382 (0.5259) | 0.8206 |
| **wavelet-HHL_glcm_DifferenceVariance** | 0.2692 (0.0571) | 1.0219 (4.3785) | 0.4457 |
| **wavelet-HHL_glcm_Id** | 0.7384 (0.0157) | 0.7236 (0.0867) | 0.2926 |
| **wavelet-HHL_glcm_Idm** | 0.7358 (0.0194) | 0.7179 (0.1031) | 0.3304 |
| **wavelet-HHL_glcm_Idmn** | 0.9778 (0.0193) | 0.9794 (0.0179) | 0.0852 |
| **wavelet-HHL_glcm_Idn** | 0.9173 (0.0274) | 0.9205 (0.0277) | 0.0344 |
| **wavelet-HHL_glcm_Imc1** | -0.0741 (0.0100) | -0.0729 (0.0146) | 0.5314 |
| **wavelet-HHL_glcm_Imc2** | 0.2894 (0.0417) | 0.2937 (0.0893) | 0.9007 |
| **wavelet-HHL_glcm_InverseVariance** | 0.5016 (0.0054) | 0.4870 (0.0571) | 0.0639 |
| **wavelet-HHL_glcm_JointAverage** | 3.2692 (1.0628) | 4.2625 (3.9169) | 0.0315 |
| **wavelet-HHL_glcm_JointEnergy** | 0.2539 (0.0242) | 0.2446 (0.0540) | 0.5948 |
| **wavelet-HHL_glcm_JointEntropy** | 2.1583 (0.2570) | 2.3872 (1.1889) | 0.8406 |
| **wavelet-HHL_glcm_MCC** | 0.3096 (0.0777) | 0.3187 (0.1062) | 0.3755 |
| **wavelet-HHL_glcm_MaximumProbability** | 0.2973 (0.0189) | 0.2847 (0.0577) | 0.8808 |
| **wavelet-HHL_glcm_SumAverage** | 6.5383 (2.1256) | 8.5249 (7.8338) | 0.0315 |
| **wavelet-HHL_glcm_SumEntropy** | 1.5466 (0.1305) | 1.6722 (0.6107) | 0.9267 |
| **wavelet-HHL_glcm_SumSquares** | 0.2925 (0.0598) | 1.2551 (5.7069) | 0.6758 |
| **wavelet-HHL_gldm_DependenceEntropy** | 4.7335 (0.1732) | 4.8238 (0.4315) | 0.9577 |
| **wavelet-HHL_gldm_DependenceNonUniformity** | $1.28\times{10}^{4}$ ($2.11\times{10}^{4}$) | 6799.3555 ($1.11\times{10}^{4}$) | 0.0107 |
| **wavelet-HHL_gldm_DependenceNonUniformityNormalized** | 0.0915 (0.0110) | 0.0958 (0.0246) | 0.409 |
| **wavelet-HHL_gldm_DependenceVariance** | 11.1686 (2.4391) | 11.0215 (3.1152) | 0.8282 |
| **wavelet-HHL_gldm_GrayLevelNonUniformity** | $6.18\times{10}^{4}$ ($1.02\times{10}^{5}$) | $3.17\times{10}^{4}$ ($4.88\times{10}^{4}$) | 0.0111 |
| **wavelet-HHL_gldm_GrayLevelVariance** | 0.2958 (0.0602) | 1.2682 (5.7270) | 0.5588 |
| **wavelet-HHL_gldm_HighGrayLevelEmphasis** | 12.0999 (8.6718) | 34.7353 (106.9638) | 0.0426 |
| **wavelet-HHL_gldm_LargeDependenceEmphasis** | 164.8944 (18.3361) | 163.9527 (34.4984) | 0.0248 |
| **wavelet-HHL_gldm_LargeDependenceHighGrayLevelEmphasis** | 2015.6856 (1484.1230) | 2835.1307 (3414.1216) | 0.0314 |
| **wavelet-HHL_gldm_LargeDependenceLowGrayLevelEmphasis** | 22.9030 (17.0751) | 22.7486 (21.4698) | 0.3752 |
| **wavelet-HHL_gldm_LowGrayLevelEmphasis** | 0.1466 (0.1249) | 0.1351 (0.1260) | 0.1584 |
| **wavelet-HHL_gldm_SmallDependenceEmphasis** | 0.0124 (0.0052) | 0.0225 (0.0559) | 0.5418 |
| **wavelet-HHL_gldm_SmallDependenceHighGrayLevelEmphasis** | 0.1629 (0.1616) | 6.2331 (37.8295) | 0.1273 |
| **wavelet-HHL_gldm_SmallDependenceLowGrayLevelEmphasis** | 0.0023 (0.0022) | 0.0019 (0.0032) | 0.3228 |
| **wavelet-HHL_glrlm_GrayLevelNonUniformity** | $3.26\times{10}^{4}$ ($5.34\times{10}^{4}$) | $1.68\times{10}^{4}$ ($2.54\times{10}^{4}$) | 0.0105 |
| **wavelet-HHL_glrlm_GrayLevelNonUniformityNormalized** | 0.4695 (0.0356) | 0.4544 (0.0874) | 0.8592 |
| **wavelet-HHL_glrlm_GrayLevelVariance** | 0.3235 (0.0889) | 1.3313 (5.8510) | 0.4187 |
| **wavelet-HHL_glrlm_HighGrayLevelRunEmphasis** | 12.1431 (8.6836) | 34.8323 (107.0674) | 0.0418 |
| **wavelet-HHL_glrlm_LongRunEmphasis** | 11.0467 (4.7449) | 11.3171 (4.9127) | 0.0373 |
| **wavelet-HHL_glrlm_LongRunHighGrayLevelEmphasis** | 139.2155 (122.0148) | 203.8162 (255.1562) | 0.0208 |
| **wavelet-HHL_glrlm_LongRunLowGrayLevelEmphasis** | 1.4213 (0.9313) | 1.5407 (1.4656) | 0.6496 |
| **wavelet-HHL_glrlm_LowGrayLevelRunEmphasis** | 0.1478 (0.1243) | 0.1357 (0.1261) | 0.1514 |
| **wavelet-HHL_glrlm_RunEntropy** | 2.9623 (0.1611) | 3.0644 (0.3605) | 0.0194 |
| **wavelet-HHL_glrlm_RunLengthNonUniformity** | $2.98\times{10}^{4}$ ($5.52\times{10}^{4}$) | $1.62\times{10}^{4}$ ($2.88\times{10}^{4}$) | 0.0094 |
| **wavelet-HHL_glrlm_RunLengthNonUniformityNormalized** | 0.3908 (0.0336) | 0.4020 (0.0963) | 0.0118 |
| **wavelet-HHL_glrlm_RunPercentage** | 0.5627 (0.0327) | 0.5682 (0.0746) | 0.0066 |
| **wavelet-HHL_glrlm_RunVariance** | 3.7399 (1.8717) | 3.8318 (1.8549) | 0.0391 |
| **wavelet-HHL_glrlm_ShortRunEmphasis** | 0.6132 (0.0324) | 0.6195 (0.0699) | 0.0353 |
| **wavelet-HHL_glrlm_ShortRunHighGrayLevelEmphasis** | 7.4387 (5.3594) | 27.7716 (101.6609) | 0.0484 |
| **wavelet-HHL_glrlm_ShortRunLowGrayLevelEmphasis** | 0.0930 (0.0816) | 0.0826 (0.0764) | 0.0904 |
| **wavelet-HHL_glszm_GrayLevelNonUniformity** | 395.7022 (1328.7151) | 157.9199 (414.9964) | 0.0243 |
| **wavelet-HHL_glszm_GrayLevelNonUniformityNormalized** | 0.3972 (0.0822) | 0.3536 (0.1082) | 0.0182 |
| **wavelet-HHL_glszm_GrayLevelVariance** | 2.1429 (0.6136) | 3.8048 (7.6863) | 0.0348 |
| **wavelet-HHL_glszm_HighGrayLevelZoneEmphasis** | 14.2487 (8.9979) | 38.2434 (108.7104) | 0.023 |
| **wavelet-HHL_glszm_LargeAreaEmphasis** | $3.88\times{10}^{7}$ ($1.02\times{10}^{8}$) | $3.94\times{10}^{7}$ ($1.74\times{10}^{8}$) | 0.2434 |
| **wavelet-HHL_glszm_LargeAreaHighGrayLevelEmphasis** | $7.24\times{10}^{8}$ ($2.71\times{10}^{9}$) | $3.58\times{10}^{8}$ ($1.00\times{10}^{9}$) | 0.1039 |
| **wavelet-HHL_glszm_LargeAreaLowGrayLevelEmphasis** | $3.11\times{10}^{6}$ ($6.65\times{10}^{6}$) | $9.93\times{10}^{6}$ ($8.72\times{10}^{7}$) | 0.7996 |
| **wavelet-HHL_glszm_LowGrayLevelZoneEmphasis** | 0.2808 (0.1908) | 0.2459 (0.1853) | 0.0411 |
| **wavelet-HHL_glszm_SizeZoneNonUniformity** | 107.9984 (309.9741) | 203.0095 (1460.1182) | 0.0473 |
| **wavelet-HHL_glszm_SizeZoneNonUniformityNormalized** | 0.1989 (0.0846) | 0.1958 (0.0770) | 0.0771 |
| **wavelet-HHL_glszm_SmallAreaEmphasis** | 0.3164 (0.1212) | 0.3476 (0.1254) | 0.2893 |
| **wavelet-HHL_glszm_SmallAreaHighGrayLevelEmphasis** | 4.7280 (3.1867) | 18.3792 (68.9395) | 0.0272 |
| **wavelet-HHL_glszm_SmallAreaLowGrayLevelEmphasis** | 0.0858 (0.0716) | 0.0861 (0.0793) | 0.6011 |
| **wavelet-HHL_glszm_ZoneEntropy** | 3.8506 (0.9003) | 4.0973 (1.0924) | 0.0294 |
| **wavelet-HHL_glszm_ZonePercentage** | 0.0053 (0.0059) | 0.0171 (0.0657) | 0.3963 |
| **wavelet-HHL_glszm_ZoneVariance** | $3.78\times{10}^{7}$ ($9.87\times{10}^{7}$) | $3.51\times{10}^{7}$ ($1.45\times{10}^{8}$) | 0.2023 |
| **wavelet-HHL_ngtdm_Busyness** | 870.1088 (1195.3920) | 694.4284 (1413.1421) | 0.0589 |
| **wavelet-HHL_ngtdm_Coarseness** | 0.0007 (0.0013) | 0.0005 (0.0029) | 0.7486 |
| **wavelet-HHL_ngtdm_Complexity** | 11.2445 (9.9914) | 138.0068 (750.2489) | 0.0741 |
| **wavelet-HHL_ngtdm_Contrast** | 0.0179 (0.0234) | 0.0204 (0.0328) | 0.1363 |
| **wavelet-HHL_ngtdm_Strength** | 0.0056 (0.0099) | 0.0247 (0.1165) | 0.4177 |
| **wavelet-HHH_firstorder_10Percentile** | -0.7620 (0.3989) | -1.0725 (2.6727) | 0.9543 |
| **wavelet-HHH_firstorder_90Percentile** | 0.7637 (0.4202) | 1.0760 (2.6423) | 0.9051 |
| **wavelet-HHH_firstorder_Energy** | $6.91\times{10}^{4}$ ($1.56\times{10}^{5}$) | $1.19\times{10}^{5}$ ($6.31\times{10}^{5}$) | 0.6266 |
| **wavelet-HHH_firstorder_Entropy** | 1.0006 (0.0054) | 1.0087 (0.0856) | 0.6682 |
| **wavelet-HHH_firstorder_InterquartileRange** | 0.7742 (0.3841) | 1.0851 (2.6854) | 0.9175 |
| **wavelet-HHH_firstorder_Kurtosis** | 18.5052 (37.6801) | 47.5387 (112.2097) | 0.006 |
| **wavelet-HHH_firstorder_Maximum** | 6.7159 (6.9568) | 12.2500 (25.8321) | 0.0245 |
| **wavelet-HHH_firstorder_MeanAbsoluteDeviation** | 0.4953 (0.2690) | 0.6982 (1.6683) | 0.9485 |
| **wavelet-HHH_firstorder_Mean** | 0.0011 (0.0066) | $-3.76\times{10}^{-5}$ (0.0206) | 0.8654 |
| **wavelet-HHH_firstorder_Median** | 0.0001 (0.0063) | 0.0020 (0.0277) | 0.4563 |
| **wavelet-HHH_firstorder_Minimum** | -6.8707 (7.2835) | -12.6049 (25.7797) | 0.0131 |
| **wavelet-HHH_firstorder_Range** | 13.5866 (14.1007) | 24.8549 (51.4442) | 0.0165 |
| **wavelet-HHH_firstorder_RobustMeanAbsoluteDeviation** | 0.3227 (0.1626) | 0.4528 (1.1228) | 0.9346 |
| **wavelet-HHH_firstorder_RootMeanSquared** | 0.7057 (0.4725) | 1.0283 (2.2697) | 0.8328 |
| **wavelet-HHH_firstorder_Skewness** | 0.0206 (0.3800) | -0.0104 (1.0606) | 0.7186 |
| **wavelet-HHH_firstorder_TotalEnergy** | $6.91\times{10}^{4}$ ($1.56\times{10}^{5}$) | $1.19\times{10}^{5}$ ($6.31\times{10}^{5}$) | 0.6324 |
| **wavelet-HHH_firstorder_Uniformity** | 0.5000 (0.0006) | 0.4985 (0.0167) | 0.8966 |
| **wavelet-HHH_firstorder_Variance** | 0.7184 (1.7302) | 6.1913 (51.3246) | 0.5393 |
| **wavelet-HHH_glcm_Autocorrelation** | 2.2989 (0.4497) | 3.9042 (9.6729) | 0.0544 |
| **wavelet-HHH_glcm_ClusterProminence** | 0.4928 (0.0129) | 0.6365 (1.4415) | 0.4807 |
| **wavelet-HHH_glcm_ClusterShade** | -0.0009 (0.0057) | -0.0011 (0.0185) | 0.3552 |
| **wavelet-HHH_glcm_ClusterTendency** | 0.4916 (0.0054) | 0.5030 (0.1237) | 0.905 |
| **wavelet-HHH_glcm_Contrast** | 0.5087 (0.0055) | 0.5198 (0.1362) | 0.9 |
| **wavelet-HHH_glcm_Correlation** | -0.0170 (0.0101) | -0.0159 (0.0109) | 0.9989 |
| **wavelet-HHH_glcm_DifferenceAverage** | 0.5085 (0.0052) | 0.5118 (0.0468) | 0.9992 |
| **wavelet-HHH_glcm_DifferenceEntropy** | 0.9805 (0.0078) | 0.9853 (0.0696) | 0.6172 |
| **wavelet-HHH_glcm_DifferenceVariance** | 0.2433 (0.0026) | 0.2482 (0.0578) | 0.5474 |
| **wavelet-HHH_glcm_Id** | 0.7458 (0.0026) | 0.7451 (0.0123) | 0.9703 |
| **wavelet-HHH_glcm_Idm** | 0.7457 (0.0026) | 0.7448 (0.0152) | 0.9785 |
| **wavelet-HHH_glcm_Idmn** | 0.8999 (0.0098) | 0.9059 (0.0228) | 0.0993 |
| **wavelet-HHH_glcm_Idn** | 0.8319 (0.0090) | 0.8385 (0.0250) | 0.0985 |
| **wavelet-HHH_glcm_Imc1** | -0.0201 (0.0068) | -0.0220 (0.0143) | 0.8429 |
| **wavelet-HHH_glcm_Imc2** | 0.1539 (0.0363) | 0.1616 (0.0545) | 0.552 |
| **wavelet-HHH_glcm_InverseVariance** | 0.5084 (0.0052) | 0.5077 (0.0057) | 0.9352 |
| **wavelet-HHH_glcm_JointAverage** | 1.5135 (0.1125) | 1.7184 (0.9792) | 0.0548 |
| **wavelet-HHH_glcm_JointEnergy** | 0.2568 (0.0023) | 0.2563 (0.0136) | 0.8018 |
| **wavelet-HHH_glcm_JointEntropy** | 1.9807 (0.0105) | 1.9937 (0.1626) | 0.6331 |
| **wavelet-HHH_glcm_MCC** | 0.1330 (0.0443) | 0.1405 (0.0571) | 0.6845 |
| **wavelet-HHH_glcm_MaximumProbability** | 0.2841 (0.0073) | 0.2842 (0.0152) | 0.997 |
| **wavelet-HHH_glcm_SumAverage** | 3.0270 (0.2249) | 3.4368 (1.9583) | 0.0548 |
| **wavelet-HHH_glcm_SumEntropy** | 1.4719 (0.0109) | 1.4783 (0.0815) | 0.7819 |
| **wavelet-HHH_glcm_SumSquares** | 0.2501 (0.0010) | 0.2557 (0.0649) | 0.6094 |
| **wavelet-HHH_gldm_DependenceEntropy** | 4.3716 (0.2066) | 4.3812 (0.1940) | 0.3399 |
| **wavelet-HHH_gldm_DependenceNonUniformity** | $1.81\times{10}^{4}$ ($3.20\times{10}^{4}$) | 8980.6963 ($1.48\times{10}^{4}$) | 0.0099 |
| **wavelet-HHH_gldm_DependenceNonUniformityNormalized** | 0.1172 (0.0205) | 0.1172 (0.0171) | 0.2533 |
| **wavelet-HHH_gldm_DependenceVariance** | 7.1526 (2.0832) | 7.1450 (1.7300) | 0.2173 |
| **wavelet-HHH_gldm_GrayLevelNonUniformity** | $6.62\times{10}^{4}$ ($1.15\times{10}^{5}$) | $3.42\times{10}^{4}$ ($5.20\times{10}^{4}$) | 0.0103 |
| **wavelet-HHH_gldm_GrayLevelVariance** | 0.2501 (0.0012) | 0.2564 (0.0688) | 0.5761 |
| **wavelet-HHH_gldm_HighGrayLevelEmphasis** | 2.5519 (0.4506) | 4.1639 (9.7043) | 0.0537 |
| **wavelet-HHH_gldm_LargeDependenceEmphasis** | 164.6174 (15.4486) | 166.7865 (15.1415) | 0.1405 |
| **wavelet-HHH_gldm_LargeDependenceHighGrayLevelEmphasis** | 421.1571 (83.2424) | 672.4200 (1550.6859) | 0.0272 |
| **wavelet-HHH_gldm_LargeDependenceLowGrayLevelEmphasis** | 101.6418 (13.0958) | 96.5465 (25.8098) | 0.348 |
| **wavelet-HHH_gldm_LowGrayLevelEmphasis** | 0.6191 (0.0499) | 0.5775 (0.1457) | 0.0537 |
| **wavelet-HHH_gldm_SmallDependenceEmphasis** | 0.0082 (0.0019) | 0.0088 (0.0084) | 0.9644 |
| **wavelet-HHH_gldm_SmallDependenceHighGrayLevelEmphasis** | 0.0210 (0.0060) | 0.0632 (0.3841) | 0.3674 |
| **wavelet-HHH_gldm_SmallDependenceLowGrayLevelEmphasis** | 0.0051 (0.0013) | 0.0048 (0.0041) | 0.8373 |
| **wavelet-HHH_glrlm_GrayLevelNonUniformity** | $3.52\times{10}^{4}$ ($6.04\times{10}^{4}$) | $1.83\times{10}^{4}$ ($2.74\times{10}^{4}$) | 0.0104 |
| **wavelet-HHH_glrlm_GrayLevelNonUniformityNormalized** | 0.4999 (0.0010) | 0.4981 (0.0192) | 0.5572 |
| **wavelet-HHH_glrlm_GrayLevelVariance** | 0.2502 (0.0019) | 0.2589 (0.0862) | 0.5258 |
| **wavelet-HHH_glrlm_HighGrayLevelRunEmphasis** | 2.5495 (0.4516) | 4.1648 (9.7146) | 0.0528 |
| **wavelet-HHH_glrlm_LongRunEmphasis** | 5.0077 (0.6525) | 5.1738 (0.7857) | 0.1142 |
| **wavelet-HHH_glrlm_LongRunHighGrayLevelEmphasis** | 12.7894 (2.7288) | 25.2754 (84.9269) | 0.022 |
| **wavelet-HHH_glrlm_LongRunLowGrayLevelEmphasis** | 3.0971 (0.4856) | 2.9473 (0.7778) | 0.4313 |
| **wavelet-HHH_glrlm_LowGrayLevelRunEmphasis** | 0.6197 (0.0497) | 0.5779 (0.1458) | 0.0514 |
| **wavelet-HHH_glrlm_RunEntropy** | 2.7747 (0.1084) | 2.8009 (0.0795) | 0.0251 |
| **wavelet-HHH_glrlm_RunLengthNonUniformity** | $2.62\times{10}^{4}$ ($4.41\times{10}^{4}$) | $1.38\times{10}^{4}$ ($2.03\times{10}^{4}$) | 0.0106 |
| **wavelet-HHH_glrlm_RunLengthNonUniformityNormalized** | 0.3837 (0.0252) | 0.3807 (0.0242) | 0.0235 |
| **wavelet-HHH_glrlm_RunPercentage** | 0.5566 (0.0275) | 0.5525 (0.0234) | 0.0198 |
| **wavelet-HHH_glrlm_RunVariance** | 1.5314 (0.2760) | 1.6074 (0.3261) | 0.0577 |
| **wavelet-HHH_glrlm_ShortRunEmphasis** | 0.6251 (0.0246) | 0.6216 (0.0215) | 0.0293 |
| **wavelet-HHH_glrlm_ShortRunHighGrayLevelEmphasis** | 1.5928 (0.2904) | 2.6321 (6.3021) | 0.0799 |
| **wavelet-HHH_glrlm_ShortRunLowGrayLevelEmphasis** | 0.3877 (0.0351) | 0.3589 (0.0908) | 0.0206 |
| **wavelet-HHH_glszm_GrayLevelNonUniformity** | 2.1404 (1.6143) | 3.4479 (11.5552) | 0.556 |
| **wavelet-HHH_glszm_GrayLevelNonUniformityNormalized** | 0.5252 (0.0614) | 0.5092 (0.0913) | 0.5584 |
| **wavelet-HHH_glszm_GrayLevelVariance** | 0.2579 (0.1660) | 0.4849 (1.2341) | 0.1689 |
| **wavelet-HHH_glszm_HighGrayLevelZoneEmphasis** | 2.6223 (0.8500) | 4.3166 (10.8941) | 0.3141 |
| **wavelet-HHH_glszm_LargeAreaEmphasis** | $5.31\times{10}^{9}$ ($1.67\times{10}^{10}$) | $1.82\times{10}^{9}$ ($1.08\times{10}^{10}$) | 0.1285 |
| **wavelet-HHH_glszm_LargeAreaHighGrayLevelEmphasis** | $1.33\times{10}^{10}$ ($4.17\times{10}^{10}$) | $4.91\times{10}^{9}$ ($2.75\times{10}^{10}$) | 0.1547 |
| **wavelet-HHH_glszm_LargeAreaLowGrayLevelEmphasis** | $3.32\times{10}^{9}$ ($1.04\times{10}^{10}$) | $1.11\times{10}^{9}$ ($6.76\times{10}^{9}$) | 0.1187 |
| **wavelet-HHH_glszm_LowGrayLevelZoneEmphasis** | 0.6164 (0.0983) | 0.6037 (0.1423) | 0.9977 |
| **wavelet-HHH_glszm_SizeZoneNonUniformity** | 1.5808 (1.3103) | 4.3637 (18.4652) | 0.5694 |
| **wavelet-HHH_glszm_SizeZoneNonUniformityNormalized** | 0.4159 (0.1264) | 0.3993 (0.1047) | 0.1158 |
| **wavelet-HHH_glszm_SmallAreaEmphasis** | 0.2198 (0.2529) | 0.2536 (0.2403) | 0.6111 |
| **wavelet-HHH_glszm_SmallAreaHighGrayLevelEmphasis** | 0.5731 (0.7687) | 1.8708 (8.3835) | 0.484 |
| **wavelet-HHH_glszm_SmallAreaLowGrayLevelEmphasis** | 0.1362 (0.1744) | 0.1524 (0.1680) | 0.4471 |
| **wavelet-HHH_glszm_ZoneEntropy** | 1.5255 (0.5485) | 1.6335 (0.6828) | 0.5094 |
| **wavelet-HHH_glszm_ZonePercentage** | 0.0004 (0.0007) | 0.0010 (0.0069) | 0.8023 |
| **wavelet-HHH_glszm_ZoneVariance** | $3.12\times{10}^{9}$ ($1.22\times{10}^{10}$) | $7.21\times{10}^{8}$ ($5.30\times{10}^{9}$) | 0.0732 |
| **wavelet-HHH_ngtdm_Busyness** | $3.38\times{10}^{4}$ ($5.91\times{10}^{4}$) | $1.56\times{10}^{4}$ ($2.50\times{10}^{4}$) | 0.0039 |
| **wavelet-HHH_ngtdm_Coarseness** | 0.0006 (0.0012) | 0.0005 (0.0031) | 0.8146 |
| **wavelet-HHH_ngtdm_Complexity** | 0.5660 (0.3803) | 1.9981 (8.8090) | 0.097 |
| **wavelet-HHH_ngtdm_Contrast** | 0.1247 (0.0151) | 0.1162 (0.0321) | 0.1053 |
| **wavelet-HHH_ngtdm_Strength** | 0.0007 (0.0012) | 0.0018 (0.0103) | 0.9488 |
| **wavelet-LLL_firstorder_10Percentile** | -44.3866 (87.8071) | -5.5698 (113.6752) | 0.0023 |
| **wavelet-LLL_firstorder_90Percentile** | 106.5189 (54.8173) | 175.9101 (84.8548) | < 0.0001 |
| **wavelet-LLL_firstorder_Energy** | $1.19\times{10}^{9}$ ($1.80\times{10}^{9}$) | $1.69\times{10}^{9}$ ($2.76\times{10}^{9}$) | 0.1378 |
| **wavelet-LLL_firstorder_Entropy** | 3.1238 (0.6300) | 3.3495 (0.6720) | 0.0369 |
| **wavelet-LLL_firstorder_InterquartileRange** | 68.9920 (42.6223) | 72.7783 (50.0678) | 0.6096 |
| **wavelet-LLL_firstorder_Kurtosis** | 36.8325 (38.6664) | 38.7785 (57.9037) | 0.5665 |
| **wavelet-LLL_firstorder_Maximum** | 446.8845 (243.0156) | 654.0456 (650.0998) | 0.0043 |
| **wavelet-LLL_firstorder_MeanAbsoluteDeviation** | 53.3172 (26.3211) | 68.0502 (48.3132) | 0.0317 |
| **wavelet-LLL_firstorder_Mean** | 36.1190 (63.8909) | 91.8095 (54.4052) | < 0.0001 |
| **wavelet-LLL_firstorder_Median** | 50.2591 (67.7926) | 111.6485 (43.7557) | < 0.0001 |
| **wavelet-LLL_firstorder_Minimum** | -1038.0686 (465.1916) | -1194.1365 (519.7934) | 0.0048 |
| **wavelet-LLL_firstorder_Range** | 1484.9531 (586.0729) | 1848.1821 (864.2589) | 0.0002 |
| **wavelet-LLL_firstorder_RobustMeanAbsoluteDeviation** | 30.0863 (17.6308) | 32.9194 (24.3968) | 0.9854 |
| **wavelet-LLL_firstorder_RootMeanSquared** | 109.5485 (46.0543) | 160.3650 (73.5056) | < 0.0001 |
| **wavelet-LLL_firstorder_Skewness** | -3.3647 (2.0675) | -3.1776 (3.0270) | 0.5986 |
| **wavelet-LLL_firstorder_TotalEnergy** | $1.19\times{10}^{9}$ ($1.80\times{10}^{9}$) | $1.69\times{10}^{9}$ ($2.76\times{10}^{9}$) | 0.1377 |
| **wavelet-LLL_firstorder_Uniformity** | 0.1742 (0.0733) | 0.1564 (0.0576) | 0.0684 |
| **wavelet-LLL_firstorder_Variance** | 8760.0667 (8237.7335) | $1.97\times{10}^{4}$ ($3.40\times{10}^{4}$) | 0.0027 |
| **wavelet-LLL_glcm_Autocorrelation** | 2305.4891 (1700.0387) | 3223.6682 (3040.3093) | 0.0008 |
| **wavelet-LLL_glcm_ClusterProminence** | $6.29\times{10}^{4}$ ($1.31\times{10}^{5}$) | $7.97\times{10}^{5}$ ($5.47\times{10}^{6}$) | 0.0329 |
| **wavelet-LLL_glcm_ClusterShade** | -901.0077 (1644.4258) | -938.7714 ($2.30\times{10}^{4}$) | 0.3797 |
| **wavelet-LLL_glcm_ClusterTendency** | 39.9984 (38.0321) | 95.7519 (190.0070) | 0.0043 |
| **wavelet-LLL_glcm_Contrast** | 3.7443 (3.5133) | 7.4039 (11.8643) | 0.0123 |
| **wavelet-LLL_glcm_Correlation** | 0.8039 (0.0668) | 0.8119 (0.0925) | 0.0212 |
| **wavelet-LLL_glcm_DifferenceAverage** | 0.9962 (0.4237) | 1.2444 (0.8504) | 0.1307 |
| **wavelet-LLL_glcm_DifferenceEntropy** | 1.7740 (0.4005) | 1.9478 (0.5162) | 0.0864 |
| **wavelet-LLL_glcm_DifferenceVariance** | 2.5242 (2.6496) | 5.0255 (7.6066) | 0.0057 |
| **wavelet-LLL_glcm_Id** | 0.6702 (0.0858) | 0.6511 (0.0965) | 0.8526 |
| **wavelet-LLL_glcm_Idm** | 0.6456 (0.1012) | 0.6238 (0.1138) | 0.9062 |
| **wavelet-LLL_glcm_Idmn** | 0.9982 (0.0028) | 0.9986 (0.0021) | 0.0248 |
| **wavelet-LLL_glcm_Idn** | 0.9802 (0.0161) | 0.9828 (0.0102) | 0.0076 |
| **wavelet-LLL_glcm_Imc1** | -0.3012 (0.0557) | -0.3034 (0.0587) | 0.024 |
| **wavelet-LLL_glcm_Imc2** | 0.8920 (0.0587) | 0.9048 (0.0504) | 0.0012 |
| **wavelet-LLL_glcm_InverseVariance** | 0.4230 (0.0418) | 0.4135 (0.0476) | 0.4569 |
| **wavelet-LLL_glcm_JointAverage** | 44.1819 (18.6760) | 52.7381 (20.5346) | 0.0003 |
| **wavelet-LLL_glcm_JointEnergy** | 0.0716 (0.0470) | 0.0606 (0.0337) | 0.0623 |
| **wavelet-LLL_glcm_JointEntropy** | 5.1102 (1.0390) | 5.4783 (1.1773) | 0.0879 |
| **wavelet-LLL_glcm_MCC** | 0.8680 (0.0483) | 0.8726 (0.0536) | 0.045 |
| **wavelet-LLL_glcm_MaximumProbability** | 0.1609 (0.0862) | 0.1427 (0.0670) | 0.1449 |
| **wavelet-LLL_glcm_SumAverage** | 88.3638 (37.3520) | 105.4763 (41.0692) | 0.0003 |
| **wavelet-LLL_glcm_SumEntropy** | 3.9002 (0.6502) | 4.1311 (0.6910) | 0.0298 |
| **wavelet-LLL_glcm_SumSquares** | 10.9357 (10.2188) | 25.7889 (50.0102) | 0.0042 |
| **wavelet-LLL_gldm_DependenceEntropy** | 7.0949 (0.4456) | 7.3172 (0.4543) | < 0.0001 |
| **wavelet-LLL_gldm_DependenceNonUniformity** | 6908.7071 ($1.30\times{10}^{4}$) | 3552.3757 (5766.1021) | 0.0081 |
| **wavelet-LLL_gldm_DependenceNonUniformityNormalized** | 0.0567 (0.0167) | 0.0570 (0.0282) | 0.0435 |
| **wavelet-LLL_gldm_DependenceVariance** | 31.5297 (10.3059) | 33.0843 (11.4688) | 0.0176 |
| **wavelet-LLL_gldm_GrayLevelNonUniformity** | $3.14\times{10}^{4}$ ($6.06\times{10}^{4}$) | $1.23\times{10}^{4}$ ($2.19\times{10}^{4}$) | 0.001 |
| **wavelet-LLL_gldm_GrayLevelVariance** | 14.0947 (13.1749) | 31.6443 (54.4676) | 0.0027 |
| **wavelet-LLL_gldm_HighGrayLevelEmphasis** | 2292.4378 (1691.1662) | 3200.1640 (3026.9760) | 0.0008 |
| **wavelet-LLL_gldm_LargeDependenceEmphasis** | 164.9337 (80.8962) | 155.8135 (64.8121) | 0.9825 |
| **wavelet-LLL_gldm_LargeDependenceHighGrayLevelEmphasis** | $3.95\times{10}^{5}$ ($3.16\times{10}^{5}$) | $4.78\times{10}^{5}$ ($5.75\times{10}^{5}$) | 0.0155 |
| **wavelet-LLL_gldm_LargeDependenceLowGrayLevelEmphasis** | 0.1449 (0.1490) | 0.1247 (0.3460) | 0.0926 |
| **wavelet-LLL_gldm_LowGrayLevelEmphasis** | 0.0019 (0.0038) | 0.0011 (0.0033) | 0.0712 |
| **wavelet-LLL_gldm_SmallDependenceEmphasis** | 0.0568 (0.0374) | 0.0706 (0.0588) | 0.4495 |
| **wavelet-LLL_gldm_SmallDependenceHighGrayLevelEmphasis** | 107.0820 (128.3501) | 229.0440 (349.7217) | 0.0029 |
| **wavelet-LLL_gldm_SmallDependenceLowGrayLevelEmphasis** | 0.0004 (0.0007) | 0.0002 (0.0014) | 0.6039 |
| **wavelet-LLL_glrlm_GrayLevelNonUniformity** | $1.36\times{10}^{4}$ ($2.89\times{10}^{4}$) | 5584.8998 (9711.4743) | 0.0034 |
| **wavelet-LLL_glrlm_GrayLevelNonUniformityNormalized** | 0.1400 (0.0544) | 0.1243 (0.0436) | 0.0264 |
| **wavelet-LLL_glrlm_GrayLevelVariance** | 18.8045 (16.6762) | 41.4912 (65.0126) | 0.0006 |
| **wavelet-LLL_glrlm_HighGrayLevelRunEmphasis** | 2264.1945 (1670.8827) | 3157.3285 (3007.0692) | 0.0009 |
| **wavelet-LLL_glrlm_LongRunEmphasis** | 5.9256 (4.7735) | 5.0854 (4.2423) | 0.0738 |
| **wavelet-LLL_glrlm_LongRunHighGrayLevelEmphasis** | $1.40\times{10}^{4}$ ($1.56\times{10}^{4}$) | $1.49\times{10}^{4}$ ($1.74\times{10}^{4}$) | 0.3585 |
| **wavelet-LLL_glrlm_LongRunLowGrayLevelEmphasis** | 0.0060 (0.0071) | 0.0089 (0.0874) | 0.0444 |
| **wavelet-LLL_glrlm_LowGrayLevelRunEmphasis** | 0.0021 (0.0042) | 0.0012 (0.0038) | 0.0859 |
| **wavelet-LLL_glrlm_RunEntropy** | 4.8813 (0.3443) | 5.0713 (0.3887) | < 0.0001 |
| **wavelet-LLL_glrlm_RunLengthNonUniformity** | $2.97\times{10}^{4}$ ($5.74\times{10}^{4}$) | $1.87\times{10}^{4}$ ($2.75\times{10}^{4}$) | 0.0555 |
| **wavelet-LLL_glrlm_RunLengthNonUniformityNormalized** | 0.4899 (0.1266) | 0.5077 (0.1185) | 0.834 |
| **wavelet-LLL_glrlm_RunPercentage** | 0.6113 (0.1227) | 0.6258 (0.1072) | 0.8217 |
| **wavelet-LLL_glrlm_RunVariance** | 2.5455 (3.1924) | 2.0686 (2.7707) | 0.0733 |
| **wavelet-LLL_glrlm_ShortRunEmphasis** | 0.7135 (0.0980) | 0.7301 (0.0828) | 0.7817 |
| **wavelet-LLL_glrlm_ShortRunHighGrayLevelEmphasis** | 1587.0057 (1236.6973) | 2327.8417 (2208.2934) | 0.0007 |
| **wavelet-LLL_glrlm_ShortRunLowGrayLevelEmphasis** | 0.0018 (0.0038) | 0.0010 (0.0032) | 0.092 |
| **wavelet-LLL_glszm_GrayLevelNonUniformity** | 137.6681 (265.4626) | 86.9485 (107.7987) | 0.0131 |
| **wavelet-LLL_glszm_GrayLevelNonUniformityNormalized** | 0.0530 (0.0222) | 0.0411 (0.0197) | < 0.0001 |
| **wavelet-LLL_glszm_GrayLevelVariance** | 78.8457 (57.2902) | 146.4697 (198.6311) | < 0.0001 |
| **wavelet-LLL_glszm_HighGrayLevelZoneEmphasis** | 1849.0937 (1354.8891) | 2609.0951 (2814.5640) | 0.0015 |
| **wavelet-LLL_glszm_LargeAreaEmphasis** | $2.29\times{10}^{6}$ ($5.63\times{10}^{6}$) | $5.31\times{10}^{5}$ ($1.72\times{10}^{6}$) | 0.0006 |
| **wavelet-LLL_glszm_LargeAreaHighGrayLevelEmphasis** | $5.42\times{10}^{9}$ ($1.80\times{10}^{10}$) | $1.41\times{10}^{9}$ ($3.67\times{10}^{9}$) | 0.0103 |
| **wavelet-LLL_glszm_LargeAreaLowGrayLevelEmphasis** | 1561.2458 (4727.1289) | 616.6162 (5415.2416) | 0.0024 |
| **wavelet-LLL_glszm_LowGrayLevelZoneEmphasis** | 0.0055 (0.0094) | 0.0033 (0.0070) | 0.0226 |
| **wavelet-LLL_glszm_SizeZoneNonUniformity** | 734.3848 (918.5167) | 897.4720 (1162.0023) | 0.2671 |
| **wavelet-LLL_glszm_SizeZoneNonUniformityNormalized** | 0.3003 (0.0934) | 0.3387 (0.0798) | 0.0017 |
| **wavelet-LLL_glszm_SmallAreaEmphasis** | 0.5555 (0.0995) | 0.5973 (0.0781) | 0.0006 |
| **wavelet-LLL_glszm_SmallAreaHighGrayLevelEmphasis** | 994.2613 (793.3333) | 1526.6251 (1740.5796) | 0.0005 |
| **wavelet-LLL_glszm_SmallAreaLowGrayLevelEmphasis** | 0.0036 (0.0047) | 0.0025 (0.0044) | 0.0444 |
| **wavelet-LLL_glszm_ZoneEntropy** | 7.0019 (0.7234) | 7.2111 (0.6164) | 0.0047 |
| **wavelet-LLL_glszm_ZonePercentage** | 0.0521 (0.0403) | 0.0678 (0.0675) | 0.4325 |
| **wavelet-LLL_glszm_ZoneVariance** | $2.28\times{10}^{6}$ ($5.63\times{10}^{6}$) | $5.29\times{10}^{5}$ ($1.72\times{10}^{6}$) | 0.0006 |
| **wavelet-LLL_ngtdm_Busyness** | 2.3682 (4.8819) | 1.3052 (7.7370) | 0.0002 |
| **wavelet-LLL_ngtdm_Coarseness** | 0.0026 (0.0048) | 0.0018 (0.0105) | 0.7218 |
| **wavelet-LLL_ngtdm_Complexity** | 1153.1306 (974.3687) | 2676.3358 (4729.3714) | 0.0021 |
| **wavelet-LLL_ngtdm_Contrast** | 0.0123 (0.0172) | 0.0118 (0.0190) | 0.098 |
| **wavelet-LLL_ngtdm_Strength** | 0.7129 (0.1479) | 0.7317 (0.1319) | 0.2376 |
