# Supplementary material for: Clinical radiomics-based machine learning versus three-dimension convolutional neural network analysis for differentiation of thymic epithelial tumors from other prevascular mediastinal tumors on chest computed tomography scan
Source: Front Oncol. 2023 Apr 18;13:1105100. doi: 10.3389/fonc.2023.1105100 (PMC10151670; doi:10.3389/fonc.2023.1105100)
Supplement: Supplementary file 2 [file Table_2.docx]

**Supplementary table 2. Radiomics features extracted from CECT**

|  | **Others (n = 77)** | **TET (n = 296)** | ***p* value** |
| --- | --- | --- | --- |
|  | **Mean (SD)** | **Mean (SD)** |  |
| **original_shape_Elongation** | 0.7378 (0.1316) | 0.7387 (0.1242) | 0.9518 |
| **original_shape_Flatness** | 0.5170 (0.1200) | 0.5097 (0.1382) | 0.6746 |
| **original_shape_LeastAxisLength** | 34.6103 (22.1136) | 29.4622 (14.0697) | 0.0126 |
| **original_shape_MajorAxisLength** | 66.0564 (36.8340) | 58.9319 (26.0293) | 0.0521 |
| **original_shape_Maximum2DDiameterColumn** | 73.8858 (42.3743) | 64.5714 (28.8824) | 0.0239 |
| **original_shape_Maximum2DDiameterRow** | 71.8580 (39.3373) | 64.6026 (27.9530) | 0.0649 |
| **original_shape_Maximum2DDiameterSlice** | 63.0694 (38.1973) | 55.6167 (25.9290) | 0.0443 |
| **original_shape_Maximum3DDiameter** | 80.7231 (45.7646) | 71.5301 (31.6037) | 0.0406 |
| **original_shape_MeshVolume** | $1.48\times{10}^{5}$ ($2.41\times{10}^{5}$) | $7.24\times{10}^{4}$ ($1.08\times{10}^{5}$) | 0.0001 |
| **original_shape_MinorAxisLength** | 49.2586 (29.0524) | 43.0354 (18.9449) | 0.0236 |
| **original_shape_Sphericity** | 0.6396 (0.0750) | 0.6446 (0.0758) | 0.6025 |
| **original_shape_SurfaceArea** | $1.78\times{10}^{4}$ ($1.97\times{10}^{4}$) | $1.16\times{10}^{4}$ ($1.07\times{10}^{4}$) | 0.0003 |
| **original_shape_SurfaceVolumeRatio** | 0.2738 (0.1787) | 0.2582 (0.1203) | 0.3631 |
| **original_shape_VoxelVolume** | $1.48\times{10}^{5}$ ($2.41\times{10}^{5}$) | $7.24\times{10}^{4}$ ($1.08\times{10}^{5}$) | 0.0001 |
| **original_firstorder_10Percentile** | -8.6870 (37.2336) | 22.8605 (32.3740) | <0.0001 |
| **original_firstorder_90Percentile** | 56.9766 (27.1420) | 91.1429 (22.3530) | <0.0001 |
| **original_firstorder_Energy** | $3.42\times{10}^{8}$ ($5.95\times{10}^{8}$) | $4.12\times{10}^{8}$ ($5.99\times{10}^{8}$) | 0.3614 |
| **original_firstorder_Entropy** | 2.0940 (0.4349) | 2.1924 (0.3707) | 0.0464 |
| **original_firstorder_InterquartileRange** | 32.3669 (21.3983) | 30.2399 (10.5937) | 0.2199 |
| **original_firstorder_Kurtosis** | 22.8699 (18.2851) | 29.4943 (25.9564) | 0.0358 |
| **original_firstorder_Maximum** | 210.8182 (116.9910) | 265.0236 (225.2919) | 0.0421 |
| **original_firstorder_MeanAbsoluteDeviation** | 22.7552 (11.2948) | 24.6970 (10.4509) | 0.1541 |
| **original_firstorder_Mean** | 24.9297 (28.2816) | 58.7759 (22.0541) | <0.0001 |
| **original_firstorder_Median** | 27.9351 (29.5663) | 64.8986 (20.9250) | <0.0001 |
| **original_firstorder_Minimum** | -374.7792 (174.6990) | -415.6385 (177.1373) | 0.0714 |
| **original_firstorder_Range** | 585.5974 (230.4602) | 680.6622 (294.9357) | 0.009 |
| **original_firstorder_RobustMeanAbsoluteDeviation** | 13.9303 (8.6312) | 13.3186 (4.7626) | 0.4078 |
| **original_firstorder_RootMeanSquared** | 49.1634 (20.4949) | 74.2837 (21.3127) | <0.0001 |
| **original_firstorder_Skewness** | -2.1137 (1.7699) | -2.8062 (2.3390) | 0.0159 |
| **original_firstorder_TotalEnergy** | $3.42\times{10}^{8}$ ($5.95\times{10}^{8}$) | $4.12\times{10}^{8}$ ($5.99\times{10}^{8}$) | 0.3614 |
| **original_firstorder_Uniformity** | 0.3170 (0.0891) | 0.3045 (0.0712) | 0.1943 |
| **original_firstorder_Variance** | 1420.6766 (1533.1548) | 2031.4187 (2813.1193) | 0.0674 |
| **original_glcm_Autocorrelation** | 341.3988 (265.1080) | 456.1939 (308.2805) | 0.003 |
| **original_glcm_ClusterProminence** | 1724.6153 (5015.6839) | 8915.0410 ($6.54\times{10}^{4}$) | 0.3365 |
| **original_glcm_ClusterShade** | -49.2438 (146.6516) | -46.1507 (839.0226) | 0.9743 |
| **original_glcm_ClusterTendency** | 6.8720 (7.7704) | 9.4769 (14.9253) | 0.1399 |
| **original_glcm_Contrast** | 0.8555 (0.4350) | 1.1407 (0.9876) | 0.014 |
| **original_glcm_Correlation** | 0.6921 (0.1247) | 0.7307 (0.0842) | 0.0014 |
| **original_glcm_DifferenceAverage** | 0.5363 (0.1399) | 0.5710 (0.1783) | 0.1145 |
| **original_glcm_DifferenceEntropy** | 1.2689 (0.1951) | 1.3279 (0.2214) | 0.0335 |
| **original_glcm_DifferenceVariance** | 0.5379 (0.2910) | 0.7709 (0.7225) | 0.0059 |
| **original_glcm_Id** | 0.7660 (0.0467) | 0.7627 (0.0463) | 0.5705 |
| **original_glcm_Idm** | 0.7579 (0.0518) | 0.7536 (0.0516) | 0.5178 |
| **original_glcm_Idmn** | 0.9973 (0.0043) | 0.9982 (0.0020) | 0.0093 |
| **original_glcm_Idn** | 0.9750 (0.0166) | 0.9786 (0.0099) | 0.0167 |
| **original_glcm_Imc1** | -0.2528 (0.0790) | -0.2618 (0.0584) | 0.2678 |
| **original_glcm_Imc2** | 0.7562 (0.1218) | 0.7882 (0.0817) | 0.0065 |
| **original_glcm_InverseVariance** | 0.3961 (0.0539) | 0.3913 (0.0503) | 0.4671 |
| **original_glcm_JointAverage** | 17.0382 (7.0884) | 20.0919 (7.1131) | 0.0009 |
| **original_glcm_JointEnergy** | 0.1652 (0.0703) | 0.1573 (0.0593) | 0.3157 |
| **original_glcm_JointEntropy** | 3.4801 (0.6500) | 3.6218 (0.5943) | 0.0683 |
| **original_glcm_MCC** | 0.7835 (0.0859) | 0.7957 (0.0729) | 0.2092 |
| **original_glcm_MaximumProbability** | 0.3082 (0.1038) | 0.3001 (0.0936) | 0.5099 |
| **original_glcm_SumAverage** | 34.0764 (14.1768) | 40.1837 (14.2262) | 0.0009 |
| **original_glcm_SumEntropy** | 2.7881 (0.4935) | 2.8930 (0.4102) | 0.0566 |
| **original_glcm_SumSquares** | 1.9319 (2.0274) | 2.6544 (3.9298) | 0.1198 |
| **original_gldm_DependenceEntropy** | 6.3181 (0.4319) | 6.4510 (0.3436) | 0.0045 |
| **original_gldm_DependenceNonUniformity** | 7124.0819 ($1.26\times{10}^{4}$) | 3298.6602 (5125.7734) | 0.0001 |
| **original_gldm_DependenceNonUniformityNormalized** | 0.0465 (0.0070) | 0.0448 (0.0064) | 0.0417 |
| **original_gldm_DependenceVariance** | 39.6876 (8.2683) | 41.6547 (8.4393) | 0.0681 |
| **original_gldm_GrayLevelNonUniformity** | $5.25\times{10}^{4}$ ($9.23\times{10}^{4}$) | $2.41\times{10}^{4}$ ($4.00\times{10}^{4}$) | 0.0001 |
| **original_gldm_GrayLevelVariance** | 2.3586 (2.4557) | 3.3442 (4.5144) | 0.0658 |
| **original_gldm_HighGrayLevelEmphasis** | 340.1331 (264.1358) | 453.2488 (306.4010) | 0.0032 |
| **original_gldm_LargeDependenceEmphasis** | 247.0614 (70.6271) | 247.6814 (61.4357) | 0.9391 |
| **original_gldm_LargeDependenceHighGrayLevelEmphasis** | $8.77\times{10}^{4}$ ($8.00\times{10}^{4}$) | $1.14\times{10}^{5}$ ($8.26\times{10}^{4}$) | 0.0132 |
| **original_gldm_LargeDependenceLowGrayLevelEmphasis** | 2.0228 (3.4669) | 1.0513 (1.5762) | 0.0004 |
| **original_gldm_LowGrayLevelEmphasis** | 0.0116 (0.0235) | 0.0050 (0.0082) | 0.0001 |
| **original_gldm_SmallDependenceEmphasis** | 0.0250 (0.0118) | 0.0287 (0.0152) | 0.0488 |
| **original_gldm_SmallDependenceHighGrayLevelEmphasis** | 7.1395 (6.2856) | 11.3882 (10.6499) | 0.0009 |
| **original_gldm_SmallDependenceLowGrayLevelEmphasis** | 0.0006 (0.0012) | 0.0003 (0.0006) | 0.0037 |
| **original_glrlm_GrayLevelNonUniformity** | $1.93\times{10}^{4}$ ($3.84\times{10}^{4}$) | 8698.0353 ($1.50\times{10}^{4}$) | 0.0002 |
| **original_glrlm_GrayLevelNonUniformityNormalized** | 0.2491 (0.0610) | 0.2323 (0.0493) | 0.012 |
| **original_glrlm_GrayLevelVariance** | 3.4614 (3.4392) | 5.3189 (7.1825) | 0.0282 |
| **original_glrlm_HighGrayLevelRunEmphasis** | 335.4923 (260.1574) | 444.0174 (299.2827) | 0.0039 |
| **original_glrlm_LongRunEmphasis** | 10.6016 (6.3028) | 9.7316 (4.8605) | 0.1908 |
| **original_glrlm_LongRunHighGrayLevelEmphasis** | 3733.5917 (4300.8206) | 4428.2693 (3813.6678) | 0.1666 |
| **original_glrlm_LongRunLowGrayLevelEmphasis** | 0.0829 (0.1327) | 0.0430 (0.0811) | 0.001 |
| **original_glrlm_LowGrayLevelRunEmphasis** | 0.0128 (0.0265) | 0.0055 (0.0089) | 0.0001 |
| **original_glrlm_RunEntropy** | 4.3193 (0.3677) | 4.4585 (0.2887) | 0.0004 |
| **original_glrlm_RunLengthNonUniformity** | $2.48\times{10}^{4}$ ($4.60\times{10}^{4}$) | $1.22\times{10}^{4}$ ($1.77\times{10}^{4}$) | 0.0002 |
| **original_glrlm_RunLengthNonUniformityNormalized** | 0.3755 (0.0688) | 0.3776 (0.0632) | 0.8 |
| **original_glrlm_RunPercentage** | 0.4916 (0.0884) | 0.4918 (0.0770) | 0.9859 |
| **original_glrlm_RunVariance** | 5.1237 (3.9499) | 4.6468 (3.1231) | 0.2606 |
| **original_glrlm_ShortRunEmphasis** | 0.6226 (0.0617) | 0.6273 (0.0560) | 0.5284 |
| **original_glrlm_ShortRunHighGrayLevelEmphasis** | 204.0938 (154.8488) | 275.9284 (189.9874) | 0.0024 |
| **original_glrlm_ShortRunLowGrayLevelEmphasis** | 0.0091 (0.0197) | 0.0037 (0.0065) | 0.0001 |
| **original_glszm_GrayLevelNonUniformity** | 256.8842 (700.8434) | 99.6803 (160.9762) | 0.0005 |
| **original_glszm_GrayLevelNonUniformityNormalized** | 0.1201 (0.0642) | 0.0920 (0.0400) | <0.0001 |
| **original_glszm_GrayLevelVariance** | 15.8049 (10.9051) | 23.2909 (24.4588) | 0.0092 |
| **original_glszm_HighGrayLevelZoneEmphasis** | 270.1064 (198.3708) | 345.5103 (245.5193) | 0.0132 |
| **original_glszm_LargeAreaEmphasis** | $5.17\times{10}^{6}$ ($9.98\times{10}^{6}$) | $1.92\times{10}^{6}$ ($4.27\times{10}^{6}$) | <0.0001 |
| **original_glszm_LargeAreaHighGrayLevelEmphasis** | $1.87\times{10}^{9}$ ($3.98\times{10}^{9}$) | $8.92\times{10}^{8}$ ($2.09\times{10}^{9}$) | 0.0032 |
| **original_glszm_LargeAreaLowGrayLevelEmphasis** | $1.96\times{10}^{4}$ ($4.40\times{10}^{4}$) | 7624.8108 ($2.78\times{10}^{4}$) | 0.0034 |
| **original_glszm_LowGrayLevelZoneEmphasis** | 0.0286 (0.0631) | 0.0136 (0.0206) | 0.0006 |
| **original_glszm_SizeZoneNonUniformity** | 289.9971 (338.8618) | 258.6454 (266.8198) | 0.3872 |
| **original_glszm_SizeZoneNonUniformityNormalized** | 0.2022 (0.0615) | 0.2381 (0.0669) | <0.0001 |
| **original_glszm_SmallAreaEmphasis** | 0.4345 (0.0922) | 0.4872 (0.0823) | <0.0001 |
| **original_glszm_SmallAreaHighGrayLevelEmphasis** | 110.2597 (88.8408) | 157.0870 (124.3138) | 0.0021 |
| **original_glszm_SmallAreaLowGrayLevelEmphasis** | 0.0127 (0.0206) | 0.0084 (0.0104) | 0.0104 |
| **original_glszm_ZoneEntropy** | 6.2200 (0.7916) | 6.4141 (0.6047) | 0.0196 |
| **original_glszm_ZonePercentage** | 0.0201 (0.0133) | 0.0243 (0.0167) | 0.0412 |
| **original_glszm_ZoneVariance** | $5.16\times{10}^{6}$ ($9.97\times{10}^{6}$) | $1.91\times{10}^{6}$ ($4.27\times{10}^{6}$) | <0.0001 |
| **original_ngtdm_Busyness** | 21.4464 (37.0773) | 9.4238 (21.2459) | 0.0002 |
| **original_ngtdm_Coarseness** | 0.0018 (0.0042) | 0.0009 (0.0019) | 0.0051 |
| **original_ngtdm_Complexity** | 146.7617 (111.6330) | 229.9651 (278.1166) | 0.0106 |
| **original_ngtdm_Contrast** | 0.0067 (0.0103) | 0.0048 (0.0053) | 0.0269 |
| **original_ngtdm_Strength** | 0.5110 (0.8437) | 0.6657 (0.9300) | 0.1864 |
| **wavelet-LLH_firstorder_10Percentile** | -6.8244 (5.5699) | -9.7009 (6.9929) | 0.0009 |
| **wavelet-LLH_firstorder_90Percentile** | 3.1095 (1.5247) | 3.4488 (2.1023) | 0.185 |
| **wavelet-LLH_firstorder_Energy** | $3.58\times{10}^{6}$ ($4.80\times{10}^{6}$) | $4.05\times{10}^{6}$ ($7.71\times{10}^{6}$) | 0.6118 |
| **wavelet-LLH_firstorder_Entropy** | 1.0560 (0.1216) | 1.1423 (0.1553) | <0.0001 |
| **wavelet-LLH_firstorder_InterquartileRange** | 3.8688 (2.6263) | 4.6713 (3.2979) | 0.0487 |
| **wavelet-LLH_firstorder_Kurtosis** | 20.5842 (16.1334) | 33.0240 (144.9963) | 0.4532 |
| **wavelet-LLH_firstorder_Maximum** | 35.3641 (19.9985) | 46.8746 (63.2728) | 0.1162 |
| **wavelet-LLH_firstorder_MeanAbsoluteDeviation** | 3.5104 (1.8343) | 4.6752 (2.5290) | 0.0002 |
| **wavelet-LLH_firstorder_Mean** | -1.4495 (1.5632) | -2.2273 (1.7719) | 0.0005 |
| **wavelet-LLH_firstorder_Median** | -0.5971 (1.0089) | -0.7495 (0.9158) | 0.2037 |
| **wavelet-LLH_firstorder_Minimum** | -53.6223 (21.3787) | -65.6576 (37.7489) | 0.0076 |
| **wavelet-LLH_firstorder_Range** | 88.9864 (36.9069) | 112.5322 (87.7208) | 0.0219 |
| **wavelet-LLH_firstorder_RobustMeanAbsoluteDeviation** | 1.7560 (1.1709) | 2.2028 (1.5064) | 0.0161 |
| **wavelet-LLH_firstorder_RootMeanSquared** | 6.0028 (2.6692) | 8.0929 (3.9978) | <0.0001 |
| **wavelet-LLH_firstorder_Skewness** | -2.2759 (1.6025) | -1.8862 (3.7358) | 0.3722 |
| **wavelet-LLH_firstorder_TotalEnergy** | $3.58\times{10}^{6}$ ($4.80\times{10}^{6}$) | $4.05\times{10}^{6}$ ($7.71\times{10}^{6}$) | 0.6118 |
| **wavelet-LLH_firstorder_Uniformity** | 0.5092 (0.0483) | 0.4897 (0.0436) | 0.0007 |
| **wavelet-LLH_firstorder_Variance** | 38.5529 (33.8299) | 73.3339 (113.2853) | 0.0081 |
| **wavelet-LLH_glcm_Autocorrelation** | 10.6290 (6.4213) | 14.9218 (30.4594) | 0.2201 |
| **wavelet-LLH_glcm_ClusterProminence** | 1.6253 (1.4539) | 9.7717 (87.0477) | 0.4125 |
| **wavelet-LLH_glcm_ClusterShade** | -0.0354 (0.3332) | -0.1935 (1.0034) | 0.174 |
| **wavelet-LLH_glcm_ClusterTendency** | 0.8000 (0.1654) | 0.9516 (0.3650) | 0.0004 |
| **wavelet-LLH_glcm_Contrast** | 0.2493 (0.0496) | 0.2675 (0.1695) | 0.3525 |
| **wavelet-LLH_glcm_Correlation** | 0.5192 (0.0814) | 0.5593 (0.0781) | 0.0001 |
| **wavelet-LLH_glcm_DifferenceAverage** | 0.2485 (0.0485) | 0.2543 (0.0584) | 0.4226 |
| **wavelet-LLH_glcm_DifferenceEntropy** | 0.7944 (0.0834) | 0.8086 (0.0916) | 0.2168 |
| **wavelet-LLH_glcm_DifferenceVariance** | 0.1832 (0.0255) | 0.1965 (0.1173) | 0.3219 |
| **wavelet-LLH_glcm_Id** | 0.8759 (0.0241) | 0.8739 (0.0236) | 0.5203 |
| **wavelet-LLH_glcm_Idm** | 0.8758 (0.0242) | 0.8737 (0.0244) | 0.4942 |
| **wavelet-LLH_glcm_Idmn** | 0.9855 (0.0092) | 0.9887 (0.0065) | 0.0005 |
| **wavelet-LLH_glcm_Idn** | 0.9535 (0.0106) | 0.9576 (0.0104) | 0.0026 |
| **wavelet-LLH_glcm_Imc1** | -0.2180 (0.0624) | -0.2445 (0.0503) | 0.0001 |
| **wavelet-LLH_glcm_Imc2** | 0.5807 (0.0837) | 0.6302 (0.0722) | <0.0001 |
| **wavelet-LLH_glcm_InverseVariance** | 0.2478 (0.0477) | 0.2501 (0.0407) | 0.6762 |
| **wavelet-LLH_glcm_JointAverage** | 3.0902 (0.9768) | 3.5126 (1.5558) | 0.0239 |
| **wavelet-LLH_glcm_JointEnergy** | 0.3360 (0.0727) | 0.3183 (0.0570) | 0.0233 |
| **wavelet-LLH_glcm_JointEntropy** | 1.8499 (0.2218) | 1.9678 (0.2535) | 0.0002 |
| **wavelet-LLH_glcm_MCC** | 0.5818 (0.0707) | 0.6273 (0.0724) | <0.0001 |
| **wavelet-LLH_glcm_MaximumProbability** | 0.4478 (0.1044) | 0.4398 (0.0758) | 0.4449 |
| **wavelet-LLH_glcm_SumAverage** | 6.1805 (1.9535) | 7.0252 (3.1116) | 0.0239 |
| **wavelet-LLH_glcm_SumEntropy** | 1.5991 (0.1858) | 1.7066 (0.1979) | <0.0001 |
| **wavelet-LLH_glcm_SumSquares** | 0.2623 (0.0466) | 0.3048 (0.1251) | 0.0037 |
| **wavelet-LLH_gldm_DependenceEntropy** | 5.2712 (0.2181) | 5.3656 (0.2161) | 0.0007 |
| **wavelet-LLH_gldm_DependenceNonUniformity** | 9039.6184 ($1.43\times{10}^{4}$) | 4388.6366 (6344.3527) | <0.0001 |
| **wavelet-LLH_gldm_DependenceNonUniformityNormalized** | 0.0621 (0.0122) | 0.0611 (0.0110) | 0.4773 |
| **wavelet-LLH_gldm_DependenceVariance** | 32.8886 (4.5612) | 33.3119 (3.4354) | 0.3711 |
| **wavelet-LLH_gldm_GrayLevelNonUniformity** | $7.30\times{10}^{4}$ ($1.19\times{10}^{5}$) | $3.52\times{10}^{4}$ ($5.30\times{10}^{4}$) | <0.0001 |
| **wavelet-LLH_gldm_GrayLevelVariance** | 0.2682 (0.0467) | 0.3168 (0.1682) | 0.0127 |
| **wavelet-LLH_gldm_HighGrayLevelEmphasis** | 10.7401 (6.4102) | 15.0202 (30.5348) | 0.2225 |
| **wavelet-LLH_gldm_LargeDependenceEmphasis** | 385.2944 (38.3397) | 387.9339 (38.8905) | 0.595 |
| **wavelet-LLH_gldm_LargeDependenceHighGrayLevelEmphasis** | 4149.4612 (2547.4977) | 5416.0306 (7305.3952) | 0.1354 |
| **wavelet-LLH_gldm_LargeDependenceLowGrayLevelEmphasis** | 75.8503 (91.7996) | 50.1712 (52.5604) | 0.0015 |
| **wavelet-LLH_gldm_LowGrayLevelEmphasis** | 0.1927 (0.2189) | 0.1282 (0.1193) | 0.0006 |
| **wavelet-LLH_gldm_SmallDependenceEmphasis** | 0.0059 (0.0018) | 0.0059 (0.0024) | 0.805 |
| **wavelet-LLH_gldm_SmallDependenceHighGrayLevelEmphasis** | 0.0602 (0.0336) | 0.1403 (0.9104) | 0.4409 |
| **wavelet-LLH_gldm_SmallDependenceLowGrayLevelEmphasis** | 0.0012 (0.0014) | 0.0009 (0.0006) | 0.0012 |
| **wavelet-LLH_glrlm_GrayLevelNonUniformity** | $2.25\times{10}^{4}$ ($3.95\times{10}^{4}$) | $1.04\times{10}^{4}$ ($1.69\times{10}^{4}$) | 0.0001 |
| **wavelet-LLH_glrlm_GrayLevelNonUniformityNormalized** | 0.4788 (0.0295) | 0.4549 (0.0398) | <0.0001 |
| **wavelet-LLH_glrlm_GrayLevelVariance** | 0.3062 (0.0675) | 0.3912 (0.2925) | 0.0118 |
| **wavelet-LLH_glrlm_HighGrayLevelRunEmphasis** | 10.7671 (6.2385) | 15.0411 (30.5289) | 0.223 |
| **wavelet-LLH_glrlm_LongRunEmphasis** | 19.8028 (6.5524) | 20.1055 (4.9106) | 0.6549 |
| **wavelet-LLH_glrlm_LongRunHighGrayLevelEmphasis** | 221.4162 (172.6628) | 270.3216 (279.1549) | 0.1437 |
| **wavelet-LLH_glrlm_LongRunLowGrayLevelEmphasis** | 3.6053 (4.0899) | 2.6653 (3.0650) | 0.0266 |
| **wavelet-LLH_glrlm_LowGrayLevelRunEmphasis** | 0.1846 (0.1885) | 0.1301 (0.1086) | 0.0011 |
| **wavelet-LLH_glrlm_RunEntropy** | 3.9173 (0.2861) | 4.0559 (0.2034) | <0.0001 |
| **wavelet-LLH_glrlm_RunLengthNonUniformity** | 9134.6238 ($1.71\times{10}^{4}$) | 4351.2347 (7205.0734) | 0.0002 |
| **wavelet-LLH_glrlm_RunLengthNonUniformityNormalized** | 0.1911 (0.0268) | 0.1905 (0.0337) | 0.8761 |
| **wavelet-LLH_glrlm_RunPercentage** | 0.3175 (0.0395) | 0.3154 (0.0422) | 0.6936 |
| **wavelet-LLH_glrlm_RunVariance** | 8.0253 (3.5203) | 8.1522 (2.4335) | 0.7127 |
| **wavelet-LLH_glrlm_ShortRunEmphasis** | 0.3883 (0.0378) | 0.3884 (0.0445) | 0.9829 |
| **wavelet-LLH_glrlm_ShortRunHighGrayLevelEmphasis** | 4.1594 (2.4532) | 6.3605 (18.7305) | 0.3047 |
| **wavelet-LLH_glrlm_ShortRunLowGrayLevelEmphasis** | 0.0695 (0.0626) | 0.0520 (0.0376) | 0.002 |
| **wavelet-LLH_glszm_GrayLevelNonUniformity** | 37.7929 (39.7240) | 25.9041 (27.9978) | 0.0027 |
| **wavelet-LLH_glszm_GrayLevelNonUniformityNormalized** | 0.3744 (0.1516) | 0.3212 (0.0968) | 0.0002 |
| **wavelet-LLH_glszm_GrayLevelVariance** | 0.9643 (0.5509) | 1.7817 (3.7605) | 0.0582 |
| **wavelet-LLH_glszm_HighGrayLevelZoneEmphasis** | 10.0189 (5.1564) | 14.5803 (31.2002) | 0.2024 |
| **wavelet-LLH_glszm_LargeAreaEmphasis** | $1.14\times{10}^{8}$ ($2.83\times{10}^{8}$) | $3.82\times{10}^{7}$ ($1.02\times{10}^{8}$) | 0.0002 |
| **wavelet-LLH_glszm_LargeAreaHighGrayLevelEmphasis** | $1.47\times{10}^{9}$ ($3.57\times{10}^{9}$) | $6.12\times{10}^{8}$ ($2.01\times{10}^{9}$) | 0.0057 |
| **wavelet-LLH_glszm_LargeAreaLowGrayLevelEmphasis** | $1.10\times{10}^{7}$ ($2.80\times{10}^{7}$) | $3.37\times{10}^{6}$ ($6.77\times{10}^{6}$) | <0.0001 |
| **wavelet-LLH_glszm_LowGrayLevelZoneEmphasis** | 0.2388 (0.1252) | 0.2322 (0.1185) | 0.6665 |
| **wavelet-LLH_glszm_SizeZoneNonUniformity** | 26.7909 (33.8215) | 17.4279 (35.4502) | 0.0379 |
| **wavelet-LLH_glszm_SizeZoneNonUniformityNormalized** | 0.1916 (0.0640) | 0.1601 (0.0617) | 0.0001 |
| **wavelet-LLH_glszm_SmallAreaEmphasis** | 0.3941 (0.1270) | 0.3624 (0.1028) | 0.0226 |
| **wavelet-LLH_glszm_SmallAreaHighGrayLevelEmphasis** | 4.4442 (2.8893) | 6.2034 (16.2276) | 0.3445 |
| **wavelet-LLH_glszm_SmallAreaLowGrayLevelEmphasis** | 0.0785 (0.0561) | 0.0744 (0.0490) | 0.532 |
| **wavelet-LLH_glszm_ZoneEntropy** | 4.2002 (0.8324) | 4.5336 (0.7540) | 0.0008 |
| **wavelet-LLH_glszm_ZonePercentage** | 0.0023 (0.0025) | 0.0023 (0.0026) | 0.9922 |
| **wavelet-LLH_glszm_ZoneVariance** | $1.13\times{10}^{8}$ ($2.81\times{10}^{8}$) | $3.76\times{10}^{7}$ ($1.01\times{10}^{8}$) | 0.0002 |
| **wavelet-LLH_ngtdm_Busyness** | 1084.1379 (1854.7584) | 441.2783 (760.2063) | <0.0001 |
| **wavelet-LLH_ngtdm_Coarseness** | 0.0020 (0.0051) | 0.0009 (0.0026) | 0.0104 |
| **wavelet-LLH_ngtdm_Complexity** | 2.8940 (2.4394) | 6.6546 (30.9297) | 0.2876 |
| **wavelet-LLH_ngtdm_Contrast** | 0.0118 (0.0102) | 0.0091 (0.0068) | 0.0061 |
| **wavelet-LLH_ngtdm_Strength** | 0.0055 (0.0095) | 0.0112 (0.0427) | 0.2423 |
| **wavelet-LHL_firstorder_10Percentile** | -20.5635 (5.3574) | -20.4164 (6.2819) | 0.8507 |
| **wavelet-LHL_firstorder_90Percentile** | 18.4076 (5.5898) | 16.7620 (5.5779) | 0.0217 |
| **wavelet-LHL_firstorder_Energy** | $5.61\times{10}^{7}$ ($1.30\times{10}^{8}$) | $2.44\times{10}^{7}$ ($4.19\times{10}^{7}$) | 0.0004 |
| **wavelet-LHL_firstorder_Entropy** | 1.5611 (0.2645) | 1.5436 (0.2640) | 0.6049 |
| **wavelet-LHL_firstorder_InterquartileRange** | 19.5512 (5.3052) | 18.5317 (5.3589) | 0.137 |
| **wavelet-LHL_firstorder_Kurtosis** | 15.2284 (16.1133) | 19.8447 (21.2790) | 0.0767 |
| **wavelet-LHL_firstorder_Maximum** | 148.0237 (91.5839) | 142.7023 (93.3864) | 0.655 |
| **wavelet-LHL_firstorder_MeanAbsoluteDeviation** | 12.5978 (3.2456) | 12.3207 (3.7612) | 0.5545 |
| **wavelet-LHL_firstorder_Mean** | -1.1505 (1.5922) | -1.8008 (1.6318) | 0.0019 |
| **wavelet-LHL_firstorder_Median** | -0.8265 (1.4269) | -1.0610 (1.2622) | 0.1585 |
| **wavelet-LHL_firstorder_Minimum** | -203.4166 (106.1673) | -234.7079 (141.5879) | 0.071 |
| **wavelet-LHL_firstorder_Range** | 351.4403 (182.9224) | 377.4103 (222.5158) | 0.3457 |
| **wavelet-LHL_firstorder_RobustMeanAbsoluteDeviation** | 8.1964 (2.2035) | 7.7655 (2.2507) | 0.1337 |
| **wavelet-LHL_firstorder_RootMeanSquared** | 17.5149 (4.4606) | 17.9781 (6.5140) | 0.5563 |
| **wavelet-LHL_firstorder_Skewness** | -0.7088 (1.0701) | -1.3014 (1.3860) | 0.0005 |
| **wavelet-LHL_firstorder_TotalEnergy** | $5.61\times{10}^{7}$ ($1.30\times{10}^{8}$) | $2.44\times{10}^{7}$ ($4.19\times{10}^{7}$) | 0.0004 |
| **wavelet-LHL_firstorder_Uniformity** | 0.4007 (0.0566) | 0.4077 (0.0513) | 0.297 |
| **wavelet-LHL_firstorder_Variance** | 322.5828 (164.9904) | 359.6040 (391.9575) | 0.4186 |
| **wavelet-LHL_glcm_Autocorrelation** | 100.7240 (83.0302) | 138.7893 (184.5206) | 0.0787 |
| **wavelet-LHL_glcm_ClusterProminence** | 29.0898 (43.2368) | 134.7216 (656.9138) | 0.1597 |
| **wavelet-LHL_glcm_ClusterShade** | -1.1592 (2.4563) | -3.6883 (11.1948) | 0.0498 |
| **wavelet-LHL_glcm_ClusterTendency** | 1.3017 (0.5501) | 1.5096 (1.6081) | 0.2649 |
| **wavelet-LHL_glcm_Contrast** | 0.9635 (0.4155) | 0.9776 (0.8661) | 0.8903 |
| **wavelet-LHL_glcm_Correlation** | 0.1388 (0.1028) | 0.1863 (0.0947) | 0.0001 |
| **wavelet-LHL_glcm_DifferenceAverage** | 0.6548 (0.1435) | 0.6288 (0.1603) | 0.1962 |
| **wavelet-LHL_glcm_DifferenceEntropy** | 1.3075 (0.2002) | 1.2832 (0.2058) | 0.3543 |
| **wavelet-LHL_glcm_DifferenceVariance** | 0.4693 (0.1915) | 0.5168 (0.5537) | 0.4589 |
| **wavelet-LHL_glcm_Id** | 0.7130 (0.0393) | 0.7233 (0.0389) | 0.0385 |
| **wavelet-LHL_glcm_Idm** | 0.7010 (0.0478) | 0.7131 (0.0465) | 0.0453 |
| **wavelet-LHL_glcm_Idmn** | 0.9927 (0.0068) | 0.9946 (0.0045) | 0.0054 |
| **wavelet-LHL_glcm_Idn** | 0.9526 (0.0201) | 0.9581 (0.0158) | 0.0098 |
| **wavelet-LHL_glcm_Imc1** | -0.1012 (0.0166) | -0.0978 (0.0156) | 0.1024 |
| **wavelet-LHL_glcm_Imc2** | 0.4118 (0.0653) | 0.3969 (0.0679) | 0.086 |
| **wavelet-LHL_glcm_InverseVariance** | 0.4724 (0.0163) | 0.4651 (0.0211) | 0.005 |
| **wavelet-LHL_glcm_JointAverage** | 9.0950 (4.2610) | 10.3211 (5.6780) | 0.0777 |
| **wavelet-LHL_glcm_JointEnergy** | 0.1927 (0.0464) | 0.1989 (0.0424) | 0.2585 |
| **wavelet-LHL_glcm_JointEntropy** | 2.8869 (0.4886) | 2.8434 (0.4941) | 0.4911 |
| **wavelet-LHL_glcm_MCC** | 0.4662 (0.1027) | 0.4671 (0.1008) | 0.9501 |
| **wavelet-LHL_glcm_MaximumProbability** | 0.2618 (0.0468) | 0.2641 (0.0424) | 0.6781 |
| **wavelet-LHL_glcm_SumAverage** | 18.1900 (8.5220) | 20.6423 (11.3559) | 0.0777 |
| **wavelet-LHL_glcm_SumEntropy** | 2.0127 (0.2718) | 2.0178 (0.2749) | 0.8846 |
| **wavelet-LHL_glcm_SumSquares** | 0.5663 (0.2295) | 0.6218 (0.6134) | 0.4365 |
| **wavelet-LHL_gldm_DependenceEntropy** | 5.4956 (0.2140) | 5.5209 (0.2072) | 0.343 |
| **wavelet-LHL_gldm_DependenceNonUniformity** | 9281.4168 ($1.52\times{10}^{4}$) | 4503.6772 (6804.7062) | 0.0001 |
| **wavelet-LHL_gldm_DependenceNonUniformityNormalized** | 0.0610 (0.0047) | 0.0601 (0.0048) | 0.1227 |
| **wavelet-LHL_gldm_DependenceVariance** | 21.6672 (3.1116) | 22.4575 (3.2598) | 0.0566 |
| **wavelet-LHL_gldm_GrayLevelNonUniformity** | $5.54\times{10}^{4}$ ($8.06\times{10}^{4}$) | $2.93\times{10}^{4}$ ($4.23\times{10}^{4}$) | 0.0001 |
| **wavelet-LHL_gldm_GrayLevelVariance** | 0.6134 (0.2528) | 0.6795 (0.6217) | 0.3625 |
| **wavelet-LHL_gldm_HighGrayLevelEmphasis** | 101.1283 (82.9999) | 139.0725 (184.6603) | 0.0799 |
| **wavelet-LHL_gldm_LargeDependenceEmphasis** | 164.1289 (30.4861) | 176.2396 (31.8874) | 0.0029 |
| **wavelet-LHL_gldm_LargeDependenceHighGrayLevelEmphasis** | $1.68\times{10}^{4}$ ($1.47\times{10}^{4}$) | $2.35\times{10}^{4}$ ($2.95\times{10}^{4}$) | 0.0564 |
| **wavelet-LHL_gldm_LargeDependenceLowGrayLevelEmphasis** | 4.9274 (6.5288) | 3.6667 (4.4346) | 0.0466 |
| **wavelet-LHL_gldm_LowGrayLevelEmphasis** | 0.0323 (0.0453) | 0.0211 (0.0248) | 0.0039 |
| **wavelet-LHL_gldm_SmallDependenceEmphasis** | 0.0218 (0.0074) | 0.0213 (0.0093) | 0.678 |
| **wavelet-LHL_gldm_SmallDependenceHighGrayLevelEmphasis** | 2.1427 (1.8715) | 3.3502 (6.5358) | 0.1098 |
| **wavelet-LHL_gldm_SmallDependenceLowGrayLevelEmphasis** | 0.0010 (0.0018) | 0.0006 (0.0008) | 0.0018 |
| **wavelet-LHL_glrlm_GrayLevelNonUniformity** | $2.92\times{10}^{4}$ ($4.40\times{10}^{4}$) | $1.49\times{10}^{4}$ ($2.16\times{10}^{4}$) | 0.0001 |
| **wavelet-LHL_glrlm_GrayLevelNonUniformityNormalized** | 0.3633 (0.0602) | 0.3679 (0.0558) | 0.5233 |
| **wavelet-LHL_glrlm_GrayLevelVariance** | 0.7949 (0.3364) | 0.9186 (0.8465) | 0.2098 |
| **wavelet-LHL_glrlm_HighGrayLevelRunEmphasis** | 101.0516 (82.8044) | 138.7826 (184.4955) | 0.0813 |
| **wavelet-LHL_glrlm_LongRunEmphasis** | 7.8768 (2.7666) | 8.2736 (2.6937) | 0.2529 |
| **wavelet-LHL_glrlm_LongRunHighGrayLevelEmphasis** | 845.0666 (807.7145) | 1094.3108 (1381.3028) | 0.1303 |
| **wavelet-LHL_glrlm_LongRunLowGrayLevelEmphasis** | 0.2145 (0.2521) | 0.1712 (0.2180) | 0.1343 |
| **wavelet-LHL_glrlm_LowGrayLevelRunEmphasis** | 0.0333 (0.0471) | 0.0217 (0.0255) | 0.004 |
| **wavelet-LHL_glrlm_RunEntropy** | 3.3860 (0.1650) | 3.4547 (0.1560) | 0.0007 |
| **wavelet-LHL_glrlm_RunLengthNonUniformity** | $4.21\times{10}^{4}$ ($8.30\times{10}^{4}$) | $1.80\times{10}^{4}$ ($2.91\times{10}^{4}$) | <0.0001 |
| **wavelet-LHL_glrlm_RunLengthNonUniformityNormalized** | 0.4247 (0.0554) | 0.4060 (0.0557) | 0.0091 |
| **wavelet-LHL_glrlm_RunPercentage** | 0.5821 (0.0497) | 0.5642 (0.0505) | 0.0058 |
| **wavelet-LHL_glrlm_RunVariance** | 2.8281 (1.1849) | 3.0287 (1.1096) | 0.1644 |
| **wavelet-LHL_glrlm_ShortRunEmphasis** | 0.6451 (0.0512) | 0.6303 (0.0529) | 0.0278 |
| **wavelet-LHL_glrlm_ShortRunHighGrayLevelEmphasis** | 64.8429 (53.0775) | 89.4333 (125.0208) | 0.0927 |
| **wavelet-LHL_glrlm_ShortRunLowGrayLevelEmphasis** | 0.0225 (0.0334) | 0.0140 (0.0165) | 0.0017 |
| **wavelet-LHL_glszm_GrayLevelNonUniformity** | 612.4955 (1035.2469) | 294.1467 (539.3445) | 0.0002 |
| **wavelet-LHL_glszm_GrayLevelNonUniformityNormalized** | 0.2841 (0.0797) | 0.2587 (0.0759) | 0.0101 |
| **wavelet-LHL_glszm_GrayLevelVariance** | 4.4903 (2.3725) | 5.7510 (5.6340) | 0.0558 |
| **wavelet-LHL_glszm_HighGrayLevelZoneEmphasis** | 99.3644 (80.4078) | 131.8419 (179.4624) | 0.1227 |
| **wavelet-LHL_glszm_LargeAreaEmphasis** | $4.87\times{10}^{6}$ ($9.02\times{10}^{6}$) | $3.13\times{10}^{6}$ ($7.69\times{10}^{6}$) | 0.0897 |
| **wavelet-LHL_glszm_LargeAreaHighGrayLevelEmphasis** | $8.01\times{10}^{8}$ ($1.75\times{10}^{9}$) | $4.32\times{10}^{8}$ ($1.07\times{10}^{9}$) | 0.0201 |
| **wavelet-LHL_glszm_LargeAreaLowGrayLevelEmphasis** | $4.58\times{10}^{4}$ ($7.82\times{10}^{4}$) | $1.09\times{10}^{5}$ ($9.56\times{10}^{5}$) | 0.5634 |
| **wavelet-LHL_glszm_LowGrayLevelZoneEmphasis** | 0.0586 (0.0940) | 0.0392 (0.0513) | 0.0155 |
| **wavelet-LHL_glszm_SizeZoneNonUniformity** | 224.9850 (396.3684) | 124.3820 (167.2226) | 0.0008 |
| **wavelet-LHL_glszm_SizeZoneNonUniformityNormalized** | 0.1270 (0.0369) | 0.1391 (0.0407) | 0.0187 |
| **wavelet-LHL_glszm_SmallAreaEmphasis** | 0.2949 (0.0688) | 0.3279 (0.0746) | 0.0005 |
| **wavelet-LHL_glszm_SmallAreaHighGrayLevelEmphasis** | 28.4215 (24.0432) | 43.7652 (72.7880) | 0.0692 |
| **wavelet-LHL_glszm_SmallAreaLowGrayLevelEmphasis** | 0.0189 (0.0276) | 0.0161 (0.0215) | 0.332 |
| **wavelet-LHL_glszm_ZoneEntropy** | 5.5292 (0.8020) | 5.6607 (0.6111) | 0.1171 |
| **wavelet-LHL_glszm_ZonePercentage** | 0.0166 (0.0079) | 0.0166 (0.0099) | 0.9986 |
| **wavelet-LHL_glszm_ZoneVariance** | $4.86\times{10}^{6}$ ($9.02\times{10}^{6}$) | $3.11\times{10}^{6}$ ($7.53\times{10}^{6}$) | 0.0817 |
| **wavelet-LHL_ngtdm_Busyness** | 92.8718 (104.2696) | 65.1997 (146.4740) | 0.1202 |
| **wavelet-LHL_ngtdm_Coarseness** | 0.0010 (0.0028) | 0.0005 (0.0011) | 0.0054 |
| **wavelet-LHL_ngtdm_Complexity** | 80.7856 (75.0549) | 102.3848 (170.0341) | 0.2779 |
| **wavelet-LHL_ngtdm_Contrast** | 0.0062 (0.0061) | 0.0048 (0.0043) | 0.0154 |
| **wavelet-LHL_ngtdm_Strength** | 0.0575 (0.1099) | 0.0757 (0.1669) | 0.3627 |
| **wavelet-LHH_firstorder_10Percentile** | -1.2294 (0.5095) | -1.2977 (0.9522) | 0.5446 |
| **wavelet-LHH_firstorder_90Percentile** | 1.2638 (0.5381) | 1.2859 (0.9684) | 0.8481 |
| **wavelet-LHH_firstorder_Energy** | $2.23\times{10}^{5}$ ($4.72\times{10}^{5}$) | $1.39\times{10}^{5}$ ($5.12\times{10}^{5}$) | 0.1914 |
| **wavelet-LHH_firstorder_Entropy** | 0.9985 (0.0056) | 1.0010 (0.0126) | 0.0907 |
| **wavelet-LHH_firstorder_InterquartileRange** | 1.2097 (0.5046) | 1.2409 (0.9544) | 0.7818 |
| **wavelet-LHH_firstorder_Kurtosis** | 11.9361 (10.5812) | 20.4285 (65.8918) | 0.2609 |
| **wavelet-LHH_firstorder_Maximum** | 12.2622 (6.8654) | 13.9359 (10.1896) | 0.1739 |
| **wavelet-LHH_firstorder_MeanAbsoluteDeviation** | 0.8107 (0.3285) | 0.8539 (0.6142) | 0.5521 |
| **wavelet-LHH_firstorder_Mean** | 0.0199 (0.0509) | -0.0010 (0.0514) | 0.0015 |
| **wavelet-LHH_firstorder_Median** | 0.0139 (0.0480) | 0.0007 (0.0273) | 0.0016 |
| **wavelet-LHH_firstorder_Minimum** | -11.1562 (6.6396) | -12.6860 (10.1014) | 0.2087 |
| **wavelet-LHH_firstorder_Range** | 23.4184 (12.7666) | 26.6219 (19.7105) | 0.1767 |
| **wavelet-LHH_firstorder_RobustMeanAbsoluteDeviation** | 0.5104 (0.2126) | 0.5244 (0.3995) | 0.7674 |
| **wavelet-LHH_firstorder_RootMeanSquared** | 1.1386 (0.4471) | 1.2363 (0.8478) | 0.3302 |
| **wavelet-LHH_firstorder_Skewness** | 0.1689 (0.5001) | 0.1631 (1.2230) | 0.9674 |
| **wavelet-LHH_firstorder_TotalEnergy** | $2.23\times{10}^{5}$ ($4.72\times{10}^{5}$) | $1.39\times{10}^{5}$ ($5.12\times{10}^{5}$) | 0.1914 |
| **wavelet-LHH_firstorder_Uniformity** | 0.5011 (0.0038) | 0.5001 (0.0018) | 0.001 |
| **wavelet-LHH_firstorder_Variance** | 1.4909 (1.5582) | 2.2421 (7.7384) | 0.3978 |
| **wavelet-LHH_glcm_Autocorrelation** | 2.4370 (0.7799) | 2.6506 (2.2232) | 0.4075 |
| **wavelet-LHH_glcm_ClusterProminence** | 0.5306 (0.0278) | 0.5471 (0.0465) | 0.0031 |
| **wavelet-LHH_glcm_ClusterShade** | -0.0079 (0.0358) | 0.0026 (0.0162) | 0.0002 |
| **wavelet-LHH_glcm_ClusterTendency** | 0.5293 (0.0253) | 0.5433 (0.0253) | <0.0001 |
| **wavelet-LHH_glcm_Contrast** | 0.4682 (0.0301) | 0.4574 (0.0273) | 0.0025 |
| **wavelet-LHH_glcm_Correlation** | 0.0615 (0.0558) | 0.0860 (0.0513) | 0.0003 |
| **wavelet-LHH_glcm_DifferenceAverage** | 0.4682 (0.0301) | 0.4571 (0.0264) | 0.0015 |
| **wavelet-LHH_glcm_DifferenceEntropy** | 0.9612 (0.0130) | 0.9645 (0.0145) | 0.0644 |
| **wavelet-LHH_glcm_DifferenceVariance** | 0.2369 (0.0043) | 0.2380 (0.0049) | 0.0634 |
| **wavelet-LHH_glcm_Id** | 0.7659 (0.0151) | 0.7715 (0.0131) | 0.0013 |
| **wavelet-LHH_glcm_Idm** | 0.7659 (0.0151) | 0.7715 (0.0131) | 0.0014 |
| **wavelet-LHH_glcm_Idmn** | 0.9091 (0.0128) | 0.9140 (0.0172) | 0.0202 |
| **wavelet-LHH_glcm_Idn** | 0.8463 (0.0137) | 0.8528 (0.0176) | 0.0031 |
| **wavelet-LHH_glcm_Imc1** | -0.0385 (0.0122) | -0.0363 (0.0106) | 0.1207 |
| **wavelet-LHH_glcm_Imc2** | 0.2033 (0.0466) | 0.1890 (0.0363) | 0.0041 |
| **wavelet-LHH_glcm_InverseVariance** | 0.4682 (0.0301) | 0.4569 (0.0260) | 0.0011 |
| **wavelet-LHH_glcm_JointAverage** | 1.5441 (0.1953) | 1.5756 (0.3836) | 0.4859 |
| **wavelet-LHH_glcm_JointEnergy** | 0.2643 (0.0077) | 0.2624 (0.0040) | 0.0023 |
| **wavelet-LHH_glcm_JointEntropy** | 1.9581 (0.0210) | 1.9651 (0.0240) | 0.0202 |
| **wavelet-LHH_glcm_MCC** | 0.1755 (0.0405) | 0.1645 (0.0338) | 0.0155 |
| **wavelet-LHH_glcm_MaximumProbability** | 0.3035 (0.0255) | 0.2959 (0.0117) | 0.0002 |
| **wavelet-LHH_glcm_SumAverage** | 3.0881 (0.3906) | 3.1512 (0.7673) | 0.4859 |
| **wavelet-LHH_glcm_SumEntropy** | 1.4898 (0.0284) | 1.5074 (0.0265) | <0.0001 |
| **wavelet-LHH_glcm_SumSquares** | 0.2494 (0.0024) | 0.2502 (0.0031) | 0.0417 |
| **wavelet-LHH_gldm_DependenceEntropy** | 4.9327 (0.1548) | 4.9925 (0.1448) | 0.0016 |
| **wavelet-LHH_gldm_DependenceNonUniformity** | $1.27\times{10}^{4}$ ($2.16\times{10}^{4}$) | 5889.5399 (9446.3540) | <0.0001 |
| **wavelet-LHH_gldm_DependenceNonUniformityNormalized** | 0.0767 (0.0101) | 0.0741 (0.0088) | 0.0232 |
| **wavelet-LHH_gldm_DependenceVariance** | 14.5960 (4.0193) | 15.5153 (3.3917) | 0.0425 |
| **wavelet-LHH_gldm_GrayLevelNonUniformity** | $7.40\times{10}^{4}$ ($1.20\times{10}^{5}$) | $3.62\times{10}^{4}$ ($5.39\times{10}^{4}$) | 0.0001 |
| **wavelet-LHH_gldm_GrayLevelVariance** | 0.2495 (0.0019) | 0.2503 (0.0038) | 0.0828 |
| **wavelet-LHH_gldm_HighGrayLevelEmphasis** | 2.6742 (0.7789) | 2.8847 (2.2290) | 0.4156 |
| **wavelet-LHH_gldm_LargeDependenceEmphasis** | 198.7306 (16.2755) | 209.2897 (15.5730) | <0.0001 |
| **wavelet-LHH_gldm_LargeDependenceHighGrayLevelEmphasis** | 533.0221 (167.2173) | 590.7859 (389.7875) | 0.2051 |
| **wavelet-LHH_gldm_LargeDependenceLowGrayLevelEmphasis** | 119.5451 (21.3862) | 127.0423 (25.3332) | 0.0176 |
| **wavelet-LHH_gldm_LowGrayLevelEmphasis** | 0.6031 (0.0873) | 0.5998 (0.1083) | 0.8035 |
| **wavelet-LHH_gldm_SmallDependenceEmphasis** | 0.0083 (0.0019) | 0.0079 (0.0015) | 0.0765 |
| **wavelet-LHH_gldm_SmallDependenceHighGrayLevelEmphasis** | 0.0214 (0.0064) | 0.0243 (0.0407) | 0.5289 |
| **wavelet-LHH_gldm_SmallDependenceLowGrayLevelEmphasis** | 0.0052 (0.0017) | 0.0048 (0.0012) | 0.0152 |
| **wavelet-LHH_glrlm_GrayLevelNonUniformity** | $3.77\times{10}^{4}$ ($6.16\times{10}^{4}$) | $1.81\times{10}^{4}$ ($2.71\times{10}^{4}$) | <0.0001 |
| **wavelet-LHH_glrlm_GrayLevelNonUniformityNormalized** | 0.5002 (0.0005) | 0.4998 (0.0029) | 0.2851 |
| **wavelet-LHH_glrlm_GrayLevelVariance** | 0.2499 (0.0003) | 0.2506 (0.0067) | 0.3747 |
| **wavelet-LHH_glrlm_HighGrayLevelRunEmphasis** | 2.6695 (0.7776) | 2.8959 (2.2299) | 0.3813 |
| **wavelet-LHH_glrlm_LongRunEmphasis** | 6.5999 (0.8215) | 6.8932 (0.6910) | 0.0016 |
| **wavelet-LHH_glrlm_LongRunHighGrayLevelEmphasis** | 17.6523 (5.7203) | 19.4538 (12.1796) | 0.208 |
| **wavelet-LHH_glrlm_LongRunLowGrayLevelEmphasis** | 3.9836 (0.8083) | 4.1734 (0.8647) | 0.0829 |
| **wavelet-LHH_glrlm_LowGrayLevelRunEmphasis** | 0.6043 (0.0861) | 0.5971 (0.1074) | 0.5853 |
| **wavelet-LHH_glrlm_RunEntropy** | 2.9344 (0.0957) | 2.9910 (0.0781) | <0.0001 |
| **wavelet-LHH_glrlm_RunLengthNonUniformity** | $2.76\times{10}^{4}$ ($4.64\times{10}^{4}$) | $1.28\times{10}^{4}$ ($1.95\times{10}^{4}$) | <0.0001 |
| **wavelet-LHH_glrlm_RunLengthNonUniformityNormalized** | 0.3493 (0.0204) | 0.3368 (0.0186) | <0.0001 |
| **wavelet-LHH_glrlm_RunPercentage** | 0.5170 (0.0225) | 0.5034 (0.0201) | <0.0001 |
| **wavelet-LHH_glrlm_RunVariance** | 2.0688 (0.3255) | 2.1973 (0.2669) | 0.0004 |
| **wavelet-LHH_glrlm_ShortRunEmphasis** | 0.5793 (0.0220) | 0.5672 (0.0202) | <0.0001 |
| **wavelet-LHH_glrlm_ShortRunHighGrayLevelEmphasis** | 1.5381 (0.4308) | 1.6535 (1.3688) | 0.466 |
| **wavelet-LHH_glrlm_ShortRunLowGrayLevelEmphasis** | 0.3517 (0.0527) | 0.3379 (0.0619) | 0.0737 |
| **wavelet-LHH_glszm_GrayLevelNonUniformity** | 13.2037 (12.3799) | 11.6819 (26.1197) | 0.6198 |
| **wavelet-LHH_glszm_GrayLevelNonUniformityNormalized** | 0.5536 (0.0722) | 0.5456 (0.0814) | 0.4327 |
| **wavelet-LHH_glszm_GrayLevelVariance** | 0.2314 (0.0811) | 0.2646 (0.2504) | 0.2527 |
| **wavelet-LHH_glszm_HighGrayLevelZoneEmphasis** | 2.4491 (1.0149) | 2.7468 (2.4584) | 0.2995 |
| **wavelet-LHH_glszm_LargeAreaEmphasis** | $6.19\times{10}^{8}$ ($1.58\times{10}^{9}$) | $2.18\times{10}^{8}$ ($7.72\times{10}^{8}$) | 0.0017 |
| **wavelet-LHH_glszm_LargeAreaHighGrayLevelEmphasis** | $1.57\times{10}^{9}$ ($3.93\times{10}^{9}$) | $7.42\times{10}^{8}$ ($4.57\times{10}^{9}$) | 0.1437 |
| **wavelet-LHH_glszm_LargeAreaLowGrayLevelEmphasis** | $3.85\times{10}^{8}$ ($9.93\times{10}^{8}$) | $1.16\times{10}^{8}$ ($2.58\times{10}^{8}$) | <0.0001 |
| **wavelet-LHH_glszm_LowGrayLevelZoneEmphasis** | 0.6633 (0.1456) | 0.6438 (0.1551) | 0.3205 |
| **wavelet-LHH_glszm_SizeZoneNonUniformity** | 9.3940 (9.9576) | 8.9031 (29.8524) | 0.8871 |
| **wavelet-LHH_glszm_SizeZoneNonUniformityNormalized** | 0.3519 (0.0834) | 0.3599 (0.1088) | 0.5485 |
| **wavelet-LHH_glszm_SmallAreaEmphasis** | 0.5254 (0.1764) | 0.5618 (0.1435) | 0.0601 |
| **wavelet-LHH_glszm_SmallAreaHighGrayLevelEmphasis** | 1.3115 (0.7961) | 1.5442 (1.6704) | 0.2361 |
| **wavelet-LHH_glszm_SmallAreaLowGrayLevelEmphasis** | 0.3442 (0.1417) | 0.3639 (0.1358) | 0.2618 |
| **wavelet-LHH_glszm_ZoneEntropy** | 2.4652 (0.5225) | 2.5032 (0.4612) | 0.5316 |
| **wavelet-LHH_glszm_ZonePercentage** | 0.0008 (0.0013) | 0.0007 (0.0011) | 0.4724 |
| **wavelet-LHH_glszm_ZoneVariance** | $5.94\times{10}^{8}$ ($1.54\times{10}^{9}$) | $2.02\times{10}^{8}$ ($7.41\times{10}^{8}$) | 0.0016 |
| **wavelet-LHH_ngtdm_Busyness** | $3.47\times{10}^{4}$ ($6.07\times{10}^{4}$) | $1.45\times{10}^{4}$ ($2.06\times{10}^{4}$) | <0.0001 |
| **wavelet-LHH_ngtdm_Coarseness** | 0.0008 (0.0020) | 0.0004 (0.0008) | 0.0031 |
| **wavelet-LHH_ngtdm_Complexity** | 0.5581 (0.4383) | 0.7767 (1.8354) | 0.3006 |
| **wavelet-LHH_ngtdm_Contrast** | 0.1114 (0.0199) | 0.1048 (0.0256) | 0.0373 |
| **wavelet-LHH_ngtdm_Strength** | 0.0008 (0.0020) | 0.0004 (0.0010) | 0.0315 |
| **wavelet-HLL_firstorder_10Percentile** | -24.6625 (12.8521) | -27.7225 (21.3741) | 0.2308 |
| **wavelet-HLL_firstorder_90Percentile** | 16.8905 (4.7102) | 17.1206 (4.9575) | 0.7142 |
| **wavelet-HLL_firstorder_Energy** | $5.28\times{10}^{7}$ ($7.16\times{10}^{7}$) | $4.09\times{10}^{7}$ ($5.48\times{10}^{7}$) | 0.112 |
| **wavelet-HLL_firstorder_Entropy** | 1.6534 (0.3344) | 1.7219 (0.3599) | 0.1321 |
| **wavelet-HLL_firstorder_InterquartileRange** | 19.7056 (6.0709) | 20.6481 (9.3125) | 0.4002 |
| **wavelet-HLL_firstorder_Kurtosis** | 25.7052 (25.1238) | 25.0002 (22.0918) | 0.8087 |
| **wavelet-HLL_firstorder_Maximum** | 172.8815 (110.4460) | 181.4793 (118.9183) | 0.5668 |
| **wavelet-HLL_firstorder_MeanAbsoluteDeviation** | 14.6318 (5.6603) | 16.0462 (8.0258) | 0.1467 |
| **wavelet-HLL_firstorder_Mean** | -3.9494 (4.9116) | -4.8373 (6.7799) | 0.2819 |
| **wavelet-HLL_firstorder_Median** | -1.5393 (2.2060) | -1.6613 (2.3922) | 0.6859 |
| **wavelet-HLL_firstorder_Minimum** | -276.4601 (147.0853) | -299.3430 (144.5370) | 0.2183 |
| **wavelet-HLL_firstorder_Range** | 449.3416 (238.3112) | 480.8223 (244.6136) | 0.3125 |
| **wavelet-HLL_firstorder_RobustMeanAbsoluteDeviation** | 8.3735 (2.6339) | 8.8688 (4.2478) | 0.3302 |
| **wavelet-HLL_firstorder_RootMeanSquared** | 23.6807 (11.4155) | 26.4595 (14.5691) | 0.1211 |
| **wavelet-HLL_firstorder_Skewness** | -2.1068 (1.9686) | -2.3349 (1.8796) | 0.3481 |
| **wavelet-HLL_firstorder_TotalEnergy** | $5.28\times{10}^{7}$ ($7.16\times{10}^{7}$) | $4.09\times{10}^{7}$ ($5.48\times{10}^{7}$) | 0.112 |
| **wavelet-HLL_firstorder_Uniformity** | 0.3910 (0.0614) | 0.3818 (0.0603) | 0.234 |
| **wavelet-HLL_firstorder_Variance** | 649.9862 (654.6256) | 842.4367 (1100.6486) | 0.1431 |
| **wavelet-HLL_glcm_Autocorrelation** | 175.7593 (161.1357) | 198.2562 (171.4728) | 0.2999 |
| **wavelet-HLL_glcm_ClusterProminence** | 337.8643 (666.7548) | 576.3296 (1262.6098) | 0.1106 |
| **wavelet-HLL_glcm_ClusterShade** | -18.6911 (34.7492) | -27.1323 (49.9875) | 0.1636 |
| **wavelet-HLL_glcm_ClusterTendency** | 2.6954 (2.6846) | 3.6013 (4.9697) | 0.1241 |
| **wavelet-HLL_glcm_Contrast** | 1.2129 (0.7257) | 1.4526 (1.4631) | 0.1646 |
| **wavelet-HLL_glcm_Correlation** | 0.2601 (0.1840) | 0.3010 (0.1662) | 0.0609 |
| **wavelet-HLL_glcm_DifferenceAverage** | 0.6837 (0.1552) | 0.7113 (0.2169) | 0.2952 |
| **wavelet-HLL_glcm_DifferenceEntropy** | 1.3535 (0.2265) | 1.3954 (0.2568) | 0.1934 |
| **wavelet-HLL_glcm_DifferenceVariance** | 0.6707 (0.4899) | 0.8410 (0.9186) | 0.1177 |
| **wavelet-HLL_glcm_Id** | 0.7107 (0.0365) | 0.7081 (0.0408) | 0.6068 |
| **wavelet-HLL_glcm_Idm** | 0.6985 (0.0445) | 0.6949 (0.0494) | 0.5634 |
| **wavelet-HLL_glcm_Idmn** | 0.9939 (0.0065) | 0.9949 (0.0070) | 0.2842 |
| **wavelet-HLL_glcm_Idn** | 0.9586 (0.0216) | 0.9617 (0.0182) | 0.2 |
| **wavelet-HLL_glcm_Imc1** | -0.1215 (0.0334) | -0.1247 (0.0351) | 0.4756 |
| **wavelet-HLL_glcm_Imc2** | 0.4738 (0.1093) | 0.4853 (0.1150) | 0.4322 |
| **wavelet-HLL_glcm_InverseVariance** | 0.4703 (0.0178) | 0.4650 (0.0198) | 0.0312 |
| **wavelet-HLL_glcm_JointAverage** | 11.8962 (5.8578) | 12.8443 (5.7317) | 0.1988 |
| **wavelet-HLL_glcm_JointEnergy** | 0.1897 (0.0462) | 0.1840 (0.0444) | 0.3188 |
| **wavelet-HLL_glcm_JointEntropy** | 3.0008 (0.5570) | 3.1117 (0.6157) | 0.152 |
| **wavelet-HLL_glcm_MCC** | 0.5380 (0.1303) | 0.5479 (0.1371) | 0.5695 |
| **wavelet-HLL_glcm_MaximumProbability** | 0.2587 (0.0381) | 0.2524 (0.0396) | 0.2093 |
| **wavelet-HLL_glcm_SumAverage** | 23.7925 (11.7155) | 25.6887 (11.4635) | 0.1988 |
| **wavelet-HLL_glcm_SumEntropy** | 2.1192 (0.3571) | 2.2009 (0.3874) | 0.0948 |
| **wavelet-HLL_glcm_SumSquares** | 0.9771 (0.8301) | 1.2635 (1.5939) | 0.1286 |
| **wavelet-HLL_gldm_DependenceEntropy** | 5.5759 (0.3025) | 5.6757 (0.2966) | 0.0091 |
| **wavelet-HLL_gldm_DependenceNonUniformity** | 9588.6620 ($1.61\times{10}^{4}$) | 4502.5129 (7087.7253) | <0.0001 |
| **wavelet-HLL_gldm_DependenceNonUniformityNormalized** | 0.0615 (0.0061) | 0.0591 (0.0052) | 0.0006 |
| **wavelet-HLL_gldm_DependenceVariance** | 21.9936 (3.6614) | 23.2883 (3.5691) | 0.0051 |
| **wavelet-HLL_gldm_GrayLevelNonUniformity** | $6.23\times{10}^{4}$ ($1.02\times{10}^{5}$) | $2.91\times{10}^{4}$ ($4.53\times{10}^{4}$) | <0.0001 |
| **wavelet-HLL_gldm_GrayLevelVariance** | 1.1422 (1.0414) | 1.4517 (1.7617) | 0.141 |
| **wavelet-HLL_gldm_HighGrayLevelEmphasis** | 175.8603 (160.8621) | 198.3987 (171.4317) | 0.2988 |
| **wavelet-HLL_gldm_LargeDependenceEmphasis** | 165.6236 (34.8828) | 166.7198 (31.6878) | 0.7913 |
| **wavelet-HLL_gldm_LargeDependenceHighGrayLevelEmphasis** | $2.97\times{10}^{4}$ ($2.76\times{10}^{4}$) | $3.24\times{10}^{4}$ ($2.58\times{10}^{4}$) | 0.4153 |
| **wavelet-HLL_gldm_LargeDependenceLowGrayLevelEmphasis** | 3.0023 (4.2097) | 2.6122 (7.3731) | 0.6563 |
| **wavelet-HLL_gldm_LowGrayLevelEmphasis** | 0.0200 (0.0263) | 0.0162 (0.0402) | 0.4392 |
| **wavelet-HLL_gldm_SmallDependenceEmphasis** | 0.0252 (0.0129) | 0.0270 (0.0156) | 0.3419 |
| **wavelet-HLL_gldm_SmallDependenceHighGrayLevelEmphasis** | 4.1269 (4.2919) | 5.4292 (6.8619) | 0.1137 |
| **wavelet-HLL_gldm_SmallDependenceLowGrayLevelEmphasis** | 0.0007 (0.0009) | 0.0006 (0.0008) | 0.1276 |
| **wavelet-HLL_glrlm_GrayLevelNonUniformity** | $3.10\times{10}^{4}$ ($5.09\times{10}^{4}$) | $1.46\times{10}^{4}$ ($2.25\times{10}^{4}$) | <0.0001 |
| **wavelet-HLL_glrlm_GrayLevelNonUniformityNormalized** | 0.3506 (0.0675) | 0.3373 (0.0651) | 0.1153 |
| **wavelet-HLL_glrlm_GrayLevelVariance** | 1.6108 (1.4995) | 2.0525 (2.2661) | 0.1061 |
| **wavelet-HLL_glrlm_HighGrayLevelRunEmphasis** | 174.3682 (159.8393) | 196.4083 (170.5119) | 0.3069 |
| **wavelet-HLL_glrlm_LongRunEmphasis** | 8.0511 (3.0868) | 7.7052 (2.7787) | 0.3424 |
| **wavelet-HLL_glrlm_LongRunHighGrayLevelEmphasis** | 1482.2813 (1734.1765) | 1459.7659 (1183.9168) | 0.8936 |
| **wavelet-HLL_glrlm_LongRunLowGrayLevelEmphasis** | 0.1491 (0.2207) | 0.1188 (0.2548) | 0.3405 |
| **wavelet-HLL_glrlm_LowGrayLevelRunEmphasis** | 0.0207 (0.0270) | 0.0167 (0.0402) | 0.4126 |
| **wavelet-HLL_glrlm_RunEntropy** | 3.5118 (0.2244) | 3.5997 (0.2483) | 0.005 |
| **wavelet-HLL_glrlm_RunLengthNonUniformity** | $3.46\times{10}^{4}$ ($5.78\times{10}^{4}$) | $1.80\times{10}^{4}$ ($2.62\times{10}^{4}$) | 0.0003 |
| **wavelet-HLL_glrlm_RunLengthNonUniformityNormalized** | 0.4267 (0.0654) | 0.4265 (0.0609) | 0.9837 |
| **wavelet-HLL_glrlm_RunPercentage** | 0.5812 (0.0587) | 0.5809 (0.0533) | 0.9665 |
| **wavelet-HLL_glrlm_RunVariance** | 2.9908 (1.3316) | 2.8976 (1.1783) | 0.5478 |
| **wavelet-HLL_glrlm_ShortRunEmphasis** | 0.6481 (0.0609) | 0.6511 (0.0565) | 0.6797 |
| **wavelet-HLL_glrlm_ShortRunHighGrayLevelEmphasis** | 112.2034 (104.5397) | 128.9959 (118.8813) | 0.2589 |
| **wavelet-HLL_glrlm_ShortRunLowGrayLevelEmphasis** | 0.0140 (0.0180) | 0.0110 (0.0243) | 0.3241 |
| **wavelet-HLL_glszm_GrayLevelNonUniformity** | 524.3295 (1191.3422) | 251.1042 (485.8639) | 0.0022 |
| **wavelet-HLL_glszm_GrayLevelNonUniformityNormalized** | 0.2192 (0.1062) | 0.1958 (0.0919) | 0.0555 |
| **wavelet-HLL_glszm_GrayLevelVariance** | 8.4823 (6.1014) | 10.0886 (7.4729) | 0.0826 |
| **wavelet-HLL_glszm_HighGrayLevelZoneEmphasis** | 153.8498 (148.8093) | 173.0326 (159.6905) | 0.3417 |
| **wavelet-HLL_glszm_LargeAreaEmphasis** | $6.83\times{10}^{6}$ ($1.39\times{10}^{7}$) | $3.22\times{10}^{6}$ ($9.61\times{10}^{6}$) | 0.0082 |
| **wavelet-HLL_glszm_LargeAreaHighGrayLevelEmphasis** | $1.46\times{10}^{9}$ ($3.06\times{10}^{9}$) | $5.28\times{10}^{8}$ ($1.43\times{10}^{9}$) | 0.0001 |
| **wavelet-HLL_glszm_LargeAreaLowGrayLevelEmphasis** | $5.62\times{10}^{4}$ ($9.88\times{10}^{4}$) | $2.95\times{10}^{5}$ ($4.45\times{10}^{6}$) | 0.6388 |
| **wavelet-HLL_glszm_LowGrayLevelZoneEmphasis** | 0.0384 (0.0507) | 0.0285 (0.0427) | 0.0812 |
| **wavelet-HLL_glszm_SizeZoneNonUniformity** | 241.6230 (313.0370) | 175.9468 (210.3738) | 0.0296 |
| **wavelet-HLL_glszm_SizeZoneNonUniformityNormalized** | 0.1543 (0.0519) | 0.1615 (0.0527) | 0.285 |
| **wavelet-HLL_glszm_SmallAreaEmphasis** | 0.3484 (0.0974) | 0.3704 (0.0881) | 0.0566 |
| **wavelet-HLL_glszm_SmallAreaHighGrayLevelEmphasis** | 54.3580 (55.4808) | 63.9065 (64.5419) | 0.2353 |
| **wavelet-HLL_glszm_SmallAreaLowGrayLevelEmphasis** | 0.0128 (0.0156) | 0.0121 (0.0182) | 0.7679 |
| **wavelet-HLL_glszm_ZoneEntropy** | 5.8021 (0.6747) | 6.0174 (0.6418) | 0.0099 |
| **wavelet-HLL_glszm_ZonePercentage** | 0.0209 (0.0145) | 0.0228 (0.0176) | 0.385 |
| **wavelet-HLL_glszm_ZoneVariance** | $6.82\times{10}^{6}$ ($1.39\times{10}^{7}$) | $3.18\times{10}^{6}$ ($9.27\times{10}^{6}$) | 0.0064 |
| **wavelet-HLL_ngtdm_Busyness** | 81.3077 (112.7598) | 112.4261 (1190.8919) | 0.8192 |
| **wavelet-HLL_ngtdm_Coarseness** | 0.0010 (0.0026) | 0.0005 (0.0010) | 0.0028 |
| **wavelet-HLL_ngtdm_Complexity** | 132.8020 (127.6774) | 165.7273 (185.8042) | 0.1433 |
| **wavelet-HLL_ngtdm_Contrast** | 0.0069 (0.0071) | 0.0064 (0.0096) | 0.6533 |
| **wavelet-HLL_ngtdm_Strength** | 0.1813 (0.5882) | 0.1449 (0.2506) | 0.4133 |
| **wavelet-HLH_firstorder_10Percentile** | -1.2848 (0.4896) | -1.4269 (0.8036) | 0.1396 |
| **wavelet-HLH_firstorder_90Percentile** | 1.3113 (0.5753) | 1.4472 (0.9291) | 0.2223 |
| **wavelet-HLH_firstorder_Energy** | $1.94\times{10}^{5}$ ($2.89\times{10}^{5}$) | $1.54\times{10}^{5}$ ($3.65\times{10}^{5}$) | 0.3719 |
| **wavelet-HLH_firstorder_Entropy** | 1.0000 (0.0041) | 1.0005 (0.0068) | 0.5253 |
| **wavelet-HLH_firstorder_InterquartileRange** | 1.2198 (0.4776) | 1.3164 (0.8015) | 0.3122 |
| **wavelet-HLH_firstorder_Kurtosis** | 15.9687 (11.3553) | 37.7625 (230.5069) | 0.4079 |
| **wavelet-HLH_firstorder_Maximum** | 15.4456 (9.1107) | 18.5727 (12.8161) | 0.0449 |
| **wavelet-HLH_firstorder_MeanAbsoluteDeviation** | 0.8825 (0.3575) | 0.9850 (0.5476) | 0.1202 |
| **wavelet-HLH_firstorder_Mean** | 0.0276 (0.0894) | 0.0261 (0.1269) | 0.9226 |
| **wavelet-HLH_firstorder_Median** | 0.0012 (0.0348) | -0.0014 (0.0410) | 0.6131 |
| **wavelet-HLH_firstorder_Minimum** | -12.4617 (7.1698) | -14.1928 (9.9448) | 0.1527 |
| **wavelet-HLH_firstorder_Range** | 27.9072 (15.6334) | 32.7654 (22.0535) | 0.07 |
| **wavelet-HLH_firstorder_RobustMeanAbsoluteDeviation** | 0.5178 (0.2031) | 0.5625 (0.3391) | 0.2689 |
| **wavelet-HLH_firstorder_RootMeanSquared** | 1.3427 (0.5996) | 1.5308 (0.8036) | 0.0558 |
| **wavelet-HLH_firstorder_Skewness** | 0.6050 (0.9818) | 0.9644 (2.7413) | 0.2589 |
| **wavelet-HLH_firstorder_TotalEnergy** | $1.94\times{10}^{5}$ ($2.89\times{10}^{5}$) | $1.54\times{10}^{5}$ ($3.65\times{10}^{5}$) | 0.3719 |
| **wavelet-HLH_firstorder_Uniformity** | 0.5003 (0.0010) | 0.5002 (0.0011) | 0.4958 |
| **wavelet-HLH_firstorder_Variance** | 2.1491 (2.2224) | 2.9703 (5.5883) | 0.2075 |
| **wavelet-HLH_glcm_Autocorrelation** | 2.4735 (0.8924) | 2.6344 (2.2105) | 0.5324 |
| **wavelet-HLH_glcm_ClusterProminence** | 0.5379 (0.0327) | 0.5490 (0.0365) | 0.0157 |
| **wavelet-HLH_glcm_ClusterShade** | 0.0012 (0.0184) | 0.0039 (0.0192) | 0.2755 |
| **wavelet-HLH_glcm_ClusterTendency** | 0.5364 (0.0303) | 0.5461 (0.0269) | 0.0062 |
| **wavelet-HLH_glcm_Contrast** | 0.4632 (0.0308) | 0.4539 (0.0282) | 0.0116 |
| **wavelet-HLH_glcm_Correlation** | 0.0733 (0.0611) | 0.0923 (0.0550) | 0.0085 |
| **wavelet-HLH_glcm_DifferenceAverage** | 0.4631 (0.0308) | 0.4537 (0.0280) | 0.0104 |
| **wavelet-HLH_glcm_DifferenceEntropy** | 0.9601 (0.0176) | 0.9588 (0.0133) | 0.481 |
| **wavelet-HLH_glcm_DifferenceVariance** | 0.2366 (0.0057) | 0.2362 (0.0043) | 0.4846 |
| **wavelet-HLH_glcm_Id** | 0.7684 (0.0154) | 0.7732 (0.0140) | 0.0101 |
| **wavelet-HLH_glcm_Idm** | 0.7684 (0.0154) | 0.7732 (0.0140) | 0.0102 |
| **wavelet-HLH_glcm_Idmn** | 0.9123 (0.0173) | 0.9189 (0.0208) | 0.0102 |
| **wavelet-HLH_glcm_Idn** | 0.8502 (0.0179) | 0.8574 (0.0207) | 0.0051 |
| **wavelet-HLH_glcm_Imc1** | -0.0401 (0.0174) | -0.0417 (0.0119) | 0.3751 |
| **wavelet-HLH_glcm_Imc2** | 0.2026 (0.0472) | 0.1999 (0.0373) | 0.598 |
| **wavelet-HLH_glcm_InverseVariance** | 0.4631 (0.0308) | 0.4536 (0.0279) | 0.0097 |
| **wavelet-HLH_glcm_JointAverage** | 1.5511 (0.2236) | 1.5709 (0.3794) | 0.6605 |
| **wavelet-HLH_glcm_JointEnergy** | 0.2638 (0.0060) | 0.2642 (0.0047) | 0.4986 |
| **wavelet-HLH_glcm_JointEntropy** | 1.9595 (0.0190) | 1.9588 (0.0174) | 0.7641 |
| **wavelet-HLH_glcm_MCC** | 0.1768 (0.0460) | 0.1753 (0.0341) | 0.7522 |
| **wavelet-HLH_glcm_MaximumProbability** | 0.2993 (0.0143) | 0.2986 (0.0142) | 0.7146 |
| **wavelet-HLH_glcm_SumAverage** | 3.1021 (0.4472) | 3.1419 (0.7588) | 0.6605 |
| **wavelet-HLH_glcm_SumEntropy** | 1.4962 (0.0254) | 1.5047 (0.0246) | 0.0075 |
| **wavelet-HLH_glcm_SumSquares** | 0.2499 (0.0008) | 0.2500 (0.0015) | 0.5674 |
| **wavelet-HLH_gldm_DependenceEntropy** | 4.9711 (0.1793) | 5.0303 (0.1573) | 0.0045 |
| **wavelet-HLH_gldm_DependenceNonUniformity** | $1.28\times{10}^{4}$ ($2.26\times{10}^{4}$) | 5798.1466 (9492.9068) | <0.0001 |
| **wavelet-HLH_gldm_DependenceNonUniformityNormalized** | 0.0752 (0.0111) | 0.0721 (0.0094) | 0.0138 |
| **wavelet-HLH_gldm_DependenceVariance** | 15.5342 (4.5694) | 16.5327 (4.0037) | 0.0593 |
| **wavelet-HLH_gldm_GrayLevelNonUniformity** | $7.40\times{10}^{4}$ ($1.20\times{10}^{5}$) | $3.62\times{10}^{4}$ ($5.39\times{10}^{4}$) | 0.0001 |
| **wavelet-HLH_gldm_GrayLevelVariance** | 0.2499 (0.0010) | 0.2501 (0.0019) | 0.5497 |
| **wavelet-HLH_gldm_HighGrayLevelEmphasis** | 2.7077 (0.8944) | 2.8652 (2.2125) | 0.5413 |
| **wavelet-HLH_gldm_LargeDependenceEmphasis** | 203.5903 (21.4004) | 212.6003 (17.5832) | 0.0002 |
| **wavelet-HLH_gldm_LargeDependenceHighGrayLevelEmphasis** | 547.8237 (191.9853) | 598.0526 (432.0559) | 0.3208 |
| **wavelet-HLH_gldm_LargeDependenceLowGrayLevelEmphasis** | 123.3919 (25.5628) | 129.7448 (25.5093) | 0.0524 |
| **wavelet-HLH_gldm_LowGrayLevelEmphasis** | 0.6019 (0.0997) | 0.6025 (0.1058) | 0.97 |
| **wavelet-HLH_gldm_SmallDependenceEmphasis** | 0.0083 (0.0020) | 0.0079 (0.0012) | 0.0381 |
| **wavelet-HLH_gldm_SmallDependenceHighGrayLevelEmphasis** | 0.0219 (0.0077) | 0.0230 (0.0232) | 0.6969 |
| **wavelet-HLH_gldm_SmallDependenceLowGrayLevelEmphasis** | 0.0051 (0.0016) | 0.0048 (0.0011) | 0.0573 |
| **wavelet-HLH_glrlm_GrayLevelNonUniformity** | $3.71\times{10}^{4}$ ($6.06\times{10}^{4}$) | $1.80\times{10}^{4}$ ($2.69\times{10}^{4}$) | 0.0001 |
| **wavelet-HLH_glrlm_GrayLevelNonUniformityNormalized** | 0.5001 (0.0012) | 0.5000 (0.0014) | 0.2833 |
| **wavelet-HLH_glrlm_GrayLevelVariance** | 0.2501 (0.0016) | 0.2503 (0.0034) | 0.4906 |
| **wavelet-HLH_glrlm_HighGrayLevelRunEmphasis** | 2.7149 (0.8969) | 2.8786 (2.2121) | 0.5257 |
| **wavelet-HLH_glrlm_LongRunEmphasis** | 6.7899 (0.8754) | 7.1965 (0.7614) | 0.0001 |
| **wavelet-HLH_glrlm_LongRunHighGrayLevelEmphasis** | 18.2976 (6.7437) | 20.2303 (13.8321) | 0.2352 |
| **wavelet-HLH_glrlm_LongRunLowGrayLevelEmphasis** | 4.1126 (0.8876) | 4.3807 (0.8899) | 0.019 |
| **wavelet-HLH_glrlm_LowGrayLevelRunEmphasis** | 0.6004 (0.0992) | 0.5993 (0.1049) | 0.9372 |
| **wavelet-HLH_glrlm_RunEntropy** | 2.9568 (0.1183) | 3.0063 (0.0863) | <0.0001 |
| **wavelet-HLH_glrlm_RunLengthNonUniformity** | $2.62\times{10}^{4}$ ($4.32\times{10}^{4}$) | $1.27\times{10}^{4}$ ($1.91\times{10}^{4}$) | 0.0001 |
| **wavelet-HLH_glrlm_RunLengthNonUniformityNormalized** | 0.3461 (0.0267) | 0.3350 (0.0195) | 0.0001 |
| **wavelet-HLH_glrlm_RunPercentage** | 0.5118 (0.0294) | 0.5003 (0.0217) | 0.0001 |
| **wavelet-HLH_glrlm_RunVariance** | 2.1840 (0.3936) | 2.3299 (0.3164) | 0.0007 |
| **wavelet-HLH_glrlm_ShortRunEmphasis** | 0.5769 (0.0261) | 0.5650 (0.0205) | <0.0001 |
| **wavelet-HLH_glrlm_ShortRunHighGrayLevelEmphasis** | 1.5625 (0.5016) | 1.6325 (1.2895) | 0.6406 |
| **wavelet-HLH_glrlm_ShortRunLowGrayLevelEmphasis** | 0.3470 (0.0612) | 0.3381 (0.0605) | 0.2481 |
| **wavelet-HLH_glszm_GrayLevelNonUniformity** | 14.7569 (12.8559) | 11.8847 (12.2972) | 0.0713 |
| **wavelet-HLH_glszm_GrayLevelNonUniformityNormalized** | 0.5488 (0.0895) | 0.5435 (0.0856) | 0.6324 |
| **wavelet-HLH_glszm_GrayLevelVariance** | 0.2463 (0.1538) | 0.2850 (0.2968) | 0.27 |
| **wavelet-HLH_glszm_HighGrayLevelZoneEmphasis** | 2.6477 (1.2302) | 2.8503 (2.7348) | 0.527 |
| **wavelet-HLH_glszm_LargeAreaEmphasis** | $5.89\times{10}^{8}$ ($1.56\times{10}^{9}$) | $1.80\times{10}^{8}$ ($5.73\times{10}^{8}$) | 0.0003 |
| **wavelet-HLH_glszm_LargeAreaHighGrayLevelEmphasis** | $1.53\times{10}^{9}$ ($3.90\times{10}^{9}$) | $4.88\times{10}^{8}$ ($1.46\times{10}^{9}$) | 0.0002 |
| **wavelet-HLH_glszm_LargeAreaLowGrayLevelEmphasis** | $3.63\times{10}^{8}$ ($9.80\times{10}^{8}$) | $1.09\times{10}^{8}$ ($3.58\times{10}^{8}$) | 0.0003 |
| **wavelet-HLH_glszm_LowGrayLevelZoneEmphasis** | 0.6290 (0.1551) | 0.6376 (0.1576) | 0.6678 |
| **wavelet-HLH_glszm_SizeZoneNonUniformity** | 10.1229 (9.8944) | 7.7036 (12.4694) | 0.1155 |
| **wavelet-HLH_glszm_SizeZoneNonUniformityNormalized** | 0.3463 (0.0981) | 0.3143 (0.0929) | 0.0081 |
| **wavelet-HLH_glszm_SmallAreaEmphasis** | 0.5192 (0.1737) | 0.5129 (0.1454) | 0.742 |
| **wavelet-HLH_glszm_SmallAreaHighGrayLevelEmphasis** | 1.4126 (0.8491) | 1.5122 (1.7786) | 0.6336 |
| **wavelet-HLH_glszm_SmallAreaLowGrayLevelEmphasis** | 0.3187 (0.1309) | 0.3232 (0.1295) | 0.7874 |
| **wavelet-HLH_glszm_ZoneEntropy** | 2.5659 (0.5835) | 2.7221 (0.4912) | 0.0174 |
| **wavelet-HLH_glszm_ZonePercentage** | 0.0009 (0.0013) | 0.0007 (0.0008) | 0.1003 |
| **wavelet-HLH_glszm_ZoneVariance** | $5.66\times{10}^{8}$ ($1.52\times{10}^{9}$) | $1.69\times{10}^{8}$ ($5.56\times{10}^{8}$) | 0.0003 |
| **wavelet-HLH_ngtdm_Busyness** | $3.18\times{10}^{4}$ ($5.72\times{10}^{4}$) | $1.26\times{10}^{4}$ ($1.76\times{10}^{4}$) | <0.0001 |
| **wavelet-HLH_ngtdm_Coarseness** | 0.0008 (0.0018) | 0.0004 (0.0008) | 0.0036 |
| **wavelet-HLH_ngtdm_Complexity** | 0.6748 (0.8304) | 0.9427 (2.0832) | 0.2698 |
| **wavelet-HLH_ngtdm_Contrast** | 0.1074 (0.0254) | 0.0971 (0.0320) | 0.0093 |
| **wavelet-HLH_ngtdm_Strength** | 0.0008 (0.0018) | 0.0005 (0.0009) | 0.0439 |
| **wavelet-HHL_firstorder_10Percentile** | -12.9011 (3.6942) | -11.9964 (3.6717) | 0.0551 |
| **wavelet-HHL_firstorder_90Percentile** | 12.8355 (3.7603) | 11.8402 (3.7493) | 0.0388 |
| **wavelet-HHL_firstorder_Energy** | $2.18\times{10}^{7}$ ($4.96\times{10}^{7}$) | $8.64\times{10}^{6}$ ($1.69\times{10}^{7}$) | 0.0002 |
| **wavelet-HHL_firstorder_Entropy** | 1.1842 (0.1609) | 1.1621 (0.1551) | 0.2704 |
| **wavelet-HHL_firstorder_InterquartileRange** | 13.3517 (3.8862) | 12.2709 (3.7746) | 0.0267 |
| **wavelet-HHL_firstorder_Kurtosis** | 7.9953 (8.6752) | 8.3958 (9.3846) | 0.735 |
| **wavelet-HHL_firstorder_Maximum** | 92.1143 (51.9619) | 89.0204 (52.2086) | 0.6431 |
| **wavelet-HHL_firstorder_MeanAbsoluteDeviation** | 8.1514 (2.2976) | 7.6007 (2.3259) | 0.0643 |
| **wavelet-HHL_firstorder_Mean** | -0.0220 (0.2720) | -0.0348 (0.2133) | 0.6594 |
| **wavelet-HHL_firstorder_Median** | -0.0561 (0.2316) | -0.0904 (0.1870) | 0.1738 |
| **wavelet-HHL_firstorder_Minimum** | -75.4837 (46.4197) | -69.7186 (41.0712) | 0.2865 |
| **wavelet-HHL_firstorder_Range** | 167.5980 (91.7314) | 158.7390 (89.3716) | 0.4414 |
| **wavelet-HHL_firstorder_RobustMeanAbsoluteDeviation** | 5.5497 (1.6123) | 5.1033 (1.5679) | 0.0275 |
| **wavelet-HHL_firstorder_RootMeanSquared** | 10.6401 (2.9598) | 10.0781 (3.1925) | 0.1634 |
| **wavelet-HHL_firstorder_Skewness** | 0.2649 (0.6648) | 0.3151 (0.5961) | 0.5215 |
| **wavelet-HHL_firstorder_TotalEnergy** | $2.18\times{10}^{7}$ ($4.96\times{10}^{7}$) | $8.64\times{10}^{6}$ ($1.69\times{10}^{7}$) | 0.0002 |
| **wavelet-HHL_firstorder_Uniformity** | 0.4710 (0.0309) | 0.4759 (0.0278) | 0.18 |
| **wavelet-HHL_firstorder_Variance** | 121.7850 (67.7925) | 111.6784 (81.3348) | 0.3164 |
| **wavelet-HHL_glcm_Autocorrelation** | 19.3504 (22.1393) | 17.1448 (17.8533) | 0.36 |
| **wavelet-HHL_glcm_ClusterProminence** | 2.1421 (3.6714) | 2.6966 (7.6107) | 0.5354 |
| **wavelet-HHL_glcm_ClusterShade** | 0.0624 (0.1751) | 0.0900 (0.2263) | 0.3215 |
| **wavelet-HHL_glcm_ClusterTendency** | 0.6387 (0.1609) | 0.6329 (0.2279) | 0.8331 |
| **wavelet-HHL_glcm_Contrast** | 0.6377 (0.1705) | 0.6163 (0.2105) | 0.4106 |
| **wavelet-HHL_glcm_Correlation** | 0.0013 (0.0229) | 0.0115 (0.0272) | 0.0027 |
| **wavelet-HHL_glcm_DifferenceAverage** | 0.5561 (0.0664) | 0.5427 (0.0662) | 0.1136 |
| **wavelet-HHL_glcm_DifferenceEntropy** | 1.0848 (0.1384) | 1.0683 (0.1302) | 0.327 |
| **wavelet-HHL_glcm_DifferenceVariance** | 0.2986 (0.0869) | 0.2950 (0.1182) | 0.8052 |
| **wavelet-HHL_glcm_Id** | 0.7336 (0.0191) | 0.7385 (0.0178) | 0.0347 |
| **wavelet-HHL_glcm_Idm** | 0.7297 (0.0237) | 0.7354 (0.0218) | 0.0458 |
| **wavelet-HHL_glcm_Idmn** | 0.9829 (0.0147) | 0.9833 (0.0124) | 0.8063 |
| **wavelet-HHL_glcm_Idn** | 0.9271 (0.0267) | 0.9271 (0.0238) | 0.9946 |
| **wavelet-HHL_glcm_Imc1** | -0.0741 (0.0126) | -0.0679 (0.0135) | 0.0003 |
| **wavelet-HHL_glcm_Imc2** | 0.3021 (0.0523) | 0.2795 (0.0603) | 0.0028 |
| **wavelet-HHL_glcm_InverseVariance** | 0.5002 (0.0053) | 0.4969 (0.0086) | 0.001 |
| **wavelet-HHL_glcm_JointAverage** | 3.9911 (1.8618) | 3.8048 (1.6350) | 0.3877 |
| **wavelet-HHL_glcm_JointEnergy** | 0.2447 (0.0284) | 0.2481 (0.0243) | 0.2952 |
| **wavelet-HHL_glcm_JointEntropy** | 2.2605 (0.3036) | 2.2212 (0.2893) | 0.2938 |
| **wavelet-HHL_glcm_MCC** | 0.3451 (0.1006) | 0.3191 (0.1064) | 0.0547 |
| **wavelet-HHL_glcm_MaximumProbability** | 0.2903 (0.0213) | 0.2898 (0.0186) | 0.8411 |
| **wavelet-HHL_glcm_SumAverage** | 7.9822 (3.7237) | 7.6096 (3.2700) | 0.3877 |
| **wavelet-HHL_glcm_SumEntropy** | 1.6024 (0.1532) | 1.5912 (0.1512) | 0.5627 |
| **wavelet-HHL_glcm_SumSquares** | 0.3191 (0.0824) | 0.3123 (0.1090) | 0.6099 |
| **wavelet-HHL_gldm_DependenceEntropy** | 4.7912 (0.1830) | 4.7931 (0.1785) | 0.9326 |
| **wavelet-HHL_gldm_DependenceNonUniformity** | $1.37\times{10}^{4}$ ($2.14\times{10}^{4}$) | 6820.1187 ($1.04\times{10}^{4}$) | 0.0001 |
| **wavelet-HHL_gldm_DependenceNonUniformityNormalized** | 0.0888 (0.0106) | 0.0889 (0.0092) | 0.9521 |
| **wavelet-HHL_gldm_DependenceVariance** | 11.8084 (2.5052) | 11.7314 (2.3970) | 0.8035 |
| **wavelet-HHL_gldm_GrayLevelNonUniformity** | $6.74\times{10}^{4}$ ($1.05\times{10}^{5}$) | $3.42\times{10}^{4}$ ($5.01\times{10}^{4}$) | 0.0001 |
| **wavelet-HHL_gldm_GrayLevelVariance** | 0.3247 (0.0836) | 0.3184 (0.1093) | 0.6373 |
| **wavelet-HHL_gldm_HighGrayLevelEmphasis** | 19.6788 (22.1680) | 17.4636 (17.9114) | 0.3592 |
| **wavelet-HHL_gldm_LargeDependenceEmphasis** | 163.4184 (17.8026) | 169.4857 (14.4269) | 0.0019 |
| **wavelet-HHL_gldm_LargeDependenceHighGrayLevelEmphasis** | 3258.2736 (3788.3960) | 2901.6517 (2862.9916) | 0.3653 |
| **wavelet-HHL_gldm_LargeDependenceLowGrayLevelEmphasis** | 18.1610 (16.4089) | 19.1073 (14.0496) | 0.6118 |
| **wavelet-HHL_gldm_LowGrayLevelEmphasis** | 0.1154 (0.1049) | 0.1138 (0.0814) | 0.8866 |
| **wavelet-HHL_gldm_SmallDependenceEmphasis** | 0.0137 (0.0049) | 0.0128 (0.0052) | 0.1801 |
| **wavelet-HHL_gldm_SmallDependenceHighGrayLevelEmphasis** | 0.2991 (0.3876) | 0.2752 (0.4236) | 0.6538 |
| **wavelet-HHL_gldm_SmallDependenceLowGrayLevelEmphasis** | 0.0020 (0.0021) | 0.0017 (0.0014) | 0.1333 |
| **wavelet-HHL_glrlm_GrayLevelNonUniformity** | $3.55\times{10}^{4}$ ($5.51\times{10}^{4}$) | $1.80\times{10}^{4}$ ($2.60\times{10}^{4}$) | 0.0001 |
| **wavelet-HHL_glrlm_GrayLevelNonUniformityNormalized** | 0.4555 (0.0426) | 0.4620 (0.0382) | 0.1988 |
| **wavelet-HHL_glrlm_GrayLevelVariance** | 0.3700 (0.1260) | 0.3607 (0.1556) | 0.6268 |
| **wavelet-HHL_glrlm_HighGrayLevelRunEmphasis** | 19.7467 (22.1976) | 17.5439 (17.9593) | 0.363 |
| **wavelet-HHL_glrlm_LongRunEmphasis** | 9.4937 (3.0454) | 9.7216 (2.7750) | 0.5298 |
| **wavelet-HHL_glrlm_LongRunHighGrayLevelEmphasis** | 200.5663 (279.7162) | 165.3815 (162.7647) | 0.1541 |
| **wavelet-HHL_glrlm_LongRunLowGrayLevelEmphasis** | 1.0094 (0.8536) | 1.0910 (0.8540) | 0.4551 |
| **wavelet-HHL_glrlm_LowGrayLevelRunEmphasis** | 0.1168 (0.1056) | 0.1149 (0.0819) | 0.8593 |
| **wavelet-HHL_glrlm_RunEntropy** | 3.0129 (0.1702) | 3.0267 (0.1359) | 0.4534 |
| **wavelet-HHL_glrlm_RunLengthNonUniformity** | $3.42\times{10}^{4}$ ($5.88\times{10}^{4}$) | $1.60\times{10}^{4}$ ($2.39\times{10}^{4}$) | <0.0001 |
| **wavelet-HHL_glrlm_RunLengthNonUniformityNormalized** | 0.3953 (0.0333) | 0.3861 (0.0265) | 0.0108 |
| **wavelet-HHL_glrlm_RunPercentage** | 0.5659 (0.0316) | 0.5561 (0.0259) | 0.0049 |
| **wavelet-HHL_glrlm_RunVariance** | 3.1997 (1.2050) | 3.2836 (1.0558) | 0.5469 |
| **wavelet-HHL_glrlm_ShortRunEmphasis** | 0.6188 (0.0326) | 0.6102 (0.0275) | 0.0201 |
| **wavelet-HHL_glrlm_ShortRunHighGrayLevelEmphasis** | 12.2134 (13.6939) | 10.8706 (11.5243) | 0.3823 |
| **wavelet-HHL_glrlm_ShortRunLowGrayLevelEmphasis** | 0.0740 (0.0679) | 0.0707 (0.0501) | 0.6329 |
| **wavelet-HHL_glszm_GrayLevelNonUniformity** | 543.3673 (1440.8114) | 203.7265 (490.8289) | 0.0008 |
| **wavelet-HHL_glszm_GrayLevelNonUniformityNormalized** | 0.3768 (0.0942) | 0.3712 (0.0855) | 0.6148 |
| **wavelet-HHL_glszm_GrayLevelVariance** | 2.5344 (1.1121) | 2.5537 (1.1230) | 0.8929 |
| **wavelet-HHL_glszm_HighGrayLevelZoneEmphasis** | 22.7835 (22.8185) | 20.6658 (19.2204) | 0.4086 |
| **wavelet-HHL_glszm_LargeAreaEmphasis** | $2.89\times{10}^{7}$ ($7.18\times{10}^{7}$) | $2.78\times{10}^{7}$ ($1.06\times{10}^{8}$) | 0.9331 |
| **wavelet-HHL_glszm_LargeAreaHighGrayLevelEmphasis** | $7.39\times{10}^{8}$ ($2.45\times{10}^{9}$) | $2.87\times{10}^{8}$ ($8.47\times{10}^{8}$) | 0.0088 |
| **wavelet-HHL_glszm_LargeAreaLowGrayLevelEmphasis** | $2.10\times{10}^{6}$ ($4.56\times{10}^{6}$) | $4.83\times{10}^{6}$ ($2.52\times{10}^{7}$) | 0.3452 |
| **wavelet-HHL_glszm_LowGrayLevelZoneEmphasis** | 0.2205 (0.1875) | 0.2351 (0.1855) | 0.5388 |
| **wavelet-HHL_glszm_SizeZoneNonUniformity** | 155.7014 (339.9376) | 71.8491 (139.1134) | 0.001 |
| **wavelet-HHL_glszm_SizeZoneNonUniformityNormalized** | 0.1805 (0.0512) | 0.1900 (0.0575) | 0.1859 |
| **wavelet-HHL_glszm_SmallAreaEmphasis** | 0.3204 (0.1007) | 0.3544 (0.0946) | 0.006 |
| **wavelet-HHL_glszm_SmallAreaHighGrayLevelEmphasis** | 7.9342 (9.9554) | 7.2305 (6.7789) | 0.4661 |
| **wavelet-HHL_glszm_SmallAreaLowGrayLevelEmphasis** | 0.0711 (0.0686) | 0.0927 (0.0894) | 0.0492 |
| **wavelet-HHL_glszm_ZoneEntropy** | 4.1121 (0.8915) | 4.1126 (0.8000) | 0.9958 |
| **wavelet-HHL_glszm_ZonePercentage** | 0.0073 (0.0055) | 0.0064 (0.0059) | 0.2423 |
| **wavelet-HHL_glszm_ZoneVariance** | $2.82\times{10}^{7}$ ($7.10\times{10}^{7}$) | $2.55\times{10}^{7}$ ($9.42\times{10}^{7}$) | 0.8142 |
| **wavelet-HHL_ngtdm_Busyness** | 586.6316 (680.1349) | 573.0379 (1419.3436) | 0.935 |
| **wavelet-HHL_ngtdm_Coarseness** | 0.0007 (0.0016) | 0.0003 (0.0007) | 0.0048 |
| **wavelet-HHL_ngtdm_Complexity** | 19.1750 (18.9460) | 17.8283 (22.3872) | 0.6283 |
| **wavelet-HHL_ngtdm_Contrast** | 0.0130 (0.0155) | 0.0121 (0.0121) | 0.6035 |
| **wavelet-HHL_ngtdm_Strength** | 0.0117 (0.0363) | 0.0079 (0.0157) | 0.1655 |
| **wavelet-HHH_firstorder_10Percentile** | -0.8394 (0.2753) | -0.8336 (0.5743) | 0.9318 |
| **wavelet-HHH_firstorder_90Percentile** | 0.8426 (0.2745) | 0.8366 (0.5721) | 0.9279 |
| **wavelet-HHH_firstorder_Energy** | $8.86\times{10}^{4}$ ($1.80\times{10}^{5}$) | $5.03\times{10}^{4}$ ($2.16\times{10}^{5}$) | 0.1534 |
| **wavelet-HHH_firstorder_Entropy** | 0.9999 (0.0002) | 1.0000 (0.0012) | 0.4741 |
| **wavelet-HHH_firstorder_InterquartileRange** | 0.8509 (0.2734) | 0.8433 (0.5869) | 0.9115 |
| **wavelet-HHH_firstorder_Kurtosis** | 5.3741 (3.1693) | 7.9900 (27.2740) | 0.4018 |
| **wavelet-HHH_firstorder_Maximum** | 5.0695 (3.6714) | 5.6303 (5.0802) | 0.3642 |
| **wavelet-HHH_firstorder_MeanAbsoluteDeviation** | 0.5329 (0.1731) | 0.5318 (0.3599) | 0.9801 |
| **wavelet-HHH_firstorder_Mean** | 0.0009 (0.0068) | 0.0012 (0.0085) | 0.7505 |
| **wavelet-HHH_firstorder_Median** | 0.0010 (0.0064) | $9.94\times{10}^{-5}$ (0.0067) | 0.2694 |
| **wavelet-HHH_firstorder_Minimum** | -5.6467 (3.3424) | -5.9438 (5.1075) | 0.6287 |
| **wavelet-HHH_firstorder_Range** | 10.7162 (6.6230) | 11.5741 (10.0535) | 0.4785 |
| **wavelet-HHH_firstorder_RobustMeanAbsoluteDeviation** | 0.3556 (0.1148) | 0.3525 (0.2453) | 0.9145 |
| **wavelet-HHH_firstorder_RootMeanSquared** | 0.6987 (0.2276) | 0.7044 (0.4655) | 0.9176 |
| **wavelet-HHH_firstorder_Skewness** | -0.0294 (0.1511) | -0.0341 (0.3997) | 0.9192 |
| **wavelet-HHH_firstorder_TotalEnergy** | $8.86\times{10}^{4}$ ($1.80\times{10}^{5}$) | $5.03\times{10}^{4}$ ($2.16\times{10}^{5}$) | 0.1534 |
| **wavelet-HHH_firstorder_Uniformity** | 0.5000 (0.0002) | 0.5000 (0.0002) | 0.5142 |
| **wavelet-HHH_firstorder_Variance** | 0.5393 (0.4351) | 0.7121 (3.1567) | 0.6325 |
| **wavelet-HHH_glcm_Autocorrelation** | 2.2488 (0.0126) | 2.3088 (0.6662) | 0.4301 |
| **wavelet-HHH_glcm_ClusterProminence** | 0.4925 (0.0046) | 0.4953 (0.0065) | 0.0006 |
| **wavelet-HHH_glcm_ClusterShade** | -0.0008 (0.0040) | -0.0002 (0.0042) | 0.2177 |
| **wavelet-HHH_glcm_ClusterTendency** | 0.4925 (0.0046) | 0.4952 (0.0064) | 0.0008 |
| **wavelet-HHH_glcm_Contrast** | 0.5074 (0.0047) | 0.5048 (0.0065) | 0.0013 |
| **wavelet-HHH_glcm_Correlation** | -0.0148 (0.0094) | -0.0097 (0.0129) | 0.001 |
| **wavelet-HHH_glcm_DifferenceAverage** | 0.5074 (0.0047) | 0.5048 (0.0065) | 0.0012 |
| **wavelet-HHH_glcm_DifferenceEntropy** | 0.9795 (0.0086) | 0.9819 (0.0071) | 0.0109 |
| **wavelet-HHH_glcm_DifferenceVariance** | 0.2430 (0.0029) | 0.2438 (0.0024) | 0.0112 |
| **wavelet-HHH_glcm_Id** | 0.7463 (0.0024) | 0.7476 (0.0032) | 0.0012 |
| **wavelet-HHH_glcm_Idm** | 0.7463 (0.0024) | 0.7476 (0.0032) | 0.0012 |
| **wavelet-HHH_glcm_Idmn** | 0.8992 (0.0058) | 0.8998 (0.0072) | 0.5229 |
| **wavelet-HHH_glcm_Idn** | 0.8314 (0.0050) | 0.8324 (0.0074) | 0.2579 |
| **wavelet-HHH_glcm_Imc1** | -0.0205 (0.0086) | -0.0182 (0.0070) | 0.0121 |
| **wavelet-HHH_glcm_Imc2** | 0.1519 (0.0414) | 0.1428 (0.0363) | 0.0586 |
| **wavelet-HHH_glcm_InverseVariance** | 0.5074 (0.0047) | 0.5048 (0.0065) | 0.0011 |
| **wavelet-HHH_glcm_JointAverage** | 1.5008 (0.0040) | 1.5137 (0.1420) | 0.4294 |
| **wavelet-HHH_glcm_JointEnergy** | 0.2570 (0.0029) | 0.2562 (0.0024) | 0.0111 |
| **wavelet-HHH_glcm_JointEntropy** | 1.9794 (0.0085) | 1.9819 (0.0073) | 0.01 |
| **wavelet-HHH_glcm_MCC** | 0.1292 (0.0364) | 0.1213 (0.0315) | 0.0574 |
| **wavelet-HHH_glcm_MaximumProbability** | 0.2835 (0.0085) | 0.2815 (0.0078) | 0.0483 |
| **wavelet-HHH_glcm_SumAverage** | 3.0017 (0.0080) | 3.0273 (0.2841) | 0.4294 |
| **wavelet-HHH_glcm_SumEntropy** | 1.4720 (0.0115) | 1.4770 (0.0114) | 0.0006 |
| **wavelet-HHH_glcm_SumSquares** | 0.2500 ($5.38\times{10}^{-5}$) | 0.2500 (0.0002) | 0.5472 |
| **wavelet-HHH_gldm_DependenceEntropy** | 4.3912 (0.1848) | 4.4254 (0.1546) | 0.0988 |
| **wavelet-HHH_gldm_DependenceNonUniformity** | $1.97\times{10}^{4}$ ($3.33\times{10}^{4}$) | 9086.5520 ($1.45\times{10}^{4}$) | <0.0001 |
| **wavelet-HHH_gldm_DependenceNonUniformityNormalized** | 0.1152 (0.0180) | 0.1129 (0.0143) | 0.2355 |
| **wavelet-HHH_gldm_DependenceVariance** | 7.2123 (1.9045) | 7.4451 (1.6073) | 0.2772 |
| **wavelet-HHH_gldm_GrayLevelNonUniformity** | $7.40\times{10}^{4}$ ($1.20\times{10}^{5}$) | $3.62\times{10}^{4}$ ($5.39\times{10}^{4}$) | 0.0001 |
| **wavelet-HHH_gldm_GrayLevelVariance** | 0.2500 ($7.91\times{10}^{-5}$) | 0.2500 (0.0002) | 0.4652 |
| **wavelet-HHH_gldm_HighGrayLevelEmphasis** | 2.5021 (0.0140) | 2.5608 (0.6666) | 0.4408 |
| **wavelet-HHH_gldm_LargeDependenceEmphasis** | 167.1121 (15.2384) | 170.4017 (9.9757) | 0.0229 |
| **wavelet-HHH_gldm_LargeDependenceHighGrayLevelEmphasis** | 419.0189 (37.0725) | 436.6795 (115.9336) | 0.1883 |
| **wavelet-HHH_gldm_LargeDependenceLowGrayLevelEmphasis** | 104.1354 (9.9248) | 105.5963 (10.3355) | 0.2661 |
| **wavelet-HHH_gldm_LowGrayLevelEmphasis** | 0.6245 (0.0035) | 0.6202 (0.0480) | 0.4352 |
| **wavelet-HHH_gldm_SmallDependenceEmphasis** | 0.0081 (0.0018) | 0.0078 (0.0012) | 0.2048 |
| **wavelet-HHH_gldm_SmallDependenceHighGrayLevelEmphasis** | 0.0201 (0.0044) | 0.0202 (0.0067) | 0.9921 |
| **wavelet-HHH_gldm_SmallDependenceLowGrayLevelEmphasis** | 0.0050 (0.0011) | 0.0049 (0.0008) | 0.1205 |
| **wavelet-HHH_glrlm_GrayLevelNonUniformity** | $3.92\times{10}^{4}$ ($6.32\times{10}^{4}$) | $1.93\times{10}^{4}$ ($2.83\times{10}^{4}$) | 0.0001 |
| **wavelet-HHH_glrlm_GrayLevelNonUniformityNormalized** | 0.5000 ($8.52\times{10}^{-5}$) | 0.5000 (0.0002) | 0.2358 |
| **wavelet-HHH_glrlm_GrayLevelVariance** | 0.2500 ($4.32\times{10}^{-5}$) | 0.2500 (0.0004) | 0.4271 |
| **wavelet-HHH_glrlm_HighGrayLevelRunEmphasis** | 2.5000 (0.0104) | 2.5602 (0.6666) | 0.4297 |
| **wavelet-HHH_glrlm_LongRunEmphasis** | 5.1331 (0.6796) | 5.1890 (0.4506) | 0.3887 |
| **wavelet-HHH_glrlm_LongRunHighGrayLevelEmphasis** | 12.8557 (1.6792) | 13.2868 (3.5254) | 0.2981 |
| **wavelet-HHH_glrlm_LongRunLowGrayLevelEmphasis** | 3.2024 (0.4310) | 3.2170 (0.3756) | 0.7693 |
| **wavelet-HHH_glrlm_LowGrayLevelRunEmphasis** | 0.6250 (0.0026) | 0.6204 (0.0480) | 0.3975 |
| **wavelet-HHH_glrlm_RunEntropy** | 2.7867 (0.1061) | 2.8052 (0.0671) | 0.0609 |
| **wavelet-HHH_glrlm_RunLengthNonUniformity** | $2.90\times{10}^{4}$ ($4.61\times{10}^{4}$) | $1.44\times{10}^{4}$ ($2.07\times{10}^{4}$) | <0.0001 |
| **wavelet-HHH_glrlm_RunLengthNonUniformityNormalized** | 0.3802 (0.0244) | 0.3758 (0.0158) | 0.0563 |
| **wavelet-HHH_glrlm_RunPercentage** | 0.5528 (0.0268) | 0.5478 (0.0173) | 0.0451 |
| **wavelet-HHH_glrlm_RunVariance** | 1.5718 (0.2804) | 1.6041 (0.1845) | 0.2258 |
| **wavelet-HHH_glrlm_ShortRunEmphasis** | 0.6217 (0.0240) | 0.6183 (0.0155) | 0.1279 |
| **wavelet-HHH_glrlm_ShortRunHighGrayLevelEmphasis** | 1.5533 (0.0594) | 1.5825 (0.4108) | 0.5356 |
| **wavelet-HHH_glrlm_ShortRunLowGrayLevelEmphasis** | 0.3888 (0.0154) | 0.3836 (0.0313) | 0.1598 |
| **wavelet-HHH_glszm_GrayLevelNonUniformity** | 2.4012 (1.5711) | 2.5434 (2.4534) | 0.6291 |
| **wavelet-HHH_glszm_GrayLevelNonUniformityNormalized** | 0.5388 (0.0530) | 0.5400 (0.0550) | 0.8645 |
| **wavelet-HHH_glszm_GrayLevelVariance** | 0.2347 (0.0527) | 0.2429 (0.1531) | 0.6466 |
| **wavelet-HHH_glszm_HighGrayLevelZoneEmphasis** | 2.5685 (0.4491) | 2.5668 (1.0038) | 0.989 |
| **wavelet-HHH_glszm_LargeAreaEmphasis** | $4.68\times{10}^{9}$ ($1.16\times{10}^{10}$) | $1.40\times{10}^{9}$ ($6.46\times{10}^{9}$) | 0.0011 |
| **wavelet-HHH_glszm_LargeAreaHighGrayLevelEmphasis** | $1.17\times{10}^{10}$ ($2.91\times{10}^{10}$) | $3.54\times{10}^{9}$ ($1.62\times{10}^{10}$) | 0.0012 |
| **wavelet-HHH_glszm_LargeAreaLowGrayLevelEmphasis** | $2.92\times{10}^{9}$ ($7.27\times{10}^{9}$) | $8.70\times{10}^{8}$ ($4.04\times{10}^{9}$) | 0.0011 |
| **wavelet-HHH_glszm_LowGrayLevelZoneEmphasis** | 0.6112 (0.1069) | 0.6246 (0.1127) | 0.3495 |
| **wavelet-HHH_glszm_SizeZoneNonUniformity** | 1.6729 (1.2217) | 1.8781 (4.0551) | 0.6614 |
| **wavelet-HHH_glszm_SizeZoneNonUniformityNormalized** | 0.3924 (0.0977) | 0.3824 (0.1036) | 0.4442 |
| **wavelet-HHH_glszm_SmallAreaEmphasis** | 0.3048 (0.2433) | 0.3116 (0.2368) | 0.8251 |
| **wavelet-HHH_glszm_SmallAreaHighGrayLevelEmphasis** | 0.8060 (0.7494) | 0.8209 (1.1041) | 0.9111 |
| **wavelet-HHH_glszm_SmallAreaLowGrayLevelEmphasis** | 0.1820 (0.1760) | 0.1967 (0.1787) | 0.5189 |
| **wavelet-HHH_glszm_ZoneEntropy** | 1.6483 (0.5310) | 1.6864 (0.5109) | 0.5642 |
| **wavelet-HHH_glszm_ZonePercentage** | 0.0004 (0.0008) | 0.0002 (0.0004) | 0.0187 |
| **wavelet-HHH_glszm_ZoneVariance** | $3.28\times{10}^{9}$ ($9.33\times{10}^{9}$) | $8.15\times{10}^{8}$ ($4.34\times{10}^{9}$) | 0.0008 |
| **wavelet-HHH_ngtdm_Busyness** | $3.70\times{10}^{4}$ ($6.16\times{10}^{4}$) | $1.82\times{10}^{4}$ ($2.74\times{10}^{4}$) | 0.0001 |
| **wavelet-HHH_ngtdm_Coarseness** | 0.0006 (0.0015) | 0.0003 (0.0007) | 0.0036 |
| **wavelet-HHH_ngtdm_Complexity** | 0.5269 (0.1748) | 0.5474 (0.5180) | 0.7327 |
| **wavelet-HHH_ngtdm_Contrast** | 0.1256 (0.0097) | 0.1250 (0.0105) | 0.6068 |
| **wavelet-HHH_ngtdm_Strength** | 0.0006 (0.0015) | 0.0003 (0.0007) | 0.0041 |
| **wavelet-LLL_firstorder_10Percentile** | -18.8406 (106.9406) | 68.3341 (93.0975) | <0.0001 |
| **wavelet-LLL_firstorder_90Percentile** | 155.1098 (77.4886) | 252.5676 (63.2727) | <0.0001 |
| **wavelet-LLL_firstorder_Energy** | $2.64\times{10}^{9}$ ($4.64\times{10}^{9}$) | $3.26\times{10}^{9}$ ($4.73\times{10}^{9}$) | 0.309 |
| **wavelet-LLL_firstorder_Entropy** | 3.3594 (0.5380) | 3.5098 (0.4347) | 0.0106 |
| **wavelet-LLL_firstorder_InterquartileRange** | 84.5229 (62.6853) | 79.3754 (32.1592) | 0.3192 |
| **wavelet-LLL_firstorder_Kurtosis** | 26.8218 (20.9138) | 31.2839 (28.1327) | 0.1941 |
| **wavelet-LLL_firstorder_Maximum** | 566.5010 (306.7085) | 699.8645 (576.6533) | 0.0511 |
| **wavelet-LLL_firstorder_MeanAbsoluteDeviation** | 61.1527 (33.1775) | 67.6387 (30.4330) | 0.103 |
| **wavelet-LLL_firstorder_Mean** | 70.6130 (80.0619) | 166.4241 (62.3310) | <0.0001 |
| **wavelet-LLL_firstorder_Median** | 79.8860 (83.6079) | 184.7855 (58.9889) | <0.0001 |
| **wavelet-LLL_firstorder_Minimum** | -1052.8967 (492.5467) | -1168.5130 (496.7013) | 0.0692 |
| **wavelet-LLL_firstorder_Range** | 1619.3978 (635.3271) | 1868.3775 (785.3643) | 0.0105 |
| **wavelet-LLL_firstorder_RobustMeanAbsoluteDeviation** | 36.3012 (25.2065) | 35.0564 (14.2922) | 0.5698 |
| **wavelet-LLL_firstorder_RootMeanSquared** | 137.1691 (58.8023) | 209.3241 (60.2025) | <0.0001 |
| **wavelet-LLL_firstorder_Skewness** | -2.3936 (1.8620) | -3.0115 (2.3834) | 0.0353 |
| **wavelet-LLL_firstorder_TotalEnergy** | $2.64\times{10}^{9}$ ($4.64\times{10}^{9}$) | $3.26\times{10}^{9}$ ($4.73\times{10}^{9}$) | 0.309 |
| **wavelet-LLL_firstorder_Uniformity** | 0.1428 (0.0546) | 0.1300 (0.0382) | 0.0174 |
| **wavelet-LLL_firstorder_Variance** | $1.09\times{10}^{4}$ ($1.23\times{10}^{4}$) | $1.59\times{10}^{4}$ ($2.22\times{10}^{4}$) | 0.0615 |
| **wavelet-LLL_glcm_Autocorrelation** | 2536.6663 (2078.9681) | 3408.7609 (2349.2783) | 0.0032 |
| **wavelet-LLL_glcm_ClusterProminence** | $1.09\times{10}^{5}$ ($3.19\times{10}^{5}$) | $5.42\times{10}^{5}$ ($3.93\times{10}^{6}$) | 0.3357 |
| **wavelet-LLL_glcm_ClusterShade** | -1127.3289 (3311.8441) | -1160.9581 ($1.83\times{10}^{4}$) | 0.9872 |
| **wavelet-LLL_glcm_ClusterTendency** | 52.2907 (62.1200) | 73.3682 (117.8936) | 0.1309 |
| **wavelet-LLL_glcm_Contrast** | 4.1393 (3.3795) | 6.4772 (7.0813) | 0.0052 |
| **wavelet-LLL_glcm_Correlation** | 0.8139 (0.0673) | 0.8125 (0.0672) | 0.8647 |
| **wavelet-LLL_glcm_DifferenceAverage** | 1.1031 (0.4151) | 1.2556 (0.5050) | 0.015 |
| **wavelet-LLL_glcm_DifferenceEntropy** | 1.8904 (0.3564) | 2.0172 (0.3505) | 0.0051 |
| **wavelet-LLL_glcm_DifferenceVariance** | 2.6989 (2.3885) | 4.5721 (5.2831) | 0.0026 |
| **wavelet-LLL_glcm_Id** | 0.6428 (0.0713) | 0.6275 (0.0632) | 0.0659 |
| **wavelet-LLL_glcm_Idm** | 0.6140 (0.0857) | 0.5966 (0.0752) | 0.0787 |
| **wavelet-LLL_glcm_Idmn** | 0.9981 (0.0033) | 0.9986 (0.0018) | 0.0682 |
| **wavelet-LLL_glcm_Idn** | 0.9797 (0.0168) | 0.9822 (0.0100) | 0.1026 |
| **wavelet-LLL_glcm_Imc1** | -0.3001 (0.0519) | -0.2916 (0.0452) | 0.1536 |
| **wavelet-LLL_glcm_Imc2** | 0.9063 (0.0518) | 0.9129 (0.0374) | 0.2078 |
| **wavelet-LLL_glcm_InverseVariance** | 0.4382 (0.0316) | 0.4386 (0.0316) | 0.9192 |
| **wavelet-LLL_glcm_JointAverage** | 46.1344 (20.0362) | 54.7601 (19.8668) | 0.0008 |
| **wavelet-LLL_glcm_JointEnergy** | 0.0503 (0.0303) | 0.0421 (0.0208) | 0.0056 |
| **wavelet-LLL_glcm_JointEntropy** | 5.5188 (0.8773) | 5.7902 (0.7481) | 0.0066 |
| **wavelet-LLL_glcm_MCC** | 0.8737 (0.0522) | 0.8686 (0.0495) | 0.4233 |
| **wavelet-LLL_glcm_MaximumProbability** | 0.1204 (0.0636) | 0.1032 (0.0460) | 0.0078 |
| **wavelet-LLL_glcm_SumAverage** | 92.2689 (40.0723) | 109.5202 (39.7337) | 0.0008 |
| **wavelet-LLL_glcm_SumEntropy** | 4.1652 (0.5594) | 4.3126 (0.4417) | 0.0143 |
| **wavelet-LLL_glcm_SumSquares** | 14.1075 (16.2062) | 19.9614 (30.8542) | 0.1089 |
| **wavelet-LLL_gldm_DependenceEntropy** | 7.3004 (0.4487) | 7.4636 (0.3105) | 0.0002 |
| **wavelet-LLL_gldm_DependenceNonUniformity** | 8022.3737 ($1.38\times{10}^{4}$) | 3925.0865 (6045.5791) | 0.0001 |
| **wavelet-LLL_gldm_DependenceNonUniformityNormalized** | 0.0589 (0.0187) | 0.0577 (0.0147) | 0.5564 |
| **wavelet-LLL_gldm_DependenceVariance** | 28.8387 (9.4592) | 28.3721 (9.1572) | 0.6926 |
| **wavelet-LLL_gldm_GrayLevelNonUniformity** | $2.54\times{10}^{4}$ ($5.09\times{10}^{4}$) | $1.08\times{10}^{4}$ ($1.93\times{10}^{4}$) | 0.0001 |
| **wavelet-LLL_gldm_GrayLevelVariance** | 17.5454 (19.6738) | 25.5632 (35.6935) | 0.0587 |
| **wavelet-LLL_gldm_HighGrayLevelEmphasis** | 2525.7505 (2071.2907) | 3384.5423 (2334.1367) | 0.0035 |
| **wavelet-LLL_gldm_LargeDependenceEmphasis** | 137.8691 (55.3066) | 127.2080 (45.9837) | 0.0836 |
| **wavelet-LLL_gldm_LargeDependenceHighGrayLevelEmphasis** | $3.64\times{10}^{5}$ ($3.53\times{10}^{5}$) | $4.42\times{10}^{5}$ ($3.54\times{10}^{5}$) | 0.0844 |
| **wavelet-LLL_gldm_LargeDependenceLowGrayLevelEmphasis** | 0.1595 (0.2766) | 0.0822 (0.2010) | 0.0059 |
| **wavelet-LLL_gldm_LowGrayLevelEmphasis** | 0.0022 (0.0048) | 0.0009 (0.0021) | 0.0004 |
| **wavelet-LLL_gldm_SmallDependenceEmphasis** | 0.0607 (0.0379) | 0.0705 (0.0395) | 0.0534 |
| **wavelet-LLL_gldm_SmallDependenceHighGrayLevelEmphasis** | 127.7414 (128.3926) | 213.4920 (203.5110) | 0.0005 |
| **wavelet-LLL_gldm_SmallDependenceLowGrayLevelEmphasis** | 0.0004 (0.0010) | 0.0002 (0.0004) | 0.0009 |
| **wavelet-LLL_glrlm_GrayLevelNonUniformity** | $1.29\times{10}^{4}$ ($2.66\times{10}^{4}$) | 5641.1394 (9808.8405) | 0.0002 |
| **wavelet-LLL_glrlm_GrayLevelNonUniformityNormalized** | 0.1198 (0.0413) | 0.1094 (0.0290) | 0.0108 |
| **wavelet-LLL_glrlm_GrayLevelVariance** | 22.1105 (23.7102) | 33.6461 (48.7178) | 0.0446 |
| **wavelet-LLL_glrlm_HighGrayLevelRunEmphasis** | 2506.7362 (2056.8893) | 3343.0786 (2301.3846) | 0.0039 |
| **wavelet-LLL_glrlm_LongRunEmphasis** | 4.2740 (1.9350) | 3.8050 (1.4534) | 0.0196 |
| **wavelet-LLL_glrlm_LongRunHighGrayLevelEmphasis** | $1.10\times{10}^{4}$ ($1.06\times{10}^{4}$) | $1.30\times{10}^{4}$ ($1.03\times{10}^{4}$) | 0.1359 |
| **wavelet-LLL_glrlm_LongRunLowGrayLevelEmphasis** | 0.0061 (0.0107) | 0.0029 (0.0081) | 0.0047 |
| **wavelet-LLL_glrlm_LowGrayLevelRunEmphasis** | 0.0024 (0.0054) | 0.0010 (0.0022) | 0.0003 |
| **wavelet-LLL_glrlm_RunEntropy** | 4.9373 (0.3548) | 5.0461 (0.2780) | 0.0042 |
| **wavelet-LLL_glrlm_RunLengthNonUniformity** | $4.13\times{10}^{4}$ ($6.74\times{10}^{4}$) | $2.24\times{10}^{4}$ ($2.97\times{10}^{4}$) | 0.0003 |
| **wavelet-LLL_glrlm_RunLengthNonUniformityNormalized** | 0.5227 (0.1036) | 0.5394 (0.0854) | 0.1442 |
| **wavelet-LLL_glrlm_RunPercentage** | 0.6477 (0.0931) | 0.6640 (0.0773) | 0.1156 |
| **wavelet-LLL_glrlm_RunVariance** | 1.5671 (1.0353) | 1.3249 (0.7740) | 0.0238 |
| **wavelet-LLL_glrlm_ShortRunEmphasis** | 0.7421 (0.0742) | 0.7569 (0.0593) | 0.0647 |
| **wavelet-LLL_glrlm_ShortRunHighGrayLevelEmphasis** | 1832.3365 (1498.3559) | 2508.0853 (1736.3779) | 0.0019 |
| **wavelet-LLL_glrlm_ShortRunLowGrayLevelEmphasis** | 0.0021 (0.0048) | 0.0008 (0.0018) | 0.0003 |
| **wavelet-LLL_glszm_GrayLevelNonUniformity** | 154.8628 (250.3286) | 98.0617 (91.1841) | 0.0016 |
| **wavelet-LLL_glszm_GrayLevelNonUniformityNormalized** | 0.0481 (0.0209) | 0.0403 (0.0142) | 0.0001 |
| **wavelet-LLL_glszm_GrayLevelVariance** | 87.9648 (68.0888) | 130.0344 (155.3385) | 0.021 |
| **wavelet-LLL_glszm_HighGrayLevelZoneEmphasis** | 2129.4515 (1704.7681) | 2782.6669 (1953.4227) | 0.0077 |
| **wavelet-LLL_glszm_LargeAreaEmphasis** | $1.38\times{10}^{6}$ ($3.52\times{10}^{6}$) | $3.66\times{10}^{5}$ ($1.19\times{10}^{6}$) | <0.0001 |
| **wavelet-LLL_glszm_LargeAreaHighGrayLevelEmphasis** | $3.75\times{10}^{9}$ ($1.23\times{10}^{10}$) | $1.20\times{10}^{9}$ ($3.22\times{10}^{9}$) | 0.0016 |
| **wavelet-LLL_glszm_LargeAreaLowGrayLevelEmphasis** | 722.8140 (2028.4695) | 232.3128 (1201.2432) | 0.0069 |
| **wavelet-LLL_glszm_LowGrayLevelZoneEmphasis** | 0.0062 (0.0160) | 0.0025 (0.0043) | 0.0005 |
| **wavelet-LLL_glszm_SizeZoneNonUniformity** | 982.9184 (1068.0894) | 965.9573 (952.8069) | 0.8922 |
| **wavelet-LLL_glszm_SizeZoneNonUniformityNormalized** | 0.3038 (0.0810) | 0.3445 (0.0769) | 0.0001 |
| **wavelet-LLL_glszm_SmallAreaEmphasis** | 0.5628 (0.0813) | 0.6034 (0.0741) | <0.0001 |
| **wavelet-LLL_glszm_SmallAreaHighGrayLevelEmphasis** | 1172.5516 (1016.1862) | 1634.8287 (1216.3355) | 0.0023 |
| **wavelet-LLL_glszm_SmallAreaLowGrayLevelEmphasis** | 0.0040 (0.0101) | 0.0019 (0.0025) | 0.001 |
| **wavelet-LLL_glszm_ZoneEntropy** | 7.1182 (0.7002) | 7.2058 (0.5002) | 0.2115 |
| **wavelet-LLL_glszm_ZonePercentage** | 0.0553 (0.0430) | 0.0650 (0.0434) | 0.0822 |
| **wavelet-LLL_glszm_ZoneVariance** | $1.38\times{10}^{6}$ ($3.52\times{10}^{6}$) | $3.65\times{10}^{5}$ ($1.19\times{10}^{6}$) | <0.0001 |
| **wavelet-LLL_ngtdm_Busyness** | 1.8775 (3.2885) | 0.9050 (3.2280) | 0.0195 |
| **wavelet-LLL_ngtdm_Coarseness** | 0.0026 (0.0057) | 0.0013 (0.0026) | 0.0047 |
| **wavelet-LLL_ngtdm_Complexity** | 1421.5996 (1237.2495) | 2410.9004 (3103.6312) | 0.0065 |
| **wavelet-LLL_ngtdm_Contrast** | 0.0139 (0.0227) | 0.0105 (0.0136) | 0.0996 |
| **wavelet-LLL_ngtdm_Strength** | 4.2053 (5.9110) | 5.9090 (8.0302) | 0.0823 |
