# Supplementary material for: Clinical radiomics-based machine learning versus three-dimension convolutional neural network analysis for differentiation of thymic epithelial tumors from other prevascular mediastinal tumors on chest computed tomography scan
Source: Front Oncol. 2023 Apr 18;13:1105100. doi: 10.3389/fonc.2023.1105100 (PMC10151670; doi:10.3389/fonc.2023.1105100)
Supplement: Supplementary file 3 [file Table_3.docx]

**Supplementary Table 3. Top 20 feature selection with Lasso Logistic Regression in UECT**

| **Selection Group** | **Lambda** | **Selected Variables** | | |
| --- | --- | --- | --- | --- |
| Selection_1 | 0.04863 | original_firstorder_Median | wavelet-LLH_glcm_MCC | wavelet-LLH_glrlm_RunEntropy |
|  |  | wavelet-LLH_glszm_LargeAreaLowGrayLevelEmphasis | wavelet-HLH_ngtdm_Busyness | wavelet-LLL_firstorder_RootMeanSquared |
|  |  | wavelet-LLL_glszm_LargeAreaEmphasis |  |  |
| Selection_2 | 0.03511 | original_firstorder_Median | original_glrlm_RunEntropy | wavelet-LLH_glcm_Correlation |
|  |  | wavelet-LLH_glcm_MCC | wavelet-LLH_glrlm_RunEntropy | wavelet-LLH_glrlm_ShortRunLowGrayLevelEmphasis |
|  |  | wavelet-LLH_glszm_LargeAreaLowGrayLevelEmphasis | wavelet-LHL_glszm_SmallAreaEmphasis | wavelet-LHH_glrlm_RunEntropy |
|  |  | wavelet-HLH_ngtdm_Busyness | wavelet-HHL_glszm_GrayLevelNonUniformityNormalized | wavelet-LLL_firstorder_RootMeanSquared |
|  |  | wavelet-LLL_glszm_LargeAreaEmphasis |  |  |
| Selection_3 | 0.02535 | original_shape_Sphericity | original_firstorder_Median | original_glrlm_RunEntropy |
|  |  | wavelet-LLH_glcm_Correlation | wavelet-LLH_glcm_MCC | wavelet-LLH_glrlm_RunEntropy |
|  |  | wavelet-LLH_glrlm_ShortRunLowGrayLevelEmphasis | wavelet-LLH_glszm_LargeAreaLowGrayLevelEmphasis | wavelet-LHL_firstorder_Skewness |
|  |  | wavelet-LHL_glszm_SmallAreaEmphasis | wavelet-LHH_glcm_ClusterShade | wavelet-LHH_glrlm_RunEntropy |
|  |  | wavelet-HLH_ngtdm_Busyness | wavelet-HHL_glszm_GrayLevelNonUniformityNormalized | wavelet-HHH_glszm_SizeZoneNonUniformityNormalized |
|  |  | wavelet-HHH_glszm_SmallAreaEmphasis | wavelet-LLL_firstorder_RootMeanSquared | wavelet-LLL_glszm_LargeAreaEmphasis |
| Selection_4 | 0.01831 | original_shape_Sphericity | original_firstorder_Median | original_glrlm_RunEntropy |
|  |  | wavelet-LLH_glcm_Correlation | wavelet-LLH_glcm_MCC | wavelet-LLH_glrlm_RunEntropy |
|  |  | wavelet-LLH_glrlm_ShortRunLowGrayLevelEmphasis | wavelet-LLH_glszm_LowGrayLevelZoneEmphasis | wavelet-LHL_glszm_SmallAreaEmphasis |
|  |  | wavelet-LHH_firstorder_Mean | wavelet-LHH_glcm_MaximumProbability | wavelet-LHH_glrlm_RunEntropy |
|  |  | wavelet-LHH_ngtdm_Busyness | wavelet-HLL_glszm_ZoneVariance | wavelet-HLH_glcm_MCC |
|  |  | wavelet-HLH_glszm_SmallAreaLowGrayLevelEmphasis | wavelet-HLH_ngtdm_Busyness | wavelet-HHL_glszm_GrayLevelNonUniformityNormalized |
|  |  | wavelet-HHH_glszm_SizeZoneNonUniformityNormalized | wavelet-HHH_glszm_SmallAreaEmphasis | wavelet-LLL_firstorder_RootMeanSquared |
| Selection_5 | 0.01322 | original_shape_Sphericity | original_shape_SurfaceVolumeRatio | original_firstorder_Median |
|  |  | original_firstorder_c | original_glrlm_RunEntropy | wavelet-LLH_firstorder_Kurtosis |
|  |  | wavelet-LLH_glcm_Correlation | wavelet-LLH_glcm_MCC | wavelet-LLH_glcm_MaximumProbability |
|  |  | wavelet-LLH_glrlm_ShortRunLowGrayLevelEmphasis | wavelet-LLH_glszm_LowGrayLevelZoneEmphasis | wavelet-LHL_firstorder_Skewness |
|  |  | wavelet-LHL_glszm_SmallAreaEmphasis | wavelet-LHH_firstorder_Mean | wavelet-LHH_glcm_MaximumProbability |
|  |  | wavelet-LHH_glrlm_RunEntropy | wavelet-LHH_glszm_SizeZoneNonUniformityNormalized | wavelet-LHH_ngtdm_Busyness |
|  |  | wavelet-HLL_firstorder_Maximum | wavelet-HLL_glszm_ZoneVariance | wavelet-HLH_glcm_MCC |
|  |  | wavelet-HLH_glcm_MaximumProbability | wavelet-HLH_glszm_SmallAreaLowGrayLevelEmphasis | wavelet-HLH_ngtdm_Busyness |
|  |  | wavelet-HHL_glcm_Idmn | wavelet-HHL_glszm_GrayLevelNonUniformityNormalized | wavelet-HHH_glrlm_ShortRunLowGrayLevelEmphasis |
|  |  | wavelet-HHH_glszm_SizeZoneNonUniformityNormalized | wavelet-HHH_glszm_SmallAreaEmphasis | wavelet-LLL_firstorder_RootMeanSquared |
|  |  | wavelet-LLL_glcm_MaximumProbability |  |  |
| Selection_6 | 0.00955 | original_shape_Sphericity | original_shape_SurfaceVolumeRatio | original_firstorder_Median |
|  |  | original_firstorder_Skewness | wavelet-LLH_firstorder_Kurtosis | wavelet-LLH_glcm_MCC |
|  |  | wavelet-LLH_glcm_MaximumProbability | wavelet-LLH_glrlm_LongRunHighGrayLevelEmphasis | wavelet-LLH_glrlm_ShortRunLowGrayLevelEmphasis |
|  |  | wavelet-LLH_glszm_LowGrayLevelZoneEmphasis | wavelet-LHL_firstorder_Mean | wavelet-LHL_firstorder_Skewness |
|  |  | wavelet-LHL_glcm_Idmn | wavelet-LHL_glszm_SmallAreaEmphasis | wavelet-LHH_firstorder_Kurtosis |
|  |  | wavelet-LHH_firstorder_Mean | wavelet-LHH_glcm_ClusterShade | wavelet-LHH_glcm_MaximumProbability |
|  |  | wavelet-LHH_glrlm_RunEntropy | wavelet-LHH_glszm_SizeZoneNonUniformityNormalized | wavelet-LHH_ngtdm_Busyness |
|  |  | wavelet-HLL_firstorder_Maximum | wavelet-HLL_firstorder_Skewness | wavelet-HLL_glszm_ZoneVariance |
|  |  | wavelet-HLL_ngtdm_Strength | wavelet-HLH_firstorder_Kurtosis | wavelet-HLH_glcm_MCC |
|  |  | wavelet-HLH_glcm_MaximumProbability | wavelet-HLH_glszm_SmallAreaLowGrayLevelEmphasis | wavelet-HLH_glszm_ZoneEntropy |
|  |  | wavelet-HLH_ngtdm_Busyness | wavelet-HHL_firstorder_Skewness | wavelet-HHL_glcm_Idmn |
|  |  | wavelet-HHL_glcm_Imc1 | wavelet-HHL_glszm_GrayLevelNonUniformityNormalized | wavelet-HHH_glrlm_ShortRunLowGrayLevelEmphasis |
|  |  | wavelet-HHH_glszm_SizeZoneNonUniformityNormalized | wavelet-HHH_glszm_SmallAreaEmphasis | wavelet-LLL_firstorder_RootMeanSquared |
|  |  | wavelet-LLL_glcm_Imc1 | wavelet-LLL_glcm_MaximumProbability | wavelet-LLL_gldm_LargeDependenceLowGrayLevelEmphasis |
| Selection_7 | 0.00689 | original_shape_Flatness | original_shape_Sphericity | original_shape_SurfaceVolumeRatio |
|  |  | original_firstorder_Kurtosis | original_firstorder_Median | original_firstorder_Skewness |
|  |  | original_glcm_InverseVariance | original_glcm_MCC | original_gldm_LargeDependenceLowGrayLevelEmphasis |
|  |  | wavelet-LLH_firstorder_Kurtosis | wavelet-LLH_glcm_MCC | wavelet-LLH_glcm_MaximumProbability |
|  |  | wavelet-LLH_glrlm_LongRunHighGrayLevelEmphasis | wavelet-LLH_glrlm_LowGrayLevelRunEmphasis | wavelet-LLH_glszm_LowGrayLevelZoneEmphasis |
|  |  | wavelet-LLH_ngtdm_Busyness | wavelet-LHL_firstorder_Mean | wavelet-LHL_firstorder_Skewness |
|  |  | wavelet-LHL_glcm_Idmn | wavelet-LHL_glcm_Idn | wavelet-LHL_glszm_GrayLevelNonUniformityNormalized |
|  |  | wavelet-LHL_glszm_SmallAreaEmphasis | wavelet-LHH_firstorder_Kurtosis | wavelet-LHH_firstorder_Mean |
|  |  | wavelet-LHH_glcm_ClusterShade | wavelet-LHH_glcm_MaximumProbability | wavelet-LHH_glrlm_RunEntropy |
|  |  | wavelet-LHH_glszm_SizeZoneNonUniformityNormalized | wavelet-LHH_ngtdm_Busyness | wavelet-HLL_firstorder_Maximum |
|  |  | wavelet-HLL_firstorder_Skewness | wavelet-HLL_glcm_Correlation | wavelet-HLL_glszm_GrayLevelVariance |
|  |  | wavelet-HLL_glszm_LargeAreaEmphasis | wavelet-HLL_glszm_ZoneVariance | wavelet-HLL_ngtdm_Strength |
|  |  | wavelet-HLH_firstorder_Kurtosis | wavelet-HLH_glcm_InverseVariance | wavelet-HLH_glcm_MCC |
|  |  | wavelet-HLH_glcm_MaximumProbability | wavelet-HLH_glszm_SmallAreaLowGrayLevelEmphasis | wavelet-HLH_glszm_ZoneEntropy |
|  |  | wavelet-HLH_ngtdm_Busyness | wavelet-HHL_firstorder_Skewness | wavelet-HHL_glcm_Idmn |
|  |  | wavelet-HHL_glcm_Imc1 | wavelet-HHL_glcm_MCC | wavelet-HHL_glszm_GrayLevelNonUniformityNormalized |
|  |  | wavelet-HHL_glszm_LargeAreaEmphasis | wavelet-HHH_glrlm_RunEntropy | wavelet-HHH_glrlm_ShortRunLowGrayLevelEmphasis |
|  |  | wavelet-HHH_glszm_SizeZoneNonUniformityNormalized | wavelet-HHH_glszm_SmallAreaEmphasis | wavelet-LLL_firstorder_RootMeanSquared |
|  |  | wavelet-LLL_glcm_Imc1 | wavelet-LLL_glcm_MaximumProbability |  |
| Selection_8 | 0.00498 | original_shape_Flatness | original_shape_Sphericity | original_shape_SurfaceArea |
|  |  | original_shape_SurfaceVolumeRatio | original_firstorder_Kurtosis | original_firstorder_Mean |
|  |  | original_firstorder_Median | original_firstorder_Skewness | original_glcm_InverseVariance |
|  |  | original_glcm_MCC | wavelet-LLH_firstorder_Kurtosis | wavelet-LLH_glcm_MCC |
|  |  | wavelet-LLH_glcm_MaximumProbability | wavelet-LLH_glrlm_LongRunHighGrayLevelEmphasis | wavelet-LLH_glrlm_LongRunLowGrayLevelEmphasis |
|  |  | wavelet-LLH_glrlm_ShortRunEmphasis | wavelet-LLH_glszm_LowGrayLevelZoneEmphasis | wavelet-LLH_ngtdm_Busyness |
|  |  | wavelet-LLH_ngtdm_Contrast | wavelet-LHL_firstorder_Mean | wavelet-LHL_firstorder_Skewness |
|  |  | wavelet-LHL_glcm_Idmn | wavelet-LHL_glcm_Idn | wavelet-LHL_glszm_GrayLevelNonUniformityNormalized |
|  |  | wavelet-LHH_firstorder_Kurtosis | wavelet-LHH_firstorder_Mean | wavelet-LHH_glcm_ClusterShade |
|  |  | wavelet-LHH_glcm_MaximumProbability | wavelet-LHH_glrlm_RunEntropy | wavelet-LHH_glszm_SizeZoneNonUniformityNormalized |
|  |  | wavelet-LHH_ngtdm_Busyness | wavelet-HLL_firstorder_Maximum | wavelet-HLL_firstorder_Skewness |
|  |  | wavelet-HLL_glcm_Correlation | wavelet-HLL_glcm_Idn | wavelet-HLL_glszm_GrayLevelVariance |
|  |  | wavelet-HLL_glszm_LargeAreaEmphasis | wavelet-HLL_glszm_ZoneVariance | wavelet-HLL_ngtdm_Strength |
|  |  | wavelet-HLH_firstorder_Kurtosis | wavelet-HLH_glcm_InverseVariance | wavelet-HLH_glcm_MCC |
|  |  | wavelet-HLH_glcm_MaximumProbability | wavelet-HLH_glszm_SmallAreaLowGrayLevelEmphasis | wavelet-HLH_glszm_ZoneEntropy |
|  |  | wavelet-HLH_ngtdm_Busyness | wavelet-HHL_firstorder_Skewness | wavelet-HHL_glcm_Idmn |
|  |  | wavelet-HHL_glcm_Imc1 | wavelet-HHL_glcm_MCC | wavelet-HHL_glszm_GrayLevelNonUniformityNormalized |
|  |  | wavelet-HHL_glszm_LargeAreaEmphasis | wavelet-HHH_glrlm_RunEntropy | wavelet-HHH_glrlm_ShortRunLowGrayLevelEmphasis |
|  |  | wavelet-HHH_glszm_GrayLevelNonUniformityNormalized | wavelet-HHH_glszm_SizeZoneNonUniformityNormalized | wavelet-HHH_glszm_SmallAreaEmphasis |
|  |  | wavelet-LLL_firstorder_Median | wavelet-LLL_firstorder_RootMeanSquared | wavelet-LLL_glcm_Correlation |
|  |  | wavelet-LLL_glcm_Imc1 | wavelet-LLL_glcm_MaximumProbability |  |
| Selection_9 | 0.00359 | original_shape_Elongation | original_shape_Flatness | original_shape_MinorAxisLength |
|  |  | original_shape_Sphericity | original_shape_SurfaceArea | original_shape_SurfaceVolumeRatio |
|  |  | original_firstorder_Kurtosis | original_firstorder_Mean | original_firstorder_Median |
|  |  | original_firstorder_Skewness | original_glcm_InverseVariance | original_glcm_JointAverage |
|  |  | original_glcm_MCC | original_glcm_SumAverage | original_ngtdm_Strength |
|  |  | wavelet-LLH_firstorder_Kurtosis | wavelet-LLH_glcm_MCC | wavelet-LLH_glcm_MaximumProbability |
|  |  | wavelet-LLH_glrlm_LongRunEmphasis | wavelet-LLH_glrlm_LongRunHighGrayLevelEmphasis | wavelet-LLH_glrlm_LongRunLowGrayLevelEmphasis |
|  |  | wavelet-LLH_glrlm_ShortRunEmphasis | wavelet-LLH_glszm_LowGrayLevelZoneEmphasis | wavelet-LLH_glszm_SmallAreaLowGrayLevelEmphasis |
|  |  | wavelet-LLH_ngtdm_Busyness | wavelet-LLH_ngtdm_Contrast | wavelet-LHL_firstorder_Maximum |
|  |  | wavelet-LHL_firstorder_Mean | wavelet-LHL_firstorder_Skewness | wavelet-LHL_glcm_Idmn |
|  |  | wavelet-LHL_glcm_Idn | wavelet-LHL_glszm_GrayLevelNonUniformityNormalized | wavelet-LHH_firstorder_Kurtosis |
|  |  | wavelet-LHH_firstorder_Mean | wavelet-LHH_glcm_ClusterShade | wavelet-LHH_glcm_MaximumProbability |
|  |  | wavelet-LHH_glrlm_RunEntropy | wavelet-LHH_glszm_SizeZoneNonUniformityNormalized | wavelet-LHH_ngtdm_Busyness |
|  |  | wavelet-LHH_ngtdm_Contrast | wavelet-HLL_firstorder_Kurtosis | wavelet-HLL_firstorder_Maximum |
|  |  | wavelet-HLL_firstorder_Skewness | wavelet-HLL_glcm_Correlation | wavelet-HLL_glcm_Idn |
|  |  | wavelet-HLL_glrlm_LongRunLowGrayLevelEmphasis | wavelet-HLL_glrlm_RunVariance | wavelet-HLL_glszm_GrayLevelNonUniformityNormalized |
|  |  | wavelet-HLL_glszm_GrayLevelVariance | wavelet-HLL_glszm_LargeAreaEmphasis | wavelet-HLL_glszm_ZoneVariance |
|  |  | wavelet-HLL_ngtdm_Strength | wavelet-HLH_firstorder_Kurtosis | wavelet-HLH_glcm_InverseVariance |
|  |  | wavelet-HLH_glcm_MCC | wavelet-HLH_glcm_MaximumProbability | wavelet-HLH_glszm_SmallAreaLowGrayLevelEmphasis |
|  |  | wavelet-HLH_glszm_ZoneEntropy | wavelet-HLH_ngtdm_Busyness | wavelet-HHL_glcm_Idmn |
|  |  | wavelet-HHL_glcm_Imc1 | wavelet-HHL_glcm_MCC | wavelet-HHL_glszm_GrayLevelNonUniformityNormalized |
|  |  | wavelet-HHL_glszm_LargeAreaEmphasis | wavelet-HHL_glszm_SizeZoneNonUniformityNormalized | wavelet-HHL_glszm_ZoneVariance |
|  |  | wavelet-HHL_ngtdm_Contrast | wavelet-HHH_firstorder_Skewness | wavelet-HHH_glrlm_RunEntropy |
|  |  | wavelet-HHH_glrlm_ShortRunLowGrayLevelEmphasis | wavelet-HHH_glszm_GrayLevelNonUniformityNormalized | wavelet-HHH_glszm_SizeZoneNonUniformityNormalized |
|  |  | wavelet-HHH_glszm_SmallAreaEmphasis | wavelet-LLL_firstorder_Median | wavelet-LLL_firstorder_RootMeanSquared |
|  |  | wavelet-LLL_glcm_Correlation | wavelet-LLL_glcm_Imc1 | wavelet-LLL_glcm_MCC |
|  |  | wavelet-LLL_glcm_MaximumProbability | wavelet-LLL_glszm_SizeZoneNonUniformityNormalized |  |
| Selection_10 | 0.00260 | original_shape_Flatness | original_shape_MinorAxisLength | original_shape_Sphericity |
|  |  | original_shape_SurfaceArea | original_shape_SurfaceVolumeRatio | original_firstorder_Kurtosis |
|  |  | original_firstorder_Median | original_firstorder_Skewness | original_glcm_Correlation |
|  |  | original_glcm_InverseVariance | original_glcm_JointAverage | original_glcm_MCC |
|  |  | original_glcm_SumAverage | original_ngtdm_Contrast | original_ngtdm_Strength |
|  |  | wavelet-LLH_firstorder_Kurtosis | wavelet-LLH_firstorder_RootMeanSquared | wavelet-LLH_glcm_MCC |
|  |  | wavelet-LLH_glcm_MaximumProbability | wavelet-LLH_glrlm_LongRunEmphasis | wavelet-LLH_glrlm_LongRunHighGrayLevelEmphasis |
|  |  | wavelet-LLH_glrlm_LongRunLowGrayLevelEmphasis | wavelet-LLH_glrlm_ShortRunEmphasis | wavelet-LLH_glszm_LowGrayLevelZoneEmphasis |
|  |  | wavelet-LLH_glszm_SmallAreaLowGrayLevelEmphasis | wavelet-LLH_ngtdm_Busyness | wavelet-LLH_ngtdm_Contrast |
|  |  | wavelet-LHL_firstorder_Maximum | wavelet-LHL_firstorder_Mean | wavelet-LHL_firstorder_Skewness |
|  |  | wavelet-LHL_glcm_Idmn | wavelet-LHL_glcm_Idn | wavelet-LHL_glszm_GrayLevelNonUniformityNormalized |
|  |  | wavelet-LHL_glszm_LargeAreaHighGrayLevelEmphasis | wavelet-LHH_firstorder_Kurtosis | wavelet-LHH_firstorder_Mean |
|  |  | wavelet-LHH_glcm_ClusterShade | wavelet-LHH_glcm_Idmn | wavelet-LHH_glcm_MaximumProbability |
|  |  | wavelet-LHH_glrlm_RunEntropy | wavelet-LHH_glszm_GrayLevelNonUniformityNormalized | wavelet-LHH_glszm_SizeZoneNonUniformityNormalized |
|  |  | wavelet-LHH_ngtdm_Busyness | wavelet-LHH_ngtdm_Contrast | wavelet-HLL_firstorder_Kurtosis |
|  |  | wavelet-HLL_firstorder_Maximum | wavelet-HLL_firstorder_Skewness | wavelet-HLL_glcm_Correlation |
|  |  | wavelet-HLL_glcm_Idn | wavelet-HLL_glcm_Imc2 | wavelet-HLL_glrlm_LongRunLowGrayLevelEmphasis |
|  |  | wavelet-HLL_glrlm_RunVariance | wavelet-HLL_glszm_GrayLevelNonUniformityNormalized | wavelet-HLL_glszm_GrayLevelVariance |
|  |  | wavelet-HLL_glszm_LargeAreaEmphasis | wavelet-HLL_glszm_ZoneEntropy | wavelet-HLL_glszm_ZoneVariance |
|  |  | wavelet-HLL_ngtdm_Strength | wavelet-HLH_firstorder_Kurtosis | wavelet-HLH_glcm_InverseVariance |
|  |  | wavelet-HLH_glcm_MCC | wavelet-HLH_glcm_MaximumProbability | wavelet-HLH_glszm_SmallAreaLowGrayLevelEmphasis |
|  |  | wavelet-HLH_glszm_ZoneEntropy | wavelet-HLH_ngtdm_Busyness | wavelet-HHL_glcm_Correlation |
|  |  | wavelet-HHL_glcm_Idmn | wavelet-HHL_glcm_Imc1 | wavelet-HHL_glcm_MCC |
|  |  | wavelet-HHL_glrlm_ShortRunLowGrayLevelEmphasis | wavelet-HHL_glszm_GrayLevelNonUniformityNormalized | wavelet-HHL_glszm_LowGrayLevelZoneEmphasis |
|  |  | wavelet-HHL_glszm_SizeZoneNonUniformityNormalized | wavelet-HHL_glszm_ZoneVariance | wavelet-HHL_ngtdm_Contrast |
|  |  | wavelet-HHH_firstorder_Skewness | wavelet-HHH_glrlm_RunEntropy | wavelet-HHH_glrlm_ShortRunLowGrayLevelEmphasis |
|  |  | wavelet-HHH_glszm_GrayLevelNonUniformityNormalized | wavelet-HHH_glszm_SizeZoneNonUniformityNormalized | wavelet-HHH_glszm_SmallAreaEmphasis |
|  |  | wavelet-HHH_ngtdm_Contrast | wavelet-LLL_firstorder_Median | wavelet-LLL_glcm_Correlation |
|  |  | wavelet-LLL_glcm_Imc1 | wavelet-LLL_glcm_MaximumProbability | wavelet-LLL_glszm_LowGrayLevelZoneEmphasis |
| Selection_11 | 0.00187 | original_shape_Flatness | original_shape_Maximum2DDiameterSlice | original_shape_MinorAxisLength |
|  |  | original_shape_Sphericity | original_shape_SurfaceArea | original_shape_SurfaceVolumeRatio |
|  |  | original_firstorder_Kurtosis | original_firstorder_Skewness | original_glcm_Correlation |
|  |  | original_glcm_Idn | original_glcm_InverseVariance | original_glcm_JointAverage |
|  |  | original_glcm_MCC | original_glcm_SumAverage | original_gldm_LargeDependenceLowGrayLevelEmphasis |
|  |  | original_ngtdm_Contrast | original_ngtdm_Strength | wavelet-LLH_firstorder_10Percentile |
|  |  | wavelet-LLH_firstorder_Kurtosis | wavelet-LLH_firstorder_RootMeanSquared | wavelet-LLH_firstorder_Skewness |
|  |  | wavelet-LLH_glcm_MCC | wavelet-LLH_glcm_MaximumProbability | wavelet-LLH_gldm_LargeDependenceLowGrayLevelEmphasis |
|  |  | wavelet-LLH_glrlm_LongRunEmphasis | wavelet-LLH_glrlm_LongRunHighGrayLevelEmphasis | wavelet-LLH_glrlm_LongRunLowGrayLevelEmphasis |
|  |  | wavelet-LLH_glrlm_ShortRunEmphasis | wavelet-LLH_glszm_LowGrayLevelZoneEmphasis | wavelet-LLH_glszm_SizeZoneNonUniformity |
|  |  | wavelet-LLH_glszm_SizeZoneNonUniformityNormalized | wavelet-LLH_glszm_SmallAreaLowGrayLevelEmphasis | wavelet-LLH_glszm_ZoneEntropy |
|  |  | wavelet-LLH_ngtdm_Busyness | wavelet-LLH_ngtdm_Contrast | wavelet-LHL_firstorder_Maximum |
|  |  | wavelet-LHL_firstorder_Mean | wavelet-LHL_firstorder_Skewness | wavelet-LHL_glcm_Correlation |
|  |  | wavelet-LHL_glcm_Idmn | wavelet-LHL_glcm_Idn | wavelet-LHL_gldm_DependenceEntropy |
|  |  | wavelet-LHL_glszm_GrayLevelNonUniformityNormalized | wavelet-LHL_glszm_LargeAreaHighGrayLevelEmphasis | wavelet-LHH_firstorder_Kurtosis |
|  |  | wavelet-LHH_firstorder_Mean | wavelet-LHH_glcm_ClusterShade | wavelet-LHH_glcm_Idmn |
|  |  | wavelet-LHH_glcm_MaximumProbability | wavelet-LHH_glrlm_RunEntropy | wavelet-LHH_glszm_GrayLevelNonUniformityNormalized |
|  |  | wavelet-LHH_glszm_LowGrayLevelZoneEmphasis | wavelet-LHH_glszm_SizeZoneNonUniformityNormalized | wavelet-LHH_ngtdm_Contrast |
|  |  | wavelet-HLL_firstorder_Kurtosis | wavelet-HLL_firstorder_Maximum | wavelet-HLL_firstorder_Skewness |
|  |  | wavelet-HLL_glcm_Correlation | wavelet-HLL_glcm_Idn | wavelet-HLL_glcm_Imc2 |
|  |  | wavelet-HLL_glrlm_LongRunLowGrayLevelEmphasis | wavelet-HLL_glrlm_RunVariance | wavelet-HLL_glszm_GrayLevelNonUniformityNormalized |
|  |  | wavelet-HLL_glszm_GrayLevelVariance | wavelet-HLL_glszm_LargeAreaEmphasis | wavelet-HLL_glszm_SmallAreaLowGrayLevelEmphasis |
|  |  | wavelet-HLL_glszm_ZoneEntropy | wavelet-HLL_glszm_ZoneVariance | wavelet-HLL_ngtdm_Strength |
|  |  | wavelet-HLH_firstorder_Kurtosis | wavelet-HLH_glcm_InverseVariance | wavelet-HLH_glcm_MCC |
|  |  | wavelet-HLH_glcm_MaximumProbability | wavelet-HLH_glszm_SmallAreaEmphasis | wavelet-HLH_glszm_SmallAreaLowGrayLevelEmphasis |
|  |  | wavelet-HLH_glszm_ZoneEntropy | wavelet-HHL_glcm_Correlation | wavelet-HHL_glcm_Idmn |
|  |  | wavelet-HHL_glcm_Imc1 | wavelet-HHL_glcm_MCC | wavelet-HHL_gldm_LargeDependenceHighGrayLevelEmphasis |
|  |  | wavelet-HHL_glrlm_ShortRunLowGrayLevelEmphasis | wavelet-HHL_glszm_GrayLevelNonUniformityNormalized | wavelet-HHL_glszm_LowGrayLevelZoneEmphasis |
|  |  | wavelet-HHL_glszm_SizeZoneNonUniformityNormalized | wavelet-HHL_glszm_SmallAreaLowGrayLevelEmphasis | wavelet-HHL_glszm_ZoneEntropy |
|  |  | wavelet-HHL_glszm_ZoneVariance | wavelet-HHL_ngtdm_Busyness | wavelet-HHL_ngtdm_Contrast |
|  |  | wavelet-HHH_firstorder_Skewness | wavelet-HHH_glrlm_RunEntropy | wavelet-HHH_glrlm_ShortRunLowGrayLevelEmphasis |
|  |  | wavelet-HHH_glszm_GrayLevelNonUniformityNormalized | wavelet-HHH_glszm_SizeZoneNonUniformityNormalized | wavelet-HHH_glszm_SmallAreaEmphasis |
|  |  | wavelet-HHH_ngtdm_Contrast | wavelet-LLL_firstorder_10Percentile | wavelet-LLL_firstorder_Median |
|  |  | wavelet-LLL_glcm_Correlation | wavelet-LLL_glcm_Imc1 | wavelet-LLL_glcm_MaximumProbability |
|  |  | wavelet-LLL_glrlm_ShortRunLowGrayLevelEmphasis | wavelet-LLL_glszm_GrayLevelVariance | wavelet-LLL_glszm_LowGrayLevelZoneEmphasis |
| Selection_12 | 0.00135 | original_shape_Flatness | original_shape_Maximum2DDiameterSlice | original_shape_Maximum3DDiameter |
|  |  | original_shape_MinorAxisLength | original_shape_Sphericity | original_shape_SurfaceArea |
|  |  | original_shape_SurfaceVolumeRatio | original_firstorder_Kurtosis | original_firstorder_Skewness |
|  |  | original_glcm_Correlation | original_glcm_Idn | original_glcm_InverseVariance |
|  |  | original_glcm_JointAverage | original_glcm_MCC | original_glcm_SumAverage |
|  |  | original_glszm_SmallAreaEmphasis | original_ngtdm_Contrast | original_ngtdm_Strength |
|  |  | wavelet-LLH_firstorder_10Percentile | wavelet-LLH_firstorder_Kurtosis | wavelet-LLH_firstorder_RootMeanSquared |
|  |  | wavelet-LLH_firstorder_Skewness | wavelet-LLH_glcm_JointAverage | wavelet-LLH_glcm_MCC |
|  |  | wavelet-LLH_glcm_MaximumProbability | wavelet-LLH_glcm_SumAverage | wavelet-LLH_gldm_LargeDependenceLowGrayLevelEmphasis |
|  |  | wavelet-LLH_glrlm_LongRunEmphasis | wavelet-LLH_glrlm_LongRunHighGrayLevelEmphasis | wavelet-LLH_glrlm_ShortRunEmphasis |
|  |  | wavelet-LLH_glszm_SizeZoneNonUniformity | wavelet-LLH_glszm_SizeZoneNonUniformityNormalized | wavelet-LLH_glszm_SmallAreaLowGrayLevelEmphasis |
|  |  | wavelet-LLH_glszm_ZoneEntropy | wavelet-LLH_ngtdm_Busyness | wavelet-LLH_ngtdm_Contrast |
|  |  | wavelet-LHL_firstorder_Maximum | wavelet-LHL_firstorder_Mean | wavelet-LHL_firstorder_Skewness |
|  |  | wavelet-LHL_glcm_Correlation | wavelet-LHL_glcm_Idmn | wavelet-LHL_glcm_Idn |
|  |  | wavelet-LHL_gldm_DependenceEntropy | wavelet-LHL_glszm_GrayLevelNonUniformityNormalized | wavelet-LHL_glszm_LargeAreaHighGrayLevelEmphasis |
|  |  | wavelet-LHL_glszm_SizeZoneNonUniformityNormalized | wavelet-LHH_firstorder_Kurtosis | wavelet-LHH_firstorder_Mean |
|  |  | wavelet-LHH_glcm_ClusterShade | wavelet-LHH_glcm_Idmn | wavelet-LHH_glcm_MCC |
|  |  | wavelet-LHH_glcm_MaximumProbability | wavelet-LHH_glrlm_RunEntropy | wavelet-LHH_glszm_GrayLevelNonUniformityNormalized |
|  |  | wavelet-LHH_glszm_LowGrayLevelZoneEmphasis | wavelet-LHH_glszm_SizeZoneNonUniformityNormalized | wavelet-LHH_ngtdm_Contrast |
|  |  | wavelet-HLL_firstorder_Kurtosis | wavelet-HLL_firstorder_Maximum | wavelet-HLL_firstorder_Skewness |
|  |  | wavelet-HLL_glcm_Correlation | wavelet-HLL_glcm_Idn | wavelet-HLL_glcm_Imc2 |
|  |  | wavelet-HLL_glrlm_LongRunLowGrayLevelEmphasis | wavelet-HLL_glrlm_RunVariance | wavelet-HLL_glszm_GrayLevelNonUniformity |
|  |  | wavelet-HLL_glszm_GrayLevelNonUniformityNormalized | wavelet-HLL_glszm_GrayLevelVariance | wavelet-HLL_glszm_LargeAreaEmphasis |
|  |  | wavelet-HLL_glszm_SmallAreaEmphasis | wavelet-HLL_glszm_SmallAreaLowGrayLevelEmphasis | wavelet-HLL_glszm_ZoneEntropy |
|  |  | wavelet-HLL_glszm_ZoneVariance | wavelet-HLL_ngtdm_Strength | wavelet-HLH_firstorder_Kurtosis |
|  |  | wavelet-HLH_firstorder_Median | wavelet-HLH_glcm_Idn | wavelet-HLH_glcm_InverseVariance |
|  |  | wavelet-HLH_glcm_MCC | wavelet-HLH_glcm_MaximumProbability | wavelet-HLH_glrlm_ShortRunLowGrayLevelEmphasis |
|  |  | wavelet-HLH_glszm_GrayLevelNonUniformityNormalized | wavelet-HLH_glszm_SmallAreaEmphasis | wavelet-HLH_glszm_SmallAreaLowGrayLevelEmphasis |
|  |  | wavelet-HLH_glszm_ZoneEntropy | wavelet-HHL_firstorder_Median | wavelet-HHL_glcm_Correlation |
|  |  | wavelet-HHL_glcm_Idmn | wavelet-HHL_glcm_Imc1 | wavelet-HHL_glcm_MCC |
|  |  | wavelet-HHL_gldm_LargeDependenceHighGrayLevelEmphasis | wavelet-HHL_glrlm_ShortRunLowGrayLevelEmphasis | wavelet-HHL_glszm_GrayLevelNonUniformityNormalized |
|  |  | wavelet-HHL_glszm_LowGrayLevelZoneEmphasis | wavelet-HHL_glszm_SizeZoneNonUniformityNormalized | wavelet-HHL_glszm_SmallAreaLowGrayLevelEmphasis |
|  |  | wavelet-HHL_glszm_ZoneEntropy | wavelet-HHL_glszm_ZoneVariance | wavelet-HHL_ngtdm_Busyness |
|  |  | wavelet-HHH_firstorder_Kurtosis | wavelet-HHH_firstorder_Skewness | wavelet-HHH_glrlm_RunEntropy |
|  |  | wavelet-HHH_glrlm_ShortRunLowGrayLevelEmphasis | wavelet-HHH_glszm_GrayLevelNonUniformityNormalized | wavelet-HHH_glszm_SizeZoneNonUniformityNormalized |
|  |  | wavelet-HHH_glszm_SmallAreaEmphasis | wavelet-HHH_ngtdm_Contrast | wavelet-LLL_firstorder_10Percentile |
|  |  | wavelet-LLL_firstorder_Median | wavelet-LLL_glcm_Correlation | wavelet-LLL_glcm_Imc1 |
|  |  | wavelet-LLL_glcm_MaximumProbability | wavelet-LLL_gldm_LargeDependenceLowGrayLevelEmphasis | wavelet-LLL_glrlm_ShortRunLowGrayLevelEmphasis |
|  |  | wavelet-LLL_glszm_GrayLevelVariance | wavelet-LLL_glszm_LowGrayLevelZoneEmphasis | wavelet-LLL_glszm_SmallAreaLowGrayLevelEmphasis |
|  |  | wavelet-LLL_glszm_ZoneVariance |  |  |
| Selection_13 | 0.00098 | original_shape_Flatness | original_shape_Maximum2DDiameterSlice | original_shape_Maximum3DDiameter |
|  |  | original_shape_MinorAxisLength | original_shape_Sphericity | original_shape_SurfaceArea |
|  |  | original_shape_SurfaceVolumeRatio | original_firstorder_Kurtosis | original_firstorder_Skewness |
|  |  | original_glcm_Correlation | original_glcm_Idn | original_glcm_InverseVariance |
|  |  | original_glcm_JointAverage | original_glcm_MCC | original_glcm_SumAverage |
|  |  | original_glrlm_LongRunHighGrayLevelEmphasis | original_glszm_SmallAreaEmphasis | original_glszm_SmallAreaLowGrayLevelEmphasis |
|  |  | original_ngtdm_Contrast | original_ngtdm_Strength | wavelet-LLH_firstorder_10Percentile |
|  |  | wavelet-LLH_firstorder_Kurtosis | wavelet-LLH_firstorder_RootMeanSquared | wavelet-LLH_firstorder_Skewness |
|  |  | wavelet-LLH_glcm_JointAverage | wavelet-LLH_glcm_MCC | wavelet-LLH_glcm_MaximumProbability |
|  |  | wavelet-LLH_glcm_SumAverage | wavelet-LLH_gldm_LargeDependenceLowGrayLevelEmphasis | wavelet-LLH_glrlm_LongRunEmphasis |
|  |  | wavelet-LLH_glrlm_ShortRunEmphasis | wavelet-LLH_glszm_SizeZoneNonUniformity | wavelet-LLH_glszm_SizeZoneNonUniformityNormalized |
|  |  | wavelet-LLH_glszm_SmallAreaLowGrayLevelEmphasis | wavelet-LLH_glszm_ZoneEntropy | wavelet-LLH_ngtdm_Busyness |
|  |  | wavelet-LLH_ngtdm_Contrast | wavelet-LHL_firstorder_Mean | wavelet-LHL_firstorder_Skewness |
|  |  | wavelet-LHL_glcm_Correlation | wavelet-LHL_glcm_Idmn | wavelet-LHL_glcm_Idn |
|  |  | wavelet-LHL_glcm_Imc2 | wavelet-LHL_glcm_MaximumProbability | wavelet-LHL_gldm_DependenceEntropy |
|  |  | wavelet-LHL_gldm_LargeDependenceLowGrayLevelEmphasis | wavelet-LHL_glszm_GrayLevelNonUniformityNormalized | wavelet-LHL_glszm_LargeAreaHighGrayLevelEmphasis |
|  |  | wavelet-LHL_glszm_SizeZoneNonUniformityNormalized | wavelet-LHH_firstorder_Mean | wavelet-LHH_glcm_ClusterShade |
|  |  | wavelet-LHH_glcm_Idmn | wavelet-LHH_glcm_MCC | wavelet-LHH_glcm_MaximumProbability |
|  |  | wavelet-LHH_glszm_GrayLevelNonUniformityNormalized | wavelet-LHH_glszm_LowGrayLevelZoneEmphasis | wavelet-LHH_glszm_SizeZoneNonUniformity |
|  |  | wavelet-LHH_glszm_SizeZoneNonUniformityNormalized | wavelet-LHH_glszm_SmallAreaLowGrayLevelEmphasis | wavelet-LHH_ngtdm_Contrast |
|  |  | wavelet-HLL_firstorder_Kurtosis | wavelet-HLL_firstorder_Maximum | wavelet-HLL_firstorder_Skewness |
|  |  | wavelet-HLL_glcm_Correlation | wavelet-HLL_glcm_Idn | wavelet-HLL_glcm_Imc2 |
|  |  | wavelet-HLL_glrlm_LongRunLowGrayLevelEmphasis | wavelet-HLL_glrlm_RunVariance | wavelet-HLL_glszm_GrayLevelNonUniformity |
|  |  | wavelet-HLL_glszm_GrayLevelNonUniformityNormalized | wavelet-HLL_glszm_GrayLevelVariance | wavelet-HLL_glszm_LargeAreaEmphasis |
|  |  | wavelet-HLL_glszm_SmallAreaEmphasis | wavelet-HLL_glszm_SmallAreaLowGrayLevelEmphasis | wavelet-HLL_glszm_ZoneEntropy |
|  |  | wavelet-HLL_glszm_ZoneVariance | wavelet-HLL_ngtdm_Strength | wavelet-HLH_firstorder_Kurtosis |
|  |  | wavelet-HLH_firstorder_Median | wavelet-HLH_glcm_Idn | wavelet-HLH_glcm_InverseVariance |
|  |  | wavelet-HLH_glcm_MCC | wavelet-HLH_glcm_MaximumProbability | wavelet-HLH_glrlm_ShortRunLowGrayLevelEmphasis |
|  |  | wavelet-HLH_glszm_GrayLevelNonUniformityNormalized | wavelet-HLH_glszm_SmallAreaEmphasis | wavelet-HLH_glszm_SmallAreaLowGrayLevelEmphasis |
|  |  | wavelet-HLH_glszm_ZoneEntropy | wavelet-HHL_firstorder_Median | wavelet-HHL_firstorder_Skewness |
|  |  | wavelet-HHL_glcm_Correlation | wavelet-HHL_glcm_Idmn | wavelet-HHL_glcm_Imc1 |
|  |  | wavelet-HHL_glcm_MCC | wavelet-HHL_gldm_LargeDependenceHighGrayLevelEmphasis | wavelet-HHL_glrlm_ShortRunLowGrayLevelEmphasis |
|  |  | wavelet-HHL_glszm_GrayLevelNonUniformityNormalized | wavelet-HHL_glszm_LowGrayLevelZoneEmphasis | wavelet-HHL_glszm_SmallAreaEmphasis |
|  |  | wavelet-HHL_glszm_SmallAreaLowGrayLevelEmphasis | wavelet-HHL_glszm_ZoneEntropy | wavelet-HHL_glszm_ZoneVariance |
|  |  | wavelet-HHL_ngtdm_Busyness | wavelet-HHL_ngtdm_Contrast | wavelet-HHH_firstorder_Kurtosis |
|  |  | wavelet-HHH_firstorder_Skewness | wavelet-HHH_gldm_DependenceEntropy | wavelet-HHH_glrlm_RunEntropy |
|  |  | wavelet-HHH_glrlm_ShortRunLowGrayLevelEmphasis | wavelet-HHH_glszm_GrayLevelNonUniformityNormalized | wavelet-HHH_glszm_SizeZoneNonUniformityNormalized |
|  |  | wavelet-HHH_glszm_SmallAreaEmphasis | wavelet-HHH_glszm_ZoneEntropy | wavelet-HHH_ngtdm_Contrast |
|  |  | wavelet-LLL_firstorder_10Percentile | wavelet-LLL_firstorder_Median | wavelet-LLL_glcm_Correlation |
|  |  | wavelet-LLL_glcm_Imc1 | wavelet-LLL_glcm_MaximumProbability | wavelet-LLL_gldm_LargeDependenceLowGrayLevelEmphasis |
|  |  | wavelet-LLL_glrlm_ShortRunLowGrayLevelEmphasis | wavelet-LLL_glszm_GrayLevelVariance | wavelet-LLL_glszm_LowGrayLevelZoneEmphasis |
|  |  | wavelet-LLL_glszm_SmallAreaLowGrayLevelEmphasis | wavelet-LLL_glszm_ZonePercentage | wavelet-LLL_glszm_ZoneVariance |
| Selection_14 | 0.00071 | original_shape_Elongation | original_shape_Flatness | original_shape_Maximum2DDiameterSlice |
|  |  | original_shape_Maximum3DDiameter | original_shape_MinorAxisLength | original_shape_Sphericity |
|  |  | original_shape_SurfaceArea | original_firstorder_Kurtosis | original_firstorder_Skewness |
|  |  | original_glcm_Correlation | original_glcm_Idn | original_glcm_InverseVariance |
|  |  | original_glcm_JointAverage | original_glcm_MCC | original_glcm_SumAverage |
|  |  | original_glrlm_LongRunHighGrayLevelEmphasis | original_glszm_SmallAreaEmphasis | original_glszm_SmallAreaLowGrayLevelEmphasis |
|  |  | original_ngtdm_Busyness | original_ngtdm_Contrast | original_ngtdm_Strength |
|  |  | wavelet-LLH_firstorder_10Percentile | wavelet-LLH_firstorder_Kurtosis | wavelet-LLH_firstorder_RootMeanSquared |
|  |  | wavelet-LLH_firstorder_Skewness | wavelet-LLH_glcm_Idn | wavelet-LLH_glcm_JointAverage |
|  |  | wavelet-LLH_glcm_MCC | wavelet-LLH_glcm_MaximumProbability | wavelet-LLH_glcm_SumAverage |
|  |  | wavelet-LLH_gldm_DependenceVariance | wavelet-LLH_gldm_LargeDependenceLowGrayLevelEmphasis | wavelet-LLH_glrlm_LongRunEmphasis |
|  |  | wavelet-LLH_glrlm_ShortRunEmphasis | wavelet-LLH_glszm_SizeZoneNonUniformity | wavelet-LLH_glszm_SizeZoneNonUniformityNormalized |
|  |  | wavelet-LLH_glszm_SmallAreaLowGrayLevelEmphasis | wavelet-LLH_glszm_ZoneEntropy | wavelet-LLH_ngtdm_Busyness |
|  |  | wavelet-LLH_ngtdm_Contrast | wavelet-LHL_firstorder_Mean | wavelet-LHL_glcm_Correlation |
|  |  | wavelet-LHL_glcm_Idmn | wavelet-LHL_glcm_Idn | wavelet-LHL_glcm_Imc2 |
|  |  | wavelet-LHL_glcm_MaximumProbability | wavelet-LHL_gldm_DependenceEntropy | wavelet-LHL_glszm_GrayLevelNonUniformityNormalized |
|  |  | wavelet-LHL_glszm_LargeAreaHighGrayLevelEmphasis | wavelet-LHL_glszm_SizeZoneNonUniformityNormalized | wavelet-LHH_firstorder_Mean |
|  |  | wavelet-LHH_glcm_ClusterShade | wavelet-LHH_glcm_Idmn | wavelet-LHH_glcm_MCC |
|  |  | wavelet-LHH_glcm_MaximumProbability | wavelet-LHH_glszm_GrayLevelNonUniformityNormalized | wavelet-LHH_glszm_LowGrayLevelZoneEmphasis |
|  |  | wavelet-LHH_glszm_SizeZoneNonUniformity | wavelet-LHH_glszm_SizeZoneNonUniformityNormalized | wavelet-LHH_glszm_SmallAreaLowGrayLevelEmphasis |
|  |  | wavelet-LHH_ngtdm_Contrast | wavelet-HLL_firstorder_Kurtosis | wavelet-HLL_firstorder_Maximum |
|  |  | wavelet-HLL_firstorder_Skewness | wavelet-HLL_glcm_Correlation | wavelet-HLL_glcm_Idn |
|  |  | wavelet-HLL_glcm_Imc2 | wavelet-HLL_glrlm_LongRunHighGrayLevelEmphasis | wavelet-HLL_glrlm_LongRunLowGrayLevelEmphasis |
|  |  | wavelet-HLL_glrlm_RunVariance | wavelet-HLL_glszm_GrayLevelNonUniformity | wavelet-HLL_glszm_GrayLevelNonUniformityNormalized |
|  |  | wavelet-HLL_glszm_GrayLevelVariance | wavelet-HLL_glszm_LargeAreaEmphasis | wavelet-HLL_glszm_SmallAreaEmphasis |
|  |  | wavelet-HLL_glszm_SmallAreaLowGrayLevelEmphasis | wavelet-HLL_glszm_ZoneEntropy | wavelet-HLL_glszm_ZoneVariance |
|  |  | wavelet-HLL_ngtdm_Strength | wavelet-HLH_firstorder_Kurtosis | wavelet-HLH_firstorder_Median |
|  |  | wavelet-HLH_glcm_Idn | wavelet-HLH_glcm_InverseVariance | wavelet-HLH_glcm_MCC |
|  |  | wavelet-HLH_glcm_MaximumProbability | wavelet-HLH_glrlm_ShortRunLowGrayLevelEmphasis | wavelet-HLH_glszm_GrayLevelNonUniformityNormalized |
|  |  | wavelet-HLH_glszm_SmallAreaEmphasis | wavelet-HLH_glszm_SmallAreaLowGrayLevelEmphasis | wavelet-HLH_glszm_ZoneEntropy |
|  |  | wavelet-HHL_firstorder_Median | wavelet-HHL_firstorder_Skewness | wavelet-HHL_glcm_Idmn |
|  |  | wavelet-HHL_glcm_Imc1 | wavelet-HHL_glcm_MCC | wavelet-HHL_gldm_LargeDependenceHighGrayLevelEmphasis |
|  |  | wavelet-HHL_glrlm_ShortRunLowGrayLevelEmphasis | wavelet-HHL_glszm_GrayLevelNonUniformityNormalized | wavelet-HHL_glszm_SmallAreaEmphasis |
|  |  | wavelet-HHL_glszm_SmallAreaLowGrayLevelEmphasis | wavelet-HHL_glszm_ZoneEntropy | wavelet-HHL_glszm_ZoneVariance |
|  |  | wavelet-HHL_ngtdm_Busyness | wavelet-HHL_ngtdm_Contrast | wavelet-HHH_firstorder_Kurtosis |
|  |  | wavelet-HHH_firstorder_Skewness | wavelet-HHH_gldm_DependenceEntropy | wavelet-HHH_glrlm_RunEntropy |
|  |  | wavelet-HHH_glrlm_ShortRunLowGrayLevelEmphasis | wavelet-HHH_glszm_GrayLevelNonUniformityNormalized | wavelet-HHH_glszm_SizeZoneNonUniformityNormalized |
|  |  | wavelet-HHH_glszm_SmallAreaEmphasis | wavelet-HHH_glszm_ZoneEntropy | wavelet-HHH_ngtdm_Contrast |
|  |  | wavelet-LLL_firstorder_10Percentile | wavelet-LLL_firstorder_InterquartileRange | wavelet-LLL_firstorder_Median |
|  |  | wavelet-LLL_glcm_Correlation | wavelet-LLL_glcm_Imc1 | wavelet-LLL_glcm_MaximumProbability |
|  |  | wavelet-LLL_gldm_LargeDependenceLowGrayLevelEmphasis | wavelet-LLL_glrlm_ShortRunLowGrayLevelEmphasis | wavelet-LLL_glszm_GrayLevelVariance |
|  |  | wavelet-LLL_glszm_LowGrayLevelZoneEmphasis | wavelet-LLL_glszm_SizeZoneNonUniformityNormalized | wavelet-LLL_glszm_SmallAreaLowGrayLevelEmphasis |
|  |  | wavelet-LLL_glszm_ZonePercentage | wavelet-LLL_glszm_ZoneVariance |  |
| Selection_15 | 0.00051 | original_shape_Elongation | original_shape_Flatness | original_shape_Maximum2DDiameterSlice |
|  |  | original_shape_Maximum3DDiameter | original_shape_MinorAxisLength | original_shape_Sphericity |
|  |  | original_shape_SurfaceArea | original_firstorder_Kurtosis | original_firstorder_Skewness |
|  |  | original_glcm_Correlation | original_glcm_Idn | original_glcm_Imc2 |
|  |  | original_glcm_InverseVariance | original_glcm_JointAverage | original_glcm_MCC |
|  |  | original_glcm_SumAverage | original_glrlm_LongRunHighGrayLevelEmphasis | original_glszm_SmallAreaEmphasis |
|  |  | original_glszm_SmallAreaLowGrayLevelEmphasis | original_ngtdm_Busyness | original_ngtdm_Contrast |
|  |  | original_ngtdm_Strength | wavelet-LLH_firstorder_10Percentile | wavelet-LLH_firstorder_Kurtosis |
|  |  | wavelet-LLH_firstorder_RootMeanSquared | wavelet-LLH_firstorder_Skewness | wavelet-LLH_glcm_Idn |
|  |  | wavelet-LLH_glcm_JointAverage | wavelet-LLH_glcm_MCC | wavelet-LLH_glcm_MaximumProbability |
|  |  | wavelet-LLH_glcm_SumAverage | wavelet-LLH_gldm_DependenceVariance | wavelet-LLH_gldm_LargeDependenceLowGrayLevelEmphasis |
|  |  | wavelet-LLH_gldm_SmallDependenceLowGrayLevelEmphasis | wavelet-LLH_glrlm_LongRunEmphasis | wavelet-LLH_glrlm_ShortRunEmphasis |
|  |  | wavelet-LLH_glszm_SizeZoneNonUniformity | wavelet-LLH_glszm_SizeZoneNonUniformityNormalized | wavelet-LLH_glszm_SmallAreaLowGrayLevelEmphasis |
|  |  | wavelet-LLH_glszm_ZoneEntropy | wavelet-LLH_ngtdm_Busyness | wavelet-LHL_firstorder_Mean |
|  |  | wavelet-LHL_glcm_Correlation | wavelet-LHL_glcm_Idmn | wavelet-LHL_glcm_Idn |
|  |  | wavelet-LHL_glcm_Imc2 | wavelet-LHL_glcm_MaximumProbability | wavelet-LHL_gldm_DependenceEntropy |
|  |  | wavelet-LHL_glszm_GrayLevelNonUniformityNormalized | wavelet-LHL_glszm_LargeAreaHighGrayLevelEmphasis | wavelet-LHL_glszm_SizeZoneNonUniformityNormalized |
|  |  | wavelet-LHH_firstorder_Kurtosis | wavelet-LHH_firstorder_Mean | wavelet-LHH_glcm_ClusterShade |
|  |  | wavelet-LHH_glcm_Idmn | wavelet-LHH_glcm_MCC | wavelet-LHH_glcm_MaximumProbability |
|  |  | wavelet-LHH_glrlm_RunEntropy | wavelet-LHH_glszm_GrayLevelNonUniformityNormalized | wavelet-LHH_glszm_LowGrayLevelZoneEmphasis |
|  |  | wavelet-LHH_glszm_SizeZoneNonUniformity | wavelet-LHH_glszm_SizeZoneNonUniformityNormalized | wavelet-LHH_glszm_SmallAreaLowGrayLevelEmphasis |
|  |  | wavelet-LHH_ngtdm_Contrast | wavelet-HLL_firstorder_Kurtosis | wavelet-HLL_firstorder_Maximum |
|  |  | wavelet-HLL_firstorder_Median | wavelet-HLL_firstorder_Skewness | wavelet-HLL_glcm_Correlation |
|  |  | wavelet-HLL_glcm_Idn | wavelet-HLL_glcm_Imc2 | wavelet-HLL_glrlm_LongRunHighGrayLevelEmphasis |
|  |  | wavelet-HLL_glrlm_LongRunLowGrayLevelEmphasis | wavelet-HLL_glrlm_RunVariance | wavelet-HLL_glszm_GrayLevelNonUniformity |
|  |  | wavelet-HLL_glszm_GrayLevelNonUniformityNormalized | wavelet-HLL_glszm_GrayLevelVariance | wavelet-HLL_glszm_LargeAreaEmphasis |
|  |  | wavelet-HLL_glszm_SmallAreaEmphasis | wavelet-HLL_glszm_SmallAreaLowGrayLevelEmphasis | wavelet-HLL_glszm_ZoneEntropy |
|  |  | wavelet-HLL_glszm_ZoneVariance | wavelet-HLL_ngtdm_Strength | wavelet-HLH_firstorder_Kurtosis |
|  |  | wavelet-HLH_firstorder_Median | wavelet-HLH_glcm_Idn | wavelet-HLH_glcm_InverseVariance |
|  |  | wavelet-HLH_glcm_MCC | wavelet-HLH_glcm_MaximumProbability | wavelet-HLH_glrlm_ShortRunLowGrayLevelEmphasis |
|  |  | wavelet-HLH_glszm_GrayLevelNonUniformityNormalized | wavelet-HLH_glszm_SmallAreaEmphasis | wavelet-HLH_glszm_SmallAreaLowGrayLevelEmphasis |
|  |  | wavelet-HLH_glszm_ZoneEntropy | wavelet-HHL_firstorder_Median | wavelet-HHL_firstorder_Skewness |
|  |  | wavelet-HHL_glcm_Idmn | wavelet-HHL_glcm_Imc1 | wavelet-HHL_glcm_MCC |
|  |  | wavelet-HHL_gldm_LargeDependenceHighGrayLevelEmphasis | wavelet-HHL_glrlm_ShortRunLowGrayLevelEmphasis | wavelet-HHL_glszm_GrayLevelNonUniformityNormalized |
|  |  | wavelet-HHL_glszm_SmallAreaEmphasis | wavelet-HHL_glszm_SmallAreaLowGrayLevelEmphasis | wavelet-HHL_glszm_ZoneEntropy |
|  |  | wavelet-HHL_glszm_ZoneVariance | wavelet-HHL_ngtdm_Busyness | wavelet-HHL_ngtdm_Contrast |
|  |  | wavelet-HHH_firstorder_Kurtosis | wavelet-HHH_firstorder_Skewness | wavelet-HHH_gldm_DependenceEntropy |
|  |  | wavelet-HHH_glrlm_RunEntropy | wavelet-HHH_glszm_GrayLevelNonUniformityNormalized | wavelet-HHH_glszm_SizeZoneNonUniformityNormalized |
|  |  | wavelet-HHH_glszm_SmallAreaEmphasis | wavelet-HHH_glszm_ZoneEntropy | wavelet-HHH_ngtdm_Contrast |
|  |  | wavelet-LLL_firstorder_10Percentile | wavelet-LLL_firstorder_InterquartileRange | wavelet-LLL_firstorder_Median |
|  |  | wavelet-LLL_glcm_Correlation | wavelet-LLL_glcm_Imc1 | wavelet-LLL_glcm_MaximumProbability |
|  |  | wavelet-LLL_gldm_DependenceEntropy | wavelet-LLL_gldm_LargeDependenceLowGrayLevelEmphasis | wavelet-LLL_glrlm_ShortRunLowGrayLevelEmphasis |
|  |  | wavelet-LLL_glszm_GrayLevelVariance | wavelet-LLL_glszm_LowGrayLevelZoneEmphasis | wavelet-LLL_glszm_SizeZoneNonUniformityNormalized |
|  |  | wavelet-LLL_glszm_SmallAreaLowGrayLevelEmphasis | wavelet-LLL_glszm_ZonePercentage | wavelet-LLL_glszm_ZoneVariance |
| Selection_16 | 0.00037 | original_shape_Elongation | original_shape_Flatness | original_shape_Maximum2DDiameterSlice |
|  |  | original_shape_Maximum3DDiameter | original_shape_MinorAxisLength | original_shape_Sphericity |
|  |  | original_shape_SurfaceArea | original_firstorder_Kurtosis | original_firstorder_Skewness |
|  |  | original_glcm_Correlation | original_glcm_Idn | original_glcm_Imc2 |
|  |  | original_glcm_InverseVariance | original_glcm_JointAverage | original_glcm_MCC |
|  |  | original_glcm_SumAverage | original_glrlm_GrayLevelNonUniformity | original_glrlm_LongRunHighGrayLevelEmphasis |
|  |  | original_glszm_SmallAreaEmphasis | original_glszm_SmallAreaLowGrayLevelEmphasis | original_ngtdm_Busyness |
|  |  | original_ngtdm_Contrast | original_ngtdm_Strength | wavelet-LLH_firstorder_10Percentile |
|  |  | wavelet-LLH_firstorder_Kurtosis | wavelet-LLH_firstorder_RootMeanSquared | wavelet-LLH_firstorder_Skewness |
|  |  | wavelet-LLH_glcm_Idn | wavelet-LLH_glcm_JointAverage | wavelet-LLH_glcm_MCC |
|  |  | wavelet-LLH_glcm_MaximumProbability | wavelet-LLH_glcm_SumAverage | wavelet-LLH_gldm_DependenceVariance |
|  |  | wavelet-LLH_gldm_LargeDependenceLowGrayLevelEmphasis | wavelet-LLH_gldm_SmallDependenceLowGrayLevelEmphasis | wavelet-LLH_glrlm_LongRunEmphasis |
|  |  | wavelet-LLH_glrlm_ShortRunEmphasis | wavelet-LLH_glszm_SizeZoneNonUniformity | wavelet-LLH_glszm_SizeZoneNonUniformityNormalized |
|  |  | wavelet-LLH_glszm_SmallAreaLowGrayLevelEmphasis | wavelet-LLH_glszm_ZoneEntropy | wavelet-LLH_ngtdm_Busyness |
|  |  | wavelet-LHL_firstorder_Mean | wavelet-LHL_glcm_Correlation | wavelet-LHL_glcm_Idmn |
|  |  | wavelet-LHL_glcm_Idn | wavelet-LHL_glcm_Imc2 | wavelet-LHL_glcm_MaximumProbability |
|  |  | wavelet-LHL_gldm_DependenceEntropy | wavelet-LHL_glszm_GrayLevelNonUniformityNormalized | wavelet-LHL_glszm_LargeAreaHighGrayLevelEmphasis |
|  |  | wavelet-LHL_glszm_SizeZoneNonUniformityNormalized | wavelet-LHH_firstorder_Mean | wavelet-LHH_glcm_ClusterShade |
|  |  | wavelet-LHH_glcm_Idmn | wavelet-LHH_glcm_MCC | wavelet-LHH_glcm_MaximumProbability |
|  |  | wavelet-LHH_glrlm_RunEntropy | wavelet-LHH_glszm_GrayLevelNonUniformityNormalized | wavelet-LHH_glszm_LowGrayLevelZoneEmphasis |
|  |  | wavelet-LHH_glszm_SizeZoneNonUniformity | wavelet-LHH_glszm_SizeZoneNonUniformityNormalized | wavelet-LHH_glszm_SmallAreaLowGrayLevelEmphasis |
|  |  | wavelet-LHH_ngtdm_Contrast | wavelet-HLL_firstorder_Kurtosis | wavelet-HLL_firstorder_Maximum |
|  |  | wavelet-HLL_firstorder_Median | wavelet-HLL_firstorder_Skewness | wavelet-HLL_glcm_Correlation |
|  |  | wavelet-HLL_glcm_Idn | wavelet-HLL_glcm_Imc2 | wavelet-HLL_glrlm_LongRunHighGrayLevelEmphasis |
|  |  | wavelet-HLL_glrlm_LongRunLowGrayLevelEmphasis | wavelet-HLL_glrlm_RunVariance | wavelet-HLL_glszm_GrayLevelNonUniformity |
|  |  | wavelet-HLL_glszm_GrayLevelNonUniformityNormalized | wavelet-HLL_glszm_GrayLevelVariance | wavelet-HLL_glszm_LargeAreaEmphasis |
|  |  | wavelet-HLL_glszm_SmallAreaEmphasis | wavelet-HLL_glszm_SmallAreaLowGrayLevelEmphasis | wavelet-HLL_glszm_ZoneEntropy |
|  |  | wavelet-HLL_glszm_ZoneVariance | wavelet-HLL_ngtdm_Strength | wavelet-HLH_firstorder_Kurtosis |
|  |  | wavelet-HLH_firstorder_Median | wavelet-HLH_glcm_Idn | wavelet-HLH_glcm_InverseVariance |
|  |  | wavelet-HLH_glcm_MCC | wavelet-HLH_glcm_MaximumProbability | wavelet-HLH_glrlm_ShortRunLowGrayLevelEmphasis |
|  |  | wavelet-HLH_glszm_GrayLevelNonUniformityNormalized | wavelet-HLH_glszm_SmallAreaEmphasis | wavelet-HLH_glszm_SmallAreaLowGrayLevelEmphasis |
|  |  | wavelet-HLH_glszm_ZoneEntropy | wavelet-HHL_firstorder_Kurtosis | wavelet-HHL_firstorder_Median |
|  |  | wavelet-HHL_firstorder_Skewness | wavelet-HHL_glcm_Idmn | wavelet-HHL_glcm_MCC |
|  |  | wavelet-HHL_gldm_LargeDependenceHighGrayLevelEmphasis | wavelet-HHL_glrlm_ShortRunLowGrayLevelEmphasis | wavelet-HHL_glszm_GrayLevelNonUniformityNormalized |
|  |  | wavelet-HHL_glszm_LargeAreaEmphasis | wavelet-HHL_glszm_SmallAreaEmphasis | wavelet-HHL_glszm_SmallAreaLowGrayLevelEmphasis |
|  |  | wavelet-HHL_glszm_ZoneEntropy | wavelet-HHL_glszm_ZoneVariance | wavelet-HHL_ngtdm_Busyness |
|  |  | wavelet-HHL_ngtdm_Contrast | wavelet-HHH_firstorder_Kurtosis | wavelet-HHH_firstorder_Mean |
|  |  | wavelet-HHH_firstorder_Skewness | wavelet-HHH_gldm_DependenceEntropy | wavelet-HHH_glrlm_RunEntropy |
|  |  | wavelet-HHH_glszm_GrayLevelNonUniformityNormalized | wavelet-HHH_glszm_SizeZoneNonUniformityNormalized | wavelet-HHH_glszm_SmallAreaEmphasis |
|  |  | wavelet-HHH_glszm_ZoneEntropy | wavelet-HHH_ngtdm_Contrast | wavelet-LLL_firstorder_10Percentile |
|  |  | wavelet-LLL_firstorder_InterquartileRange | wavelet-LLL_firstorder_Median | wavelet-LLL_glcm_Correlation |
|  |  | wavelet-LLL_glcm_Imc1 | wavelet-LLL_glcm_MaximumProbability | wavelet-LLL_gldm_DependenceEntropy |
|  |  | wavelet-LLL_gldm_LargeDependenceHighGrayLevelEmphasis | wavelet-LLL_gldm_LargeDependenceLowGrayLevelEmphasis | wavelet-LLL_glrlm_ShortRunLowGrayLevelEmphasis |
|  |  | wavelet-LLL_glszm_GrayLevelVariance | wavelet-LLL_glszm_LowGrayLevelZoneEmphasis | wavelet-LLL_glszm_SizeZoneNonUniformityNormalized |
|  |  | wavelet-LLL_glszm_SmallAreaLowGrayLevelEmphasis | wavelet-LLL_glszm_ZoneVariance |  |
| Selection_17 | 0.00027 | original_shape_Elongation | original_shape_Flatness | original_shape_Maximum2DDiameterSlice |
|  |  | original_shape_Maximum3DDiameter | original_shape_MinorAxisLength | original_shape_Sphericity |
|  |  | original_shape_SurfaceArea | original_firstorder_Kurtosis | original_firstorder_Skewness |
|  |  | original_glcm_Correlation | original_glcm_Idn | original_glcm_Imc2 |
|  |  | original_glcm_InverseVariance | original_glcm_JointAverage | original_glcm_MCC |
|  |  | original_glcm_SumAverage | original_glrlm_GrayLevelNonUniformity | original_glrlm_LongRunHighGrayLevelEmphasis |
|  |  | original_glszm_SmallAreaEmphasis | original_glszm_SmallAreaLowGrayLevelEmphasis | original_ngtdm_Busyness |
|  |  | original_ngtdm_Contrast | original_ngtdm_Strength | wavelet-LLH_firstorder_10Percentile |
|  |  | wavelet-LLH_firstorder_Kurtosis | wavelet-LLH_firstorder_RootMeanSquared | wavelet-LLH_firstorder_Skewness |
|  |  | wavelet-LLH_glcm_Idn | wavelet-LLH_glcm_JointAverage | wavelet-LLH_glcm_MCC |
|  |  | wavelet-LLH_glcm_MaximumProbability | wavelet-LLH_glcm_SumAverage | wavelet-LLH_gldm_DependenceVariance |
|  |  | wavelet-LLH_gldm_SmallDependenceLowGrayLevelEmphasis | wavelet-LLH_glrlm_LongRunEmphasis | wavelet-LLH_glrlm_ShortRunEmphasis |
|  |  | wavelet-LLH_glszm_SizeZoneNonUniformity | wavelet-LLH_glszm_SizeZoneNonUniformityNormalized | wavelet-LLH_glszm_SmallAreaLowGrayLevelEmphasis |
|  |  | wavelet-LLH_glszm_ZoneEntropy | wavelet-LLH_ngtdm_Busyness | wavelet-LHL_firstorder_Mean |
|  |  | wavelet-LHL_glcm_Correlation | wavelet-LHL_glcm_Idmn | wavelet-LHL_glcm_Idn |
|  |  | wavelet-LHL_glcm_Imc2 | wavelet-LHL_glcm_MaximumProbability | wavelet-LHL_gldm_DependenceEntropy |
|  |  | wavelet-LHL_glszm_GrayLevelNonUniformityNormalized | wavelet-LHL_glszm_LargeAreaHighGrayLevelEmphasis | wavelet-LHL_glszm_SizeZoneNonUniformityNormalized |
|  |  | wavelet-LHH_firstorder_Mean | wavelet-LHH_glcm_ClusterShade | wavelet-LHH_glcm_Idmn |
|  |  | wavelet-LHH_glcm_MCC | wavelet-LHH_glcm_MaximumProbability | wavelet-LHH_glrlm_RunEntropy |
|  |  | wavelet-LHH_glszm_GrayLevelNonUniformityNormalized | wavelet-LHH_glszm_LowGrayLevelZoneEmphasis | wavelet-LHH_glszm_SizeZoneNonUniformity |
|  |  | wavelet-LHH_glszm_SizeZoneNonUniformityNormalized | wavelet-LHH_glszm_SmallAreaLowGrayLevelEmphasis | wavelet-LHH_glszm_ZoneEntropy |
|  |  | wavelet-LHH_ngtdm_Contrast | wavelet-HLL_firstorder_Kurtosis | wavelet-HLL_firstorder_Maximum |
|  |  | wavelet-HLL_firstorder_Median | wavelet-HLL_firstorder_Skewness | wavelet-HLL_glcm_Correlation |
|  |  | wavelet-HLL_glcm_Idn | wavelet-HLL_glcm_Imc2 | wavelet-HLL_glrlm_LongRunHighGrayLevelEmphasis |
|  |  | wavelet-HLL_glrlm_LongRunLowGrayLevelEmphasis | wavelet-HLL_glrlm_RunVariance | wavelet-HLL_glszm_GrayLevelNonUniformity |
|  |  | wavelet-HLL_glszm_GrayLevelNonUniformityNormalized | wavelet-HLL_glszm_GrayLevelVariance | wavelet-HLL_glszm_LargeAreaEmphasis |
|  |  | wavelet-HLL_glszm_SmallAreaEmphasis | wavelet-HLL_glszm_SmallAreaLowGrayLevelEmphasis | wavelet-HLL_glszm_ZoneEntropy |
|  |  | wavelet-HLL_glszm_ZoneVariance | wavelet-HLL_ngtdm_Strength | wavelet-HLH_firstorder_Kurtosis |
|  |  | wavelet-HLH_firstorder_Median | wavelet-HLH_glcm_Correlation | wavelet-HLH_glcm_Idn |
|  |  | wavelet-HLH_glcm_InverseVariance | wavelet-HLH_glcm_MCC | wavelet-HLH_glcm_MaximumProbability |
|  |  | wavelet-HLH_glrlm_ShortRunLowGrayLevelEmphasis | wavelet-HLH_glszm_GrayLevelNonUniformityNormalized | wavelet-HLH_glszm_SmallAreaEmphasis |
|  |  | wavelet-HLH_glszm_SmallAreaLowGrayLevelEmphasis | wavelet-HLH_glszm_ZoneEntropy | wavelet-HHL_firstorder_Kurtosis |
|  |  | wavelet-HHL_firstorder_Maximum | wavelet-HHL_firstorder_Median | wavelet-HHL_firstorder_Skewness |
|  |  | wavelet-HHL_glcm_Imc1 | wavelet-HHL_glcm_MCC | wavelet-HHL_gldm_LargeDependenceHighGrayLevelEmphasis |
|  |  | wavelet-HHL_glrlm_ShortRunLowGrayLevelEmphasis | wavelet-HHL_glszm_GrayLevelNonUniformityNormalized | wavelet-HHL_glszm_LargeAreaEmphasis |
|  |  | wavelet-HHL_glszm_SmallAreaEmphasis | wavelet-HHL_glszm_SmallAreaLowGrayLevelEmphasis | wavelet-HHL_glszm_ZoneEntropy |
|  |  | wavelet-HHL_glszm_ZoneVariance | wavelet-HHL_ngtdm_Busyness | wavelet-HHL_ngtdm_Contrast |
|  |  | wavelet-HHH_firstorder_Kurtosis | wavelet-HHH_firstorder_Mean | wavelet-HHH_firstorder_Skewness |
|  |  | wavelet-HHH_firstorder_TotalEnergy | wavelet-HHH_gldm_DependenceEntropy | wavelet-HHH_glrlm_RunEntropy |
|  |  | wavelet-HHH_glszm_GrayLevelNonUniformityNormalized | wavelet-HHH_glszm_SizeZoneNonUniformityNormalized | wavelet-HHH_glszm_SmallAreaEmphasis |
|  |  | wavelet-HHH_glszm_ZoneEntropy | wavelet-HHH_ngtdm_Contrast | wavelet-LLL_firstorder_10Percentile |
|  |  | wavelet-LLL_firstorder_InterquartileRange | wavelet-LLL_firstorder_Median | wavelet-LLL_glcm_Correlation |
|  |  | wavelet-LLL_glcm_Imc1 | wavelet-LLL_glcm_MaximumProbability | wavelet-LLL_gldm_DependenceEntropy |
|  |  | wavelet-LLL_gldm_LargeDependenceHighGrayLevelEmphasis | wavelet-LLL_gldm_LargeDependenceLowGrayLevelEmphasis | wavelet-LLL_glrlm_ShortRunLowGrayLevelEmphasis |
|  |  | wavelet-LLL_glszm_GrayLevelVariance | wavelet-LLL_glszm_LowGrayLevelZoneEmphasis | wavelet-LLL_glszm_SizeZoneNonUniformityNormalized |
|  |  | wavelet-LLL_glszm_SmallAreaLowGrayLevelEmphasis | wavelet-LLL_glszm_ZoneVariance |  |
| Selection_18 | 0.00019 | original_shape_Elongation | original_shape_Flatness | original_shape_Maximum2DDiameterSlice |
|  |  | original_shape_Maximum3DDiameter | original_shape_MinorAxisLength | original_shape_Sphericity |
|  |  | original_firstorder_Kurtosis | original_glcm_Correlation | original_glcm_Idn |
|  |  | original_glcm_Imc2 | original_glcm_InverseVariance | original_glcm_JointAverage |
|  |  | original_glcm_MCC | original_glcm_SumAverage | original_glrlm_GrayLevelNonUniformity |
|  |  | original_glrlm_LongRunHighGrayLevelEmphasis | original_glszm_SmallAreaEmphasis | original_glszm_SmallAreaLowGrayLevelEmphasis |
|  |  | original_ngtdm_Busyness | original_ngtdm_Contrast | original_ngtdm_Strength |
|  |  | wavelet-LLH_firstorder_10Percentile | wavelet-LLH_firstorder_Kurtosis | wavelet-LLH_firstorder_RootMeanSquared |
|  |  | wavelet-LLH_firstorder_Skewness | wavelet-LLH_glcm_Idn | wavelet-LLH_glcm_JointAverage |
|  |  | wavelet-LLH_glcm_MCC | wavelet-LLH_glcm_MaximumProbability | wavelet-LLH_glcm_SumAverage |
|  |  | wavelet-LLH_gldm_DependenceVariance | wavelet-LLH_gldm_SmallDependenceLowGrayLevelEmphasis | wavelet-LLH_glrlm_LongRunEmphasis |
|  |  | wavelet-LLH_glrlm_ShortRunEmphasis | wavelet-LLH_glszm_SizeZoneNonUniformity | wavelet-LLH_glszm_SizeZoneNonUniformityNormalized |
|  |  | wavelet-LLH_glszm_SmallAreaLowGrayLevelEmphasis | wavelet-LLH_glszm_ZoneEntropy | wavelet-LLH_ngtdm_Busyness |
|  |  | wavelet-LHL_firstorder_Mean | wavelet-LHL_glcm_Correlation | wavelet-LHL_glcm_Idmn |
|  |  | wavelet-LHL_glcm_Idn | wavelet-LHL_glcm_Imc2 | wavelet-LHL_glcm_MaximumProbability |
|  |  | wavelet-LHL_gldm_DependenceEntropy | wavelet-LHL_glszm_GrayLevelNonUniformityNormalized | wavelet-LHL_glszm_LargeAreaHighGrayLevelEmphasis |
|  |  | wavelet-LHL_glszm_SizeZoneNonUniformityNormalized | wavelet-LHH_firstorder_Mean | wavelet-LHH_glcm_ClusterShade |
|  |  | wavelet-LHH_glcm_Idmn | wavelet-LHH_glcm_MCC | wavelet-LHH_glcm_MaximumProbability |
|  |  | wavelet-LHH_glrlm_RunEntropy | wavelet-LHH_glszm_GrayLevelNonUniformityNormalized | wavelet-LHH_glszm_LowGrayLevelZoneEmphasis |
|  |  | wavelet-LHH_glszm_SizeZoneNonUniformityNormalized | wavelet-LHH_glszm_SmallAreaLowGrayLevelEmphasis | wavelet-LHH_glszm_ZoneEntropy |
|  |  | wavelet-LHH_ngtdm_Contrast | wavelet-HLL_firstorder_Kurtosis | wavelet-HLL_firstorder_Maximum |
|  |  | wavelet-HLL_firstorder_Median | wavelet-HLL_firstorder_Skewness | wavelet-HLL_glcm_Correlation |
|  |  | wavelet-HLL_glcm_Idn | wavelet-HLL_glcm_Imc2 | wavelet-HLL_glrlm_LongRunHighGrayLevelEmphasis |
|  |  | wavelet-HLL_glrlm_LongRunLowGrayLevelEmphasis | wavelet-HLL_glrlm_RunVariance | wavelet-HLL_glszm_GrayLevelNonUniformity |
|  |  | wavelet-HLL_glszm_GrayLevelNonUniformityNormalized | wavelet-HLL_glszm_GrayLevelVariance | wavelet-HLL_glszm_LargeAreaEmphasis |
|  |  | wavelet-HLL_glszm_SmallAreaEmphasis | wavelet-HLL_glszm_SmallAreaLowGrayLevelEmphasis | wavelet-HLL_glszm_ZoneEntropy |
|  |  | wavelet-HLL_glszm_ZoneVariance | wavelet-HLL_ngtdm_Strength | wavelet-HLH_firstorder_Kurtosis |
|  |  | wavelet-HLH_firstorder_Median | wavelet-HLH_glcm_Correlation | wavelet-HLH_glcm_Idn |
|  |  | wavelet-HLH_glcm_InverseVariance | wavelet-HLH_glcm_MCC | wavelet-HLH_glcm_MaximumProbability |
|  |  | wavelet-HLH_glrlm_ShortRunLowGrayLevelEmphasis | wavelet-HLH_glszm_GrayLevelNonUniformityNormalized | wavelet-HLH_glszm_SmallAreaEmphasis |
|  |  | wavelet-HLH_glszm_SmallAreaLowGrayLevelEmphasis | wavelet-HLH_glszm_ZoneEntropy | wavelet-HHL_firstorder_Kurtosis |
|  |  | wavelet-HHL_firstorder_Maximum | wavelet-HHL_firstorder_Median | wavelet-HHL_firstorder_Skewness |
|  |  | wavelet-HHL_glcm_Imc1 | wavelet-HHL_glcm_MCC | wavelet-HHL_gldm_LargeDependenceHighGrayLevelEmphasis |
|  |  | wavelet-HHL_glrlm_ShortRunLowGrayLevelEmphasis | wavelet-HHL_glszm_GrayLevelNonUniformityNormalized | wavelet-HHL_glszm_LargeAreaEmphasis |
|  |  | wavelet-HHL_glszm_SmallAreaEmphasis | wavelet-HHL_glszm_SmallAreaLowGrayLevelEmphasis | wavelet-HHL_glszm_ZoneEntropy |
|  |  | wavelet-HHL_glszm_ZoneVariance | wavelet-HHL_ngtdm_Busyness | wavelet-HHL_ngtdm_Contrast |
|  |  | wavelet-HHH_firstorder_Kurtosis | wavelet-HHH_firstorder_Mean | wavelet-HHH_firstorder_Skewness |
|  |  | wavelet-HHH_firstorder_TotalEnergy | wavelet-HHH_gldm_DependenceEntropy | wavelet-HHH_glrlm_RunEntropy |
|  |  | wavelet-HHH_glszm_GrayLevelNonUniformityNormalized | wavelet-HHH_glszm_SizeZoneNonUniformityNormalized | wavelet-HHH_glszm_SmallAreaEmphasis |
|  |  | wavelet-HHH_glszm_ZoneEntropy | wavelet-HHH_ngtdm_Contrast | wavelet-LLL_firstorder_10Percentile |
|  |  | wavelet-LLL_firstorder_InterquartileRange | wavelet-LLL_firstorder_Median | wavelet-LLL_glcm_Correlation |
|  |  | wavelet-LLL_glcm_Imc1 | wavelet-LLL_glcm_MCC | wavelet-LLL_glcm_MaximumProbability |
|  |  | wavelet-LLL_gldm_DependenceEntropy | wavelet-LLL_gldm_LargeDependenceHighGrayLevelEmphasis | wavelet-LLL_gldm_LargeDependenceLowGrayLevelEmphasis |
|  |  | wavelet-LLL_gldm_SmallDependenceLowGrayLevelEmphasis | wavelet-LLL_glrlm_ShortRunLowGrayLevelEmphasis | wavelet-LLL_glszm_GrayLevelVariance |
|  |  | wavelet-LLL_glszm_LowGrayLevelZoneEmphasis | wavelet-LLL_glszm_SizeZoneNonUniformityNormalized | wavelet-LLL_glszm_SmallAreaLowGrayLevelEmphasis |
|  |  | wavelet-LLL_glszm_ZoneVariance |  |  |
| Selection_19 | 0.00014 | original_shape_Elongation | original_shape_Flatness | original_shape_Maximum2DDiameterSlice |
|  |  | original_shape_Maximum3DDiameter | original_shape_MinorAxisLength | original_shape_Sphericity |
|  |  | original_firstorder_Kurtosis | original_glcm_Correlation | original_glcm_Idn |
|  |  | original_glcm_Imc2 | original_glcm_InverseVariance | original_glcm_JointAverage |
|  |  | original_glcm_MCC | original_glcm_MaximumProbability | original_glcm_SumAverage |
|  |  | original_glrlm_GrayLevelNonUniformity | original_glrlm_LongRunHighGrayLevelEmphasis | original_glszm_SmallAreaEmphasis |
|  |  | original_glszm_SmallAreaLowGrayLevelEmphasis | original_ngtdm_Busyness | original_ngtdm_Contrast |
|  |  | original_ngtdm_Strength | wavelet-LLH_firstorder_10Percentile | wavelet-LLH_firstorder_Kurtosis |
|  |  | wavelet-LLH_firstorder_RootMeanSquared | wavelet-LLH_firstorder_Skewness | wavelet-LLH_glcm_Idn |
|  |  | wavelet-LLH_glcm_JointAverage | wavelet-LLH_glcm_MCC | wavelet-LLH_glcm_MaximumProbability |
|  |  | wavelet-LLH_glcm_SumAverage | wavelet-LLH_gldm_DependenceVariance | wavelet-LLH_gldm_SmallDependenceLowGrayLevelEmphasis |
|  |  | wavelet-LLH_glrlm_LongRunEmphasis | wavelet-LLH_glrlm_ShortRunEmphasis | wavelet-LLH_glszm_GrayLevelNonUniformityNormalized |
|  |  | wavelet-LLH_glszm_SizeZoneNonUniformity | wavelet-LLH_glszm_SizeZoneNonUniformityNormalized | wavelet-LLH_glszm_SmallAreaLowGrayLevelEmphasis |
|  |  | wavelet-LLH_glszm_ZoneEntropy | wavelet-LLH_ngtdm_Busyness | wavelet-LHL_firstorder_Mean |
|  |  | wavelet-LHL_glcm_Correlation | wavelet-LHL_glcm_Idmn | wavelet-LHL_glcm_Idn |
|  |  | wavelet-LHL_glcm_Imc2 | wavelet-LHL_glcm_MaximumProbability | wavelet-LHL_gldm_DependenceEntropy |
|  |  | wavelet-LHL_glszm_GrayLevelNonUniformity | wavelet-LHL_glszm_GrayLevelNonUniformityNormalized | wavelet-LHL_glszm_LargeAreaHighGrayLevelEmphasis |
|  |  | wavelet-LHL_glszm_SizeZoneNonUniformityNormalized | wavelet-LHH_firstorder_Mean | wavelet-LHH_glcm_ClusterShade |
|  |  | wavelet-LHH_glcm_Idmn | wavelet-LHH_glcm_MCC | wavelet-LHH_glcm_MaximumProbability |
|  |  | wavelet-LHH_glrlm_RunEntropy | wavelet-LHH_glszm_GrayLevelNonUniformityNormalized | wavelet-LHH_glszm_LowGrayLevelZoneEmphasis |
|  |  | wavelet-LHH_glszm_SizeZoneNonUniformityNormalized | wavelet-LHH_glszm_SmallAreaLowGrayLevelEmphasis | wavelet-LHH_glszm_ZoneEntropy |
|  |  | wavelet-LHH_ngtdm_Contrast | wavelet-HLL_firstorder_Kurtosis | wavelet-HLL_firstorder_Maximum |
|  |  | wavelet-HLL_firstorder_Median | wavelet-HLL_firstorder_Skewness | wavelet-HLL_glcm_Correlation |
|  |  | wavelet-HLL_glcm_Idn | wavelet-HLL_glcm_Imc2 | wavelet-HLL_glrlm_LongRunHighGrayLevelEmphasis |
|  |  | wavelet-HLL_glrlm_LongRunLowGrayLevelEmphasis | wavelet-HLL_glrlm_RunVariance | wavelet-HLL_glszm_GrayLevelNonUniformity |
|  |  | wavelet-HLL_glszm_GrayLevelNonUniformityNormalized | wavelet-HLL_glszm_GrayLevelVariance | wavelet-HLL_glszm_LargeAreaEmphasis |
|  |  | wavelet-HLL_glszm_SmallAreaEmphasis | wavelet-HLL_glszm_SmallAreaLowGrayLevelEmphasis | wavelet-HLL_glszm_ZoneEntropy |
|  |  | wavelet-HLL_glszm_ZoneVariance | wavelet-HLL_ngtdm_Strength | wavelet-HLH_firstorder_Kurtosis |
|  |  | wavelet-HLH_firstorder_Median | wavelet-HLH_glcm_Correlation | wavelet-HLH_glcm_Idn |
|  |  | wavelet-HLH_glcm_InverseVariance | wavelet-HLH_glcm_MCC | wavelet-HLH_glcm_MaximumProbability |
|  |  | wavelet-HLH_glrlm_ShortRunLowGrayLevelEmphasis | wavelet-HLH_glszm_GrayLevelNonUniformity | wavelet-HLH_glszm_GrayLevelNonUniformityNormalized |
|  |  | wavelet-HLH_glszm_SmallAreaEmphasis | wavelet-HLH_glszm_SmallAreaLowGrayLevelEmphasis | wavelet-HLH_glszm_ZoneEntropy |
|  |  | wavelet-HHL_firstorder_Kurtosis | wavelet-HHL_firstorder_Maximum | wavelet-HHL_firstorder_Median |
|  |  | wavelet-HHL_firstorder_Skewness | wavelet-HHL_glcm_Imc1 | wavelet-HHL_glcm_MCC |
|  |  | wavelet-HHL_gldm_LargeDependenceHighGrayLevelEmphasis | wavelet-HHL_glrlm_ShortRunLowGrayLevelEmphasis | wavelet-HHL_glszm_GrayLevelNonUniformityNormalized |
|  |  | wavelet-HHL_glszm_LargeAreaEmphasis | wavelet-HHL_glszm_SmallAreaEmphasis | wavelet-HHL_glszm_SmallAreaLowGrayLevelEmphasis |
|  |  | wavelet-HHL_glszm_ZoneEntropy | wavelet-HHL_glszm_ZoneVariance | wavelet-HHL_ngtdm_Busyness |
|  |  | wavelet-HHL_ngtdm_Contrast | wavelet-HHH_firstorder_Kurtosis | wavelet-HHH_firstorder_Mean |
|  |  | wavelet-HHH_firstorder_Skewness | wavelet-HHH_firstorder_TotalEnergy | wavelet-HHH_gldm_DependenceEntropy |
|  |  | wavelet-HHH_glrlm_RunEntropy | wavelet-HHH_glrlm_ShortRunLowGrayLevelEmphasis | wavelet-HHH_glszm_GrayLevelNonUniformityNormalized |
|  |  | wavelet-HHH_glszm_SizeZoneNonUniformityNormalized | wavelet-HHH_glszm_SmallAreaEmphasis | wavelet-HHH_glszm_ZoneEntropy |
|  |  | wavelet-HHH_ngtdm_Contrast | wavelet-LLL_firstorder_10Percentile | wavelet-LLL_firstorder_InterquartileRange |
|  |  | wavelet-LLL_firstorder_Median | wavelet-LLL_glcm_Correlation | wavelet-LLL_glcm_Imc1 |
|  |  | wavelet-LLL_glcm_InverseVariance | wavelet-LLL_glcm_MCC | wavelet-LLL_glcm_MaximumProbability |
|  |  | wavelet-LLL_gldm_DependenceEntropy | wavelet-LLL_gldm_LargeDependenceHighGrayLevelEmphasis | wavelet-LLL_gldm_LargeDependenceLowGrayLevelEmphasis |
|  |  | wavelet-LLL_gldm_SmallDependenceLowGrayLevelEmphasis | wavelet-LLL_glrlm_ShortRunLowGrayLevelEmphasis | wavelet-LLL_glszm_GrayLevelVariance |
|  |  | wavelet-LLL_glszm_LowGrayLevelZoneEmphasis | wavelet-LLL_glszm_SizeZoneNonUniformityNormalized | wavelet-LLL_glszm_SmallAreaLowGrayLevelEmphasis |
|  |  | wavelet-LLL_glszm_ZoneVariance |  |  |
| Selection_20 | 0.00010 | original_shape_Elongation | original_shape_Flatness | original_shape_MajorAxisLength |
|  |  | original_shape_Maximum2DDiameterSlice | original_shape_Maximum3DDiameter | original_shape_MinorAxisLength |
|  |  | original_shape_Sphericity | original_firstorder_Kurtosis | original_glcm_Correlation |
|  |  | original_glcm_Idn | original_glcm_Imc2 | original_glcm_InverseVariance |
|  |  | original_glcm_JointAverage | original_glcm_MCC | original_glcm_MaximumProbability |
|  |  | original_glcm_SumAverage | original_gldm_DependenceNonUniformityNormalized | original_glrlm_GrayLevelNonUniformity |
|  |  | original_glrlm_LongRunHighGrayLevelEmphasis | original_glszm_SmallAreaEmphasis | original_glszm_SmallAreaLowGrayLevelEmphasis |
|  |  | original_ngtdm_Busyness | original_ngtdm_Contrast | original_ngtdm_Strength |
|  |  | wavelet-LLH_firstorder_10Percentile | wavelet-LLH_firstorder_Kurtosis | wavelet-LLH_firstorder_RootMeanSquared |
|  |  | wavelet-LLH_firstorder_Skewness | wavelet-LLH_glcm_Idn | wavelet-LLH_glcm_JointAverage |
|  |  | wavelet-LLH_glcm_MCC | wavelet-LLH_glcm_MaximumProbability | wavelet-LLH_glcm_SumAverage |
|  |  | wavelet-LLH_gldm_DependenceVariance | wavelet-LLH_gldm_SmallDependenceLowGrayLevelEmphasis | wavelet-LLH_glrlm_LongRunEmphasis |
|  |  | wavelet-LLH_glrlm_ShortRunEmphasis | wavelet-LLH_glszm_GrayLevelNonUniformityNormalized | wavelet-LLH_glszm_SizeZoneNonUniformity |
|  |  | wavelet-LLH_glszm_SizeZoneNonUniformityNormalized | wavelet-LLH_glszm_SmallAreaLowGrayLevelEmphasis | wavelet-LLH_glszm_ZoneEntropy |
|  |  | wavelet-LLH_ngtdm_Busyness | wavelet-LHL_firstorder_Mean | wavelet-LHL_glcm_Correlation |
|  |  | wavelet-LHL_glcm_Idmn | wavelet-LHL_glcm_Idn | wavelet-LHL_glcm_Imc2 |
|  |  | wavelet-LHL_glcm_MaximumProbability | wavelet-LHL_gldm_DependenceEntropy | wavelet-LHL_glszm_GrayLevelNonUniformity |
|  |  | wavelet-LHL_glszm_GrayLevelNonUniformityNormalized | wavelet-LHL_glszm_LargeAreaHighGrayLevelEmphasis | wavelet-LHL_glszm_SizeZoneNonUniformityNormalized |
|  |  | wavelet-LHL_ngtdm_Strength | wavelet-LHH_firstorder_Mean | wavelet-LHH_glcm_ClusterShade |
|  |  | wavelet-LHH_glcm_Idmn | wavelet-LHH_glcm_MCC | wavelet-LHH_glcm_MaximumProbability |
|  |  | wavelet-LHH_glrlm_RunEntropy | wavelet-LHH_glszm_GrayLevelNonUniformityNormalized | wavelet-LHH_glszm_LowGrayLevelZoneEmphasis |
|  |  | wavelet-LHH_glszm_SizeZoneNonUniformityNormalized | wavelet-LHH_glszm_SmallAreaLowGrayLevelEmphasis | wavelet-LHH_glszm_ZoneEntropy |
|  |  | wavelet-LHH_ngtdm_Contrast | wavelet-HLL_firstorder_Kurtosis | wavelet-HLL_firstorder_Maximum |
|  |  | wavelet-HLL_firstorder_Median | wavelet-HLL_firstorder_Skewness | wavelet-HLL_glcm_Correlation |
|  |  | wavelet-HLL_glcm_Idn | wavelet-HLL_glcm_Imc2 | wavelet-HLL_glrlm_LongRunHighGrayLevelEmphasis |
|  |  | wavelet-HLL_glrlm_LongRunLowGrayLevelEmphasis | wavelet-HLL_glrlm_RunVariance | wavelet-HLL_glszm_GrayLevelNonUniformity |
|  |  | wavelet-HLL_glszm_GrayLevelNonUniformityNormalized | wavelet-HLL_glszm_GrayLevelVariance | wavelet-HLL_glszm_LargeAreaEmphasis |
|  |  | wavelet-HLL_glszm_SmallAreaEmphasis | wavelet-HLL_glszm_SmallAreaLowGrayLevelEmphasis | wavelet-HLL_glszm_ZoneEntropy |
|  |  | wavelet-HLL_glszm_ZoneVariance | wavelet-HLL_ngtdm_Strength | wavelet-HLH_firstorder_Kurtosis |
|  |  | wavelet-HLH_firstorder_Median | wavelet-HLH_glcm_Correlation | wavelet-HLH_glcm_Idn |
|  |  | wavelet-HLH_glcm_InverseVariance | wavelet-HLH_glcm_MCC | wavelet-HLH_glcm_MaximumProbability |
|  |  | wavelet-HLH_glrlm_RunVariance | wavelet-HLH_glrlm_ShortRunLowGrayLevelEmphasis | wavelet-HLH_glszm_GrayLevelNonUniformity |
|  |  | wavelet-HLH_glszm_GrayLevelNonUniformityNormalized | wavelet-HLH_glszm_SmallAreaEmphasis | wavelet-HLH_glszm_SmallAreaLowGrayLevelEmphasis |
|  |  | wavelet-HLH_glszm_ZoneEntropy | wavelet-HHL_firstorder_Kurtosis | wavelet-HHL_firstorder_Maximum |
|  |  | wavelet-HHL_firstorder_Median | wavelet-HHL_firstorder_Skewness | wavelet-HHL_glcm_Imc1 |
|  |  | wavelet-HHL_glcm_MCC | wavelet-HHL_gldm_LargeDependenceHighGrayLevelEmphasis | wavelet-HHL_glrlm_ShortRunLowGrayLevelEmphasis |
|  |  | wavelet-HHL_glszm_GrayLevelNonUniformityNormalized | wavelet-HHL_glszm_LargeAreaEmphasis | wavelet-HHL_glszm_SmallAreaEmphasis |
|  |  | wavelet-HHL_glszm_SmallAreaLowGrayLevelEmphasis | wavelet-HHL_glszm_ZoneEntropy | wavelet-HHL_glszm_ZoneVariance |
|  |  | wavelet-HHL_ngtdm_Busyness | wavelet-HHL_ngtdm_Contrast | wavelet-HHH_firstorder_Kurtosis |
|  |  | wavelet-HHH_firstorder_Mean | wavelet-HHH_firstorder_Skewness | wavelet-HHH_firstorder_TotalEnergy |
|  |  | wavelet-HHH_gldm_DependenceEntropy | wavelet-HHH_glrlm_RunEntropy | wavelet-HHH_glrlm_ShortRunLowGrayLevelEmphasis |
|  |  | wavelet-HHH_glszm_GrayLevelNonUniformityNormalized | wavelet-HHH_glszm_SizeZoneNonUniformityNormalized | wavelet-HHH_glszm_SmallAreaEmphasis |
|  |  | wavelet-HHH_glszm_ZoneEntropy | wavelet-HHH_ngtdm_Contrast | wavelet-LLL_firstorder_10Percentile |
|  |  | wavelet-LLL_firstorder_InterquartileRange | wavelet-LLL_firstorder_Median | wavelet-LLL_glcm_Correlation |
|  |  | wavelet-LLL_glcm_Imc1 | wavelet-LLL_glcm_InverseVariance | wavelet-LLL_glcm_MCC |
|  |  | wavelet-LLL_glcm_MaximumProbability | wavelet-LLL_gldm_DependenceEntropy | wavelet-LLL_gldm_LargeDependenceHighGrayLevelEmphasis |
|  |  | wavelet-LLL_gldm_LargeDependenceLowGrayLevelEmphasis | wavelet-LLL_gldm_SmallDependenceLowGrayLevelEmphasis | wavelet-LLL_glrlm_ShortRunLowGrayLevelEmphasis |
|  |  | wavelet-LLL_glszm_GrayLevelVariance | wavelet-LLL_glszm_LowGrayLevelZoneEmphasis | wavelet-LLL_glszm_SizeZoneNonUniformityNormalized |
|  |  | wavelet-LLL_glszm_SmallAreaLowGrayLevelEmphasis | wavelet-LLL_glszm_ZoneVariance |  |
