# Supplementary material for: Clinical radiomics-based machine learning versus three-dimension convolutional neural network analysis for differentiation of thymic epithelial tumors from other prevascular mediastinal tumors on chest computed tomography scan
Source: Front Oncol. 2023 Apr 18;13:1105100. doi: 10.3389/fonc.2023.1105100 (PMC10151670; doi:10.3389/fonc.2023.1105100)
Supplement: Supplementary file 4 [file Table_4.docx]

**Supplementary Table 4. Top 20 feature selection with Lasso Logistic Regression in CECT**

| **Selection Group** | **Lambda** | **Selected Variables** | | |
| --- | --- | --- | --- | --- |
| Selection_1 | 0.04863 | wavelet-LHH_firstorder_Mean | wavelet-LHH_glcm_SumEntropy | wavelet-LHH_gldm_LargeDependenceEmphasis |
|  |  | wavelet-LHH_ngtdm_Busyness | wavelet-HLH_glszm_SizeZoneNonUniformityNormalized | wavelet-HLH_ngtdm_Busyness |
|  |  | wavelet-LLL_firstorder_Median |  |  |
| Selection_2 | 0.03511 | wavelet-LHL_firstorder_Mean | wavelet-LHH_firstorder_Mean | wavelet-LHH_glcm_SumEntropy |
|  |  | wavelet-LHH_glrlm_RunLengthNonUniformityNormalized | wavelet-LHH_glrlm_RunPercentage | wavelet-LHH_ngtdm_Busyness |
|  |  | wavelet-HLL_glszm_LargeAreaHighGrayLevelEmphasis | wavelet-HLH_glszm_SizeZoneNonUniformityNormalized | wavelet-HLH_ngtdm_Busyness |
|  |  | wavelet-HHL_glcm_Imc1 | wavelet-HHL_glcm_InverseVariance | wavelet-LLL_firstorder_Median |
| Selection_3 | 0.02535 | wavelet-LHL_firstorder_Mean | wavelet-LHL_firstorder_Skewness | wavelet-LHH_firstorder_Mean |
|  |  | wavelet-LHH_glcm_JointEnergy | wavelet-LHH_glcm_SumEntropy | wavelet-LHH_glrlm_RunPercentage |
|  |  | wavelet-LHH_glszm_SizeZoneNonUniformityNormalized | wavelet-LHH_ngtdm_Busyness | wavelet-HLL_firstorder_90Percentile |
|  |  | wavelet-HLL_glszm_LargeAreaHighGrayLevelEmphasis | wavelet-HLH_glszm_SizeZoneNonUniformityNormalized | wavelet-HLH_ngtdm_Busyness |
|  |  | wavelet-HHL_glcm_Imc1 | wavelet-HHL_glcm_InverseVariance | wavelet-HHL_glcm_MCC |
|  |  | wavelet-LLL_firstorder_Median |  |  |
| Selection_4 | 0.01831 | wavelet-LLH_glszm_GrayLevelVariance | wavelet-LLH_glszm_LowGrayLevelZoneEmphasis | wavelet-LHL_firstorder_Mean |
|  |  | wavelet-LHL_firstorder_Skewness | wavelet-LHL_glcm_InverseVariance | wavelet-LHH_firstorder_Mean |
|  |  | wavelet-LHH_glcm_Imc2 | wavelet-LHH_glcm_JointEnergy | wavelet-LHH_glcm_SumEntropy |
|  |  | wavelet-LHH_glrlm_RunLengthNonUniformityNormalized | wavelet-LHH_glrlm_RunPercentage | wavelet-LHH_glszm_SizeZoneNonUniformityNormalized |
|  |  | wavelet-LHH_ngtdm_Busyness | wavelet-HLL_firstorder_90Percentile | wavelet-HLL_glcm_Correlation |
|  |  | wavelet-HLH_glszm_SizeZoneNonUniformityNormalized | wavelet-HLH_ngtdm_Busyness | wavelet-HHL_glcm_Imc1 |
|  |  | wavelet-HHL_glcm_InverseVariance | wavelet-HHL_glcm_MCC | wavelet-HHL_glszm_LargeAreaHighGrayLevelEmphasis |
|  |  | wavelet-HHL_glszm_SmallAreaLowGrayLevelEmphasis | wavelet-LLL_firstorder_Median |  |
| Selection_5 | 0.01322 | original_shape_Sphericity | wavelet-LLH_glszm_GrayLevelVariance | wavelet-LLH_glszm_LowGrayLevelZoneEmphasis |
|  |  | wavelet-LHL_firstorder_Maximum | wavelet-LHL_firstorder_Mean | wavelet-LHL_firstorder_Median |
|  |  | wavelet-LHL_firstorder_Skewness | wavelet-LHL_glcm_InverseVariance | wavelet-LHH_firstorder_Mean |
|  |  | wavelet-LHH_glcm_Imc2 | wavelet-LHH_glcm_JointEnergy | wavelet-LHH_glcm_SumEntropy |
|  |  | wavelet-LHH_glrlm_RunLengthNonUniformityNormalized | wavelet-LHH_glrlm_RunPercentage | wavelet-LHH_glszm_SizeZoneNonUniformityNormalized |
|  |  | wavelet-LHH_ngtdm_Busyness | wavelet-HLL_firstorder_90Percentile | wavelet-HLL_glcm_Correlation |
|  |  | wavelet-HLH_glcm_MCC | wavelet-HLH_glrlm_LongRunLowGrayLevelEmphasis | wavelet-HLH_glszm_GrayLevelNonUniformityNormalized |
|  |  | wavelet-HLH_glszm_SizeZoneNonUniformityNormalized | wavelet-HLH_ngtdm_Busyness | wavelet-HHL_glcm_Imc1 |
|  |  | wavelet-HHL_glcm_InverseVariance | wavelet-HHL_glcm_MCC | wavelet-HHL_glcm_MaximumProbability |
|  |  | wavelet-HHL_glszm_LargeAreaHighGrayLevelEmphasis | wavelet-HHL_glszm_SmallAreaLowGrayLevelEmphasis | wavelet-HHH_firstorder_Median |
|  |  | wavelet-LLL_firstorder_Median |  |  |
| Selection_6 | 0.00955 | original_shape_Sphericity | original_glcm_DifferenceVariance | wavelet-LLH_firstorder_90Percentile |
|  |  | wavelet-LLH_glszm_GrayLevelVariance | wavelet-LLH_glszm_LowGrayLevelZoneEmphasis | wavelet-LLH_glszm_SmallAreaEmphasis |
|  |  | wavelet-LLH_ngtdm_Strength | wavelet-LHL_firstorder_Maximum | wavelet-LHL_firstorder_Mean |
|  |  | wavelet-LHL_firstorder_Median | wavelet-LHL_firstorder_Skewness | wavelet-LHL_glcm_InverseVariance |
|  |  | wavelet-LHL_glcm_MCC | wavelet-LHH_firstorder_Mean | wavelet-LHH_glcm_JointEnergy |
|  |  | wavelet-LHH_glcm_SumEntropy | wavelet-LHH_glrlm_RunPercentage | wavelet-LHH_glszm_SizeZoneNonUniformityNormalized |
|  |  | wavelet-LHH_ngtdm_Busyness | wavelet-HLL_firstorder_90Percentile | wavelet-HLL_glcm_Correlation |
|  |  | wavelet-HLL_glcm_Id | wavelet-HLL_ngtdm_Complexity | wavelet-HLH_glcm_ClusterProminence |
|  |  | wavelet-HLH_glcm_MCC | wavelet-HLH_glrlm_LongRunLowGrayLevelEmphasis | wavelet-HLH_glszm_GrayLevelNonUniformityNormalized |
|  |  | wavelet-HLH_glszm_SizeZoneNonUniformityNormalized | wavelet-HLH_glszm_ZoneEntropy | wavelet-HLH_ngtdm_Busyness |
|  |  | wavelet-HHL_glcm_Imc1 | wavelet-HHL_glcm_InverseVariance | wavelet-HHL_glcm_MCC |
|  |  | wavelet-HHL_glcm_MaximumProbability | wavelet-HHL_glrlm_ShortRunLowGrayLevelEmphasis | wavelet-HHL_glszm_LargeAreaHighGrayLevelEmphasis |
|  |  | wavelet-HHL_glszm_SmallAreaLowGrayLevelEmphasis | wavelet-HHH_firstorder_Median | wavelet-HHH_glszm_LowGrayLevelZoneEmphasis |
|  |  | wavelet-LLL_firstorder_Median | wavelet-LLL_glcm_Idmn | wavelet-LLL_glszm_SmallAreaEmphasis |
| Selection_7 | 0.00689 | original_shape_Sphericity | original_glcm_DifferenceVariance | wavelet-LLH_firstorder_90Percentile |
|  |  | wavelet-LLH_glszm_LowGrayLevelZoneEmphasis | wavelet-LLH_glszm_SmallAreaEmphasis | wavelet-LLH_ngtdm_Strength |
|  |  | wavelet-LHL_firstorder_Maximum | wavelet-LHL_firstorder_Mean | wavelet-LHL_firstorder_Median |
|  |  | wavelet-LHL_firstorder_Skewness | wavelet-LHL_glcm_InverseVariance | wavelet-LHL_glcm_MCC |
|  |  | wavelet-LHH_firstorder_Mean | wavelet-LHH_glcm_JointEnergy | wavelet-LHH_glcm_SumEntropy |
|  |  | wavelet-LHH_gldm_LargeDependenceLowGrayLevelEmphasis | wavelet-LHH_glrlm_RunPercentage | wavelet-LHH_glszm_GrayLevelNonUniformityNormalized |
|  |  | wavelet-LHH_glszm_GrayLevelVariance | wavelet-LHH_glszm_LargeAreaHighGrayLevelEmphasis | wavelet-LHH_glszm_SizeZoneNonUniformityNormalized |
|  |  | wavelet-LHH_ngtdm_Busyness | wavelet-HLL_firstorder_90Percentile | wavelet-HLL_firstorder_Kurtosis |
|  |  | wavelet-HLL_glcm_Correlation | wavelet-HLL_glcm_DifferenceVariance | wavelet-HLL_glcm_Id |
|  |  | wavelet-HLL_ngtdm_Complexity | wavelet-HLH_glcm_ClusterProminence | wavelet-HLH_glcm_MCC |
|  |  | wavelet-HLH_glrlm_LongRunLowGrayLevelEmphasis | wavelet-HLH_glszm_GrayLevelNonUniformityNormalized | wavelet-HLH_glszm_SizeZoneNonUniformityNormalized |
|  |  | wavelet-HLH_glszm_ZoneEntropy | wavelet-HLH_ngtdm_Busyness | wavelet-HHL_glcm_Imc1 |
|  |  | wavelet-HHL_glcm_InverseVariance | wavelet-HHL_glcm_MCC | wavelet-HHL_glcm_MaximumProbability |
|  |  | wavelet-HHL_glrlm_RunEntropy | wavelet-HHL_glrlm_ShortRunLowGrayLevelEmphasis | wavelet-HHL_glszm_GrayLevelNonUniformityNormalized |
|  |  | wavelet-HHL_glszm_LargeAreaHighGrayLevelEmphasis | wavelet-HHL_glszm_SmallAreaEmphasis | wavelet-HHL_glszm_SmallAreaLowGrayLevelEmphasis |
|  |  | wavelet-HHH_firstorder_Median | wavelet-HHH_glszm_LowGrayLevelZoneEmphasis | wavelet-HHH_glszm_ZoneEntropy |
|  |  | wavelet-LLL_firstorder_Median | wavelet-LLL_glcm_Correlation | wavelet-LLL_glcm_Idmn |
|  |  | wavelet-LLL_glszm_SmallAreaEmphasis |  |  |
| Selection_8 | 0.00498 | original_shape_Sphericity | original_glcm_DifferenceVariance | original_glszm_SizeZoneNonUniformity |
|  |  | wavelet-LLH_firstorder_90Percentile | wavelet-LLH_gldm_SmallDependenceLowGrayLevelEmphasis | wavelet-LLH_glszm_LowGrayLevelZoneEmphasis |
|  |  | wavelet-LLH_glszm_SmallAreaEmphasis | wavelet-LLH_ngtdm_Busyness | wavelet-LLH_ngtdm_Strength |
|  |  | wavelet-LHL_firstorder_Maximum | wavelet-LHL_firstorder_Mean | wavelet-LHL_firstorder_Median |
|  |  | wavelet-LHL_firstorder_Skewness | wavelet-LHL_glcm_InverseVariance | wavelet-LHL_glcm_MCC |
|  |  | wavelet-LHL_glszm_LargeAreaLowGrayLevelEmphasis | wavelet-LHH_firstorder_Mean | wavelet-LHH_glcm_JointEnergy |
|  |  | wavelet-LHH_glcm_SumEntropy | wavelet-LHH_gldm_LargeDependenceLowGrayLevelEmphasis | wavelet-LHH_glrlm_RunPercentage |
|  |  | wavelet-LHH_glszm_GrayLevelNonUniformityNormalized | wavelet-LHH_glszm_GrayLevelVariance | wavelet-LHH_glszm_LargeAreaHighGrayLevelEmphasis |
|  |  | wavelet-LHH_glszm_SizeZoneNonUniformityNormalized | wavelet-LHH_ngtdm_Busyness | wavelet-HLL_firstorder_Kurtosis |
|  |  | wavelet-HLL_firstorder_Maximum | wavelet-HLL_glcm_Correlation | wavelet-HLL_glcm_DifferenceVariance |
|  |  | wavelet-HLL_glcm_Id | wavelet-HLL_ngtdm_Complexity | wavelet-HLH_glcm_ClusterProminence |
|  |  | wavelet-HLH_glcm_MCC | wavelet-HLH_glrlm_LongRunLowGrayLevelEmphasis | wavelet-HLH_glszm_SizeZoneNonUniformityNormalized |
|  |  | wavelet-HLH_glszm_ZoneEntropy | wavelet-HLH_ngtdm_Busyness | wavelet-HHL_glcm_Imc1 |
|  |  | wavelet-HHL_glcm_InverseVariance | wavelet-HHL_glcm_MCC | wavelet-HHL_glcm_MaximumProbability |
|  |  | wavelet-HHL_glrlm_RunEntropy | wavelet-HHL_glrlm_ShortRunLowGrayLevelEmphasis | wavelet-HHL_glszm_GrayLevelNonUniformityNormalized |
|  |  | wavelet-HHL_glszm_LargeAreaHighGrayLevelEmphasis | wavelet-HHL_glszm_SmallAreaEmphasis | wavelet-HHL_glszm_SmallAreaLowGrayLevelEmphasis |
|  |  | wavelet-HHL_glszm_ZoneEntropy | wavelet-HHL_ngtdm_Complexity | wavelet-HHH_firstorder_Median |
|  |  | wavelet-HHH_glszm_LowGrayLevelZoneEmphasis | wavelet-HHH_glszm_ZoneEntropy | wavelet-LLL_firstorder_Median |
|  |  | wavelet-LLL_glcm_Correlation | wavelet-LLL_glcm_DifferenceVariance | wavelet-LLL_gldm_DependenceEntropy |
|  |  | wavelet-LLL_glszm_SmallAreaEmphasis | wavelet-LLL_ngtdm_Strength |  |
| Selection_9 | 0.00359 | original_shape_Sphericity | original_shape_SurfaceArea | original_firstorder_Kurtosis |
|  |  | original_glcm_DifferenceVariance | original_glszm_GrayLevelNonUniformityNormalized | original_glszm_SizeZoneNonUniformity |
|  |  | original_glszm_SmallAreaEmphasis | wavelet-LLH_firstorder_90Percentile | wavelet-LLH_gldm_SmallDependenceLowGrayLevelEmphasis |
|  |  | wavelet-LLH_glszm_LowGrayLevelZoneEmphasis | wavelet-LLH_glszm_SmallAreaEmphasis | wavelet-LLH_ngtdm_Busyness |
|  |  | wavelet-LLH_ngtdm_Strength | wavelet-LHL_firstorder_Maximum | wavelet-LHL_firstorder_Mean |
|  |  | wavelet-LHL_firstorder_Median | wavelet-LHL_firstorder_Skewness | wavelet-LHL_glcm_InverseVariance |
|  |  | wavelet-LHL_glcm_MCC | wavelet-LHL_glszm_LargeAreaLowGrayLevelEmphasis | wavelet-LHL_glszm_ZoneEntropy |
|  |  | wavelet-LHL_ngtdm_Busyness | wavelet-LHH_firstorder_Mean | wavelet-LHH_glcm_SumEntropy |
|  |  | wavelet-LHH_gldm_LargeDependenceLowGrayLevelEmphasis | wavelet-LHH_glszm_GrayLevelVariance | wavelet-LHH_glszm_LargeAreaHighGrayLevelEmphasis |
|  |  | wavelet-LHH_glszm_SizeZoneNonUniformityNormalized | wavelet-LHH_ngtdm_Busyness | wavelet-HLL_firstorder_Kurtosis |
|  |  | wavelet-HLL_firstorder_Maximum | wavelet-HLL_firstorder_Median | wavelet-HLL_glcm_Correlation |
|  |  | wavelet-HLL_glcm_DifferenceVariance | wavelet-HLL_glcm_Id | wavelet-HLL_gldm_SmallDependenceLowGrayLevelEmphasis |
|  |  | wavelet-HLL_glszm_LargeAreaHighGrayLevelEmphasis | wavelet-HLL_glszm_ZoneEntropy | wavelet-HLL_ngtdm_Contrast |
|  |  | wavelet-HLH_glcm_MCC | wavelet-HLH_glrlm_LongRunLowGrayLevelEmphasis | wavelet-HLH_glszm_SizeZoneNonUniformityNormalized |
|  |  | wavelet-HLH_glszm_ZoneEntropy | wavelet-HLH_ngtdm_Busyness | wavelet-HHL_glcm_Imc1 |
|  |  | wavelet-HHL_glcm_InverseVariance | wavelet-HHL_glcm_MCC | wavelet-HHL_glcm_MaximumProbability |
|  |  | wavelet-HHL_glrlm_RunEntropy | wavelet-HHL_glrlm_ShortRunLowGrayLevelEmphasis | wavelet-HHL_glszm_GrayLevelNonUniformityNormalized |
|  |  | wavelet-HHL_glszm_LargeAreaHighGrayLevelEmphasis | wavelet-HHL_glszm_SmallAreaEmphasis | wavelet-HHL_glszm_SmallAreaLowGrayLevelEmphasis |
|  |  | wavelet-HHL_glszm_ZoneEntropy | wavelet-HHL_ngtdm_Busyness | wavelet-HHL_ngtdm_Complexity |
|  |  | wavelet-HHH_firstorder_Median | wavelet-HHH_glszm_LowGrayLevelZoneEmphasis | wavelet-HHH_glszm_ZoneEntropy |
|  |  | wavelet-LLL_firstorder_Median | wavelet-LLL_glcm_Correlation | wavelet-LLL_glcm_DifferenceVariance |
|  |  | wavelet-LLL_gldm_DependenceEntropy | wavelet-LLL_glszm_SmallAreaEmphasis | wavelet-LLL_ngtdm_Strength |
| Selection_10 | 0.00260 | original_shape_Elongation | original_shape_Sphericity | original_shape_SurfaceArea |
|  |  | original_firstorder_Kurtosis | original_glcm_DifferenceVariance | original_glszm_GrayLevelNonUniformityNormalized |
|  |  | original_glszm_SizeZoneNonUniformity | original_glszm_SmallAreaEmphasis | original_glszm_SmallAreaLowGrayLevelEmphasis |
|  |  | wavelet-LLH_firstorder_90Percentile | wavelet-LLH_gldm_SmallDependenceLowGrayLevelEmphasis | wavelet-LLH_glszm_LargeAreaHighGrayLevelEmphasis |
|  |  | wavelet-LLH_glszm_LowGrayLevelZoneEmphasis | wavelet-LLH_glszm_SmallAreaEmphasis | wavelet-LLH_ngtdm_Busyness |
|  |  | wavelet-LLH_ngtdm_Coarseness | wavelet-LLH_ngtdm_Strength | wavelet-LHL_firstorder_Maximum |
|  |  | wavelet-LHL_firstorder_Median | wavelet-LHL_firstorder_Skewness | wavelet-LHL_glcm_InverseVariance |
|  |  | wavelet-LHL_glcm_MCC | wavelet-LHL_glrlm_RunEntropy | wavelet-LHL_glszm_ZoneEntropy |
|  |  | wavelet-LHL_ngtdm_Busyness | wavelet-LHH_firstorder_Mean | wavelet-LHH_firstorder_Uniformity |
|  |  | wavelet-LHH_glcm_SumEntropy | wavelet-LHH_gldm_LargeDependenceLowGrayLevelEmphasis | wavelet-LHH_glszm_GrayLevelVariance |
|  |  | wavelet-LHH_glszm_LargeAreaHighGrayLevelEmphasis | wavelet-LHH_glszm_SizeZoneNonUniformityNormalized | wavelet-LHH_glszm_ZoneEntropy |
|  |  | wavelet-LHH_ngtdm_Busyness | wavelet-LHH_ngtdm_Strength | wavelet-HLL_firstorder_Kurtosis |
|  |  | wavelet-HLL_firstorder_Maximum | wavelet-HLL_firstorder_Median | wavelet-HLL_glcm_Correlation |
|  |  | wavelet-HLL_glcm_DifferenceVariance | wavelet-HLL_glcm_Id | wavelet-HLL_glcm_Idm |
|  |  | wavelet-HLL_gldm_DependenceVariance | wavelet-HLL_gldm_SmallDependenceLowGrayLevelEmphasis | wavelet-HLL_glszm_LargeAreaHighGrayLevelEmphasis |
|  |  | wavelet-HLL_glszm_LowGrayLevelZoneEmphasis | wavelet-HLL_glszm_SmallAreaEmphasis | wavelet-HLL_glszm_ZoneEntropy |
|  |  | wavelet-HLL_ngtdm_Contrast | wavelet-HLH_glcm_MCC | wavelet-HLH_glcm_SumSquares |
|  |  | wavelet-HLH_glrlm_LongRunLowGrayLevelEmphasis | wavelet-HLH_glszm_SizeZoneNonUniformityNormalized | wavelet-HLH_glszm_SmallAreaLowGrayLevelEmphasis |
|  |  | wavelet-HLH_glszm_ZoneEntropy | wavelet-HHL_glcm_Imc1 | wavelet-HHL_glcm_InverseVariance |
|  |  | wavelet-HHL_glcm_MCC | wavelet-HHL_glcm_MaximumProbability | wavelet-HHL_glrlm_RunEntropy |
|  |  | wavelet-HHL_glrlm_ShortRunLowGrayLevelEmphasis | wavelet-HHL_glszm_GrayLevelNonUniformityNormalized | wavelet-HHL_glszm_LargeAreaHighGrayLevelEmphasis |
|  |  | wavelet-HHL_glszm_SmallAreaEmphasis | wavelet-HHL_glszm_SmallAreaLowGrayLevelEmphasis | wavelet-HHL_glszm_ZoneEntropy |
|  |  | wavelet-HHL_ngtdm_Complexity | wavelet-HHH_firstorder_Median | wavelet-HHH_firstorder_Range |
|  |  | wavelet-HHH_glszm_GrayLevelNonUniformityNormalized | wavelet-HHH_glszm_LowGrayLevelZoneEmphasis | wavelet-HHH_glszm_SizeZoneNonUniformityNormalized |
|  |  | wavelet-HHH_glszm_ZoneEntropy | wavelet-LLL_firstorder_Median | wavelet-LLL_glcm_Correlation |
|  |  | wavelet-LLL_glcm_DifferenceVariance | wavelet-LLL_glcm_Imc1 | wavelet-LLL_glszm_GrayLevelNonUniformityNormalized |
|  |  | wavelet-LLL_glszm_SmallAreaEmphasis | wavelet-LLL_ngtdm_Strength |  |
| Selection_11 | 0.00187 | original_shape_Elongation | original_shape_Flatness | original_shape_Sphericity |
|  |  | original_shape_SurfaceArea | original_firstorder_Kurtosis | original_glcm_Idn |
|  |  | original_glcm_MaximumProbability | original_glszm_GrayLevelNonUniformityNormalized | original_glszm_SizeZoneNonUniformity |
|  |  | original_glszm_SmallAreaEmphasis | original_glszm_SmallAreaLowGrayLevelEmphasis | wavelet-LLH_firstorder_90Percentile |
|  |  | wavelet-LLH_gldm_SmallDependenceLowGrayLevelEmphasis | wavelet-LLH_glrlm_GrayLevelNonUniformityNormalized | wavelet-LLH_glszm_LargeAreaHighGrayLevelEmphasis |
|  |  | wavelet-LLH_glszm_LowGrayLevelZoneEmphasis | wavelet-LLH_glszm_SmallAreaEmphasis | wavelet-LLH_glszm_SmallAreaLowGrayLevelEmphasis |
|  |  | wavelet-LLH_ngtdm_Busyness | wavelet-LLH_ngtdm_Strength | wavelet-LHL_firstorder_Maximum |
|  |  | wavelet-LHL_firstorder_Median | wavelet-LHL_firstorder_Skewness | wavelet-LHL_glcm_InverseVariance |
|  |  | wavelet-LHL_glcm_MCC | wavelet-LHL_glrlm_RunEntropy | wavelet-LHL_glszm_SmallAreaEmphasis |
|  |  | wavelet-LHL_glszm_ZoneEntropy | wavelet-LHL_ngtdm_Busyness | wavelet-LHH_firstorder_Mean |
|  |  | wavelet-LHH_firstorder_Uniformity | wavelet-LHH_glcm_JointEnergy | wavelet-LHH_glcm_SumEntropy |
|  |  | wavelet-LHH_gldm_LargeDependenceLowGrayLevelEmphasis | wavelet-LHH_glszm_GrayLevelVariance | wavelet-LHH_glszm_LargeAreaHighGrayLevelEmphasis |
|  |  | wavelet-LHH_glszm_SizeZoneNonUniformityNormalized | wavelet-LHH_glszm_ZoneEntropy | wavelet-LHH_glszm_ZonePercentage |
|  |  | wavelet-LHH_ngtdm_Busyness | wavelet-LHH_ngtdm_Strength | wavelet-HLL_firstorder_Kurtosis |
|  |  | wavelet-HLL_firstorder_Maximum | wavelet-HLL_firstorder_Median | wavelet-HLL_glcm_Correlation |
|  |  | wavelet-HLL_glcm_DifferenceVariance | wavelet-HLL_glcm_Id | wavelet-HLL_glcm_Idm |
|  |  | wavelet-HLL_glcm_MaximumProbability | wavelet-HLL_gldm_DependenceVariance | wavelet-HLL_gldm_SmallDependenceLowGrayLevelEmphasis |
|  |  | wavelet-HLL_glszm_LargeAreaHighGrayLevelEmphasis | wavelet-HLL_glszm_LowGrayLevelZoneEmphasis | wavelet-HLL_glszm_SmallAreaEmphasis |
|  |  | wavelet-HLL_glszm_ZoneEntropy | wavelet-HLL_ngtdm_Contrast | wavelet-HLH_glcm_Imc1 |
|  |  | wavelet-HLH_glcm_MCC | wavelet-HLH_glcm_SumSquares | wavelet-HLH_glrlm_LongRunLowGrayLevelEmphasis |
|  |  | wavelet-HLH_glszm_LowGrayLevelZoneEmphasis | wavelet-HLH_glszm_SizeZoneNonUniformityNormalized | wavelet-HLH_glszm_SmallAreaLowGrayLevelEmphasis |
|  |  | wavelet-HLH_glszm_ZoneEntropy | wavelet-HHL_glcm_Imc1 | wavelet-HHL_glcm_JointAverage |
|  |  | wavelet-HHL_glcm_MCC | wavelet-HHL_glcm_MaximumProbability | wavelet-HHL_glcm_SumAverage |
|  |  | wavelet-HHL_glrlm_RunEntropy | wavelet-HHL_glszm_GrayLevelNonUniformityNormalized | wavelet-HHL_glszm_LargeAreaHighGrayLevelEmphasis |
|  |  | wavelet-HHL_glszm_SmallAreaEmphasis | wavelet-HHL_glszm_SmallAreaLowGrayLevelEmphasis | wavelet-HHL_ngtdm_Complexity |
|  |  | wavelet-HHH_firstorder_Median | wavelet-HHH_firstorder_Minimum | wavelet-HHH_firstorder_Range |
|  |  | wavelet-HHH_firstorder_Uniformity | wavelet-HHH_glszm_GrayLevelNonUniformityNormalized | wavelet-HHH_glszm_LowGrayLevelZoneEmphasis |
|  |  | wavelet-HHH_glszm_SizeZoneNonUniformityNormalized | wavelet-HHH_glszm_ZoneEntropy | wavelet-LLL_firstorder_Median |
|  |  | wavelet-LLL_glcm_DifferenceVariance | wavelet-LLL_glcm_Imc1 | wavelet-LLL_gldm_DependenceEntropy |
|  |  | wavelet-LLL_gldm_SmallDependenceHighGrayLevelEmphasis | wavelet-LLL_glrlm_RunLengthNonUniformityNormalized | wavelet-LLL_glszm_GrayLevelNonUniformityNormalized |
|  |  | wavelet-LLL_glszm_SmallAreaEmphasis | wavelet-LLL_ngtdm_Strength |  |
| Selection_12 | 0.00135 | original_shape_Elongation | original_shape_Flatness | original_shape_Sphericity |
|  |  | original_shape_SurfaceArea | original_firstorder_Kurtosis | original_glcm_Idn |
|  |  | original_glcm_MaximumProbability | original_glrlm_RunVariance | original_glszm_SizeZoneNonUniformity |
|  |  | original_glszm_SmallAreaEmphasis | original_glszm_SmallAreaLowGrayLevelEmphasis | wavelet-LLH_firstorder_90Percentile |
|  |  | wavelet-LLH_firstorder_Minimum | wavelet-LLH_gldm_SmallDependenceLowGrayLevelEmphasis | wavelet-LLH_glrlm_GrayLevelNonUniformityNormalized |
|  |  | wavelet-LLH_glszm_GrayLevelNonUniformityNormalized | wavelet-LLH_glszm_LargeAreaHighGrayLevelEmphasis | wavelet-LLH_glszm_LowGrayLevelZoneEmphasis |
|  |  | wavelet-LLH_glszm_SmallAreaEmphasis | wavelet-LLH_glszm_SmallAreaLowGrayLevelEmphasis | wavelet-LLH_ngtdm_Busyness |
|  |  | wavelet-LLH_ngtdm_Strength | wavelet-LHL_firstorder_90Percentile | wavelet-LHL_firstorder_Maximum |
|  |  | wavelet-LHL_firstorder_Median | wavelet-LHL_firstorder_Skewness | wavelet-LHL_glcm_InverseVariance |
|  |  | wavelet-LHL_glcm_MCC | wavelet-LHL_gldm_DependenceVariance | wavelet-LHL_glrlm_RunEntropy |
|  |  | wavelet-LHL_glszm_SmallAreaEmphasis | wavelet-LHL_glszm_ZoneEntropy | wavelet-LHL_ngtdm_Busyness |
|  |  | wavelet-LHH_firstorder_Mean | wavelet-LHH_firstorder_Skewness | wavelet-LHH_glcm_JointEnergy |
|  |  | wavelet-LHH_glcm_SumEntropy | wavelet-LHH_gldm_LargeDependenceLowGrayLevelEmphasis | wavelet-LHH_glszm_GrayLevelVariance |
|  |  | wavelet-LHH_glszm_LargeAreaHighGrayLevelEmphasis | wavelet-LHH_glszm_SizeZoneNonUniformityNormalized | wavelet-LHH_glszm_ZoneEntropy |
|  |  | wavelet-LHH_glszm_ZonePercentage | wavelet-LHH_ngtdm_Busyness | wavelet-LHH_ngtdm_Strength |
|  |  | wavelet-HLL_firstorder_Kurtosis | wavelet-HLL_firstorder_Maximum | wavelet-HLL_firstorder_Median |
|  |  | wavelet-HLL_glcm_ClusterShade | wavelet-HLL_glcm_Correlation | wavelet-HLL_glcm_DifferenceVariance |
|  |  | wavelet-HLL_glcm_Idm | wavelet-HLL_glcm_Idmn | wavelet-HLL_glcm_MaximumProbability |
|  |  | wavelet-HLL_gldm_DependenceVariance | wavelet-HLL_gldm_SmallDependenceLowGrayLevelEmphasis | wavelet-HLL_glszm_LargeAreaHighGrayLevelEmphasis |
|  |  | wavelet-HLL_glszm_LowGrayLevelZoneEmphasis | wavelet-HLL_glszm_ZoneEntropy | wavelet-HLH_glcm_Imc1 |
|  |  | wavelet-HLH_glcm_MCC | wavelet-HLH_glcm_SumSquares | wavelet-HLH_glrlm_LongRunLowGrayLevelEmphasis |
|  |  | wavelet-HLH_glszm_LowGrayLevelZoneEmphasis | wavelet-HLH_glszm_SizeZoneNonUniformityNormalized | wavelet-HLH_glszm_SmallAreaLowGrayLevelEmphasis |
|  |  | wavelet-HLH_glszm_ZoneEntropy | wavelet-HHL_glcm_Imc1 | wavelet-HHL_glcm_MCC |
|  |  | wavelet-HHL_glcm_MaximumProbability | wavelet-HHL_glrlm_LongRunHighGrayLevelEmphasis | wavelet-HHL_glrlm_RunEntropy |
|  |  | wavelet-HHL_glszm_GrayLevelNonUniformityNormalized | wavelet-HHL_glszm_LargeAreaHighGrayLevelEmphasis | wavelet-HHL_glszm_SmallAreaEmphasis |
|  |  | wavelet-HHL_glszm_SmallAreaLowGrayLevelEmphasis | wavelet-HHL_ngtdm_Complexity | wavelet-HHH_firstorder_Median |
|  |  | wavelet-HHH_firstorder_Minimum | wavelet-HHH_firstorder_Range | wavelet-HHH_firstorder_Uniformity |
|  |  | wavelet-HHH_glcm_MCC | wavelet-HHH_glszm_GrayLevelNonUniformityNormalized | wavelet-HHH_glszm_LowGrayLevelZoneEmphasis |
|  |  | wavelet-HHH_glszm_SizeZoneNonUniformityNormalized | wavelet-HHH_glszm_ZoneEntropy | wavelet-LLL_firstorder_Median |
|  |  | wavelet-LLL_glcm_DifferenceVariance | wavelet-LLL_glcm_Imc1 | wavelet-LLL_gldm_DependenceEntropy |
|  |  | wavelet-LLL_gldm_SmallDependenceHighGrayLevelEmphasis | wavelet-LLL_glrlm_RunLengthNonUniformityNormalized | wavelet-LLL_glszm_GrayLevelNonUniformityNormalized |
|  |  | wavelet-LLL_glszm_SmallAreaEmphasis | wavelet-LLL_ngtdm_Strength |  |
| Selection_13 | 0.00098 | original_shape_Elongation | original_shape_Flatness | original_shape_Sphericity |
|  |  | original_shape_SurfaceArea | original_firstorder_Kurtosis | original_glcm_ClusterTendency |
|  |  | original_glcm_Correlation | original_glcm_MaximumProbability | original_glrlm_LongRunEmphasis |
|  |  | original_glrlm_RunVariance | original_glszm_SizeZoneNonUniformity | original_glszm_SmallAreaEmphasis |
|  |  | original_glszm_SmallAreaLowGrayLevelEmphasis | wavelet-LLH_firstorder_90Percentile | wavelet-LLH_firstorder_Minimum |
|  |  | wavelet-LLH_gldm_SmallDependenceLowGrayLevelEmphasis | wavelet-LLH_glrlm_GrayLevelNonUniformityNormalized | wavelet-LLH_glszm_GrayLevelNonUniformityNormalized |
|  |  | wavelet-LLH_glszm_LargeAreaHighGrayLevelEmphasis | wavelet-LLH_glszm_LowGrayLevelZoneEmphasis | wavelet-LLH_glszm_SmallAreaEmphasis |
|  |  | wavelet-LLH_glszm_SmallAreaLowGrayLevelEmphasis | wavelet-LLH_ngtdm_Busyness | wavelet-LLH_ngtdm_Strength |
|  |  | wavelet-LHL_firstorder_90Percentile | wavelet-LHL_firstorder_Maximum | wavelet-LHL_firstorder_Skewness |
|  |  | wavelet-LHL_glcm_InverseVariance | wavelet-LHL_glcm_MCC | wavelet-LHL_gldm_DependenceVariance |
|  |  | wavelet-LHL_gldm_LargeDependenceEmphasis | wavelet-LHL_glrlm_RunEntropy | wavelet-LHL_glszm_SmallAreaEmphasis |
|  |  | wavelet-LHL_glszm_ZoneEntropy | wavelet-LHL_ngtdm_Busyness | wavelet-LHH_firstorder_Mean |
|  |  | wavelet-LHH_firstorder_Minimum | wavelet-LHH_firstorder_Skewness | wavelet-LHH_glcm_JointEnergy |
|  |  | wavelet-LHH_glcm_SumEntropy | wavelet-LHH_gldm_LargeDependenceLowGrayLevelEmphasis | wavelet-LHH_glszm_GrayLevelVariance |
|  |  | wavelet-LHH_glszm_LargeAreaHighGrayLevelEmphasis | wavelet-LHH_glszm_SizeZoneNonUniformityNormalized | wavelet-LHH_glszm_ZoneEntropy |
|  |  | wavelet-LHH_glszm_ZonePercentage | wavelet-LHH_ngtdm_Busyness | wavelet-LHH_ngtdm_Strength |
|  |  | wavelet-HLL_firstorder_Kurtosis | wavelet-HLL_firstorder_Maximum | wavelet-HLL_firstorder_Median |
|  |  | wavelet-HLL_glcm_ClusterShade | wavelet-HLL_glcm_Correlation | wavelet-HLL_glcm_DifferenceVariance |
|  |  | wavelet-HLL_glcm_Idmn | wavelet-HLL_glcm_MCC | wavelet-HLL_glcm_MaximumProbability |
|  |  | wavelet-HLL_gldm_DependenceVariance | wavelet-HLL_gldm_SmallDependenceLowGrayLevelEmphasis | wavelet-HLL_glszm_LargeAreaHighGrayLevelEmphasis |
|  |  | wavelet-HLL_glszm_LowGrayLevelZoneEmphasis | wavelet-HLL_glszm_ZoneEntropy | wavelet-HLH_glcm_Imc1 |
|  |  | wavelet-HLH_glcm_MCC | wavelet-HLH_glcm_SumSquares | wavelet-HLH_glrlm_LongRunLowGrayLevelEmphasis |
|  |  | wavelet-HLH_glszm_LowGrayLevelZoneEmphasis | wavelet-HLH_glszm_SizeZoneNonUniformityNormalized | wavelet-HLH_glszm_SmallAreaLowGrayLevelEmphasis |
|  |  | wavelet-HLH_glszm_ZoneEntropy | wavelet-HHL_glcm_Imc1 | wavelet-HHL_glcm_MCC |
|  |  | wavelet-HHL_glcm_MaximumProbability | wavelet-HHL_glrlm_LongRunHighGrayLevelEmphasis | wavelet-HHL_glrlm_RunEntropy |
|  |  | wavelet-HHL_glszm_GrayLevelNonUniformityNormalized | wavelet-HHL_glszm_SizeZoneNonUniformityNormalized | wavelet-HHL_glszm_SmallAreaEmphasis |
|  |  | wavelet-HHL_glszm_SmallAreaLowGrayLevelEmphasis | wavelet-HHL_glszm_ZoneEntropy | wavelet-HHL_ngtdm_Complexity |
|  |  | wavelet-HHH_firstorder_Maximum | wavelet-HHH_firstorder_Median | wavelet-HHH_firstorder_Minimum |
|  |  | wavelet-HHH_firstorder_Range | wavelet-HHH_firstorder_Uniformity | wavelet-HHH_glcm_MCC |
|  |  | wavelet-HHH_glszm_GrayLevelNonUniformityNormalized | wavelet-HHH_glszm_LowGrayLevelZoneEmphasis | wavelet-HHH_glszm_SizeZoneNonUniformityNormalized |
|  |  | wavelet-HHH_glszm_ZoneEntropy | wavelet-HHH_ngtdm_Contrast | wavelet-LLL_firstorder_Median |
|  |  | wavelet-LLL_glcm_DifferenceVariance | wavelet-LLL_glcm_Imc1 | wavelet-LLL_gldm_DependenceEntropy |
|  |  | wavelet-LLL_gldm_SmallDependenceHighGrayLevelEmphasis | wavelet-LLL_glrlm_RunLengthNonUniformityNormalized | wavelet-LLL_glszm_GrayLevelNonUniformityNormalized |
|  |  | wavelet-LLL_glszm_SmallAreaEmphasis | wavelet-LLL_ngtdm_Strength |  |
| Selection_14 | 0.00071 | original_shape_Elongation | original_shape_Flatness | original_shape_Sphericity |
|  |  | original_shape_SurfaceArea | original_firstorder_Kurtosis | original_glcm_ClusterTendency |
|  |  | original_glcm_Correlation | original_glcm_MaximumProbability | original_glrlm_LongRunEmphasis |
|  |  | original_glrlm_RunVariance | original_glszm_SizeZoneNonUniformity | original_glszm_SmallAreaEmphasis |
|  |  | original_glszm_SmallAreaLowGrayLevelEmphasis | wavelet-LLH_firstorder_90Percentile | wavelet-LLH_firstorder_Minimum |
|  |  | wavelet-LLH_gldm_SmallDependenceLowGrayLevelEmphasis | wavelet-LLH_glrlm_GrayLevelNonUniformityNormalized | wavelet-LLH_glszm_GrayLevelNonUniformityNormalized |
|  |  | wavelet-LLH_glszm_LargeAreaHighGrayLevelEmphasis | wavelet-LLH_glszm_LowGrayLevelZoneEmphasis | wavelet-LLH_glszm_SmallAreaEmphasis |
|  |  | wavelet-LLH_glszm_SmallAreaLowGrayLevelEmphasis | wavelet-LLH_ngtdm_Busyness | wavelet-LLH_ngtdm_Strength |
|  |  | wavelet-LHL_firstorder_90Percentile | wavelet-LHL_firstorder_Maximum | wavelet-LHL_firstorder_Skewness |
|  |  | wavelet-LHL_glcm_InverseVariance | wavelet-LHL_glcm_MCC | wavelet-LHL_gldm_DependenceVariance |
|  |  | wavelet-LHL_gldm_LargeDependenceEmphasis | wavelet-LHL_glrlm_RunEntropy | wavelet-LHL_glszm_SmallAreaEmphasis |
|  |  | wavelet-LHL_glszm_ZoneEntropy | wavelet-LHL_ngtdm_Busyness | wavelet-LHH_firstorder_Mean |
|  |  | wavelet-LHH_firstorder_Minimum | wavelet-LHH_firstorder_Skewness | wavelet-LHH_glcm_JointEnergy |
|  |  | wavelet-LHH_glcm_SumEntropy | wavelet-LHH_gldm_LargeDependenceLowGrayLevelEmphasis | wavelet-LHH_glszm_GrayLevelVariance |
|  |  | wavelet-LHH_glszm_LargeAreaHighGrayLevelEmphasis | wavelet-LHH_glszm_SizeZoneNonUniformityNormalized | wavelet-LHH_glszm_ZoneEntropy |
|  |  | wavelet-LHH_glszm_ZonePercentage | wavelet-LHH_ngtdm_Busyness | wavelet-LHH_ngtdm_Strength |
|  |  | wavelet-HLL_firstorder_Kurtosis | wavelet-HLL_firstorder_Maximum | wavelet-HLL_firstorder_Median |
|  |  | wavelet-HLL_glcm_ClusterShade | wavelet-HLL_glcm_Correlation | wavelet-HLL_glcm_DifferenceVariance |
|  |  | wavelet-HLL_glcm_Idmn | wavelet-HLL_glcm_MCC | wavelet-HLL_glcm_MaximumProbability |
|  |  | wavelet-HLL_gldm_SmallDependenceLowGrayLevelEmphasis | wavelet-HLL_glszm_GrayLevelVariance | wavelet-HLL_glszm_LargeAreaHighGrayLevelEmphasis |
|  |  | wavelet-HLL_glszm_LowGrayLevelZoneEmphasis | wavelet-HLL_glszm_ZoneEntropy | wavelet-HLH_glcm_Imc1 |
|  |  | wavelet-HLH_glcm_MCC | wavelet-HLH_glcm_SumSquares | wavelet-HLH_glrlm_LongRunEmphasis |
|  |  | wavelet-HLH_glrlm_LongRunLowGrayLevelEmphasis | wavelet-HLH_glszm_LowGrayLevelZoneEmphasis | wavelet-HLH_glszm_SizeZoneNonUniformityNormalized |
|  |  | wavelet-HLH_glszm_SmallAreaLowGrayLevelEmphasis | wavelet-HLH_glszm_ZoneEntropy | wavelet-HHL_glcm_Imc1 |
|  |  | wavelet-HHL_glcm_MCC | wavelet-HHL_glcm_MaximumProbability | wavelet-HHL_glrlm_LongRunHighGrayLevelEmphasis |
|  |  | wavelet-HHL_glrlm_RunEntropy | wavelet-HHL_glszm_GrayLevelNonUniformityNormalized | wavelet-HHL_glszm_SizeZoneNonUniformityNormalized |
|  |  | wavelet-HHL_glszm_SmallAreaEmphasis | wavelet-HHL_glszm_SmallAreaLowGrayLevelEmphasis | wavelet-HHL_glszm_ZoneEntropy |
|  |  | wavelet-HHL_ngtdm_Complexity | wavelet-HHH_firstorder_Maximum | wavelet-HHH_firstorder_Median |
|  |  | wavelet-HHH_firstorder_Minimum | wavelet-HHH_firstorder_Range | wavelet-HHH_firstorder_Uniformity |
|  |  | wavelet-HHH_glcm_MCC | wavelet-HHH_glszm_GrayLevelNonUniformityNormalized | wavelet-HHH_glszm_LowGrayLevelZoneEmphasis |
|  |  | wavelet-HHH_glszm_SizeZoneNonUniformityNormalized | wavelet-HHH_glszm_ZoneEntropy | wavelet-HHH_ngtdm_Contrast |
|  |  | wavelet-LLL_firstorder_Median | wavelet-LLL_glcm_DifferenceVariance | wavelet-LLL_glcm_Imc1 |
|  |  | wavelet-LLL_gldm_DependenceEntropy | wavelet-LLL_gldm_SmallDependenceHighGrayLevelEmphasis | wavelet-LLL_glrlm_RunLengthNonUniformityNormalized |
|  |  | wavelet-LLL_glszm_GrayLevelNonUniformityNormalized | wavelet-LLL_glszm_SmallAreaEmphasis |  |
| Selection_15 | 0.00051 | original_shape_Elongation | original_shape_Flatness | original_shape_Maximum2DDiameterRow |
|  |  | original_shape_Sphericity | original_shape_SurfaceArea | original_firstorder_Kurtosis |
|  |  | original_firstorder_Skewness | original_glcm_ClusterTendency | original_glcm_Correlation |
|  |  | original_glcm_MaximumProbability | original_glrlm_LongRunEmphasis | original_glrlm_RunVariance |
|  |  | original_glszm_SizeZoneNonUniformity | original_glszm_SmallAreaEmphasis | original_glszm_SmallAreaLowGrayLevelEmphasis |
|  |  | wavelet-LLH_firstorder_90Percentile | wavelet-LLH_firstorder_Minimum | wavelet-LLH_glcm_MCC |
|  |  | wavelet-LLH_gldm_SmallDependenceLowGrayLevelEmphasis | wavelet-LLH_glrlm_GrayLevelNonUniformityNormalized | wavelet-LLH_glszm_GrayLevelNonUniformityNormalized |
|  |  | wavelet-LLH_glszm_LargeAreaHighGrayLevelEmphasis | wavelet-LLH_glszm_LowGrayLevelZoneEmphasis | wavelet-LLH_glszm_SizeZoneNonUniformityNormalized |
|  |  | wavelet-LLH_glszm_SmallAreaEmphasis | wavelet-LLH_glszm_SmallAreaLowGrayLevelEmphasis | wavelet-LLH_ngtdm_Busyness |
|  |  | wavelet-LLH_ngtdm_Contrast | wavelet-LLH_ngtdm_Strength | wavelet-LHL_firstorder_90Percentile |
|  |  | wavelet-LHL_firstorder_Maximum | wavelet-LHL_firstorder_Skewness | wavelet-LHL_glcm_InverseVariance |
|  |  | wavelet-LHL_glcm_MCC | wavelet-LHL_gldm_DependenceVariance | wavelet-LHL_gldm_LargeDependenceEmphasis |
|  |  | wavelet-LHL_glrlm_RunEntropy | wavelet-LHL_glszm_SmallAreaEmphasis | wavelet-LHL_glszm_ZoneEntropy |
|  |  | wavelet-LHH_firstorder_Mean | wavelet-LHH_firstorder_Minimum | wavelet-LHH_firstorder_Skewness |
|  |  | wavelet-LHH_glcm_JointEnergy | wavelet-LHH_glcm_SumEntropy | wavelet-LHH_gldm_LargeDependenceLowGrayLevelEmphasis |
|  |  | wavelet-LHH_glszm_GrayLevelVariance | wavelet-LHH_glszm_LargeAreaHighGrayLevelEmphasis | wavelet-LHH_glszm_SizeZoneNonUniformityNormalized |
|  |  | wavelet-LHH_glszm_ZoneEntropy | wavelet-LHH_glszm_ZonePercentage | wavelet-LHH_ngtdm_Busyness |
|  |  | wavelet-LHH_ngtdm_Strength | wavelet-HLL_firstorder_Kurtosis | wavelet-HLL_firstorder_Maximum |
|  |  | wavelet-HLL_firstorder_Median | wavelet-HLL_glcm_ClusterShade | wavelet-HLL_glcm_Correlation |
|  |  | wavelet-HLL_glcm_DifferenceVariance | wavelet-HLL_glcm_Idmn | wavelet-HLL_glcm_MCC |
|  |  | wavelet-HLL_glcm_MaximumProbability | wavelet-HLL_gldm_SmallDependenceLowGrayLevelEmphasis | wavelet-HLL_glszm_GrayLevelVariance |
|  |  | wavelet-HLL_glszm_LargeAreaHighGrayLevelEmphasis | wavelet-HLL_glszm_LowGrayLevelZoneEmphasis | wavelet-HLL_glszm_ZoneEntropy |
|  |  | wavelet-HLH_glcm_Imc1 | wavelet-HLH_glcm_MCC | wavelet-HLH_glcm_SumSquares |
|  |  | wavelet-HLH_glrlm_LongRunEmphasis | wavelet-HLH_glrlm_LongRunLowGrayLevelEmphasis | wavelet-HLH_glszm_LowGrayLevelZoneEmphasis |
|  |  | wavelet-HLH_glszm_SizeZoneNonUniformityNormalized | wavelet-HLH_glszm_SmallAreaLowGrayLevelEmphasis | wavelet-HLH_glszm_ZoneEntropy |
|  |  | wavelet-HHL_firstorder_Skewness | wavelet-HHL_glcm_Idmn | wavelet-HHL_glcm_Imc1 |
|  |  | wavelet-HHL_glcm_MCC | wavelet-HHL_glcm_MaximumProbability | wavelet-HHL_glrlm_LongRunHighGrayLevelEmphasis |
|  |  | wavelet-HHL_glszm_GrayLevelNonUniformityNormalized | wavelet-HHL_glszm_SizeZoneNonUniformityNormalized | wavelet-HHL_glszm_SmallAreaEmphasis |
|  |  | wavelet-HHL_glszm_SmallAreaLowGrayLevelEmphasis | wavelet-HHL_glszm_ZoneEntropy | wavelet-HHH_firstorder_Maximum |
|  |  | wavelet-HHH_firstorder_Median | wavelet-HHH_firstorder_Minimum | wavelet-HHH_firstorder_Range |
|  |  | wavelet-HHH_firstorder_Uniformity | wavelet-HHH_glcm_MCC | wavelet-HHH_glszm_GrayLevelNonUniformityNormalized |
|  |  | wavelet-HHH_glszm_LowGrayLevelZoneEmphasis | wavelet-HHH_glszm_SizeZoneNonUniformityNormalized | wavelet-HHH_glszm_SmallAreaEmphasis |
|  |  | wavelet-HHH_glszm_ZoneEntropy | wavelet-HHH_ngtdm_Contrast | wavelet-LLL_firstorder_Median |
|  |  | wavelet-LLL_firstorder_Skewness | wavelet-LLL_glcm_DifferenceVariance | wavelet-LLL_glcm_Imc1 |
|  |  | wavelet-LLL_gldm_DependenceEntropy | wavelet-LLL_gldm_SmallDependenceHighGrayLevelEmphasis | wavelet-LLL_glrlm_RunLengthNonUniformityNormalized |
|  |  | wavelet-LLL_glszm_GrayLevelNonUniformityNormalized | wavelet-LLL_glszm_SmallAreaEmphasis |  |
| Selection_16 | 0.00037 | original_shape_Elongation | original_shape_Flatness | original_shape_Maximum2DDiameterRow |
|  |  | original_shape_Sphericity | original_shape_SurfaceArea | original_firstorder_Kurtosis |
|  |  | original_firstorder_Median | original_firstorder_Skewness | original_glcm_ClusterTendency |
|  |  | original_glcm_Correlation | original_glcm_DifferenceVariance | original_glcm_MaximumProbability |
|  |  | original_glrlm_LongRunEmphasis | original_glrlm_RunVariance | original_glszm_SizeZoneNonUniformity |
|  |  | original_glszm_SmallAreaEmphasis | original_glszm_SmallAreaLowGrayLevelEmphasis | wavelet-LLH_firstorder_90Percentile |
|  |  | wavelet-LLH_firstorder_Minimum | wavelet-LLH_glcm_Idn | wavelet-LLH_glcm_MCC |
|  |  | wavelet-LLH_gldm_SmallDependenceLowGrayLevelEmphasis | wavelet-LLH_glrlm_GrayLevelNonUniformityNormalized | wavelet-LLH_glszm_GrayLevelNonUniformityNormalized |
|  |  | wavelet-LLH_glszm_LargeAreaHighGrayLevelEmphasis | wavelet-LLH_glszm_LowGrayLevelZoneEmphasis | wavelet-LLH_glszm_SizeZoneNonUniformityNormalized |
|  |  | wavelet-LLH_glszm_SmallAreaEmphasis | wavelet-LLH_glszm_SmallAreaLowGrayLevelEmphasis | wavelet-LLH_ngtdm_Busyness |
|  |  | wavelet-LLH_ngtdm_Contrast | wavelet-LLH_ngtdm_Strength | wavelet-LHL_firstorder_90Percentile |
|  |  | wavelet-LHL_firstorder_Maximum | wavelet-LHL_firstorder_Skewness | wavelet-LHL_glcm_InverseVariance |
|  |  | wavelet-LHL_glcm_MCC | wavelet-LHL_gldm_DependenceVariance | wavelet-LHL_gldm_LargeDependenceEmphasis |
|  |  | wavelet-LHL_glrlm_RunEntropy | wavelet-LHL_glszm_SmallAreaEmphasis | wavelet-LHL_glszm_ZoneEntropy |
|  |  | wavelet-LHH_firstorder_Mean | wavelet-LHH_firstorder_Minimum | wavelet-LHH_firstorder_Skewness |
|  |  | wavelet-LHH_glcm_JointEnergy | wavelet-LHH_glcm_SumEntropy | wavelet-LHH_gldm_LargeDependenceLowGrayLevelEmphasis |
|  |  | wavelet-LHH_glszm_GrayLevelVariance | wavelet-LHH_glszm_LargeAreaHighGrayLevelEmphasis | wavelet-LHH_glszm_SizeZoneNonUniformityNormalized |
|  |  | wavelet-LHH_glszm_ZoneEntropy | wavelet-LHH_glszm_ZonePercentage | wavelet-LHH_ngtdm_Busyness |
|  |  | wavelet-LHH_ngtdm_Strength | wavelet-HLL_firstorder_Kurtosis | wavelet-HLL_firstorder_Maximum |
|  |  | wavelet-HLL_firstorder_Median | wavelet-HLL_glcm_ClusterShade | wavelet-HLL_glcm_Correlation |
|  |  | wavelet-HLL_glcm_DifferenceVariance | wavelet-HLL_glcm_Idmn | wavelet-HLL_glcm_MCC |
|  |  | wavelet-HLL_glcm_MaximumProbability | wavelet-HLL_gldm_SmallDependenceLowGrayLevelEmphasis | wavelet-HLL_glszm_GrayLevelVariance |
|  |  | wavelet-HLL_glszm_LargeAreaHighGrayLevelEmphasis | wavelet-HLL_glszm_LowGrayLevelZoneEmphasis | wavelet-HLL_glszm_ZoneEntropy |
|  |  | wavelet-HLH_firstorder_Mean | wavelet-HLH_glcm_Imc1 | wavelet-HLH_glcm_MCC |
|  |  | wavelet-HLH_glcm_SumSquares | wavelet-HLH_glrlm_LongRunEmphasis | wavelet-HLH_glrlm_LongRunLowGrayLevelEmphasis |
|  |  | wavelet-HLH_glszm_LowGrayLevelZoneEmphasis | wavelet-HLH_glszm_SizeZoneNonUniformityNormalized | wavelet-HLH_glszm_SmallAreaLowGrayLevelEmphasis |
|  |  | wavelet-HLH_glszm_ZoneEntropy | wavelet-HHL_firstorder_Skewness | wavelet-HHL_glcm_Idmn |
|  |  | wavelet-HHL_glcm_Imc1 | wavelet-HHL_glcm_MCC | wavelet-HHL_glcm_MaximumProbability |
|  |  | wavelet-HHL_glrlm_LongRunHighGrayLevelEmphasis | wavelet-HHL_glszm_GrayLevelNonUniformityNormalized | wavelet-HHL_glszm_SizeZoneNonUniformityNormalized |
|  |  | wavelet-HHL_glszm_SmallAreaEmphasis | wavelet-HHL_glszm_SmallAreaLowGrayLevelEmphasis | wavelet-HHL_glszm_ZoneEntropy |
|  |  | wavelet-HHH_firstorder_Maximum | wavelet-HHH_firstorder_Median | wavelet-HHH_firstorder_Minimum |
|  |  | wavelet-HHH_firstorder_Range | wavelet-HHH_firstorder_Uniformity | wavelet-HHH_glcm_MCC |
|  |  | wavelet-HHH_glszm_GrayLevelNonUniformityNormalized | wavelet-HHH_glszm_LowGrayLevelZoneEmphasis | wavelet-HHH_glszm_SizeZoneNonUniformityNormalized |
|  |  | wavelet-HHH_glszm_ZoneEntropy | wavelet-HHH_ngtdm_Contrast | wavelet-LLL_firstorder_Median |
|  |  | wavelet-LLL_firstorder_Skewness | wavelet-LLL_glcm_Correlation | wavelet-LLL_glcm_DifferenceVariance |
|  |  | wavelet-LLL_glcm_Imc1 | wavelet-LLL_gldm_SmallDependenceHighGrayLevelEmphasis | wavelet-LLL_glrlm_RunLengthNonUniformityNormalized |
|  |  | wavelet-LLL_glszm_GrayLevelNonUniformityNormalized | wavelet-LLL_glszm_SmallAreaEmphasis |  |
| Selection_17 | 0.00027 | original_shape_Elongation | original_shape_Flatness | original_shape_Maximum2DDiameterRow |
|  |  | original_shape_Sphericity | original_shape_SurfaceArea | original_firstorder_Kurtosis |
|  |  | original_firstorder_Median | original_firstorder_Skewness | original_glcm_ClusterTendency |
|  |  | original_glcm_DifferenceVariance | original_glcm_MaximumProbability | original_glrlm_LongRunEmphasis |
|  |  | original_glszm_SizeZoneNonUniformity | original_glszm_SmallAreaEmphasis | original_glszm_SmallAreaLowGrayLevelEmphasis |
|  |  | wavelet-LLH_firstorder_90Percentile | wavelet-LLH_firstorder_Median | wavelet-LLH_firstorder_Minimum |
|  |  | wavelet-LLH_glcm_Idn | wavelet-LLH_glcm_MCC | wavelet-LLH_gldm_SmallDependenceLowGrayLevelEmphasis |
|  |  | wavelet-LLH_glrlm_GrayLevelNonUniformityNormalized | wavelet-LLH_glszm_GrayLevelNonUniformityNormalized | wavelet-LLH_glszm_LargeAreaHighGrayLevelEmphasis |
|  |  | wavelet-LLH_glszm_LowGrayLevelZoneEmphasis | wavelet-LLH_glszm_SizeZoneNonUniformityNormalized | wavelet-LLH_glszm_SmallAreaEmphasis |
|  |  | wavelet-LLH_glszm_SmallAreaLowGrayLevelEmphasis | wavelet-LLH_ngtdm_Busyness | wavelet-LLH_ngtdm_Contrast |
|  |  | wavelet-LLH_ngtdm_Strength | wavelet-LHL_firstorder_90Percentile | wavelet-LHL_firstorder_Maximum |
|  |  | wavelet-LHL_firstorder_Skewness | wavelet-LHL_glcm_InverseVariance | wavelet-LHL_glcm_MCC |
|  |  | wavelet-LHL_gldm_DependenceVariance | wavelet-LHL_gldm_LargeDependenceEmphasis | wavelet-LHL_glrlm_RunEntropy |
|  |  | wavelet-LHL_glszm_SmallAreaEmphasis | wavelet-LHL_glszm_ZoneEntropy | wavelet-LHH_firstorder_Mean |
|  |  | wavelet-LHH_firstorder_Minimum | wavelet-LHH_firstorder_Skewness | wavelet-LHH_glcm_SumEntropy |
|  |  | wavelet-LHH_gldm_LargeDependenceLowGrayLevelEmphasis | wavelet-LHH_glszm_GrayLevelVariance | wavelet-LHH_glszm_LargeAreaHighGrayLevelEmphasis |
|  |  | wavelet-LHH_glszm_SizeZoneNonUniformityNormalized | wavelet-LHH_glszm_ZonePercentage | wavelet-LHH_ngtdm_Busyness |
|  |  | wavelet-LHH_ngtdm_Strength | wavelet-HLL_firstorder_Kurtosis | wavelet-HLL_firstorder_Maximum |
|  |  | wavelet-HLL_firstorder_Median | wavelet-HLL_firstorder_Skewness | wavelet-HLL_glcm_ClusterShade |
|  |  | wavelet-HLL_glcm_Correlation | wavelet-HLL_glcm_DifferenceVariance | wavelet-HLL_glcm_Idmn |
|  |  | wavelet-HLL_glcm_MCC | wavelet-HLL_glcm_MaximumProbability | wavelet-HLL_gldm_SmallDependenceLowGrayLevelEmphasis |
|  |  | wavelet-HLL_glszm_GrayLevelVariance | wavelet-HLL_glszm_LargeAreaHighGrayLevelEmphasis | wavelet-HLL_glszm_LowGrayLevelZoneEmphasis |
|  |  | wavelet-HLL_glszm_ZoneEntropy | wavelet-HLH_firstorder_Mean | wavelet-HLH_glcm_Imc1 |
|  |  | wavelet-HLH_glcm_MCC | wavelet-HLH_glcm_SumSquares | wavelet-HLH_glrlm_LongRunEmphasis |
|  |  | wavelet-HLH_glrlm_LongRunLowGrayLevelEmphasis | wavelet-HLH_glszm_LowGrayLevelZoneEmphasis | wavelet-HLH_glszm_SizeZoneNonUniformityNormalized |
|  |  | wavelet-HLH_glszm_SmallAreaLowGrayLevelEmphasis | wavelet-HLH_glszm_ZoneEntropy | wavelet-HHL_firstorder_Skewness |
|  |  | wavelet-HHL_glcm_Idmn | wavelet-HHL_glcm_Imc1 | wavelet-HHL_glcm_MCC |
|  |  | wavelet-HHL_glcm_MaximumProbability | wavelet-HHL_glrlm_LongRunHighGrayLevelEmphasis | wavelet-HHL_glszm_GrayLevelNonUniformityNormalized |
|  |  | wavelet-HHL_glszm_SizeZoneNonUniformityNormalized | wavelet-HHL_glszm_SmallAreaEmphasis | wavelet-HHL_glszm_SmallAreaLowGrayLevelEmphasis |
|  |  | wavelet-HHL_glszm_ZoneEntropy | wavelet-HHH_firstorder_Maximum | wavelet-HHH_firstorder_Median |
|  |  | wavelet-HHH_firstorder_Minimum | wavelet-HHH_firstorder_Range | wavelet-HHH_firstorder_Uniformity |
|  |  | wavelet-HHH_glcm_MCC | wavelet-HHH_glszm_GrayLevelNonUniformityNormalized | wavelet-HHH_glszm_LowGrayLevelZoneEmphasis |
|  |  | wavelet-HHH_glszm_SizeZoneNonUniformityNormalized | wavelet-HHH_glszm_SmallAreaEmphasis | wavelet-HHH_glszm_ZoneEntropy |
|  |  | wavelet-HHH_ngtdm_Contrast | wavelet-LLL_firstorder_Median | wavelet-LLL_firstorder_Skewness |
|  |  | wavelet-LLL_glcm_Correlation | wavelet-LLL_glcm_DifferenceVariance | wavelet-LLL_glcm_Imc1 |
|  |  | wavelet-LLL_gldm_SmallDependenceHighGrayLevelEmphasis | wavelet-LLL_glrlm_RunLengthNonUniformityNormalized | wavelet-LLL_glszm_GrayLevelNonUniformityNormalized |
|  |  | wavelet-LLL_glszm_SmallAreaEmphasis |  |  |
| Selection_18 | 0.00019 | original_shape_Elongation | original_shape_Flatness | original_shape_Maximum2DDiameterRow |
|  |  | original_shape_Sphericity | original_shape_SurfaceArea | original_firstorder_Kurtosis |
|  |  | original_firstorder_Median | original_firstorder_Skewness | original_glcm_ClusterTendency |
|  |  | original_glcm_DifferenceVariance | original_glcm_MaximumProbability | original_glrlm_LongRunEmphasis |
|  |  | original_glszm_SizeZoneNonUniformity | original_glszm_SmallAreaEmphasis | original_glszm_SmallAreaLowGrayLevelEmphasis |
|  |  | wavelet-LLH_firstorder_90Percentile | wavelet-LLH_firstorder_Median | wavelet-LLH_firstorder_Minimum |
|  |  | wavelet-LLH_glcm_Idn | wavelet-LLH_glcm_MCC | wavelet-LLH_gldm_SmallDependenceLowGrayLevelEmphasis |
|  |  | wavelet-LLH_glrlm_GrayLevelNonUniformityNormalized | wavelet-LLH_glszm_GrayLevelNonUniformityNormalized | wavelet-LLH_glszm_LargeAreaHighGrayLevelEmphasis |
|  |  | wavelet-LLH_glszm_LowGrayLevelZoneEmphasis | wavelet-LLH_glszm_SizeZoneNonUniformityNormalized | wavelet-LLH_glszm_SmallAreaEmphasis |
|  |  | wavelet-LLH_glszm_SmallAreaLowGrayLevelEmphasis | wavelet-LLH_ngtdm_Busyness | wavelet-LLH_ngtdm_Contrast |
|  |  | wavelet-LLH_ngtdm_Strength | wavelet-LHL_firstorder_90Percentile | wavelet-LHL_firstorder_Maximum |
|  |  | wavelet-LHL_firstorder_Skewness | wavelet-LHL_glcm_Autocorrelation | wavelet-LHL_glcm_InverseVariance |
|  |  | wavelet-LHL_glcm_MCC | wavelet-LHL_gldm_DependenceVariance | wavelet-LHL_gldm_HighGrayLevelEmphasis |
|  |  | wavelet-LHL_gldm_LargeDependenceEmphasis | wavelet-LHL_glrlm_RunEntropy | wavelet-LHL_glszm_SmallAreaEmphasis |
|  |  | wavelet-LHL_glszm_ZoneEntropy | wavelet-LHH_firstorder_Mean | wavelet-LHH_firstorder_Minimum |
|  |  | wavelet-LHH_firstorder_Skewness | wavelet-LHH_glcm_SumEntropy | wavelet-LHH_gldm_LargeDependenceLowGrayLevelEmphasis |
|  |  | wavelet-LHH_glszm_GrayLevelVariance | wavelet-LHH_glszm_LargeAreaHighGrayLevelEmphasis | wavelet-LHH_glszm_SizeZoneNonUniformityNormalized |
|  |  | wavelet-LHH_glszm_ZonePercentage | wavelet-LHH_ngtdm_Busyness | wavelet-LHH_ngtdm_Strength |
|  |  | wavelet-HLL_firstorder_Kurtosis | wavelet-HLL_firstorder_Maximum | wavelet-HLL_firstorder_Median |
|  |  | wavelet-HLL_firstorder_Skewness | wavelet-HLL_glcm_ClusterShade | wavelet-HLL_glcm_Correlation |
|  |  | wavelet-HLL_glcm_DifferenceVariance | wavelet-HLL_glcm_Idmn | wavelet-HLL_glcm_MCC |
|  |  | wavelet-HLL_glcm_MaximumProbability | wavelet-HLL_gldm_SmallDependenceLowGrayLevelEmphasis | wavelet-HLL_glszm_GrayLevelVariance |
|  |  | wavelet-HLL_glszm_LargeAreaHighGrayLevelEmphasis | wavelet-HLL_glszm_LowGrayLevelZoneEmphasis | wavelet-HLL_glszm_SmallAreaEmphasis |
|  |  | wavelet-HLL_glszm_ZoneEntropy | wavelet-HLH_firstorder_Mean | wavelet-HLH_glcm_Imc1 |
|  |  | wavelet-HLH_glcm_MCC | wavelet-HLH_glcm_SumSquares | wavelet-HLH_glrlm_LongRunEmphasis |
|  |  | wavelet-HLH_glrlm_LongRunLowGrayLevelEmphasis | wavelet-HLH_glszm_LowGrayLevelZoneEmphasis | wavelet-HLH_glszm_SizeZoneNonUniformityNormalized |
|  |  | wavelet-HLH_glszm_SmallAreaLowGrayLevelEmphasis | wavelet-HLH_glszm_ZoneEntropy | wavelet-HHL_firstorder_Skewness |
|  |  | wavelet-HHL_glcm_Idmn | wavelet-HHL_glcm_Imc1 | wavelet-HHL_glcm_MCC |
|  |  | wavelet-HHL_glcm_MaximumProbability | wavelet-HHL_glrlm_LongRunHighGrayLevelEmphasis | wavelet-HHL_glrlm_RunEntropy |
|  |  | wavelet-HHL_glszm_GrayLevelNonUniformityNormalized | wavelet-HHL_glszm_SizeZoneNonUniformityNormalized | wavelet-HHL_glszm_SmallAreaEmphasis |
|  |  | wavelet-HHL_glszm_SmallAreaLowGrayLevelEmphasis | wavelet-HHL_glszm_ZoneEntropy | wavelet-HHH_firstorder_Maximum |
|  |  | wavelet-HHH_firstorder_Median | wavelet-HHH_firstorder_Minimum | wavelet-HHH_firstorder_Range |
|  |  | wavelet-HHH_firstorder_Uniformity | wavelet-HHH_glcm_MCC | wavelet-HHH_glszm_GrayLevelNonUniformityNormalized |
|  |  | wavelet-HHH_glszm_LowGrayLevelZoneEmphasis | wavelet-HHH_glszm_SizeZoneNonUniformityNormalized | wavelet-HHH_glszm_SmallAreaEmphasis |
|  |  | wavelet-HHH_glszm_ZoneEntropy | wavelet-HHH_ngtdm_Contrast | wavelet-LLL_firstorder_Median |
|  |  | wavelet-LLL_firstorder_Skewness | wavelet-LLL_glcm_Correlation | wavelet-LLL_glcm_DifferenceVariance |
|  |  | wavelet-LLL_glcm_Imc1 | wavelet-LLL_gldm_SmallDependenceHighGrayLevelEmphasis | wavelet-LLL_glrlm_RunLengthNonUniformityNormalized |
|  |  | wavelet-LLL_glszm_GrayLevelNonUniformityNormalized | wavelet-LLL_glszm_SmallAreaEmphasis |  |
| Selection_19 | 0.00014 | original_shape_Elongation | original_shape_Flatness | original_shape_Maximum2DDiameterRow |
|  |  | original_shape_Sphericity | original_shape_SurfaceArea | original_firstorder_Kurtosis |
|  |  | original_firstorder_Median | original_firstorder_Skewness | original_glcm_ClusterTendency |
|  |  | original_glcm_DifferenceVariance | original_glcm_MaximumProbability | original_glrlm_LongRunEmphasis |
|  |  | original_glszm_SizeZoneNonUniformity | original_glszm_SmallAreaEmphasis | original_glszm_SmallAreaLowGrayLevelEmphasis |
|  |  | wavelet-LLH_firstorder_90Percentile | wavelet-LLH_firstorder_Median | wavelet-LLH_firstorder_Minimum |
|  |  | wavelet-LLH_glcm_Idn | wavelet-LLH_glcm_MCC | wavelet-LLH_gldm_SmallDependenceLowGrayLevelEmphasis |
|  |  | wavelet-LLH_glrlm_GrayLevelNonUniformityNormalized | wavelet-LLH_glszm_GrayLevelNonUniformityNormalized | wavelet-LLH_glszm_LargeAreaHighGrayLevelEmphasis |
|  |  | wavelet-LLH_glszm_LowGrayLevelZoneEmphasis | wavelet-LLH_glszm_SizeZoneNonUniformityNormalized | wavelet-LLH_glszm_SmallAreaEmphasis |
|  |  | wavelet-LLH_glszm_SmallAreaLowGrayLevelEmphasis | wavelet-LLH_ngtdm_Busyness | wavelet-LLH_ngtdm_Contrast |
|  |  | wavelet-LLH_ngtdm_Strength | wavelet-LHL_firstorder_90Percentile | wavelet-LHL_firstorder_Maximum |
|  |  | wavelet-LHL_firstorder_Skewness | wavelet-LHL_glcm_Autocorrelation | wavelet-LHL_glcm_InverseVariance |
|  |  | wavelet-LHL_glcm_MCC | wavelet-LHL_gldm_DependenceVariance | wavelet-LHL_gldm_HighGrayLevelEmphasis |
|  |  | wavelet-LHL_gldm_LargeDependenceEmphasis | wavelet-LHL_glrlm_RunEntropy | wavelet-LHL_glszm_SmallAreaEmphasis |
|  |  | wavelet-LHL_glszm_ZoneEntropy | wavelet-LHH_firstorder_Mean | wavelet-LHH_firstorder_Minimum |
|  |  | wavelet-LHH_firstorder_Skewness | wavelet-LHH_glcm_SumEntropy | wavelet-LHH_gldm_LargeDependenceLowGrayLevelEmphasis |
|  |  | wavelet-LHH_glszm_GrayLevelVariance | wavelet-LHH_glszm_LargeAreaHighGrayLevelEmphasis | wavelet-LHH_glszm_SizeZoneNonUniformityNormalized |
|  |  | wavelet-LHH_glszm_SmallAreaLowGrayLevelEmphasis | wavelet-LHH_glszm_ZoneEntropy | wavelet-LHH_glszm_ZonePercentage |
|  |  | wavelet-LHH_ngtdm_Busyness | wavelet-LHH_ngtdm_Strength | wavelet-HLL_firstorder_Kurtosis |
|  |  | wavelet-HLL_firstorder_Maximum | wavelet-HLL_firstorder_Median | wavelet-HLL_firstorder_Skewness |
|  |  | wavelet-HLL_glcm_ClusterShade | wavelet-HLL_glcm_Correlation | wavelet-HLL_glcm_DifferenceVariance |
|  |  | wavelet-HLL_glcm_Idmn | wavelet-HLL_glcm_MCC | wavelet-HLL_glcm_MaximumProbability |
|  |  | wavelet-HLL_gldm_SmallDependenceLowGrayLevelEmphasis | wavelet-HLL_glszm_GrayLevelNonUniformityNormalized | wavelet-HLL_glszm_GrayLevelVariance |
|  |  | wavelet-HLL_glszm_LargeAreaHighGrayLevelEmphasis | wavelet-HLL_glszm_LowGrayLevelZoneEmphasis | wavelet-HLL_glszm_SmallAreaEmphasis |
|  |  | wavelet-HLL_glszm_ZoneEntropy | wavelet-HLL_glszm_ZoneVariance | wavelet-HLH_firstorder_Mean |
|  |  | wavelet-HLH_glcm_Imc1 | wavelet-HLH_glcm_MCC | wavelet-HLH_glcm_SumSquares |
|  |  | wavelet-HLH_glrlm_LongRunEmphasis | wavelet-HLH_glrlm_LongRunLowGrayLevelEmphasis | wavelet-HLH_glszm_LowGrayLevelZoneEmphasis |
|  |  | wavelet-HLH_glszm_SizeZoneNonUniformityNormalized | wavelet-HLH_glszm_SmallAreaLowGrayLevelEmphasis | wavelet-HLH_glszm_ZoneEntropy |
|  |  | wavelet-HHL_firstorder_Skewness | wavelet-HHL_glcm_Idmn | wavelet-HHL_glcm_Imc1 |
|  |  | wavelet-HHL_glcm_MCC | wavelet-HHL_glcm_MaximumProbability | wavelet-HHL_glrlm_LongRunHighGrayLevelEmphasis |
|  |  | wavelet-HHL_glrlm_RunEntropy | wavelet-HHL_glszm_GrayLevelNonUniformityNormalized | wavelet-HHL_glszm_SizeZoneNonUniformityNormalized |
|  |  | wavelet-HHL_glszm_SmallAreaEmphasis | wavelet-HHL_glszm_SmallAreaLowGrayLevelEmphasis | wavelet-HHL_glszm_ZoneEntropy |
|  |  | wavelet-HHH_firstorder_Maximum | wavelet-HHH_firstorder_Median | wavelet-HHH_firstorder_Minimum |
|  |  | wavelet-HHH_firstorder_Range | wavelet-HHH_firstorder_Uniformity | wavelet-HHH_glcm_MCC |
|  |  | wavelet-HHH_glszm_GrayLevelNonUniformity | wavelet-HHH_glszm_GrayLevelNonUniformityNormalized | wavelet-HHH_glszm_LowGrayLevelZoneEmphasis |
|  |  | wavelet-HHH_glszm_SizeZoneNonUniformityNormalized | wavelet-HHH_glszm_SmallAreaEmphasis | wavelet-HHH_glszm_ZoneEntropy |
|  |  | wavelet-HHH_ngtdm_Contrast | wavelet-LLL_firstorder_Median | wavelet-LLL_firstorder_Skewness |
|  |  | wavelet-LLL_glcm_Correlation | wavelet-LLL_glcm_DifferenceVariance | wavelet-LLL_glcm_Id |
|  |  | wavelet-LLL_glcm_Idm | wavelet-LLL_glcm_Imc1 | wavelet-LLL_gldm_SmallDependenceHighGrayLevelEmphasis |
|  |  | wavelet-LLL_glrlm_RunLengthNonUniformityNormalized | wavelet-LLL_glszm_GrayLevelNonUniformityNormalized | wavelet-LLL_glszm_SmallAreaEmphasis |
| Selection_20 | 0.00010 | original_shape_Elongation | original_shape_Flatness | original_shape_Maximum2DDiameterRow |
|  |  | original_shape_Sphericity | original_shape_SurfaceArea | original_firstorder_Kurtosis |
|  |  | original_firstorder_Median | original_firstorder_Skewness | original_glcm_ClusterTendency |
|  |  | original_glcm_DifferenceVariance | original_glcm_MaximumProbability | original_glrlm_LongRunEmphasis |
|  |  | original_glszm_SizeZoneNonUniformity | original_glszm_SmallAreaEmphasis | original_glszm_SmallAreaLowGrayLevelEmphasis |
|  |  | wavelet-LLH_firstorder_90Percentile | wavelet-LLH_firstorder_Median | wavelet-LLH_firstorder_Minimum |
|  |  | wavelet-LLH_glcm_Idn | wavelet-LLH_glcm_MCC | wavelet-LLH_gldm_SmallDependenceLowGrayLevelEmphasis |
|  |  | wavelet-LLH_glrlm_GrayLevelNonUniformityNormalized | wavelet-LLH_glszm_GrayLevelNonUniformityNormalized | wavelet-LLH_glszm_LargeAreaHighGrayLevelEmphasis |
|  |  | wavelet-LLH_glszm_LowGrayLevelZoneEmphasis | wavelet-LLH_glszm_SizeZoneNonUniformityNormalized | wavelet-LLH_glszm_SmallAreaEmphasis |
|  |  | wavelet-LLH_glszm_SmallAreaLowGrayLevelEmphasis | wavelet-LLH_ngtdm_Busyness | wavelet-LLH_ngtdm_Contrast |
|  |  | wavelet-LLH_ngtdm_Strength | wavelet-LHL_firstorder_90Percentile | wavelet-LHL_firstorder_Maximum |
|  |  | wavelet-LHL_firstorder_Skewness | wavelet-LHL_glcm_Autocorrelation | wavelet-LHL_glcm_InverseVariance |
|  |  | wavelet-LHL_glcm_MCC | wavelet-LHL_gldm_DependenceVariance | wavelet-LHL_gldm_HighGrayLevelEmphasis |
|  |  | wavelet-LHL_gldm_LargeDependenceEmphasis | wavelet-LHL_glrlm_RunEntropy | wavelet-LHL_glszm_SmallAreaEmphasis |
|  |  | wavelet-LHL_glszm_ZoneEntropy | wavelet-LHH_firstorder_Mean | wavelet-LHH_firstorder_Minimum |
|  |  | wavelet-LHH_firstorder_Skewness | wavelet-LHH_glcm_SumEntropy | wavelet-LHH_gldm_LargeDependenceLowGrayLevelEmphasis |
|  |  | wavelet-LHH_glszm_GrayLevelVariance | wavelet-LHH_glszm_LargeAreaHighGrayLevelEmphasis | wavelet-LHH_glszm_LargeAreaLowGrayLevelEmphasis |
|  |  | wavelet-LHH_glszm_SizeZoneNonUniformityNormalized | wavelet-LHH_glszm_SmallAreaLowGrayLevelEmphasis | wavelet-LHH_glszm_ZoneEntropy |
|  |  | wavelet-LHH_glszm_ZonePercentage | wavelet-LHH_ngtdm_Busyness | wavelet-LHH_ngtdm_Strength |
|  |  | wavelet-HLL_firstorder_Kurtosis | wavelet-HLL_firstorder_Maximum | wavelet-HLL_firstorder_Median |
|  |  | wavelet-HLL_firstorder_Skewness | wavelet-HLL_glcm_ClusterShade | wavelet-HLL_glcm_Correlation |
|  |  | wavelet-HLL_glcm_DifferenceVariance | wavelet-HLL_glcm_Idmn | wavelet-HLL_glcm_MCC |
|  |  | wavelet-HLL_glcm_MaximumProbability | wavelet-HLL_glszm_GrayLevelNonUniformityNormalized | wavelet-HLL_glszm_GrayLevelVariance |
|  |  | wavelet-HLL_glszm_LargeAreaHighGrayLevelEmphasis | wavelet-HLL_glszm_LowGrayLevelZoneEmphasis | wavelet-HLL_glszm_SmallAreaEmphasis |
|  |  | wavelet-HLL_glszm_ZoneEntropy | wavelet-HLL_glszm_ZoneVariance | wavelet-HLH_firstorder_Mean |
|  |  | wavelet-HLH_glcm_Imc1 | wavelet-HLH_glcm_MCC | wavelet-HLH_glcm_SumSquares |
|  |  | wavelet-HLH_gldm_LargeDependenceLowGrayLevelEmphasis | wavelet-HLH_glrlm_LongRunEmphasis | wavelet-HLH_glrlm_LongRunLowGrayLevelEmphasis |
|  |  | wavelet-HLH_glszm_LowGrayLevelZoneEmphasis | wavelet-HLH_glszm_SizeZoneNonUniformityNormalized | wavelet-HLH_glszm_SmallAreaLowGrayLevelEmphasis |
|  |  | wavelet-HLH_glszm_ZoneEntropy | wavelet-HHL_firstorder_Skewness | wavelet-HHL_glcm_Idmn |
|  |  | wavelet-HHL_glcm_Imc1 | wavelet-HHL_glcm_MCC | wavelet-HHL_glcm_MaximumProbability |
|  |  | wavelet-HHL_glrlm_LongRunHighGrayLevelEmphasis | wavelet-HHL_glrlm_RunEntropy | wavelet-HHL_glszm_GrayLevelNonUniformityNormalized |
|  |  | wavelet-HHL_glszm_SizeZoneNonUniformityNormalized | wavelet-HHL_glszm_SmallAreaEmphasis | wavelet-HHL_glszm_SmallAreaLowGrayLevelEmphasis |
|  |  | wavelet-HHL_glszm_ZoneEntropy | wavelet-HHH_firstorder_Maximum | wavelet-HHH_firstorder_Median |
|  |  | wavelet-HHH_firstorder_Minimum | wavelet-HHH_firstorder_Range | wavelet-HHH_firstorder_Uniformity |
|  |  | wavelet-HHH_glcm_MCC | wavelet-HHH_glszm_GrayLevelNonUniformity | wavelet-HHH_glszm_GrayLevelNonUniformityNormalized |
|  |  | wavelet-HHH_glszm_LowGrayLevelZoneEmphasis | wavelet-HHH_glszm_SizeZoneNonUniformityNormalized | wavelet-HHH_glszm_SmallAreaEmphasis |
|  |  | wavelet-HHH_glszm_ZoneEntropy | wavelet-HHH_ngtdm_Contrast | wavelet-LLL_firstorder_Median |
|  |  | wavelet-LLL_firstorder_Skewness | wavelet-LLL_glcm_Correlation | wavelet-LLL_glcm_DifferenceVariance |
|  |  | wavelet-LLL_glcm_Id | wavelet-LLL_glcm_Idm | wavelet-LLL_glcm_Imc1 |
|  |  | wavelet-LLL_gldm_SmallDependenceHighGrayLevelEmphasis | wavelet-LLL_glrlm_RunLengthNonUniformityNormalized | wavelet-LLL_glszm_GrayLevelNonUniformityNormalized |
|  |  | wavelet-LLL_glszm_SmallAreaEmphasis |  |  |
