# Supplementary material for: Clinical radiomics-based machine learning versus three-dimension convolutional neural network analysis for differentiation of thymic epithelial tumors from other prevascular mediastinal tumors on chest computed tomography scan
Source: Front Oncol. 2023 Apr 18;13:1105100. doi: 10.3389/fonc.2023.1105100 (PMC10151670; doi:10.3389/fonc.2023.1105100)
Supplement: Supplementary file 5 [file Table_5.docx]

**Supplementary Table 5. The result of Bayesian optimization of different models and various feature selection in UECT**

|  |  | **Macro F1-Score** | **Macro Precision** | **Macro Recall** | **Accuracy** | **ROC-AUC** |
| --- | --- | --- | --- | --- | --- | --- |
| CatBoost | All | 0.7788 | 0.8597 | 0.7433 | 0.8743 | 0.8877 |
|  | Selection_20 | 0.7824 | 0.8565 | 0.7469 | 0.8745 | 0.8967 |
|  | Selection_19 | 0.7873 | 0.8476 | 0.7560 | 0.8743 | 0.9029 |
|  | Selection_18 | 0.7748 | 0.8406 | 0.7447 | 0.8680 | 0.8973 |
|  | Selection_17 | 0.7728 | 0.8472 | 0.7399 | 0.8690 | 0.9000 |
|  | Selection_16 | 0.7850 | 0.8610 | 0.7538 | 0.8765 | 0.9060 |
|  | Selection_15 | 0.7881 | 0.8560 | 0.7556 | 0.8765 | 0.9026 |
|  | Selection_14 | 0.7861 | 0.8508 | 0.7549 | 0.8755 | 0.9013 |
|  | Selection_13 | 0.7804 | 0.8418 | 0.7504 | 0.8712 | 0.9108 |
|  | Selection_12 | 0.7884 | 0.8597 | 0.7544 | 0.8776 | 0.9076 |
|  | Selection_11 | 0.8006 | 0.8706 | 0.7658 | 0.8839 | 0.9012 |
|  | Selection_10 | 0.7844 | 0.8481 | 0.7524 | 0.8744 | 0.8988 |
|  | Selection_9 | 0.7792 | 0.8488 | 0.7456 | 0.8723 | 0.9012 |
|  | Selection_8 | 0.8020 | 0.8554 | 0.7717 | 0.8818 | 0.9032 |
|  | Selection_7 | 0.7802 | 0.8554 | 0.7462 | 0.8733 | 0.9034 |
|  | Selection_6 | 0.7950 | 0.8561 | 0.7642 | 0.8787 | 0.9056 |
|  | Selection_5 | 0.7996 | 0.8608 | 0.7692 | 0.8808 | 0.9117 |
|  | Selection_4 | 0.8121 | 0.8686 | 0.7813 | 0.8882 | 0.9108 |
|  | Selection_3 | 0.8021 | 0.8425 | 0.7795 | 0.8797 | 0.9105 |
|  | Selection_2 | 0.7872 | 0.8389 | 0.7620 | 0.8722 | 0.8960 |
|  | Selection_1 | 0.7927 | 0.8403 | 0.7659 | 0.8755 | 0.8921 |
| ExtraTree with Entropy | All | 0.6284 | 0.8116 | 0.6094 | 0.8244 | 0.8326 |
|  | Selection_20 | 0.6298 | 0.8696 | 0.6091 | 0.8298 | 0.8472 |
|  | Selection_19 | 0.6081 | 0.8140 | 0.5950 | 0.8191 | 0.8434 |
|  | Selection_18 | 0.6289 | 0.8596 | 0.6103 | 0.8286 | 0.8397 |
|  | Selection_17 | 0.6140 | 0.8420 | 0.6003 | 0.8244 | 0.8420 |
|  | Selection_16 | 0.6068 | 0.8248 | 0.5939 | 0.8202 | 0.8355 |
|  | Selection_15 | 0.6093 | 0.8526 | 0.5959 | 0.8234 | 0.8401 |
|  | Selection_14 | 0.6191 | 0.8611 | 0.6034 | 0.8265 | 0.8449 |
|  | Selection_13 | 0.6435 | 0.8688 | 0.6209 | 0.8340 | 0.8498 |
|  | Selection_12 | 0.6486 | 0.9018 | 0.6236 | 0.8382 | 0.8551 |
|  | Selection_11 | 0.6545 | 0.8954 | 0.6280 | 0.8393 | 0.8575 |
|  | Selection_10 | 0.6824 | 0.9201 | 0.6475 | 0.8500 | 0.8808 |
|  | Selection_9 | 0.7146 | 0.9030 | 0.6748 | 0.8585 | 0.8967 |
|  | Selection_8 | 0.7506 | 0.9118 | 0.7048 | 0.8712 | 0.9081 |
|  | Selection_7 | 0.6694 | 0.9188 | 0.6400 | 0.8468 | 0.8946 |
|  | Selection_6 | 0.6824 | 0.9150 | 0.6493 | 0.8500 | 0.8985 |
|  | Selection_5 | 0.6913 | 0.9080 | 0.6561 | 0.8521 | 0.8981 |
|  | Selection_4 | 0.7061 | 0.9074 | 0.6680 | 0.8564 | 0.8960 |
|  | Selection_3 | 0.7234 | 0.8691 | 0.6869 | 0.8574 | 0.9006 |
|  | Selection_2 | 0.7763 | 0.8713 | 0.7385 | 0.8756 | 0.8926 |
|  | Selection_1 | 0.7921 | 0.8519 | 0.7606 | 0.8786 | 0.8780 |
| ExtraTree with Gini | All | 0.6282 | 0.7774 | 0.6090 | 0.8180 | 0.8247 |
|  | Selection_20 | 0.5977 | 0.8175 | 0.5882 | 0.8170 | 0.8309 |
|  | Selection_19 | 0.5993 | 0.8212 | 0.5901 | 0.8170 | 0.8298 |
|  | Selection_18 | 0.5995 | 0.8269 | 0.5907 | 0.8181 | 0.8363 |
|  | Selection_17 | 0.5993 | 0.8286 | 0.5907 | 0.8181 | 0.8347 |
|  | Selection_16 | 0.6141 | 0.8282 | 0.6001 | 0.8212 | 0.8252 |
|  | Selection_15 | 0.6201 | 0.8407 | 0.6039 | 0.8244 | 0.8250 |
|  | Selection_14 | 0.6046 | 0.8361 | 0.5939 | 0.8202 | 0.8349 |
|  | Selection_13 | 0.5976 | 0.8180 | 0.5875 | 0.8159 | 0.8397 |
|  | Selection_12 | 0.6350 | 0.8607 | 0.6153 | 0.8308 | 0.8442 |
|  | Selection_11 | 0.6416 | 0.8747 | 0.6191 | 0.8340 | 0.8504 |
|  | Selection_10 | 0.6927 | 0.9215 | 0.6550 | 0.8531 | 0.8809 |
|  | Selection_9 | 0.7253 | 0.9118 | 0.6830 | 0.8628 | 0.8940 |
|  | Selection_8 | 0.7430 | 0.9137 | 0.6980 | 0.8691 | 0.9041 |
|  | Selection_7 | 0.6784 | 0.9196 | 0.6450 | 0.8489 | 0.8874 |
|  | Selection_6 | 0.6933 | 0.9164 | 0.6568 | 0.8532 | 0.8955 |
|  | Selection_5 | 0.6948 | 0.9084 | 0.6586 | 0.8531 | 0.8982 |
|  | Selection_4 | 0.7153 | 0.8982 | 0.6748 | 0.8585 | 0.8957 |
|  | Selection_3 | 0.6994 | 0.8653 | 0.6657 | 0.8500 | 0.9028 |
|  | Selection_2 | 0.7740 | 0.8653 | 0.7371 | 0.8734 | 0.8860 |
|  | Selection_1 | 0.7985 | 0.8595 | 0.7663 | 0.8818 | 0.8778 |
| KNN with Distance Weights | All | 0.6128 | 0.6599 | 0.6015 | 0.7861 | 0.6884 |
|  | Selection_20 | 0.5362 | 0.5546 | 0.5361 | 0.7350 | 0.5422 |
|  | Selection_19 | 0.5362 | 0.5546 | 0.5361 | 0.7350 | 0.5422 |
|  | Selection_18 | 0.5362 | 0.5546 | 0.5361 | 0.7350 | 0.5422 |
|  | Selection_17 | 0.5362 | 0.5546 | 0.5361 | 0.7350 | 0.5422 |
|  | Selection_16 | 0.5148 | 0.5337 | 0.5186 | 0.7275 | 0.5392 |
|  | Selection_15 | 0.5103 | 0.5200 | 0.5131 | 0.7159 | 0.5324 |
|  | Selection_14 | 0.5103 | 0.5200 | 0.5131 | 0.7159 | 0.5324 |
|  | Selection_13 | 0.5103 | 0.5200 | 0.5131 | 0.7159 | 0.5324 |
|  | Selection_12 | 0.5098 | 0.5205 | 0.5124 | 0.7148 | 0.5329 |
|  | Selection_11 | 0.5098 | 0.5205 | 0.5124 | 0.7148 | 0.5338 |
|  | Selection_10 | 0.5098 | 0.5205 | 0.5124 | 0.7148 | 0.5338 |
|  | Selection_9 | 0.5729 | 0.6105 | 0.5668 | 0.7660 | 0.6122 |
|  | Selection_8 | 0.5643 | 0.5858 | 0.5601 | 0.7469 | 0.6009 |
|  | Selection_7 | 0.5643 | 0.5858 | 0.5601 | 0.7469 | 0.6001 |
|  | Selection_6 | 0.5255 | 0.5484 | 0.5260 | 0.7276 | 0.5175 |
|  | Selection_5 | 0.5163 | 0.5350 | 0.5183 | 0.7213 | 0.5188 |
|  | Selection_4 | 0.5163 | 0.5350 | 0.5183 | 0.7213 | 0.5164 |
|  | Selection_3 | 0.5200 | 0.5537 | 0.5270 | 0.7521 | 0.5624 |
|  | Selection_2 | 0.5200 | 0.5537 | 0.5270 | 0.7521 | 0.5624 |
|  | Selection_1 | 0.5200 | 0.5537 | 0.5270 | 0.7521 | 0.5624 |
| KNN with Uniform Weights | All | 0.6135 | 0.6623 | 0.6015 | 0.7861 | 0.6903 |
|  | Selection_20 | 0.5149 | 0.5453 | 0.5218 | 0.7468 | 0.5468 |
|  | Selection_19 | 0.5149 | 0.5453 | 0.5218 | 0.7468 | 0.5468 |
|  | Selection_18 | 0.5149 | 0.5453 | 0.5218 | 0.7468 | 0.5468 |
|  | Selection_17 | 0.5149 | 0.5453 | 0.5218 | 0.7468 | 0.5468 |
|  | Selection_16 | 0.4927 | 0.5205 | 0.5063 | 0.7425 | 0.5435 |
|  | Selection_15 | 0.5035 | 0.5312 | 0.5123 | 0.7405 | 0.5362 |
|  | Selection_14 | 0.5035 | 0.5312 | 0.5123 | 0.7405 | 0.5362 |
|  | Selection_13 | 0.5035 | 0.5312 | 0.5123 | 0.7405 | 0.5362 |
|  | Selection_12 | 0.5061 | 0.5341 | 0.5141 | 0.7405 | 0.5372 |
|  | Selection_11 | 0.5061 | 0.5341 | 0.5141 | 0.7405 | 0.5387 |
|  | Selection_10 | 0.5061 | 0.5341 | 0.5141 | 0.7405 | 0.5387 |
|  | Selection_9 | 0.5534 | 0.6191 | 0.5525 | 0.7722 | 0.5954 |
|  | Selection_8 | 0.5362 | 0.5845 | 0.5389 | 0.7595 | 0.5927 |
|  | Selection_7 | 0.5362 | 0.5845 | 0.5389 | 0.7595 | 0.5922 |
|  | Selection_6 | 0.5347 | 0.5832 | 0.5408 | 0.7595 | 0.5578 |
|  | Selection_5 | 0.5353 | 0.5852 | 0.5408 | 0.7595 | 0.5635 |
|  | Selection_4 | 0.5353 | 0.5852 | 0.5408 | 0.7595 | 0.5618 |
|  | Selection_3 | 0.5105 | 0.5972 | 0.5272 | 0.7755 | 0.5708 |
|  | Selection_2 | 0.5105 | 0.5972 | 0.5272 | 0.7755 | 0.5708 |
|  | Selection_1 | 0.5105 | 0.5972 | 0.5272 | 0.7755 | 0.5708 |
| LightGBM | All | 0.8095 | 0.8510 | 0.7852 | 0.8829 | 0.8976 |
|  | Selection_20 | 0.8035 | 0.8377 | 0.7825 | 0.8787 | 0.9059 |
|  | Selection_19 | 0.8015 | 0.8404 | 0.7789 | 0.8787 | 0.9056 |
|  | Selection_18 | 0.8096 | 0.8420 | 0.7882 | 0.8819 | 0.9001 |
|  | Selection_17 | 0.8072 | 0.8419 | 0.7857 | 0.8808 | 0.8957 |
|  | Selection_16 | 0.8040 | 0.8390 | 0.7825 | 0.8786 | 0.8999 |
|  | Selection_15 | 0.8110 | 0.8449 | 0.7888 | 0.8829 | 0.8988 |
|  | Selection_14 | 0.8147 | 0.8469 | 0.7938 | 0.8850 | 0.9038 |
|  | Selection_13 | 0.7960 | 0.8303 | 0.7743 | 0.8744 | 0.8988 |
|  | Selection_12 | 0.8054 | 0.8388 | 0.7832 | 0.8797 | 0.8960 |
|  | Selection_11 | 0.8025 | 0.8344 | 0.7837 | 0.8776 | 0.9013 |
|  | Selection_10 | 0.7997 | 0.8295 | 0.7816 | 0.8744 | 0.8868 |
|  | Selection_9 | 0.8123 | 0.8528 | 0.7884 | 0.8851 | 0.8931 |
|  | Selection_8 | 0.8156 | 0.8530 | 0.7927 | 0.8861 | 0.8993 |
|  | Selection_7 | 0.8129 | 0.8558 | 0.7891 | 0.8861 | 0.8996 |
|  | Selection_6 | 0.8114 | 0.8344 | 0.7966 | 0.8808 | 0.8985 |
|  | Selection_5 | 0.8140 | 0.8428 | 0.7961 | 0.8829 | 0.9045 |
|  | Selection_4 | 0.8044 | 0.8376 | 0.7854 | 0.8776 | 0.8998 |
|  | Selection_3 | 0.8015 | 0.8275 | 0.7871 | 0.8744 | 0.9017 |
|  | Selection_2 | 0.8030 | 0.8240 | 0.7944 | 0.8743 | 0.8942 |
|  | Selection_1 | 0.8258 | 0.8586 | 0.8052 | 0.8914 | 0.8855 |
| LightGBM with Extra Tree | All | 0.8189 | 0.8676 | 0.7917 | 0.8903 | 0.9022 |
|  | Selection_20 | 0.8299 | 0.8671 | 0.8072 | 0.8946 | 0.9215 |
|  | Selection_19 | 0.8210 | 0.8632 | 0.7954 | 0.8904 | 0.9236 |
|  | Selection_18 | 0.8209 | 0.8647 | 0.7954 | 0.8903 | 0.9150 |
|  | Selection_17 | 0.8236 | 0.8778 | 0.7937 | 0.8935 | 0.9153 |
|  | Selection_16 | 0.8130 | 0.8543 | 0.7883 | 0.8850 | 0.9159 |
|  | Selection_15 | 0.8252 | 0.8703 | 0.7992 | 0.8936 | 0.9183 |
|  | Selection_14 | 0.8100 | 0.8542 | 0.7840 | 0.8840 | 0.9167 |
|  | Selection_13 | 0.8153 | 0.8539 | 0.7926 | 0.8861 | 0.9171 |
|  | Selection_12 | 0.8181 | 0.8606 | 0.7940 | 0.8882 | 0.9205 |
|  | Selection_11 | 0.8168 | 0.8499 | 0.7963 | 0.8861 | 0.9175 |
|  | Selection_10 | 0.8302 | 0.8610 | 0.8102 | 0.8935 | 0.9070 |
|  | Selection_9 | 0.8303 | 0.8669 | 0.8072 | 0.8946 | 0.9127 |
|  | Selection_8 | 0.8298 | 0.8632 | 0.8083 | 0.8935 | 0.9112 |
|  | Selection_7 | 0.8272 | 0.8674 | 0.8029 | 0.8936 | 0.9065 |
|  | Selection_6 | 0.8353 | 0.8743 | 0.8111 | 0.8978 | 0.9112 |
|  | Selection_5 | 0.8395 | 0.8773 | 0.8160 | 0.8999 | 0.9117 |
|  | Selection_4 | 0.8246 | 0.8604 | 0.8026 | 0.8903 | 0.9076 |
|  | Selection_3 | 0.8250 | 0.8511 | 0.8075 | 0.8893 | 0.9157 |
|  | Selection_2 | 0.8206 | 0.8470 | 0.8036 | 0.8861 | 0.9017 |
|  | Selection_1 | 0.8247 | 0.8508 | 0.8075 | 0.8893 | 0.9008 |
| NeuralNetFastAI | All | 0.7353 | 0.7348 | 0.7387 | 0.8211 | 0.8012 |
|  | Selection_20 | 0.7542 | 0.7655 | 0.7525 | 0.8372 | 0.8523 |
|  | Selection_19 | 0.7696 | 0.7834 | 0.7652 | 0.8457 | 0.8565 |
|  | Selection_18 | 0.7780 | 0.7933 | 0.7742 | 0.8543 | 0.8527 |
|  | Selection_17 | 0.7743 | 0.7824 | 0.7721 | 0.8480 | 0.8540 |
|  | Selection_16 | 0.7718 | 0.7784 | 0.7701 | 0.8478 | 0.8586 |
|  | Selection_15 | 0.7728 | 0.7785 | 0.7720 | 0.8478 | 0.8623 |
|  | Selection_14 | 0.7709 | 0.7876 | 0.7677 | 0.8468 | 0.8550 |
|  | Selection_13 | 0.7706 | 0.7819 | 0.7654 | 0.8489 | 0.8535 |
|  | Selection_12 | 0.7880 | 0.7981 | 0.7831 | 0.8595 | 0.8654 |
|  | Selection_11 | 0.8010 | 0.8186 | 0.7924 | 0.8712 | 0.8824 |
|  | Selection_10 | 0.8067 | 0.8216 | 0.8056 | 0.8690 | 0.8961 |
|  | Selection_9 | 0.8330 | 0.8350 | 0.8376 | 0.8851 | 0.9026 |
|  | Selection_8 | 0.8159 | 0.8180 | 0.8181 | 0.8744 | 0.8889 |
|  | Selection_7 | 0.8078 | 0.8170 | 0.8028 | 0.8733 | 0.8857 |
|  | Selection_6 | 0.8133 | 0.8150 | 0.8184 | 0.8722 | 0.8921 |
|  | Selection_5 | 0.8240 | 0.8256 | 0.8299 | 0.8787 | 0.8993 |
|  | Selection_4 | 0.8258 | 0.8356 | 0.8193 | 0.8850 | 0.8706 |
|  | Selection_3 | 0.8213 | 0.8303 | 0.8155 | 0.8819 | 0.8814 |
|  | Selection_2 | 0.7920 | 0.8119 | 0.7907 | 0.8573 | 0.8630 |
|  | Selection_1 | 0.7870 | 0.7919 | 0.8060 | 0.8467 | 0.8938 |
| RandomForest with Entropy | All | 0.7267 | 0.8196 | 0.6949 | 0.8499 | 0.8620 |
|  | Selection_20 | 0.6737 | 0.8223 | 0.6474 | 0.8382 | 0.8726 |
|  | Selection_19 | 0.6695 | 0.8183 | 0.6417 | 0.8350 | 0.8737 |
|  | Selection_18 | 0.6538 | 0.8280 | 0.6305 | 0.8319 | 0.8624 |
|  | Selection_17 | 0.6471 | 0.8040 | 0.6248 | 0.8286 | 0.8624 |
|  | Selection_16 | 0.6704 | 0.8134 | 0.6435 | 0.8350 | 0.8690 |
|  | Selection_15 | 0.6674 | 0.8256 | 0.6398 | 0.8350 | 0.8701 |
|  | Selection_14 | 0.6647 | 0.8197 | 0.6374 | 0.8340 | 0.8753 |
|  | Selection_13 | 0.6614 | 0.8032 | 0.6353 | 0.8307 | 0.8747 |
|  | Selection_12 | 0.6795 | 0.8283 | 0.6499 | 0.8393 | 0.8723 |
|  | Selection_11 | 0.6739 | 0.8250 | 0.6448 | 0.8372 | 0.8805 |
|  | Selection_10 | 0.7621 | 0.8628 | 0.7271 | 0.8690 | 0.8792 |
|  | Selection_9 | 0.7879 | 0.8579 | 0.7544 | 0.8776 | 0.8859 |
|  | Selection_8 | 0.7952 | 0.8569 | 0.7631 | 0.8797 | 0.8923 |
|  | Selection_7 | 0.7473 | 0.8559 | 0.7104 | 0.8627 | 0.8879 |
|  | Selection_6 | 0.7325 | 0.8456 | 0.6971 | 0.8563 | 0.8874 |
|  | Selection_5 | 0.7421 | 0.8620 | 0.7048 | 0.8627 | 0.8942 |
|  | Selection_4 | 0.7792 | 0.8647 | 0.7440 | 0.8754 | 0.8893 |
|  | Selection_3 | 0.7836 | 0.8402 | 0.7547 | 0.8723 | 0.8980 |
|  | Selection_2 | 0.7946 | 0.8387 | 0.7702 | 0.8765 | 0.8858 |
|  | Selection_1 | 0.7932 | 0.8247 | 0.7730 | 0.8723 | 0.8794 |
| RandomForest with Gini | All | 0.7464 | 0.8072 | 0.7181 | 0.8520 | 0.8560 |
|  | Selection_20 | 0.7054 | 0.8082 | 0.6785 | 0.8414 | 0.8610 |
|  | Selection_19 | 0.7161 | 0.8350 | 0.6858 | 0.8499 | 0.8563 |
|  | Selection_18 | 0.6889 | 0.8027 | 0.6613 | 0.8372 | 0.8487 |
|  | Selection_17 | 0.6736 | 0.7873 | 0.6474 | 0.8297 | 0.8554 |
|  | Selection_16 | 0.6820 | 0.7860 | 0.6560 | 0.8319 | 0.8547 |
|  | Selection_15 | 0.7097 | 0.8131 | 0.6799 | 0.8435 | 0.8555 |
|  | Selection_14 | 0.6836 | 0.7921 | 0.6574 | 0.8339 | 0.8622 |
|  | Selection_13 | 0.7141 | 0.8130 | 0.6831 | 0.8456 | 0.8606 |
|  | Selection_12 | 0.7108 | 0.8059 | 0.6817 | 0.8435 | 0.8555 |
|  | Selection_11 | 0.7186 | 0.8218 | 0.6881 | 0.8478 | 0.8670 |
|  | Selection_10 | 0.7886 | 0.8449 | 0.7585 | 0.8754 | 0.8740 |
|  | Selection_9 | 0.7965 | 0.8405 | 0.7708 | 0.8775 | 0.8813 |
|  | Selection_8 | 0.7964 | 0.8395 | 0.7720 | 0.8765 | 0.8846 |
|  | Selection_7 | 0.7844 | 0.8593 | 0.7495 | 0.8755 | 0.8820 |
|  | Selection_6 | 0.7755 | 0.8579 | 0.7401 | 0.8722 | 0.8820 |
|  | Selection_5 | 0.7737 | 0.8609 | 0.7390 | 0.8733 | 0.8873 |
|  | Selection_4 | 0.7984 | 0.8567 | 0.7685 | 0.8797 | 0.8866 |
|  | Selection_3 | 0.7854 | 0.8310 | 0.7625 | 0.8702 | 0.8929 |
|  | Selection_2 | 0.7799 | 0.8139 | 0.7609 | 0.8648 | 0.8816 |
|  | Selection_1 | 0.7971 | 0.8244 | 0.7791 | 0.8734 | 0.8770 |
| WeightedEnsemble_L2 | All | 0.8156 | 0.8565 | 0.7915 | 0.8871 | 0.8869 |
|  | Selection_20 | 0.8047 | 0.8526 | 0.7779 | 0.8829 | 0.9094 |
|  | Selection_19 | 0.8244 | 0.8692 | 0.7974 | 0.8935 | 0.9144 |
|  | Selection_18 | 0.8044 | 0.8563 | 0.7784 | 0.8809 | 0.9034 |
|  | Selection_17 | 0.8111 | 0.8444 | 0.7888 | 0.8829 | 0.9022 |
|  | Selection_16 | 0.7899 | 0.8295 | 0.7668 | 0.8712 | 0.9048 |
|  | Selection_15 | 0.8002 | 0.8354 | 0.7793 | 0.8766 | 0.9057 |
|  | Selection_14 | 0.7978 | 0.8359 | 0.7762 | 0.8745 | 0.9044 |
|  | Selection_13 | 0.8049 | 0.8451 | 0.7820 | 0.8807 | 0.9067 |
|  | Selection_12 | 0.8157 | 0.8579 | 0.7897 | 0.8872 | 0.9119 |
|  | Selection_11 | 0.8145 | 0.8527 | 0.7938 | 0.8850 | 0.9064 |
|  | Selection_10 | 0.8241 | 0.8616 | 0.7997 | 0.8914 | 0.9014 |
|  | Selection_9 | 0.8328 | 0.8605 | 0.8145 | 0.8946 | 0.9038 |
|  | Selection_8 | 0.8183 | 0.8480 | 0.8011 | 0.8850 | 0.9026 |
|  | Selection_7 | 0.8287 | 0.8490 | 0.8155 | 0.8904 | 0.8910 |
|  | Selection_6 | 0.8285 | 0.8639 | 0.8084 | 0.8936 | 0.9038 |
|  | Selection_5 | 0.8172 | 0.8473 | 0.8005 | 0.8840 | 0.8983 |
|  | Selection_4 | 0.8077 | 0.8444 | 0.7875 | 0.8807 | 0.9049 |
|  | Selection_3 | 0.8030 | 0.8348 | 0.7854 | 0.8775 | 0.9018 |
|  | Selection_2 | 0.7989 | 0.8370 | 0.7841 | 0.8754 | 0.8884 |
|  | Selection_1 | 0.8109 | 0.8441 | 0.7925 | 0.8829 | 0.8858 |
| XGBoost | All | 0.7740 | 0.8045 | 0.7576 | 0.8595 | 0.8767 |
|  | Selection_20 | 0.7813 | 0.8192 | 0.7598 | 0.8659 | 0.8923 |
|  | Selection_19 | 0.7823 | 0.8164 | 0.7628 | 0.8649 | 0.8878 |
|  | Selection_18 | 0.7794 | 0.8111 | 0.7614 | 0.8627 | 0.8850 |
|  | Selection_17 | 0.7862 | 0.8142 | 0.7689 | 0.8658 | 0.8827 |
|  | Selection_16 | 0.7706 | 0.7990 | 0.7537 | 0.8563 | 0.8850 |
|  | Selection_15 | 0.7933 | 0.8209 | 0.7771 | 0.8701 | 0.8870 |
|  | Selection_14 | 0.7812 | 0.8167 | 0.7609 | 0.8648 | 0.8892 |
|  | Selection_13 | 0.7895 | 0.8253 | 0.7680 | 0.8701 | 0.8911 |
|  | Selection_12 | 0.7733 | 0.8080 | 0.7546 | 0.8605 | 0.8898 |
|  | Selection_11 | 0.7791 | 0.8106 | 0.7614 | 0.8627 | 0.8889 |
|  | Selection_10 | 0.7731 | 0.8046 | 0.7539 | 0.8595 | 0.8796 |
|  | Selection_9 | 0.7812 | 0.8137 | 0.7609 | 0.8649 | 0.8796 |
|  | Selection_8 | 0.7905 | 0.8311 | 0.7668 | 0.8712 | 0.8846 |
|  | Selection_7 | 0.7935 | 0.8324 | 0.7736 | 0.8733 | 0.8857 |
|  | Selection_6 | 0.7996 | 0.8257 | 0.7845 | 0.8732 | 0.8914 |
|  | Selection_5 | 0.8014 | 0.8273 | 0.7852 | 0.8743 | 0.8899 |
|  | Selection_4 | 0.7896 | 0.8181 | 0.7739 | 0.8680 | 0.8918 |
|  | Selection_3 | 0.7953 | 0.8093 | 0.7885 | 0.8680 | 0.8951 |
|  | Selection_2 | 0.7758 | 0.7951 | 0.7647 | 0.8563 | 0.8857 |
|  | Selection_1 | 0.8061 | 0.8305 | 0.7891 | 0.8775 | 0.8767 |
