# Supplementary material for: Clinical radiomics-based machine learning versus three-dimension convolutional neural network analysis for differentiation of thymic epithelial tumors from other prevascular mediastinal tumors on chest computed tomography scan
Source: Front Oncol. 2023 Apr 18;13:1105100. doi: 10.3389/fonc.2023.1105100 (PMC10151670; doi:10.3389/fonc.2023.1105100)
Supplement: Supplementary file 6 [file Table_6.docx]

**Supplementary Table 6. The result of Bayesian optimization of different models and various feature selection in CECT**

|  | | **Macro F1-Score** | **Macro Precision** | **Macro Recall** | **Accuracy** | **ROC-AUC** |
| --- | --- | --- | --- | --- | --- | --- |
| CatBoost | All | 0.7444 | 0.8485 | 0.7097 | 0.8630 | 0.9070 |
|  | Selection_20 | 0.7888 | 0.8676 | 0.7533 | 0.8813 | 0.9289 |
|  | Selection_19 | 0.7810 | 0.8462 | 0.7492 | 0.8748 | 0.9296 |
|  | Selection_18 | 0.7998 | 0.8651 | 0.7690 | 0.8856 | 0.9296 |
|  | Selection_17 | 0.8151 | 0.8755 | 0.7857 | 0.8931 | 0.9335 |
|  | Selection_16 | 0.8098 | 0.8847 | 0.7751 | 0.8921 | 0.9274 |
|  | Selection_15 | 0.8069 | 0.8774 | 0.7772 | 0.8899 | 0.9291 |
|  | Selection_14 | 0.7940 | 0.8706 | 0.7613 | 0.8845 | 0.9325 |
|  | Selection_13 | 0.7896 | 0.8692 | 0.7561 | 0.8823 | 0.9280 |
|  | Selection_12 | 0.7922 | 0.8690 | 0.7579 | 0.8833 | 0.9256 |
|  | Selection_11 | 0.7846 | 0.8638 | 0.7516 | 0.8802 | 0.9280 |
|  | Selection_10 | 0.8043 | 0.8734 | 0.7715 | 0.8887 | 0.9368 |
|  | Selection_9 | 0.7957 | 0.8706 | 0.7627 | 0.8844 | 0.9341 |
|  | Selection_8 | 0.8249 | 0.8844 | 0.7928 | 0.8985 | 0.9341 |
|  | Selection_7 | 0.8112 | 0.8670 | 0.7801 | 0.8909 | 0.9354 |
|  | Selection_6 | 0.8093 | 0.8654 | 0.7801 | 0.8899 | 0.9380 |
|  | Selection_5 | 0.8311 | 0.8852 | 0.8020 | 0.9005 | 0.9418 |
|  | Selection_4 | 0.8374 | 0.8824 | 0.8111 | 0.9027 | 0.9428 |
|  | Selection_3 | 0.8286 | 0.8850 | 0.7983 | 0.8995 | 0.9354 |
|  | Selection_2 | 0.8148 | 0.8645 | 0.7869 | 0.8898 | 0.9349 |
|  | Selection_1 | 0.8311 | 0.8744 | 0.8063 | 0.8985 | 0.9402 |
| ExtraTrees with Entropy | All | 0.6567 | 0.8228 | 0.6343 | 0.8370 | 0.8558 |
|  | Selection_20 | 0.6554 | 0.8151 | 0.6312 | 0.8339 | 0.9001 |
|  | Selection_19 | 0.6629 | 0.8235 | 0.6369 | 0.8372 | 0.8987 |
|  | Selection_18 | 0.6449 | 0.8288 | 0.6221 | 0.8329 | 0.9053 |
|  | Selection_17 | 0.6535 | 0.8180 | 0.6292 | 0.8339 | 0.9043 |
|  | Selection_16 | 0.6550 | 0.8246 | 0.6296 | 0.8350 | 0.9006 |
|  | Selection_15 | 0.6136 | 0.8031 | 0.5988 | 0.8222 | 0.8843 |
|  | Selection_14 | 0.6140 | 0.8094 | 0.5988 | 0.8232 | 0.8851 |
|  | Selection_13 | 0.6028 | 0.7890 | 0.5908 | 0.8189 | 0.8828 |
|  | Selection_12 | 0.6220 | 0.8224 | 0.6056 | 0.8264 | 0.8925 |
|  | Selection_11 | 0.6274 | 0.8117 | 0.6099 | 0.8274 | 0.8852 |
|  | Selection_10 | 0.6076 | 0.7930 | 0.5952 | 0.8200 | 0.9023 |
|  | Selection_9 | 0.6437 | 0.8280 | 0.6215 | 0.8328 | 0.9059 |
|  | Selection_8 | 0.6545 | 0.8350 | 0.6290 | 0.8361 | 0.9119 |
|  | Selection_7 | 0.6657 | 0.8628 | 0.6380 | 0.8414 | 0.9199 |
|  | Selection_6 | 0.6856 | 0.8900 | 0.6529 | 0.8512 | 0.9230 |
|  | Selection_5 | 0.6986 | 0.8932 | 0.6637 | 0.8555 | 0.9314 |
|  | Selection_4 | 0.7507 | 0.8937 | 0.7083 | 0.8717 | 0.9345 |
|  | Selection_3 | 0.7818 | 0.9075 | 0.7356 | 0.8845 | 0.9398 |
|  | Selection_2 | 0.7575 | 0.8525 | 0.7205 | 0.8684 | 0.9327 |
|  | Selection_1 | 0.7960 | 0.8747 | 0.7595 | 0.8856 | 0.9372 |
| ExtraTrees with Gini | All | 0.6765 | 0.8077 | 0.6513 | 0.8381 | 0.8429 |
|  | Selection_20 | 0.6551 | 0.8151 | 0.6309 | 0.8339 | 0.8922 |
|  | Selection_19 | 0.6450 | 0.7959 | 0.6272 | 0.8338 | 0.8917 |
|  | Selection_18 | 0.6503 | 0.8217 | 0.6268 | 0.8328 | 0.8961 |
|  | Selection_17 | 0.6526 | 0.8167 | 0.6287 | 0.8339 | 0.8957 |
|  | Selection_16 | 0.6400 | 0.8142 | 0.6200 | 0.8306 | 0.8967 |
|  | Selection_15 | 0.5822 | 0.7597 | 0.5767 | 0.8091 | 0.8769 |
|  | Selection_14 | 0.6432 | 0.8096 | 0.6210 | 0.8306 | 0.8745 |
|  | Selection_13 | 0.6149 | 0.7928 | 0.5989 | 0.8210 | 0.8749 |
|  | Selection_12 | 0.6086 | 0.7951 | 0.5956 | 0.8209 | 0.8808 |
|  | Selection_11 | 0.6207 | 0.8131 | 0.6048 | 0.8253 | 0.8727 |
|  | Selection_10 | 0.6235 | 0.8070 | 0.6077 | 0.8243 | 0.8830 |
|  | Selection_9 | 0.6302 | 0.7942 | 0.6117 | 0.8253 | 0.8998 |
|  | Selection_8 | 0.6444 | 0.8306 | 0.6207 | 0.8329 | 0.9033 |
|  | Selection_7 | 0.6631 | 0.8604 | 0.6351 | 0.8403 | 0.9158 |
|  | Selection_6 | 0.6853 | 0.8871 | 0.6532 | 0.8500 | 0.9167 |
|  | Selection_5 | 0.6948 | 0.8890 | 0.6605 | 0.8533 | 0.9281 |
|  | Selection_4 | 0.7431 | 0.8928 | 0.7031 | 0.8695 | 0.9272 |
|  | Selection_3 | 0.7596 | 0.8932 | 0.7171 | 0.8748 | 0.9343 |
|  | Selection_2 | 0.7536 | 0.8537 | 0.7164 | 0.8672 | 0.9293 |
|  | Selection_1 | 0.7981 | 0.8781 | 0.7602 | 0.8867 | 0.9387 |
| KNN with Distance Weights | All | 0.6344 | 0.7036 | 0.6189 | 0.8059 | 0.7427 |
|  | Selection_20 | 0.5548 | 0.6011 | 0.5532 | 0.7695 | 0.5977 |
|  | Selection_19 | 0.5308 | 0.5741 | 0.5348 | 0.7630 | 0.5923 |
|  | Selection_18 | 0.5308 | 0.5741 | 0.5348 | 0.7630 | 0.5924 |
|  | Selection_17 | 0.5308 | 0.5741 | 0.5348 | 0.7630 | 0.5924 |
|  | Selection_16 | 0.5308 | 0.5741 | 0.5348 | 0.7630 | 0.5924 |
|  | Selection_15 | 0.5308 | 0.5741 | 0.5348 | 0.7630 | 0.5924 |
|  | Selection_14 | 0.5308 | 0.5741 | 0.5348 | 0.7630 | 0.5924 |
|  | Selection_13 | 0.5308 | 0.5741 | 0.5348 | 0.7630 | 0.5924 |
|  | Selection_12 | 0.5169 | 0.5699 | 0.5273 | 0.7661 | 0.5605 |
|  | Selection_11 | 0.5169 | 0.5699 | 0.5273 | 0.7661 | 0.5605 |
|  | Selection_10 | 0.5169 | 0.5699 | 0.5273 | 0.7661 | 0.5605 |
|  | Selection_9 | 0.5176 | 0.5856 | 0.5297 | 0.7716 | 0.5316 |
|  | Selection_8 | 0.5061 | 0.5478 | 0.5181 | 0.7553 | 0.5557 |
|  | Selection_7 | 0.5061 | 0.5478 | 0.5181 | 0.7553 | 0.5558 |
|  | Selection_6 | 0.4966 | 0.4991 | 0.5001 | 0.6992 | 0.5628 |
|  | Selection_5 | 0.4966 | 0.4991 | 0.5001 | 0.6992 | 0.5628 |
|  | Selection_4 | 0.4966 | 0.4991 | 0.5001 | 0.6992 | 0.5628 |
|  | Selection_3 | 0.5322 | 0.5411 | 0.5318 | 0.7166 | 0.4847 |
|  | Selection_2 | 0.5322 | 0.5411 | 0.5318 | 0.7166 | 0.4847 |
|  | Selection_1 | 0.5531 | 0.5908 | 0.5525 | 0.7597 | 0.5889 |
| KNN with Uniform Weights | All | 0.6257 | 0.6865 | 0.6129 | 0.7985 | 0.7423 |
|  | Selection_20 | 0.5209 | 0.5735 | 0.5290 | 0.7651 | 0.5858 |
|  | Selection_19 | 0.5139 | 0.5640 | 0.5241 | 0.7629 | 0.5806 |
|  | Selection_18 | 0.5139 | 0.5640 | 0.5241 | 0.7629 | 0.5801 |
|  | Selection_17 | 0.5139 | 0.5640 | 0.5241 | 0.7629 | 0.5801 |
|  | Selection_16 | 0.5139 | 0.5640 | 0.5241 | 0.7629 | 0.5801 |
|  | Selection_15 | 0.5139 | 0.5640 | 0.5241 | 0.7629 | 0.5801 |
|  | Selection_14 | 0.5139 | 0.5640 | 0.5241 | 0.7629 | 0.5801 |
|  | Selection_13 | 0.5139 | 0.5640 | 0.5241 | 0.7629 | 0.5801 |
|  | Selection_12 | 0.5095 | 0.5829 | 0.5245 | 0.7727 | 0.5626 |
|  | Selection_11 | 0.5095 | 0.5829 | 0.5245 | 0.7727 | 0.5626 |
|  | Selection_10 | 0.5095 | 0.5829 | 0.5245 | 0.7727 | 0.5626 |
|  | Selection_9 | 0.5061 | 0.5790 | 0.5235 | 0.7737 | 0.5357 |
|  | Selection_8 | 0.4926 | 0.5261 | 0.5107 | 0.7575 | 0.5392 |
|  | Selection_7 | 0.4926 | 0.5261 | 0.5107 | 0.7575 | 0.5392 |
|  | Selection_6 | 0.4887 | 0.5249 | 0.5041 | 0.7431 | 0.5835 |
|  | Selection_5 | 0.4887 | 0.5249 | 0.5041 | 0.7431 | 0.5835 |
|  | Selection_4 | 0.4887 | 0.5249 | 0.5041 | 0.7431 | 0.5835 |
|  | Selection_3 | 0.5492 | 0.6274 | 0.5502 | 0.7780 | 0.5248 |
|  | Selection_2 | 0.5492 | 0.6274 | 0.5502 | 0.7780 | 0.5248 |
|  | Selection_1 | 0.5764 | 0.6646 | 0.5713 | 0.7857 | 0.5988 |
| LightGBM | All | 0.7993 | 0.8461 | 0.7740 | 0.8802 | 0.9294 |
|  | Selection_20 | 0.8274 | 0.8653 | 0.8063 | 0.8954 | 0.9419 |
|  | Selection_19 | 0.8237 | 0.8618 | 0.8026 | 0.8932 | 0.9404 |
|  | Selection_18 | 0.8241 | 0.8627 | 0.8030 | 0.8941 | 0.9419 |
|  | Selection_17 | 0.8205 | 0.8626 | 0.7968 | 0.8921 | 0.9416 |
|  | Selection_16 | 0.8133 | 0.8539 | 0.7907 | 0.8878 | 0.9357 |
|  | Selection_15 | 0.8143 | 0.8525 | 0.7928 | 0.8877 | 0.9378 |
|  | Selection_14 | 0.8009 | 0.8500 | 0.7753 | 0.8823 | 0.9374 |
|  | Selection_13 | 0.8165 | 0.8611 | 0.7908 | 0.8899 | 0.9373 |
|  | Selection_12 | 0.8118 | 0.8537 | 0.7906 | 0.8867 | 0.9378 |
|  | Selection_11 | 0.8146 | 0.8513 | 0.7951 | 0.8878 | 0.9375 |
|  | Selection_10 | 0.8091 | 0.8446 | 0.7888 | 0.8845 | 0.9410 |
|  | Selection_9 | 0.8237 | 0.8574 | 0.8037 | 0.8931 | 0.9429 |
|  | Selection_8 | 0.8082 | 0.8388 | 0.7896 | 0.8825 | 0.9412 |
|  | Selection_7 | 0.8183 | 0.8506 | 0.7993 | 0.8888 | 0.9418 |
|  | Selection_6 | 0.8218 | 0.8504 | 0.8043 | 0.8909 | 0.9428 |
|  | Selection_5 | 0.8436 | 0.8677 | 0.8296 | 0.9028 | 0.9460 |
|  | Selection_4 | 0.8337 | 0.8655 | 0.8132 | 0.8985 | 0.9480 |
|  | Selection_3 | 0.8448 | 0.8693 | 0.8292 | 0.9028 | 0.9504 |
|  | Selection_2 | 0.8533 | 0.8806 | 0.8360 | 0.9081 | 0.9448 |
|  | Selection_1 | 0.8501 | 0.8715 | 0.8389 | 0.9060 | 0.9461 |
| LightGBM with ExtraTree | All | 0.7901 | 0.8365 | 0.7637 | 0.8758 | 0.9257 |
|  | Selection_20 | 0.8430 | 0.8818 | 0.8182 | 0.9060 | 0.9432 |
|  | Selection_19 | 0.8388 | 0.8833 | 0.8124 | 0.9039 | 0.9411 |
|  | Selection_18 | 0.8389 | 0.8784 | 0.8146 | 0.9028 | 0.9433 |
|  | Selection_17 | 0.8485 | 0.8850 | 0.8246 | 0.9082 | 0.9405 |
|  | Selection_16 | 0.8305 | 0.8804 | 0.8023 | 0.8996 | 0.9387 |
|  | Selection_15 | 0.8259 | 0.8684 | 0.7989 | 0.8964 | 0.9334 |
|  | Selection_14 | 0.8273 | 0.8814 | 0.7972 | 0.8995 | 0.9380 |
|  | Selection_13 | 0.8199 | 0.8653 | 0.7940 | 0.8932 | 0.9367 |
|  | Selection_12 | 0.8396 | 0.8867 | 0.8128 | 0.9040 | 0.9403 |
|  | Selection_11 | 0.8310 | 0.8852 | 0.8004 | 0.9007 | 0.9335 |
|  | Selection_10 | 0.8380 | 0.8777 | 0.8130 | 0.9017 | 0.9390 |
|  | Selection_9 | 0.8348 | 0.8771 | 0.8080 | 0.9007 | 0.9425 |
|  | Selection_8 | 0.8494 | 0.8938 | 0.8223 | 0.9092 | 0.9447 |
|  | Selection_7 | 0.8427 | 0.8767 | 0.8224 | 0.9029 | 0.9438 |
|  | Selection_6 | 0.8484 | 0.8769 | 0.8290 | 0.9060 | 0.9404 |
|  | Selection_5 | 0.8483 | 0.8801 | 0.8278 | 0.9061 | 0.9468 |
|  | Selection_4 | 0.8565 | 0.8889 | 0.8353 | 0.9115 | 0.9464 |
|  | Selection_3 | 0.8554 | 0.8861 | 0.8345 | 0.9104 | 0.9468 |
|  | Selection_2 | 0.8448 | 0.8809 | 0.8216 | 0.9050 | 0.9424 |
|  | Selection_1 | 0.8348 | 0.8634 | 0.8152 | 0.8974 | 0.9389 |
| NeuralNetFastAI | All | 0.7420 | 0.7374 | 0.7562 | 0.8222 | 0.8141 |
|  | Selection_20 | 0.7939 | 0.7943 | 0.8054 | 0.8542 | 0.8926 |
|  | Selection_19 | 0.8037 | 0.8056 | 0.8051 | 0.8694 | 0.9059 |
|  | Selection_18 | 0.8070 | 0.8104 | 0.8085 | 0.8715 | 0.9173 |
|  | Selection_17 | 0.7943 | 0.7952 | 0.7996 | 0.8606 | 0.8987 |
|  | Selection_16 | 0.8117 | 0.8130 | 0.8158 | 0.8735 | 0.9117 |
|  | Selection_15 | 0.7861 | 0.7912 | 0.7831 | 0.8607 | 0.9019 |
|  | Selection_14 | 0.7999 | 0.7968 | 0.8064 | 0.8650 | 0.9011 |
|  | Selection_13 | 0.7878 | 0.7906 | 0.7936 | 0.8585 | 0.9054 |
|  | Selection_12 | 0.8047 | 0.8093 | 0.8174 | 0.8630 | 0.9092 |
|  | Selection_11 | 0.8203 | 0.8231 | 0.8221 | 0.8813 | 0.9239 |
|  | Selection_10 | 0.8293 | 0.8385 | 0.8246 | 0.8899 | 0.9191 |
|  | Selection_9 | 0.8447 | 0.8514 | 0.8409 | 0.8987 | 0.9305 |
|  | Selection_8 | 0.8266 | 0.8322 | 0.8296 | 0.8858 | 0.9208 |
|  | Selection_7 | 0.8388 | 0.8351 | 0.8564 | 0.8854 | 0.9177 |
|  | Selection_6 | 0.8444 | 0.8458 | 0.8509 | 0.8953 | 0.9274 |
|  | Selection_5 | 0.8338 | 0.8293 | 0.8491 | 0.8857 | 0.9327 |
|  | Selection_4 | 0.8187 | 0.8223 | 0.8301 | 0.8762 | 0.9354 |
|  | Selection_3 | 0.8371 | 0.8503 | 0.8318 | 0.8942 | 0.9060 |
|  | Selection_2 | 0.7974 | 0.7983 | 0.8043 | 0.8631 | 0.9052 |
|  | Selection_1 | 0.8025 | 0.8027 | 0.8147 | 0.8653 | 0.9256 |
| RandomForest with Entropy | All | 0.7020 | 0.8088 | 0.6736 | 0.8435 | 0.8779 |
|  | Selection_20 | 0.7361 | 0.8503 | 0.7002 | 0.8607 | 0.9095 |
|  | Selection_19 | 0.7423 | 0.8432 | 0.7098 | 0.8628 | 0.9120 |
|  | Selection_18 | 0.7332 | 0.8382 | 0.6990 | 0.8587 | 0.9107 |
|  | Selection_17 | 0.7395 | 0.8526 | 0.7039 | 0.8619 | 0.9075 |
|  | Selection_16 | 0.7349 | 0.8614 | 0.6989 | 0.8630 | 0.9063 |
|  | Selection_15 | 0.6638 | 0.8245 | 0.6358 | 0.8361 | 0.8831 |
|  | Selection_14 | 0.6924 | 0.8490 | 0.6611 | 0.8490 | 0.8921 |
|  | Selection_13 | 0.6717 | 0.8290 | 0.6428 | 0.8403 | 0.8929 |
|  | Selection_12 | 0.6855 | 0.8342 | 0.6562 | 0.8436 | 0.8945 |
|  | Selection_11 | 0.6878 | 0.8216 | 0.6602 | 0.8435 | 0.8946 |
|  | Selection_10 | 0.7004 | 0.8475 | 0.6693 | 0.8490 | 0.9063 |
|  | Selection_9 | 0.7085 | 0.8368 | 0.6773 | 0.8510 | 0.9089 |
|  | Selection_8 | 0.7178 | 0.8468 | 0.6834 | 0.8553 | 0.9103 |
|  | Selection_7 | 0.7325 | 0.8633 | 0.6981 | 0.8618 | 0.9202 |
|  | Selection_6 | 0.7274 | 0.8615 | 0.6924 | 0.8596 | 0.9213 |
|  | Selection_5 | 0.7503 | 0.8637 | 0.7127 | 0.8671 | 0.9277 |
|  | Selection_4 | 0.8004 | 0.8901 | 0.7609 | 0.8899 | 0.9301 |
|  | Selection_3 | 0.8328 | 0.8932 | 0.8017 | 0.9039 | 0.9358 |
|  | Selection_2 | 0.8054 | 0.8681 | 0.7743 | 0.8877 | 0.9359 |
|  | Selection_1 | 0.8187 | 0.8555 | 0.7963 | 0.8908 | 0.9338 |
| RandomForest with Gini | All | 0.7252 | 0.8017 | 0.6976 | 0.8466 | 0.8689 |
|  | Selection_20 | 0.7733 | 0.8377 | 0.7420 | 0.8704 | 0.8991 |
|  | Selection_19 | 0.7763 | 0.8383 | 0.7462 | 0.8714 | 0.8977 |
|  | Selection_18 | 0.7768 | 0.8397 | 0.7462 | 0.8714 | 0.9021 |
|  | Selection_17 | 0.7820 | 0.8471 | 0.7512 | 0.8757 | 0.8991 |
|  | Selection_16 | 0.7743 | 0.8368 | 0.7440 | 0.8704 | 0.8976 |
|  | Selection_15 | 0.7127 | 0.8180 | 0.6846 | 0.8499 | 0.8761 |
|  | Selection_14 | 0.7250 | 0.8311 | 0.6938 | 0.8532 | 0.8755 |
|  | Selection_13 | 0.7051 | 0.8214 | 0.6747 | 0.8467 | 0.8753 |
|  | Selection_12 | 0.7251 | 0.8220 | 0.6964 | 0.8542 | 0.8867 |
|  | Selection_11 | 0.7379 | 0.8268 | 0.7078 | 0.8564 | 0.8857 |
|  | Selection_10 | 0.7461 | 0.8316 | 0.7150 | 0.8596 | 0.8921 |
|  | Selection_9 | 0.7582 | 0.8296 | 0.7285 | 0.8640 | 0.8956 |
|  | Selection_8 | 0.7430 | 0.8319 | 0.7131 | 0.8596 | 0.9045 |
|  | Selection_7 | 0.7667 | 0.8616 | 0.7322 | 0.8725 | 0.9106 |
|  | Selection_6 | 0.7651 | 0.8455 | 0.7327 | 0.8682 | 0.9101 |
|  | Selection_5 | 0.7819 | 0.8674 | 0.7470 | 0.8790 | 0.9181 |
|  | Selection_4 | 0.8173 | 0.8740 | 0.7857 | 0.8941 | 0.9221 |
|  | Selection_3 | 0.8344 | 0.8892 | 0.8029 | 0.9027 | 0.9252 |
|  | Selection_2 | 0.8265 | 0.8773 | 0.7961 | 0.8974 | 0.9236 |
|  | Selection_1 | 0.8300 | 0.8631 | 0.8092 | 0.8973 | 0.9270 |
| WeightedEnsemble_L2 | All | 0.7867 | 0.8323 | 0.7608 | 0.8737 | 0.9164 |
|  | Selection_20 | 0.8369 | 0.8706 | 0.8175 | 0.9007 | 0.9443 |
|  | Selection_19 | 0.8413 | 0.8782 | 0.8193 | 0.9038 | 0.9389 |
|  | Selection_18 | 0.8293 | 0.8667 | 0.8086 | 0.8963 | 0.9401 |
|  | Selection_17 | 0.8266 | 0.8682 | 0.8028 | 0.8952 | 0.9372 |
|  | Selection_16 | 0.8318 | 0.8692 | 0.8091 | 0.8986 | 0.9418 |
|  | Selection_15 | 0.8298 | 0.8630 | 0.8096 | 0.8964 | 0.9372 |
|  | Selection_14 | 0.8282 | 0.8758 | 0.8031 | 0.8985 | 0.9402 |
|  | Selection_13 | 0.8216 | 0.8686 | 0.7992 | 0.8942 | 0.9404 |
|  | Selection_12 | 0.8326 | 0.8626 | 0.8143 | 0.8963 | 0.9428 |
|  | Selection_11 | 0.8423 | 0.8758 | 0.8207 | 0.9039 | 0.9403 |
|  | Selection_10 | 0.8229 | 0.8545 | 0.8035 | 0.8920 | 0.9377 |
|  | Selection_9 | 0.8256 | 0.8588 | 0.8065 | 0.8931 | 0.9379 |
|  | Selection_8 | 0.8308 | 0.8537 | 0.8164 | 0.8942 | 0.9352 |
|  | Selection_7 | 0.8470 | 0.8684 | 0.8341 | 0.9028 | 0.9427 |
|  | Selection_6 | 0.8369 | 0.8654 | 0.8215 | 0.8985 | 0.9418 |
|  | Selection_5 | 0.8506 | 0.8763 | 0.8341 | 0.9072 | 0.9491 |
|  | Selection_4 | 0.8472 | 0.8767 | 0.8287 | 0.9061 | 0.9458 |
|  | Selection_3 | 0.8481 | 0.8804 | 0.8281 | 0.9070 | 0.9461 |
|  | Selection_2 | 0.8419 | 0.8627 | 0.8279 | 0.9005 | 0.9422 |
|  | Selection_1 | 0.8265 | 0.8545 | 0.8100 | 0.8941 | 0.9358 |
| XGBoost | All | 0.7756 | 0.8070 | 0.7598 | 0.8641 | 0.9137 |
|  | Selection_20 | 0.8069 | 0.8307 | 0.7921 | 0.8814 | 0.9300 |
|  | Selection_19 | 0.8041 | 0.8258 | 0.7905 | 0.8792 | 0.9289 |
|  | Selection_18 | 0.7997 | 0.8247 | 0.7859 | 0.8771 | 0.9310 |
|  | Selection_17 | 0.8025 | 0.8203 | 0.7921 | 0.8771 | 0.9267 |
|  | Selection_16 | 0.8071 | 0.8348 | 0.7910 | 0.8824 | 0.9298 |
|  | Selection_15 | 0.7868 | 0.8135 | 0.7718 | 0.8706 | 0.9277 |
|  | Selection_14 | 0.8001 | 0.8351 | 0.7823 | 0.8803 | 0.9274 |
|  | Selection_13 | 0.8039 | 0.8365 | 0.7864 | 0.8824 | 0.9317 |
|  | Selection_12 | 0.8061 | 0.8381 | 0.7883 | 0.8825 | 0.9329 |
|  | Selection_11 | 0.7960 | 0.8212 | 0.7806 | 0.8750 | 0.9298 |
|  | Selection_10 | 0.8239 | 0.8615 | 0.8066 | 0.8942 | 0.9299 |
|  | Selection_9 | 0.8235 | 0.8444 | 0.8124 | 0.8900 | 0.9303 |
|  | Selection_8 | 0.8166 | 0.8358 | 0.8061 | 0.8857 | 0.9276 |
|  | Selection_7 | 0.8373 | 0.8542 | 0.8284 | 0.8976 | 0.9357 |
|  | Selection_6 | 0.8314 | 0.8517 | 0.8213 | 0.8944 | 0.9330 |
|  | Selection_5 | 0.8366 | 0.8553 | 0.8267 | 0.8975 | 0.9363 |
|  | Selection_4 | 0.8383 | 0.8565 | 0.8267 | 0.8975 | 0.9319 |
|  | Selection_3 | 0.8496 | 0.8601 | 0.8432 | 0.9029 | 0.9366 |
|  | Selection_2 | 0.8453 | 0.8657 | 0.8323 | 0.9028 | 0.9395 |
|  | Selection_1 | 0.8490 | 0.8631 | 0.8390 | 0.9039 | 0.9379 |
